# Late‐Stage ^18^F‐Difluoromethyl Labeling of N‐Heteroaromatics with High Molar Activity for PET Imaging

**DOI:** 10.1002/anie.201907488

**Published:** 2019-08-09

**Authors:** Laura Trump, Agostinho Lemos, Bénédicte Lallemand, Patrick Pasau, Joël Mercier, Christian Lemaire, André Luxen, Christophe Genicot

**Affiliations:** ^1^ Global Chemistry, UCB NewMedicines UCB Biopharma sprl 1420 Braine-l'Alleud Belgium; ^2^ GIGA-CRC In Vivo Imaging Cyclotron Research Center-B30 Université de Liège Quartier Agora, 6 allée du six août 4000 Liège Belgium

**Keywords:** C−H activation, flow chemistry, fluorine, photochemistry, radiochemistry

## Abstract

Despite a growing interest in CHF_2_ in medicinal chemistry, there is a lack of efficient methods for the insertion of CHF^18^F into druglike compounds. Herein described is a photoredox flow reaction for ^18^F‐difluoromethylation of N‐heteroaromatics that are widely used in medicinal chemistry. Following the two‐step synthesis for a new ^18^F‐difluoromethylation reagent, the photoredox reaction is completed within two minutes and proceeds by C−H activation, circumventing the need for pre‐functionalization of the substrate. The method is operationally simple and affords straightforward access to radiolabeled N‐heteroaromatics with high molar activity suitable for biological in vivo studies and clinical application.

## Graphical abstract

**Make a trace**: A new flow photoredox methodology allows the ^18^F‐difluoromethylation of heteroaromatics by C−H activation. The method is applicable to a wide range of heteroaromatics and can be used for the preparation of radiotracers with high molar activities that are suitable for positron emission tomography studies.

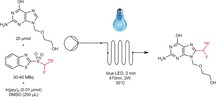

## Introduction

**Figure 1:**
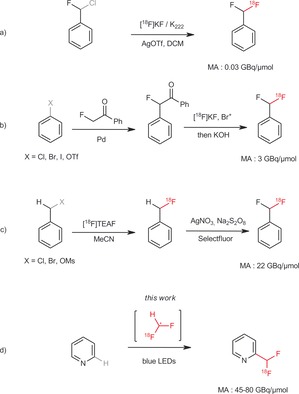
Existing methodologies for the introduction of CHF^18^F. a) Halex exchange.7a b) Prefunctionnalization and subsequent ^18^F‐fluorination.7b c) Prefunctionnalization and subsequent Halex and addition of a fluorine with selectfluor.7c d) Direct ^18^F difluoromethylation by C−H activation.

PET (positron emission tomography)^1^ is a powerful non‐invasive imaging technology to evaluate disease states (diagnosis) and support the clinical development of new drug candidates. PET is the method of choice to measure the biodistribution of a drug and the receptor occupancy of a molecule in the central nervous system (CNS), enabling a more accurate assessment of the efficacy of a drug. The use of PET relies on the preparation of labeled compounds. Carbon‐11^2^ or fluorine‐18 are the most commonly used isotopes for the synthesis and development of new radiotracers. Synthetic methodologies developed for the preparation of PET tracers should be compatible with the short half‐life of these radioisotopes. Because of a favorable half life of 110 minutes (vs. 20 min for carbon‐11) and the importance of fluorine and fluorinated groups such as CF_3_ and CHF_2_ in medicinal chemistry, fluorine‐18 is a very attractive isotope for PET studies. SNAr substitution reactions with [^18^F]fluoride is still the most prevalent method for the labeling of aromatics. This method is however limited to reactive electron‐deficient aromatics. Recently, a number of new synthetic methodologies have been developed for the incorporation of fluorine‐18 into electron‐neutral and electron‐rich aromatics to overcome this limitation, expanding significantly the chemical space accessible to radiolabeling.3a–3c Good progress has also been made for the introduction of other important fluorinated groups such as CF_3_.4a–4c Recently, there has been a growing interest in CHF_2_ in medicinal chemistry. The difluoromethyl group (CHF_2_) is less lipophilic than the trifluoromethyl one and has been successfully used to block oxidative metabolism because of aldehyde oxidase.^5^ In addition, CHF_2_ can serve as a hydrogen‐bond donor and thus can act as a lipophilic bioisoster of the hydroxy group.6a, 6b To the best of our knowledge, only three methodologies for the incorporation of CHF^18^F into druglike compounds have been reported (Figure 1). Two of them are applicable mainly to aromatics and require pre‐functionalization of the substrates.7a, 7b A limitation of these methods is the low to moderate molar activity obtained, and could make clinical application challenging. The third approach utilizes an oxidative benzylic fluorination of a pre‐labeled aromatic substrate and has not been exemplified on N‐heteroaromatics.7c In spite of these encouraging results, there is still a clear need to devise new methods to introduce CHF^18^F on drug‐like scaffolds typically found in medicinal chemistry programs. We reasoned that the use of a radical CHF^18^F could offer a straightforward way to label N‐heteroaromatics through C−H functionalization and overcome the tedious synthesis of pre‐functionalized substrates. Our aim was to produce the radical CHF^18^F through a photoredox reaction in flow to ensure optimal irradiation of the reaction mixture and a fast reaction time.

Several reagents have been described for the C−H functionalization of heteroaromatics with CHF_2_.8a–8c Based on the seminal contribution of Langlois8d, 8e on the radical trifluoromethylation of aromatics, Baran and co‐workers first disclosed the use of Zn(SO_2_CHF_2_)_2_ for C−H difluoromethylation of N‐heteroaromatics.8f More recently, the group of Maruoka reported that hypervalent iodonium reagents prepared from difluoroacetic acid are effective for the addition of CHF_2_ on heteroaromatics.8g The group of Nielsen disclosed a redox process utilizing difluoroacetic acid as a direct source of CHF_2_.8h A new and complementary method was published during the preparation of this manuscript describing an oxidative C−H difluoromethylation with copper complexes generated in situ from TMSCHF_2_.8i

However, all these described reagents are not available yet for fluorine‐18 application, either because they are not easy to label, or because they are difficult to purify and analyze (such as anionic compounds).

We envisioned that the benzothiazole sulfone reagent, described by Hu and co‐workers9a, 9b for the addition of CHF_2_ to alkenes, coupled to cyclization would serve our purpose well. The CHF_2_ radical can be produced under neutral conditions through a catalytic photoredox reaction. In addition, the opportunity to introduce fluorine‐18 through a halogen exchange (Halex) reaction and the excellent solubility of this reagent in organic solvent, as well as its easy purification, are attractive for PET chemistry. Below, we describe the labeling of Hu's reagent, as well as a novel methodology for the CHF^18^F labeling of N‐heteroaromatics under neutral conditions by a photoredox reaction in flow chemistry.

## Results and Discussion

We first turned our attention to the preparation of the labeled benzothiazole sulfone [^18^F]**3** (Table 1). The first step (the labeling step) was the introduction of no carrier added (nca) [^18^F]‐fluoride by a Halex reaction. Using K[^18^F]F, K_222_, and K_2_CO_3_ in MeCN at 120 °C, [^18^F]**2** was obtained in 15.2±0.3 % radiochemical yield (RCY; entry 4). Unless otherwise specified, all the RCYs are based on HPLC and TLC analysis of the crude reaction mixture (see the Supporting Information). Lower temperature slightly decreased the RCY. Conducting the reaction in either DMSO or DCE either did not give the desired product or led to a lower RCY (entries 1 and 2). Increasing the quantity of **1**, or replacement of the base, did not afford any improvement (entries 5 and 6). [^18^F]**2** was directly engaged in the second step. Oxidation of [^18^F]**2** to the corresponding sulfone [^18^F]**3** using RuCl_3_, *x* H_2_O, and NaIO_4_ in water on a ^t^C18 cartridge proceeded readily and quantitatively with a RCY of 13.4±0.4 % over the two steps (entry 7). No catalyst resulted in no reaction, and a lower amount of oxidant translated into a lower RCY (entries 8 and 9). After semi‐preparative HPLC purification, the molar activity of [^18^F]**3** was 81.4±11.1 GBq μmol^−1^ [2.2±0.3 Ci μmol^−1^, decay corrected (dc)] at the end‐of‐bombardment (EOB), affording one of the best molar activities for CHF^18^F addition. The duration of the synthesis of [^18^F]**3**, including HPLC purification was about 45 minutes. With these encouraging results in hand, we started the exploration of the reaction of [^18^F]**3** with N‐heteroaromatics in the presence of a photoredox catalyst.

**Table 1:** Synthesis of [^18^F]**3**. 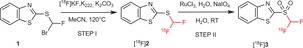

Entry	Reaction Step	Deviation from standard reaction conditions^[a,b]^	RCY [%] (of the crude mixture)
**1**	I	85 °C, DCE	7.2±0.5
**2**	I	85 °C, DMSO	0
**3**	I	85 °C	12.7±0.2
**4**	I	–	15.2±0.3
**5**	I	1 (80 μmol)	5.9±2.4
**6**	I	Et_4_N^+^HCO_3_^‐^	12.8±1.1
**7**	II	–	13.4±0.4
**8**	II	NaIO_4_ (0.12 mmol)	9.6±0.9
**9**	II	No RuCl_3_, *x* H_2_O	0.2

[a] Standard reaction conditions for I: K[^18^F]F (100–150 MBq), **1** (40 μmol), K_222_ (10 μmol), K_2_CO_3_ (20 μmol), MeCN, 120 °C, 5 min, *n*=3. [b] Standard reaction conditions for II : NaIO_4_ (240 μmol), RuCl_3_⋅x H_2_O (80 μmol), H_2_O, RT, 5 min, *n*=3.

The ^18^F‐difluoromethylation reaction was conducted using flow chemistry to ensure a better irradiation of the solution and accelerate the reaction rate, since the reaction time is a crucial parameter for fluorine‐18 chemistry. The optimization of the photochemical reaction was performed on Acyclovir^10^ (**4**), an anti‐herpetic drug (Table 2). All reactions were done in triplicate with [^18^F]**3**, previously purified by semi preparative HPLC (PREP HPLC). Performing the reaction in DMSO with Ir(ppy)_3_ as a catalyst (0.01 μmol), under irradiation with blue LEDs (2 W, 470 nm) at 35 °C turned out to be optimal (entry 3). Under these reaction conditions, the desired difluoromethylated acyclovir [^18^F]**5** was formed in only 2 minutes with an excellent RCY of 70±7 % (*n*=7). A slightly higher temperature (55 °C vs. 35 °C, entry 5), or a lower reaction time (30 s, entry 6) decreased the RCY. Changing the solvent and switching from DMSO to DMF also decreased the RCY from 70 to 44 %. The low solubility of acyclovir in MeCN and DCE prevented the use of these solvents. Interestingly, running the reaction with 0.001 μmol of Ir catalyst still afforded [^18^F]**5** but in a lower RCY. In contrast, Ru(bpy)_3_ did not lead to any product at all, whereas benzophenone was less effective than the Ir(ppy)_3_ catalyst. It is worth mentioning that the presence of water (50 μL, entry 9) had a negative impact on the RCY. The use of dried DMSO and minimizing the presence of water within the reagent [^18^F]**3** is recommended (see the Supporting Information). After HPLC purification, [^18^F]**5** was obtained with a very satisfactory RCY of 42±4 %. Gratifyingly, the molar activity of [^18^F]**5** was 44.4±11.1 GBq μmol^−1^ (1.2±0.3 Ci μmol^−1^ dc) at the EOB (around 90 min for the three steps). The estimated RCY for the three‐step synthesis is 3–4 % (dc). The desired labeled product [^18^F]**5** was not formed without either a catalyst or light supporting the photoredox mechanism (entries 10 and 11). In addition, no reaction was observed with TEMPO (entry 12), suggesting that the reaction takes place through a radical mechanism. The mechanism of the reaction is likely to be similar to the one put forward by the group of MacMillan^11^ for C−H aromatic trifluoromethylation, and involves a CHF^18^F radical (see the Supporting Information).

**Table 2:** Optimization of the ^18^F‐difluoromethylation reaction. 
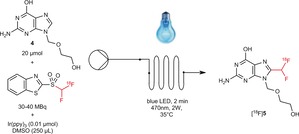

Entry	Deviation from standard reaction conditions	RCY [^18^F]**5** [%] (of the crude reaction mixture)
**1**	benzophenone^[a]^	47±5
**2**	Ru(bpy)_3_ (0.01 μmol)	0
**3**	–	70±7
**4**	After HPLC purification of [^18^F]5	42±4^[b]^
**5**	55 °C	51±10
**6**	30 s	60±8
**7**	Ir(ppy)_3_ (0.001 μmol)	42±2
**8**	DMF	44±1
**9**	DMSO/H_2_O (200/50 μL)	45±10
**10**	no catalyst	0
**11**	no light	0
**12**	TEMPO	0

[a] Benzophenone (10 μmol), 365 nm, [b] Radiochemical yield (*n*=4) of isolated product.

**Figure 2:**
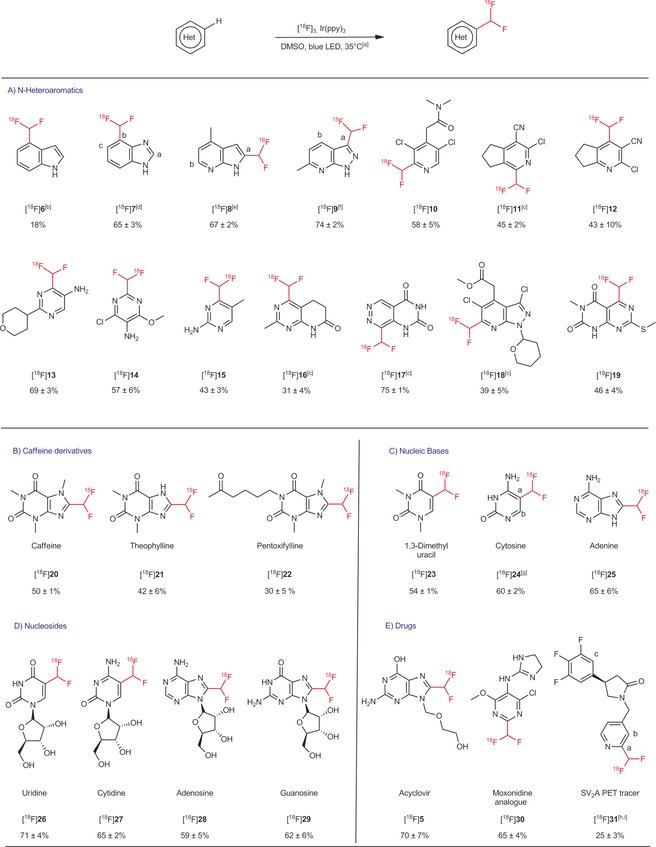
Scope of the ^18^F‐difluoromethylation reaction. [a] Standard reaction conditions: Substrate (20 μmol), [^18^F]**3** (30–40 MBq), Ir(ppy)_3_ (0.01 μmol)_,_ residence time (2 min), 35 °C, DMSO, 470 nm, *n*=3. [b] 0.05 μmol Ir(ppy)_3_. [c] 0.05 μmol Ir(ppy)_3_ and residence time of 4 min. [d] Ratio a/b/c=3:6:1. [e] Ratio a/b=90:10. [f] Ratio a/b=70:30. [g] Ratio not determined. [h] Ratio a/b/c=4:1:2. [i]  RCYs of isolated a: 4.2±0.3 % and b: 1.5±0.1 %.

With the optimal reaction conditions in hand, the scope of the photochemical reaction was investigated (Figure 2). Pleasingly, C−H ^18^F‐difluoromethylation can be carried out on a broad range of heteroaromatics such as indole, benzimidazole, azaindole, pyridine, pyrimidine. ^18^F‐difluoromethylated compounds were obtained in low to excellent RCYs (from 18 to 75 % RCY). A higher amount of catalyst and a longer residence time were sometimes needed to complete the reaction ([^18^F]**16**–[^18^F]**18**). It should be noted that more than one isomer was usually observed when several C−H bonds were available for functionalization. However, isomers were readily separated and isolated by PREP‐HPLC (see [^18^F]**31**). We also observed that some substrates in Figure 2 proved to be less reactive or even unreactive under stoichiometric batch conditions using fluorine‐19, but could be readily labeled using our flow chemistry conditions (see Section 3 in the Supporting Information). The methodology was used to label more complex scaffolds of medical interest. Xanthines such as caffeine, theophylline, and pentoxifylline turned out to be good substrates and were labelled with RCYs ranging from 30 to 65 % ([^18^F]**20**–[^18^F]**22**). The methodology could also be used to label nucleic acid bases such as uracil, cytosine, and adenine ([^18^F]**23**–[^18^F]**25**). Guanine could not be used in flow because of its limited solubility in DMSO. However, guanosine and other nucleosides such as uridine, cytidine, and adenosine ([^18^F]**26**–[^18^F]**29**) were readily labeled under standard reaction conditions.

To further demonstrate the benefit of the new methodology, the new procedure was applied to the preparation of the CHF^18^F analogue of moxonidine ([^18^F]**30**; Figure 2), and to the labeling of a new SV_2_A ligand ([^18^F]**31**). Moxonidine,^12^ a drug used for the treatment of hypertension is reported to exert its pharmacological effect through the imidazoline receptor. Starting from the precursor **36**(see Section 1d in the Supporting Information), CHF^18^F was readily introduced in place of the methyl group under the optimal reaction conditions described above. [^18^F]**30**was obtained with 65±4 % RCY.

**Figure 3:**
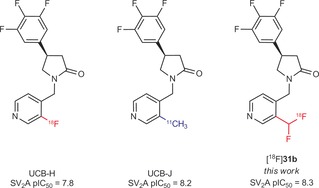
SV2A PET tracers.

SV_2_A is the molecular target of the antiepileptic drugs Keppra (Levetiracetam13a) and Briviact (Brivaracetam13b). SV_2_A is widely expressed in the brain and the potential of SV_2_A PET tracers such as UCB‐J^14^ (Figure 3) as biomarkers of synaptic density are currently being explored for CNS disorders including neurodegenerative diseases. The University of Liège in collaboration with UCB reported the synthesis and evaluation of the first ^18^F‐SV_2_A radiotracer UCB‐H.15a, 15b However, introduction of a fluorine in place of the methyl group on the pyridine slightly reduced the affinity for SV_2_A (Figure 3). Our objective was to investigate the direct introduction of CHF^18^F onto the pyridine in place of the methyl substituent known to be important to maintain high affinity for SV_2_A. Application of our procedure (unsubstituted pyridine, see the Supporting Information) gave a mixture of three isomers, [^18^F]**31 a,** [^18^F]**31 b**, and [^18^F]**31 c**, which were readily separated by HPLC. After separation, [^18^F]**31 b** was obtained with a RCY of 1.5 %.

In comparison to other reported procedures,16a, 16b our new methodology enables the direct labeling of the SV2A ligand (see Section 1d) and avoids the time and resource investment in chemistry for the preparation of a pre‐functionalized precursor. It provides access to a fluorine‐18 SV_2_A radiotracer with affinity similar to that of UCB‐J (Figure 3) and illustrates how the method can support rapid access to new PET tracers for biological evaluation.

## Conclusion

We have disclosed the development of a new methodology for the direct C−H ^18^F‐difluoromethylation of a wide range of N‐heteroaromatics. The method utilizes a new CHF^18^F reagent and a photoredox reaction17a–17c for the labeling of druglike motifs by C−H activation using a three‐step synthesis. Molar activity is an important parameter to consider when assessing the value and utility of a new labeling methods for PET imaging. The methodology described herein gives access to ^18^F‐labeled products with molar activities (44.4±11.1 GBq μmol^−1^ as shown with acyclovir) suitable for biological in vivo studies and clinical application, affording a significant advantage over existing methods. The protocol is operationally simple, and the use of a flow process for the photoredox reaction enables very short reaction time for the labeling step (less than 2 min). The reaction proceeds under mild reaction conditions and displays high functional‐group tolerance. Most functional groups used in medicinal chemistry, such as free amine, alcohol, nitrile, chlorine, amide and ether, as well as aminals in sugars appear to be compatible with reaction conditions. This method does not require pre‐functionalization of substrates and can be realized at the very last step of the synthesis as demonstrated by the late‐stage labeling of four drugs (acyclovir, theophylline, pentoxifylline, and a moxonidine analogue) and a SV_2_A ligand. Furthermore, our flow method holds potential for automation. The development of a fully automated process is currently underway and will be reported in due course. We anticipate that this novel methodology will be very useful for late‐stage labeling of druglike compounds and the discovery of new PET tracers.

## Experimental Section

General radiochemical procedure for ^18^F‐difluoromethylation. A solution of the substrate (20 μmol), Ir(ppy)_3_ (0.01 μmol) in DMSO (200 μL) was prepared. Then **[18F]3** in solution in DMSO (around 37 MBq/1mCi, 50 μL) was added. The solution was injected in a 100 μL microchip, pumped with DMSO at a flow rate of 50 μL min^−1^ (residence time of 2 min) and irradiated under blue LED (470 nm, 2 W), at a temperature of 35 °C. The exited solution was analyzed by Radio‐TLC and Radio‐UPLC for radiochemical yield (RCY) determination.

## Conflict of interest

The authors declare no conflict of interest.

## Supporting information

As a service to our authors and readers, this journal provides supporting information supplied by the authors. Such materials are peer reviewed and may be re‐organized for online delivery, but are not copy‐edited or typeset. Technical support issues arising from supporting information (other than missing files) should be addressed to the authors.

Supplementary

## References

[1] J. Mercier , L. Provins , J. Hannestad in Comprehensive Medicinal Chemistry III, Vol. 7.02 (Eds.: S. Chackalamannil, D. Rotella, E. W. Ward), Elsevier, Amsterdam, 2017, pp. 20–64.

[2] X. Deng , J. Rong , L. Wang , N. Vasdev , L. Zhang , L. Josephson , S. H. Liang , Angew. Chem. Int. Ed. 2019, 58, 2580. 10.1002/anie.201805501 PMC6405341 30054961

[3a] S. Preshlock , M. Tredwell , V. Gouverneur , Chem. Rev. 2016, 116, 719; 26751274 10.1021/acs.chemrev.5b00493

[3b] D. van der Born , A. Pees , A. J. Poot , R. V. A. Orru , A. D. Windhorst , D. J. Vugts , Chem. Soc. Rev. 2017, 46, 4709; 28608906 10.1039/c6cs00492j

[3c] H. H. Coenen , J. Ermert , J. Clin. Trans. Imaging. 2018, 6, 169.

[4a] M. Huiban , M. Tredwell , S. Mizuta , A. Wan , X. Zhang , L. T. Collier , V. Gouverneur , J. Passchier , Nat. Chem. 2013, 5, 941; 24153372 10.1038/nchem.1756

[4b] D. van der Born , C. Sewing , J. D. M. Herscheid , A. D. Windhorst , R. V. A. Orru , D. J. Vugts , Angew. Chem. Int. Ed. 2014, 53, 11046; 10.1002/anie.201406221 25155042

[4c] S. Verhoog , C. W. Kee , Y. Wang , T. Khotavivattana , T. C. Wilson , V. Kersemans , S. Smart , M. Tredwell , B. G. Davis , V. Gouverneur , J. Am. Chem. Soc. 2018, 140, 1572. 29301394 10.1021/jacs.7b10227

[5] F. O'Hara , A. C. Burns , M. R. Collins , D. Dalvie , M. A. Ornelas , A. D. N. Vaz , Y. Fujiwara , P. S. Baran , J. Med. Chem. 2014, 57, 1616. 24472070 10.1021/jm4017976 PMC3983350

[6a] Y. Zafrani , D. Yeffet , G. Sod-Moriah , A. Berliner , D. Amir , E. Gershonov , S. Saphier , J. Med. Chem. 2017, 60, 797; 28051859 10.1021/acs.jmedchem.6b01691

[6b] C. D. Sessler , M. Rahm , S. Becker , J. M. Goldberg , F. Wang , S. J. Lippard , J. Am. Chem. Soc. 2017, 139, 9325. 28576078 10.1021/jacs.7b04457 PMC6535088

[7a] S. Verhoog , L. Pfeifer , T. Khotavivattana , S. Calderwood , T. L. Collier , K. Wheelhouse , M. Tredwell , V. Gouverneur , Synlett 2016, 27, 25; 10.1002/anie.201504665 26140357

[7b] H. Shi , A. Braun , L. Wang , S. H. Liang , N. Vasdev , T. Ritter , Angew. Chem. Int. Ed. 2016, 55, 10786; 10.1002/anie.201604106 PMC5189681 27491349

[7c] G. Yuan , F. Wang , N. A. Stephenson , L. Wang , B. H. Rotstein , N. Vasdev , P. Tang , S. H. Liang , Chem. Commun. 2017, 53, 126. 10.1039/c6cc07913j PMC5179041 27917423

[8] For reviews on difluoromethylation, see:

[8a] J. Rong , C. Ni , J. Hu , Asian J. Chem. 2017, 6, 139;

[8b] D. E. Yerien , S. Barata-Vallejo , A. Postigo , Chem. Eur. J. 2017, 23, 14676; 28632338 10.1002/chem.201702311

[8c] A. Lemos , C. Lemaire , A. Luxen , Adv. Synth. Catal. 2019, 10.1002/adsc.201801121. *For specific publications* PMC6813635 31680791

[8d] Y. Ji , T. Bruecki , R. D. Baxter , Y. Fujiwara , I. B. Seiple , S. Su , D. G. Blackmond , P. S. Baran , Proc. Natl. Acad. Sci. USA 2011, 108, 14411; 21844378 10.1073/pnas.1109059108 PMC3167544

[8e] B. R. Langlois in Modern Synthesis Processes and Reactivity of Fluorinated Compounds (Eds.: H. Groult, F. R. Leroux, A. Tressaud), Elsevier, Amsterdam, 2017, chap. 5, pp. 125–140;

[8f] Y. Fujiwara , J. A. Dixon , R. A. Rodriguez , R. D. Baxter , D. D. Dixon , M. R. Collins , D. G. Blackmond , P. S. Baran , J. Am. Chem. Soc. 2012, 134, 1494; 22229949 10.1021/ja211422g PMC3269891

[8g] A. Sakamoto , H. Kashiwagi , K. Maruoka , Org. Lett. 2017, 19, 5126; 28898083 10.1021/acs.orglett.7b02416

[8h] T. T. Tung , S. B. Christensen , J. Nielsen , Chem. Eur. J. 2017, 23, 18125; 28945302 10.1002/chem.201704261

[8i] S. Zhu , Y. Liu , H. Li , X. Xu , F. Qing , J. Am. Chem. Soc. 2018, 140, 11613. 30179476 10.1021/jacs.8b08135

[9a] J. Rong , L. Deng , P. Tan , C. Ni , Y. Gu , J. Hu , Angew. Chem. Int. Ed. 2016, 55, 2743; 10.1002/anie.201510533 26797782

[9b] W. Fu , X. Han , M. Zhu , C. Xu , Z. Wang , B. Ji , X. Hao , M. Song , Chem. Commun. 2016, 52, 13413. 10.1039/c6cc07771d 27790647

[10] A. T. Dobson , B. B. Little , L. L. Scottie , Am. J. Obstet. Gynecol. 1998, 179, 527. 9731864 10.1016/s0002-9378(98)70390-4

[11] D. A. Nagib , D. W. C. MacMillan , Nature 2011, 480, 224. 22158245 10.1038/nature10647 PMC3310175

[12] C. Fenton , M. K. Keating , K. A. Lyseng-Williamson , Drugs 2006, 66, 477. 16597164 10.2165/00003495-200666040-00006

[13a] H. Klitgaard , P. Verdru , Expert Opin. Drug Discovery 2007, 2, 1537; 10.1517/17460441.2.11.1537 23484603

[13b] M. Gillard , B. Fuks , K. Leclercq , A. Matagne , Eur. J. Pharmacol. 2011, 664, 36. 21575627 10.1016/j.ejphar.2011.04.064

[14] N. B. Nabulsi , J. Mercier , D. Holden , S. Carré , S. Najafzadeh , M.-C. Vandergeten , S. Lin , A. Deo , N. Price , M. Wood , T. Lara-Jaime , F. Montel , M. Laruelle , R. E. Carson , J. Hannestad , Y. Huang , J. Nucl. Med. 2016, 57, 777. 26848175 10.2967/jnumed.115.168179

[15a] J. Mercier , L. Provins , A. Valade , Drug Discovery Today Technol. 2017, 25, 45; 10.1016/j.ddtec.2017.11.003 29233267

[15b] C. Warnier , C. Lemaire , G. Becker , G. Zaragoza , F. Giacomelli , J. Aerts , M. Otabashi , M. A. Bahri , J. Mercier , A. Plenevaux , A. Luxen , J. Med. Chem. 2016, 59, 8955. 27598384 10.1021/acs.jmedchem.6b00905

[16] During the development of our methodology, another ^18^F-labeled SV2A radiotracer with affinity comparable to that of UCB-H was disclosed by two different groups. In both cases, the tracer was labeled with fluorine-18 through aromatic nucleophilic substitution and required the preparation of a pre-functionalized precursors.

[16a] C. C. Constantinescu , C. Tresse , M. Zheng , A. Gouasmat , V. M. Caroll , L. Mistico , D. Alagille , C. M. Sandiego , C. Papin , K. Marek , J. P. Seibyl , G. D. Tamagnan , O. Barret , Mol. Imaging Biol. 2019, 10.1007/s11307-018-1260-5, 30084043

[16b] S. Li , Z. Cai , X. Wu , D. Holden , R. Pracitto , M. Kapinos , H. Gao , D. Labaree , N. Nabulsi , R. E. Carson , Y. Huang , ACS Chem. Neurosci. 2018, 10.1021/acschemneuro.8b00526. PMC6810685 30396272

[17] For recent publications on fluorine-18 photochemistry, see:

[17a] M. B. Nodwell , H. Yang , M. Colovic , Z. Yuan , H. Merkens , R. E. Martin , F. Bénard , P. Schaffer , R. Britton , J. Am. Chem. Soc. 2017, 139, 3595; 28248493 10.1021/jacs.6b11533

[17b] Z. Yuan , M. B. Nodwell , H. Yang , N. Malik , H. Merkens , F. Bénard , R. E. Martin , P. Schaffer , R. Britton , Angew. Chem. Int. Ed. 2018, 57, 12733; 10.1002/anie.201806966 30086209

[17c] W. Chen , Z. Huang , N. E. S. Tay , B. Giglio , M. Wang , H. Wang , Z. Wu , D. A. Nicewicz , Z. Li , Science 2019, 364, 1170. 31221856 10.1126/science.aav7019 PMC6680023

