# Supplementary material for: Late‐Stage 18F‐Difluoromethyl Labeling of N‐Heteroaromatics with High Molar Activity for PET Imaging
Source: Angew Chem Int Ed Engl. 2019 Aug 9;58(37):13149–54. doi: 10.1002/anie.201907488 (PMC6790700; doi:10.1002/anie.201907488)
Supplement: Supplementary file 1 — Supplementary [file ANIE-58-13149-s001.pdf]

Angewandte Chemie
Eine Zeitschrift der Gesellschaft Deutscher Chemiker

Supporting Information

Late-Stage ^{18}F-Difluoromethyl Labeling of N-Heteroaromatics with High Molar Activity for PET Imaging

Laura Trump, Agostinho Lemos, Bénédicte Lallemand, Patrick Pasau, Joël Mercier, Christian Lemaire, André Luxen, and Christophe Genicot**

anie_201907488_sm_miscellaneous_information.pdf

Table of Contents

1. Experimental procedures and analytical data S-4
   a. General Information S-4
   b. Synthesis of 3 .... S-5
   c. Difluoromethylation S-6
      i. General procedures S-6
      ii. Gradients used for Semi prep purification S-7
      iii. Scope S-11
   d. Synthesis of the different precursors S-25
      i. Synthesis of moxonidine analogue 36 .... S-25
      ii. Synthesis of SV2A-PET (39) S-26
2. Radioactive Chemistry S-26
   a. Generality S-26
   b. Automated radiosynthesis of [^{18}F]3 .... S-28
   c. Optimization of the labeling and oxidative steps S-29
   d. Isolation and determination of molar activity of the sulfone [^{18}F]3 .... S-32
   e. Optimization of the photochemical reaction S-33
   f. Isolation and Molar activity of CHF^{18}FAcyclovir [^{18}F]5 .... S-37
   g. Mechanistic studies S-38
   h. General procedure for ^{18}F-difluoromethylation S-38
   i. Scope S-39
3. Comparison batch fluorine-19/flow fluorine-18 conditions S-79
   a. General procedure for batch fluorine-19 conditions S-79
   b. Results S-80
4. References S-82
5. NMR spectra S-82
   a. ^{1}H, ^{13}C and ^{19}F NMR of 1 .... S-83
   b. ^{1}H, ^{13}C and ^{19}F NMR of 2 .... S-87
   c. ^{1}H, ^{13}C and ^{19}F NMR of 3 .... S-91
   d. ^{1}H, ^{13}C and ^{19}F NMR of 5 .... S-95
   e. ^{1}H, cosy, NOE, ^{13}C and ^{19}F NMR of 6 .... S-98
   f. ^{1}H, ^{13}C and ^{19}F NMR of 7a S-104
   g. ^{1}H, ^{13}C and ^{19}F NMR of 7b S-108
   h. ^{1}H, ^{13}C and ^{19}F NMR of 7c S-112
   i. ^{1}H, cosy, ^{13}C and ^{19}F NMR of 8a S-117
   j. ^{1}H, cosy,^{13}C and ^{19}F NMR of 8b S-122

k. ^{1}H, ^{13}C and ^{19}F NMR of 9a S-127
l. ^{1}H, ^{13}C and ^{19}F NMR of 9b S-130
m. ^{1}H, ^{13}C and ^{19}F NMR of 10 .... S-134
n. ^{1}H, ^{13}C and ^{19}F NMR of 11 .... S-138
o. ^{1}H, ^{13}C and ^{19}F NMR of 12 .... S-142
p. ^{1}H, ^{13}C and ^{19}F NMR of 13 .... S-146
q. ^{1}H, ^{13}C and ^{19}F NMR of 14 .... S-151
r. ^{1}H, ^{13}C and ^{19}F NMR of 15 .... S-155
s. ^{1}H, ^{13}C and ^{19}F NMR of 16 .... S-159
t. ^{1}H, ^{13}C and ^{19}F NMR of 17 .... S-163
u. ^{1}H, ^{13}C and ^{19}F NMR of 18 .... S-167
v. ^{1}H, ^{13}C and ^{19}F NMR of 19 .... S-171
w. ^{1}H, ^{13}C and ^{19}F NMR of 20 .... S-174
x. ^{1}H, ^{13}C and ^{19}F NMR of 21 .... S-177
y. ^{1}H, ^{13}C and ^{19}F NMR of 22 .... S-182
z. ^{1}H, ^{13}C and ^{19}F NMR of 23 .... S-186
aa. ^{1}H, HSQC, HMBC and ^{19}F NMR of 24a S-191
bb. ^{1}H, ^{13}C and ^{19}F NMR of 24b S-196
cc. ^{1}H, ^{13}C and ^{19}F NMR of 25 .... S-200
dd. ^{1}H, ^{13}C and ^{19}F NMR of 26 .... S-204
ee. ^{1}H, HSQC/HMBC and ^{19}F NMR of 27 .... S-208
ff. ^{1}H, ^{13}C and ^{19}F NMR of 28 .... S-213
gg. ^{1}H, ^{13}C and ^{19}F NMR of 29 .... S-217
hh. ^{1}H, ^{13}C and ^{19}F NMR of 30 .... S-221
ii. ^{1}H, ^{13}C and ^{19}F NMR of 31a S-225
jj. ^{1}H, HSCQ, HMBC and ^{19}F NMR of 31b S-229
kk. ^{1}H, HSCQ, HMBC and ^{19}F NMR of 31c S-234
ll. ^{1}H and ^{13}C NMR of 35 .... S-238
mm. ^{1}H and ^{13}C NMR of 36 .... S-241
nn. ^{1}H, ^{13}C and ^{19}F NMR of 39 .... S-244

1. Experimental procedures and analytical data

a. General Information

All reagents purchased from commercial sources were used as received. Technical solvents were bought from VWR international and used as received.

Flow reactions were performed on a R-series Vapourtec system, using a LED photoreactor (450 nm, 24 W, 10 mL coil reactor with FEP tubing).

UPLC analyses were run on a Waters system (Acquity UPLC® diode array detector (190–400 nm) controlled by the Empower software) and were performed using an ACQUITY UPLC® BEH C18 column (1.7 µm, 2.1 × 100 mm), at 0.5 mL/min and 45°C. Thin layer chromatography (TLC) analyses were performed on silica gel Polygram® SIL G/UV_{254} pre-coated TLC-sheets.

Semi preparative (Semi-PREP) purifications were performed using SQD Waters single quadrupole mass spectrometer. This spectrometer is equipped with an ESI source, Waters 2535 quaternary pump coupled with 2767 sample Manager and with diode array detector (210 to 400 nm.) The column used is a Waters Sunfire ODB MS C18 column (5µm, 30 x 50 mm) for acidic purification and a Waters XBridge OBD MS C18 column (5µm, 30 x 50 mm) for basic purification.

Super Fluid Critical (SFC) purifications were performed using PREP600 system from Pic Solution equipped with a diode array detector (220 nm). Columns used are GS-NO2 (10µm, 50 x 229 mm) and LuxCell4 (20 µm, 50 x 291).

NMR spectra were recorded on a BRUKER AVANCE III Ultrashield Nanobay 400 MHz NMR Spectrometer and on a BRUKER AVANCE III HD Ascend 500 MHz NMR Spectrometer fitted with a 5 mm Prodigy BBO 500 S1 cryoprobe. The compounds were analyzed in d_6-DMSO solution at a probe temperature of 300 K. Chemical shifts are given in ppm downfield from TMS (tetramethylsilane) as internal standard. For ^{19}F NMR, chemical shifts are given in ppm downfield from TFA (trifluoroacetic acid, δ -76.50) as internal standard. The NMR multiplicity signals are reported as s = singlet, d = doublet, t = triplet, m = multiplet, br = broad, or combinations of thereof. Coupling constants J are quoted in Hz and reported to the nearest 1 Hz.

HRMS were obtained using a SYNAPT G2-SI Waters Q-TOF mass spectrometer. This spectrometer is equipped with an ESI source and a Waters Acquity H-class UPLC with diode array detector (210 to 400 nm.) An Acquity UPLC HSS T3 C18 column (1.8µm, 2.1 x 50 mm) was used.

b. Synthesis of 3

2-((bromofluoromethyl)thio)benzo[*d*]thiazole (1)

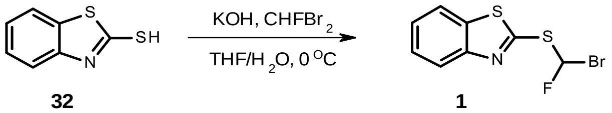

A solution of KOH (1.68 g, 30.0 mmol, 10 equiv.) and H_2O (5 mL) was prepared and placed in an ice bath. Then, a solution of 2-mercaptobenzothiazole (**32**, 0.50 g 3.0 mmol, 1.0 equiv.) in THF (5 mL) was added and, after 10 min, the cold bath was removed. The reaction mixture was stirred at room temperature for 20 min. Dibromofluoromethane (0.380 mL, 4.8 mmol, 1.6 equiv.) was then slowly added to the reaction mixture at 0°C. The reaction was stirred at 0ºC. After 2h, the crude mixture was quenched by addition of H_2O (30 mL), and the aqueous phase was extracted with DCM (3 x 40 mL). The combined organic layers were dried over anhydrous sodium sulfate (Na_2SO_4), filtered and concentrated under reduced pressure. Purification was performed on silica gel chromatography using hexane/ethyl acetate (gradient: starting from 100% hexane until 10% of ethyl acetate in hexane) to afford compound **1** (0.13 g, 0.47 mmol, yield = 16%) as a yellow oil.

^{1}H NMR (400 MHz, d_6-DMSO): δ 8.47 (d, J_{HF} = 56 Hz, 1H), 8.15 (d, J_{HH} =7.6 Hz , 1H), 8.06 (d, J_{HH} = 7.6 Hz, 1H), 7.58 (t, J_{HH} = 7.6 Hz, 1H), 7.51 (t, J_{HH} = 7.6 Hz, 1H);
^{13}C NMR (126 MHz, d_6-DMSO): δ 199.47, 152.08, 135.76, 126.76, 125.74, 122.43, 122.08, 90.55 (d, J_{CF} = 295 Hz);
^{19}F NMR (400 MHz, d_6-DMSO + TFA): δ -105.51 (d, J_{HF} = 54 Hz, 1F);
HRMS (m/z): $[M+H]^+$ calcd. for C_8H_6NFSBr, 277.9106 ; found, 277.0109.

2-(difluoromethylsulfanyl)-1,3-benzothiazole (2)

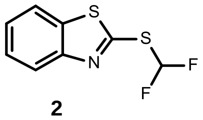

2-(difluoromethylsulfanyl)-1,3-benzothiazole **2** was commercially purchased.
^{1}H NMR (400 MHz, d_6-DMSO): δ 8.16 (d, J_{HH} = 8 Hz, 1H), 8.06 (d, J_{HH} = 8 Hz, 1H), 7.99 (t, J_{HF} = 54 Hz, 1H), 7.58 (t, J_{HH} = 7.0 Hz, 1H), 7.52 (t, J_{HH} = 7.0 Hz, 1H);
^{13}C NMR (126 MHz, d_6-DMSO): δ 155.94, 152.28, 135.95, 126.79, 125.83, 122.46, 122.07, 120.28 (t, J_{CF} = 275 Hz);
^{19}F NMR (400 MHz, d_6-DMSO + TFA): δ - 94.40 (d, J_{FH} = 54 Hz, 2F);
HRMS (m/z): $[M+H]^+$ calcd. for $C_8H_6NF_2S_2$, 217.9910 ; found, 217.9910.

2-((Difluoromethyl)sulfonyl)benzo[*d*]thiazole (3)

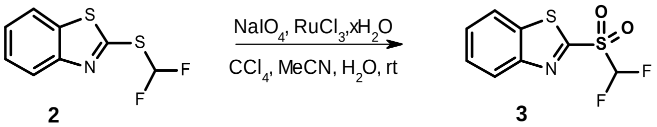

To a round-bottom flask containing 2-((difluoromethyl)thio)benzo[*d*]thiazole (**2**, 0.2 g, 1.0 mmol), were added MeCN (0.5 mL), CCl_4 (0.5 mL), H_2O (1 mL), $NaIO_4$ (1.07 g, 5.0 mmol) and $RuCl_3{\cdot}xH_2O$ (3.1 mg, 15 μmol). The resulting reaction mixture was stirred at room temperature for 2h. After completion of the reaction, H_2O (10 mL) was added, and the aqueous layer was extracted with diethyl ether (3 x 15 mL).

The combined organic layers were washed with saturated aqueous solution of $NaHCO_3$, and subsequently dried over anhydrous Na_2SO_4, filtered and concentrated to dryness. The crude was purified by column chromatography (silica gel, *n*-hexane/ethyl acetate = 90:10) to afford compound **3** (0.17 g, 0.67 mmol, 67 % yield) as a colorless solid.

^1H NMR (400 MHz, d_6-DMSO): δ 8.44 (m, 2H), 7.82 (m, 2H), 7.73 (t, J_{HF} = 52 Hz, 1H)
^{13}C NMR (126 MHz, d_6-DMSO): δ 159.01, 152.41, 137.49, 129.16, 128.55, 125.53, 123.74, 114.97 (t, J_{CF} = 282 Hz);
^{19}F NMR (400 MHz, d_6-DMSO + TFA): δ -123.97 (d, J_{FH} = 53 Hz, 2F);
HRMS (m/z): $[M+H]^+$ calcd. for $C_8H_6NF_2S_2O_3$, 249.9808 ; found, 249.9804.

c. Difluoromethylation

i. General procedures

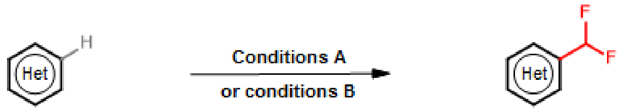

General procedure A : *Flow pseudo PET conditions*

A solution of 2-((Difluoromethyl)sulfonyl)benzo[*d*]thiazole **3** (30 mg, 0.120 mmol), the substrate (1.20 mmol, 10 equiv.) and $[Ir(ppy)_3]$ (4 mg, 5 mol%) in DMSO (2.4 mL) was prepared. Using the vapourtec flow system (Figure S1), the solution was injected in a 2 mL loop and pumped with cyclohexane as a solvent at a flow rate of 0.25 mL/min. The mixture passed through a flow photochemical reactor (10 mL, 24 W, 450 nm) at 55°C. After completion of the residence time, the crude material was collected, and the segmented flow of DMSO was filtered and directly injected in a Semi-Prep HPLC/MS system for purification. The desired product was finally analyzed by NMR and HRMS.

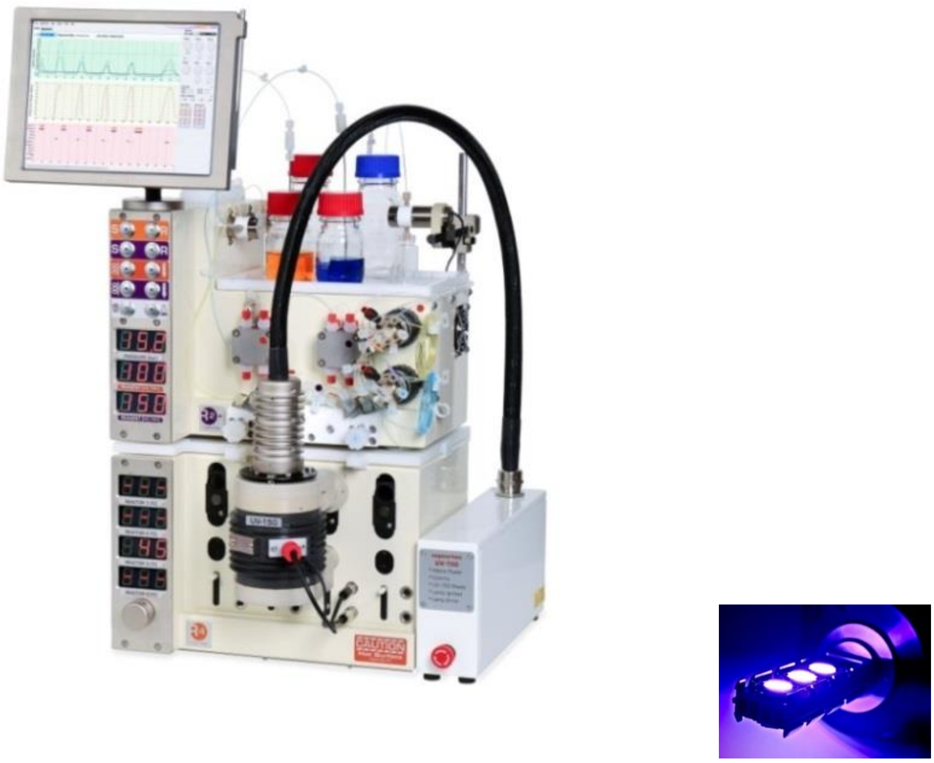

Figure S1 : Photochemistry flow instrument used from Vapourtec

General procedure B : *Baran Conditions*[1]

A solution of the substrate (0.40 mmol), $Zn(SO_2CHF_2)_2$ (250 mg, 0.80 mmol, 2 equiv.) and TFA (31 µL, 0.40 mmol, 1 equiv.) in DMSO (1.5 mL) was prepared. Then, tBuOOH (360 µL, 5.5 M in nonane, 2 mmol, 5 equiv.) was added dropwise, under vigorous stirring at rt for 5h to 24h. The crude material was purified by Semi-Prep HPLC/MS and the recovered product analyzed by NMR and HRMS.

ii. Gradients used for Semi prep purification

Purification Eluents:

Solvent A: H_2O (100%)
Solvent B: MeCN (100%)
Solvent C: H_2O/MeCN (50/50), TFA + 2% (pH ~2).
Solvent D: H_2O + NH_4HCO_3 100 mM + 500µL/L NH_4OH, (pH ~8.5).
HPLC flow rate : 35 mL/min to 45 mL/min , injection volume : 990 µL.

Gradient 1: Acidic, classic

Time (min)	A (%)	B(%)	C (%)	Flow (mL/min)
0	90	0	10	35
0.5	90	0	10	35
9	0	90	10	35
9.1	0	90	10	45
12	0	90	10	45

Gradient 2: Acidic, isocratic 95/5 (water/acetonitrile)

Time (min)	A (%)	B(%)	C (%)	Flow (mL/min)
0	90	0	10	35
0.5	90	0	10	35
0.6	90	0	10	35
9	90	0	10	35
9.1	0	90	10	45
12	0	90	10	45

Gradient 3: Acidic, gradient 95/5 to 60/40 (water/acetonitrile)

Time (min)	A (%)	B(%)	C (%)	Flow (mL/min)
0	90	0	10	35
0.5	90	0	10	35
0.6	90	0	10	35
9	55	35	10	35
9.1	0	90	10	45
12	0	90	10	45

Gradient 4: Acidic, gradient 80/20 to 70/30 (water/acetonitrile)

Time (min)	A (%)	B(%)	C (%)	Flow (mL/min)
0	90	0	10	35
0.5	90	0	10	35
0.6	75	15	10	35
9	65	25	10	35
9.1	0	90	10	45

12	0	90	10	45

Gradient 5: Acidic, gradient 85/15 to 75/25 (water/acetonitrile)

Time (min)	A (%)	B(%)	C (%)	Flow (mL/min)
0	90	0	10	35
0.5	90	0	10	35
0.6	80	10	10	35
9	70	20	10	35
9.1	0	90	10	45
12	0	90	10	45

Gradient 6: Acidic, gradient 55/45 to 35/65 (water/acetonitrile)

Time (min)	A (%)	B(%)	C (%)	Flow (mL/min)
0	90	0	10	35
0.5	90	0	10	35
0.6	50	40	10	35
9	30	60	10	35
9.1	0	90	10	45
12	0	90	10	45

Gradient 7: Acidic, gradient 99/1 to 90/10 (water/acetonitrile)

Time (min)	A (%)	B(%)	C (%)	Flow (mL/min)
0	98	0	2	35
0.5	98	0	2	35
0.6	98	0	2	35
9	85	5	10	35
9.1	0	90	10	45
12	0	90	10	45

Gradient 8: Basic, classic

Time (min)	A (%)	B(%)	D(%)	Flow (mL/min)
0	90	0	10	35
0.5	90	0	10	35
9	0	90	10	35
9.1	0	90	10	45

12	0	90	10	45

Gradient 9: Basic, gradient 60/40 to 40/60 (water/acetonitrile)

Time (min)	A (%)	B(%)	C (%)	Flow (mL/min)
0	90	0	10	35
0.5	90	0	10	35
0.6	55	35	10	35
9	45	45	10	35
9.1	0	90	10	45
12	0	90	10	45

Gradient 11: Basic, gradient 95/5 to 55/45 (water/acetonitrile)

Time (min)	A (%)	B(%)	C (%)	Flow (mL/min)
0	90	0	10	35
0.5	90	0	10	35
0.6	90	0	10	35
9	50	40	10	35
9.1	0	90	10	45
12	0	90	10	45

HRMS Eluent:

Solvent C: H_2O/MeCN (95/5) + Formic acid (750µL/L)
Solvent D: H_2O/MeCN (5/95) + Formic acid (500µL/L)
pH ~ 3

Time (min)	C (%)	D (%)	Flow (mL/min)
**0**	98	2	0.8
**0.3**	98	2	0.8
**3**	5	95	0.8
**4**	5	95	0.8
**4.1**	98	2	0.8
**5.1**	98	2	0.8

iii. Scope

2-amino-8-(difluoromethyl)-7-(2-hydroxyethoxymethyl)-9H-purin-6-ol / CHF$_2$-Acyclovir (5)

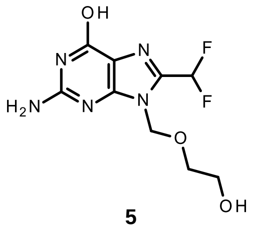

5

The general procedure A using acyclovir (0.120 mmol) yielded after purification (acidic mode, gradient 2) to 5.0 mg (15%) of the title compound as a colorless solid.

^{1}<u>*H NMR*</u> (400 MHz, d$_6$-DMSO): δ 10,82 (s, 1H), 7.14 (t, J_{HF} = 52 Hz, 1H), 6,72 (s broad, NH$_2$, 2H), 5,46 (s, 2H), 3,40 (m, 4H);
^{13}<u>*C NMR*</u> (126 MHz, d$_6$-DMSO): δ 156.0, 154.1, 152.2, 138.44 (t, J_{CF} = 27 Hz), 115.53, 109.45 (t, J_{CF} = 236 Hz), 71.67, 70.62, 59.79;
^{19}<u>*F NMR*</u> (500 MHz, d$_6$-DMSO + TFA): δ - 118.14 (d, J_{FH} = 52 Hz, 2F);
HRMS (m/z): [M+H]$^+$ calcd. for $C_9H_{12}N_5O_3F_2$, 276.0920; found, 276.0914.

4-(difluoromethyl)-1H-indole (6)

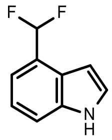

6

The general procedure B using indole (1 mmol) yielded after purification (basic mode, gradient 9) to 1.5 mg (1%) of the title compound as a purple oil.

^{1}<u>*H NMR*</u> (400 MHz, d$_6$-DMSO): δ 11.4 (s, 1H, NH), 7.58 (d, J_{HH} = 7.4 Hz, 1H), 7.48 (t, J_{HH} = 2.7 Hz, 1H), 7.24 (t, J_{HF} = 56 Hz, 1H), 7.22-7.05 (m, 2H), 6.60 (s broad, 1H);
^{13}<u>*C NMR*</u> (126 MHz, d$_6$-DMSO): δ 142.7, 134.7, 131.66, 123.76, 121.25 (t, J_{CF} = 6 Hz), 121.10, 120.91, 117.45, 113.54 (t, J_{CF} = 236 Hz);
^{19}<u>*F NMR*</u>, (400 MHz, d$_6$-DMSO + TFA): δ -111.48 (d, J_{FH}= 55 Hz, 2F);
<u>*HRMS (m/z):*</u> [M+H]$^+$ calcd. for $C_9H_8NF_2$, 168.0625; found, 168.0632.

2-(difluoromethyl)-1H-benzimidazole (7a), 4-(difluoromethyl)-1H-benzimidazole (7b) and 5-(difluoromethyl)-1H-benzimidazole (7c)

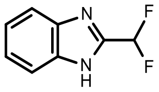

7a

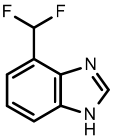

7b

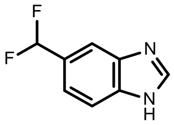

7c

The general procedure A using benzimidazole (0.15 mmol) yielded after purification (acidic mode, gradient 2) to 1.5 mg of **7a** (6%), 0.9 mg of **7b** (2.5 %) and 0.5 mg of **7c** (1.5%) as colorless solids.

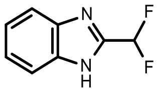

7a

^{1}*H NMR* (400 MHz, d_6-DMSO): δ 7.66 (dd, J_{HH} = 3.2 Hz, 2H), 7.31 (dd, J_{HH} =3.2 Hz, 2H), 7.27 (t, J_{HF} = 53 Hz, 1H);
^{13}*C NMR* (126 MHz, d_6-DMSO): δ 145.49 (t, J_{CF} = 28 Hz), 123.24, 116.1, 109.9 (t, J_{CF} = 236 Hz);
^{19}*F NMR* (500 MHz, d_6-DMSO + TFA): δ -117.08 (d, J_{FH} = 53 Hz , 2F);
HRMS (m/z): $[M+H]^+$ calcd. for $C_8H_7N_2F_2$, 169.0577; found, 169.0582.

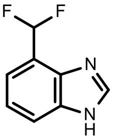

7b

^{1}*H NMR* (400 MHz, d_6-DMSO): δ 9.01 (s, 1H), 7.92 (d, J_{HH} = 8.1 Hz, 1H), 7.62 (d, J_{HH} = 8.1 Hz, 1H), 7.51 (d, J_{HH} = 7.8 Hz, 1H), 7.44 (t, J_{HF} = 55 Hz, 1H);
^{13}*C NMR* (126 MHz, d_6-DMSO): δ 136.30, 126.69, 124.67 (t, J_{CF} = 22 Hz), 124.07 (t, J_{CF} = 3 Hz), 116.98 (t, J_{CF} = 7.5 Hz), 116.02 (t, J_{CF} = 236 Hz), 114.27, 99.4;
^{19}*F NMR* (500 MHz, d_6-DMSO + TFA): δ -112.86 (d, J = 55 Hz, 2F);
HRMS (m/z): $[M+H]^+$ calcd. for $C_8H_7N_2F_2$, 169.0577; found, 169.0580.

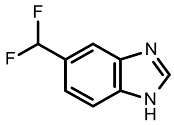

7c

^{1}*H NMR* (400 MHz, d_6-DMSO): δ 8.80 (s, 1H), 7.91 (s, 1 H), 7.81 (d, J_{HH} = 8.1 Hz, 1H), 7.53 (d, J_{HH} = 8.6 Hz, 1H), 7.16 (t, J_{HF} = 55 Hz, 1H);
^{13}*C NMR* (126 MHz, d_6-DMSO) δ 157.97, 157.71, 143.32, 139.72, 129.14 (t, J_{CF} = 3 Hz), 120.53, 115.46, 115.32 (t, J_{CF} = 236 Hz);
^{19}*F NMR* (500 MHz, d_6-DMSO + TFA): δ -108.94 (d, J_{FH} = 56 Hz, 2F);
HRMS (m/z): $[M+H]^+$ calcd. for $C_8H_7N_2F_2$, 169.0577; found, 169.0578.

2-(difluoromethyl)-4-methyl-1H-pyrrolo[2,3-b]pyridine (8a) and 6-(difluoromethyl)-4-methyl-1H-pyrrolo[2,3-b]pyridine (8b)

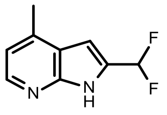

8a

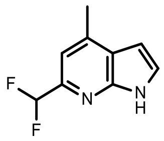

8b

The general procedure B using 4-methyl-1H-pyrrolo[2,3-b]pyridine (0.40 mmol) yielded after purification (acidic mode, gradient 1) to 2.7 mg (6%) of **8a** and 0.3mg of **8b** (0.5%) as yellowish solids.

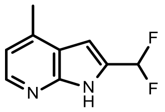

8a

<u>^{1}*H NMR*</u> (400 MHz, d_6-DMSO): δ 8.25 (d, J_{HH} = 5 Hz, 1H), 7.23 (t, J_{HF} = 54 Hz, 1H), 7.04 (d, J_{HH} = 5 Hz, 1H), 6.93 (t, J_{HH} = 2 Hz, 1H), 2.57 (s, 3H);
<u>^{13}*C NMR*</u> (126 MHz, d_6-DMSO): δ 146.80, 143.38, 142.58, 130.72 (t, J_{CF} = 25 Hz), 119.82, 116.92, 110.73 (t, J_{CF} = 234 Hz), 100.14 (t, J_{CF} = 7 Hz), 18.13;
<u>^{19}*F NMR*</u> (500 MHz, d_6-DMSO + TFA): δ -111.95 (d, J_{FH} = 55 Hz, 2F);
<u>*HRMS (m/z):*</u> $[M+H]^+$ calcd. for $C_9H_9N_2F_2$, 183.0734; found, 183.0735.

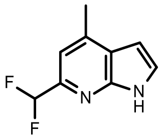

8b

<u>^{1}*H NMR*</u> (400 MHz, d_6-DMSO): δ 11.9 (s broad, 1H), 7.6 (t, J_{HH} = 3 Hz, 1H), 7.2 (s, 1H), 6.91 (t, J_{HF} = 55 Hz, 1H), 6.59 (m, 1H), 2.58 (s, 3H);
<u>^{13}*C NMR*</u> (126 MHz, d_6-DMSO): δ 147.18, 144.88 (t, J_{CF} = 24 Hz), 139.96, 127.45, 121.48, 114.97 (t, J_{CF} = 240 Hz), 112.15, 98.76, 18.16;
<u>^{19}*F NMR*</u> (500 MHz, d_6-DMSO + TFA): δ -113.25 (d, J_{FH} = 55 Hz, 2F);
<u>*HRMS (m/z):*</u> $[M+H]^+$ calcd. for $C_9H_9N_2F_2$, 183.0734; found, 183.0735.

4-(difluoromethyl)-6-methyl-1H-pyrazolo[3,4-b]pyridine (9a) and 3-(difluoromethyl)-6-methyl-1H-pyrazolo[3,4-b]pyridine (9b)

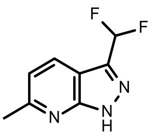

9a

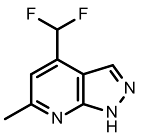

9b

The general procedure A using 6-methyl-1H-pyrazolo[3,4-b]pyridine (0.30 mmol) yielded after purification (acidic mode, gradient 3) to 2.1 mg (4%) of **9a** as a colorless solid and 1.3mg of **9b** (3%) as a yellowish solid.

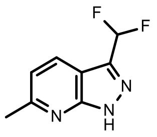

9a

^{1}H NMR (400 MHz, d_6-DMSO): δ 7.5 (s, 1H), 7.38 (t, J_{HF} = 55 Hz, 1H), 7.27 (s,1H), 2.65 (s, 3H);
^{13}C NMR (126 MHz, d_6-DMSO): δ 158.53, 152.33, 135.03 (t, J_{CF} = 24 Hz), 131.38,), 113.94 (t, J_{CF} = 7 Hz), 113.88 (t, J_{CF} = 237 Hz), 111.84, 24.41;
^{19}F NMR (500 MHz, d_6-DMSO + TFA): δ -112.62 (d, J_{FH} = 56 Hz, 2F);
HRMS (m/z): $[M+H]^+$ calcd. for $C_8H_8N_3F_2$, 184.0686; found, 184.0688.

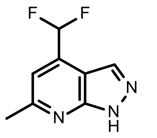

9b

^{1}H NMR (400 MHz, d_6-DMSO): δ 8.17 (d, J_{HH} = 8 Hz, 1H), 7.3 (t, J_{HF} = 54 Hz, 1H), 7.21 (d, J_{HH} = 8 Hz, 1H), 2.62 (s, 3H);
^{13}C NMR (126 MHz, d_6-DMSO): δ 159.26, 152.13, 137.65 (t, J_{CF} = 29 Hz), 129.08, 118.63, 114.29, 112.45 (t, J_{CF} = 230 Hz), 108.83, 24.47;
^{19}F NMR (400 MHz, d_6-DMSO + TFA): δ -115.48 (d, J_{FH} = 55 Hz, 2F);
HRMS (m/z): $[M+H]^+$ calcd. for $C_8H_8N_3F_2$, 184.0686; found, 184.0687.

2-[3,5-dichloro-2-(difluoromethyl)-4-pyridyl]-N,N-dimethyl-acetamide (10)

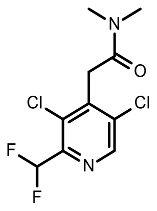

10

The general procedure B using 2-(3,5-dichloro-4-pyridyl)-N,N-dimethyl-acetamide (0.25 mmol) yielded after purification (acidic mode, gradient 1) to 7.4 mg (10%) of the title compound as a colorless solid.

^{1}H NMR (400 MHz, d_6-DMSO): δ 8.75 (s, 1H), 7.23 (t, J_{HF} = 56Hz, 1H), 4.11 (s, 2H), 3.15 (s, 3H), 2,86 (s, 3H);
^{13}C NMR (126 MHz, d_6-DMSO): δ 166.02, 146.49, 146.14 (t, J_{CF} = 23 Hz), 144.61, 135.01, 131.49, 112.00 (t, J_{CF} = 240 Hz), 36.89, 35.58, 35.01;
^{19}F NMR (500 MHz, d_6-DMSO + TFA): δ -119.44 (d, J_{FH} = 53 Hz, 2F);
HRMS (m/z): $[M+H]^+$ calcd. for $C_{10}H_{11}N_2OF_2Cl_2$, 283.0216; found, 283.0216.

3-chloro-1-(difluoromethyl)-6,7-dihydro-5H-cyclopenta[c]pyridine-4-carbonitrile (11)

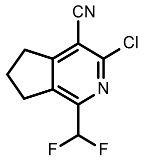

11

The general procedure B using 3-chloro-6,7-dihydro-5H-cyclopenta[c]pyridine-4-carbonitrile (0.40 mmol) yielded after purification (acidic mode, gradient 6) to 6.0 mg (7 %) of the title compound as a colorless solid.

<u>^{1}H NMR</u> (400 MHz, d_6-DMSO): δ 7.07 (t, J_{HF} = 52 Hz, 1H), 3.13 (m, 4H), 2.2 (q, J_{HH} = 8 Hz, 2H);
<u>^{13}C NMR</u> (126 MHz, d_6-DMSO): δ 165.94, 148.72 (t, J_{CF} = 26 Hz), 147.96, 138.75, 114.6, 112.71 (t, J_{CF} = 240 Hz), 108.30, 32.46, 29.17, 24.3;
<u>^{19}F NMR</u> (500 MHz, d_6-DMSO + TFA): -120.72 (d, J_{FH} = 53 Hz, 2F);
<u>HRMS (m/z)</u>: $[M+H]^+$ calcd. for $C_{10}H_8N_2F_2Cl$, 229.0344; found, 229.0342.

2-chloro-4-(difluoromethyl)-6,7-dihydro-5H-cyclopenta[b]pyridine-3-carbonitrile (12)

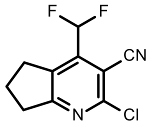

12

The general procedure B using 2-chloro-6,7-dihydro-5H-cyclopenta[b]pyridine-3-carbonitrile (0.40 mmol) yielded after purification (acidic mode, gradient 6) to 7.5 mg (8%) of the title compound as a brown solid.

<u>^{1}H NMR</u> (400 MHz, d_6-DMSO): δ 7.29 (t, J_{HF} = 52 Hz, 1H), 3.08 (m, 4H), 2.12 (q, J_{HH} = 8 Hz, 2H);
<u>^{13}C NMR</u> (126 MHz, d_6-DMSO): δ 173.46, 151.38, 141.10 (t, J_{CF} = 24 Hz), 135.33 (t, J_{CF} = 4 Hz), 113.75, 111.84 (t, J_{CF} = 240 Hz), 103.41 (t, J_{CF} = 4 Hz), 33.88, 28.54, 22.20;
<u>^{19}F NMR</u> (400 MHz, d_6-DMSO + TFA): δ -117.27 (dt, J_{FH} = 54 Hz, J_{FH}' = 2Hz, 2F);
<u>HRMS (m/z)</u>: $[M+H]^+$ calcd. for $C_{10}H_8N_2F_2Cl$, 229.0344; found, 229.0343.

4-(difluoromethyl)-2-tetrahydropyran-4-yl-pyrimidin-5-amine (13)

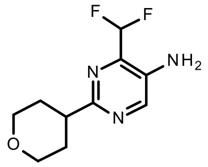

13

The general procedure B using 2-tetrahydropyran-4-ylpyrimidin-5-amine (0.40 mmol) yielded after purification (acidic mode, gradient 5) to 7.4 mg (7 %) of the title compound as a colorless solid.

<u>^{1}H NMR</u> (400 MHz, d_6-DMSO): δ 8.35 (s, 1H), 6.93 (t, J_{HF} = 52 Hz, 1H), 3.90 (d, J_{HH} = 12 Hz, 2H), 3.42 (t, J_{HH} = 12 Hz, 2H), 2.91 (m, 1H), 1.74 (m, 4H);

^{13}*C NMR* (126 MHz, d$_6$-DMSO): δ 159.27, 146.89, 138.21 (t, J_{CF} = 23 Hz), 137.84, 113.49 (t, J_{CF} = 236 Hz), 66.80, 42.02, 31.32;
^{19}*F NMR* (500 MHz, d$_6$-DMSO + TFA): δ -112.21 (d, J_{FH} = 54 Hz, 2F);
HRMS (m/z): [M+H]$^+$ calcd. for $C_{10}H_{14}N_3OF_2$, 230.1105; found, 231.1105.

4-chloro-2-(difluoromethyl)-6-methoxy-pyrimidin-5-amine (14)

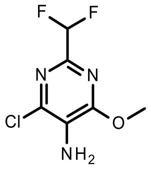

14

The general procedure B using 4-chloro-6-methoxy-pyrimidin-5-amine (0.40 mmol) yielded after purification (acidic mode, gradient 1) to 8 mg (10 %) of the title compound as a white solid.

^{1}*H NMR* (400 MHz, d$_6$-DMSO): δ 6.69 (t, J_{HF} = 54 Hz, 1H), 5.95 (s, 2H), 4.01 (s, 3H);
^{13}*C NMR* (126 MHz, d$_6$-DMSO) δ 157.39, 143.91 (t, J_{CF} = 26 Hz), 136.54, 128.96, 11.90 (t, J_{CF} = 245 Hz), 54.76;
^{19}*F NMR* (400 MHz, d$_6$-DMSO + TFA): δ -118.75 (d, J_{FH} = 52 Hz, 2F);
HRMS (m/z): [M+H]$^+$ calcd. for $C_6H_7N_3OF_2Cl$, 210.0246; found, 210.0249.

4-(difluoromethyl)-5-methyl-pyrimidin-2-amine (15)

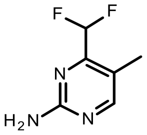

15

The general procedure A using 5-methylpyrimidin-2-amine (0.15 mmol) yielded after purification (basic mode, gradient 10) to 6.3 mg (26%) of the title compound as a colorless solid.

^{1}*H NMR* (400 MHz, d$_6$-DMSO): δ 8.25 (s, 1H), 6.77 (t, J_{HF} = 52 Hz, 1H), 6.75 (s broad, 2H), 2.15 (s, 3H); ^{13}*C NMR* (126 MHz, d$_6$-DMSO) δ 162.10, 161.49, 156.74 (t, J_{CF} = 23 Hz), 115.15, 113.74 (t, J_{CF} = 240 Hz), 12.61;
^{19}*F NMR* (400 MHz, d$_6$-DMSO + TFA): δ -130.32 (d, J_{FH} = 53 Hz, 2F);
HRMS (m/z): [M+H]$^+$ calcd. for $C_6H_8N_3F_2$, 160.0686; found, 160.0689.

4-(difluoromethyl)-2-methyl-6,8-dihydro-5H-pyrido[2,3-d]pyrimidin-7-one (16)

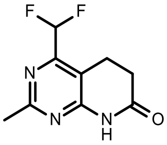

16

The general procedure B using 2-methyl-6,8-dihydro-5H-pyrido[2,3-d]pyrimidin-7-one (0.40 mmol) yielded after purification (acidic mode, gradient 5) to 10.8 mg (13 %) of the title compound as a colorless solid.

^{1}*H NMR* (400 MHz, d_6-DMSO): δ 11.07 (s, 1H), 6.99 (t, J_{HF} = 52 Hz, 1H), 3.01 (t, J_{HH} = 8 Hz, 2H), 2.59 (t, J_{HH} = 8 Hz, 2H);
^{13}*C NMR* (126 MHz, d_6-DMSO): δ 170.92, 165.43, 159.58, 154.19 (t, J_{CF} = 24 Hz), 113.22 (t, J_{CF} = 236 Hz), 111.25, 29.28, 24.94, 18.07;
^{19}*F NMR* (400 MHz, d_6-DMSO + TFA): δ -119.75 (d, J_{FH} = 54 Hz, 2F);
HRMS (m/z): [M+H]$^+$ calcd. for $C_9H_{10}N_3OF_2$, 214.0792; found, 214.0794.

5-(difluoromethyl)-1H-pyrimido[4,5-d]pyridazine-2,4-dione (17)

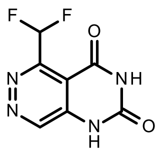

17

The general procedure B using 2-methyl-6,8-dihydro-5*H*-pyrido[2,3-*d*]pyrimidin-7-one (0.40 mmol) yielded after purification (acidic mode, gradient 2) to 14 mg (20%) of the title compound as a yellowish solid.

^{1}*H NMR* (400 MHz, d_6-DMSO): δ 12.1 (s, 1H), 11.2 (s broad, 1H), 9.42 (s, 1H), 7.54 (t, J_{HF} = 53 Hz, 1H);
^{13}*C NMR* (126 MHz, d_6-DMSO): δ 160.72, 150.22, 148.24, 143.17 (t, J_{CF} = 23 Hz), 137.20, 111.86, 110.43 (t, J_{CF} = 238 Hz);
^{19}*F NMR* (500 MHz, d_6-DMSO + TFA): δ -122.68 (d, J_{FH} = 53 Hz, 2F);
HRMS (m/z): [M+H]$^+$ calcd. for $C_7H_5N_4O_2F_2$, 215.0381; found, 215.0382.

Methyl 2-[3,5-dichloro-6-(difluoromethyl)-1-tetrahydropyran-2-yl-pyrazolo[3,4-b]pyridin-4-yl]acetate (18)

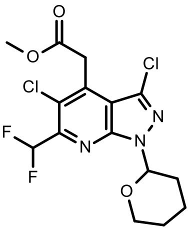

18

The general procedure A using methyl 2-(3,5-dichloro-1-tetrahydropyran-2-yl-pyrazolo[3,4-b]pyridin-4-yl)acetate (0.48 mmol, 8 equiv.) yielded after purification (basic mode, gradient 8) to 5.0 mg (3 %) of the title compound as a colorless solid.

^{1}*H NMR* (400 MHz, d_6-DMSO): δ 7.37 (t, J_{HF} = 53 Hz, 1H), 6.04 (dd, J_{HH} = 10 Hz, J'_{HH} = 2 Hz, 1H), 4.4 (s, 2H), 3.95 (d, J_{HH} = 10 Hz, 1H), 3.73 (m, 1H), 3.69 (s, 3H), 2.37 (m, 1H), 2.0 (m, 2H), 1.8 (m, 1H), 1.58 (m, 2H);
^{13}*C NMR* (126 MHz, d_6-DMSO): δ 168.47, 148.15 (t, J_{CF} = 23 Hz), 147.20, 138.46, 131.37, 124.02, 113.93, 111.92 (t, J_{CF} = 240 Hz), 81.90, 67.22, 52.61, 33.62, 28.58, 24.50, 21.99;

^{19}F NMR (400 MHz, d_6-DMSO + TFA): δ -120.84 (dd, J_{FH} = 53 Hz, J_{FH} = 29 Hz, 2F);
HRMS (m/z): $[M+H]^+$ calcd. for $C_{15}H_{16}N_3O_3F_2Cl_2$, 394.0537; found, 394.0534.

4-(difluoromethyl)-6-methyl-2-methylsulfanyl-8H-pyrimido[4,5-d]pyrimidine-5,7-dione (19)

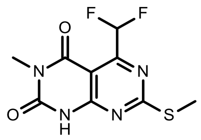

19

The general procedure B using 6-methyl-2-methylsulfanyl-8H-pyrimido[4,5-d]pyrimidine-5,7-dione (0.40 mmol) yielded after purification (acidic mode, gradient 1) to 8.5 mg (7.7%) of the title compound as a yellow solid.

^{1}H NMR (400 MHz, d_6-DMSO): δ 12.57 (s, 1H), 7.68 (t, J_{HF} = 53 Hz, 1H), 3.21 (s, 3H), 2.60 (s, 3H);
^{13}C NMR (126 MHz, d_6-DMSO) δ 175.99, 160.17, 159.23 (t, J_{CF} = 22 Hz), 157.02, 150.06, 108.93 (t, J_{CF} = 241 Hz), 101.61 (t, J_{CF} = 3 Hz), 27.18, 13.77;
^{19}F NMR (400 MHz, d_6-DMSO + TFA): δ -125.60 (d, J_{FH} = 53 Hz, 2F);
HRMS (m/z): $[M+H]^+$ calcd. for $C_9H_9N_40_2F_2S$, 275.0414; found, 275.0417.

8-(difluoromethyl)-1,3,7-trimethyl-purine-2,6-dione / CHF_2-caffeine (20)

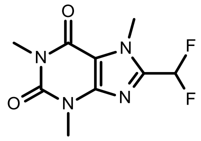

20

The general procedure A using caffeine (0.30 mmol, 5 equiv.) in DMF yielded after purification (acidic mode, gradient 2) to 2.5 mg (4%) of the title compound as a colorless solid.

^{1}H NMR (400 MHz, d_6-DMSO): δ 7.35 (t, J_{HF} = 52 Hz, 1H), 4.03 (s, 3H),3.42 (s, 3H), 3.24 (s, 3H);
^{13}C NMR (126 MHz, d_6-DMSO): δ 154.85, 150.85, 146.55, 142.6 (t, J_{CF} = 27 Hz), 108.52, 108.47 (t, J_{CF} = 236 Hz), 32.42, 29.48, 27.69;
^{19}F NMR (500 MHz, d_6-DMSO + TFA): δ -119.98 (d, J_{FH} = 52 Hz, 2F);
HRMS (m/z): $[M+H]^+$ calcd. for $C_9H_{11}N_4O_2F_2$, 245.0850; found, 245.0854.
Data in accordance with litterature[1,2]

8-(difluoromethyl)-1,3-dimethyl-7H-purine-2,6-dione / CHF_2-theophylline (21)

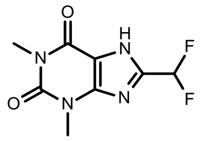

21

The general procedure A, using theophylline (0.30 mmol, 5 equiv.) in DMF yielded after purification (acidic mode, gradient 4) to 2.5 mg (5%) of the title compound as a colorless solid.

^{1}H NMR (400 MHz, d_6-DMSO): δ 7.13 (t, J_{HF} = 56 Hz, 1H), 3.43 (s, 3H), 3.24 (s, 3H);
^{13}C NMR (126 MHz, d_6-DMSO): δ 154.58, 151.07, 147.14, 143.22 (t, J_{CF} = 26 Hz), 108.66 (t, J_{CF} = 236 Hz), 108.17, 29.85, 27.87;
^{19}F NMR (500 MHz, d_6-DMSO + TFA): δ -118.57 (d, J_{FH} = 52 Hz, 2F);
HRMS (m/z): $[M+H]^+$ calcd. for $C_8H_9N_4O_2F_2$, 231.0694; found, 231.0697.

Data in accordance with litterature[1]

8-(difluoromethyl)-3,7-dimethyl-1-(5-oxohexyl)purine-2,6-dione / CHF_2-pentoxyfilline (22)

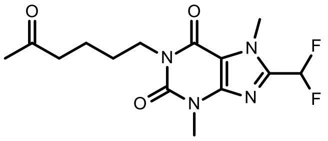

22

The general procedure B using pentoxyfilline (0.40 mmol) yielded after purification (acidic mode, gradient 1) to 9 mg (6.8%) of the title compound as a colorless solid.

^{1}H NMR (400 MHz, d_6-DMSO): δ 7.35 (t, J_{HF} = 52 Hz, 1H), 4.03 (s, 3H), 3.85 (t, J_{HH} = 7 Hz, 2H), 3.42 (s, 3H), 2.46 (t, J_{HH} = 7 Hz, 2H), 2.07 (s, 3H), 1.56-1.40 (m, 4H);
^{13}C NMR (126 MHz, d_6-DMSO): δ 208.20, 154.64, 150.55, 146.60, 142.70 (t, J_{CF} = 27 Hz), 108.48, 108.47 (t, J_{CF} = 235Hz), 42.19, 40.33, 32.41, 29.73, 29.43, 26.88, 20.49;
^{19}F NMR (400 MHz, d_6-DMSO + TFA): δ -119.37 (d, J_{FH} = 52 Hz, 2F);
HRMS (m/z): $[M+H]^+$ calcd. for $C_{14}H_{19}N_4O_3F_2$, 329.1425; found, 329.1423.

Data in accordance with litterature[1],[2]

5-(difluoromethyl)-1,3-dimethyl-pyrimidine-2,4-dione/ CHF_2-1,3-dimethyl-uracil (23)

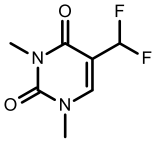

23

The general procedure A using dimethyl-uracil (0.40 mmol) yielded after purification (acidic mode, gradient 2) to 6.0 mg (26%) of the title compound as a colorless solid.

^{1}H NMR (400 MHz, d_6-DMSO): δ 8.17 (s, 1H), 6.75 (t, J_{HF} = 56 Hz, 1H), 3.36 (s, 3H), 3.17 (s, 3H);
^{13}C NMR (126 MHz, d_6-DMSO): δ 160.41, 150.91, 144.71, 112.02 (t, J_{CF} = 235 Hz), 105.01 (t, J_{CF} = 23 Hz), 36.81, 27.30;
^{19}F NMR (400 MHz, d_6-DMSO + TFA): δ -117.78 (d, J_{FH} = 55 Hz, 2F);
HRMS (m/z): $[M+H]^+$ calcd. for $C_7H_9N_2O_2F_2$, 191.0632; found, 191.0634.

Data in accordance with litterature[2]

6-amino-5-(difluoromethyl)-1H-pyrimidin-2-one and 6-amino-4-(difluoromethyl)-1H-pyrimidin-2-one / CHF_2-Cytosine (24)

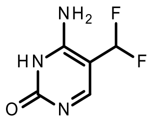

24a

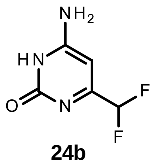

24b

The general procedure B using cytosine (0.40 mmol) yielded after purification (SFC mode, isocratic 75/25; CO_2/MeOH) to 0.5 mg (0.8%) of **24a** and 1.3 mg of **24b** (2.1%) as colorless solids.

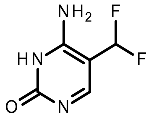

24a

<u>^{1}*H NMR*</u> (500 MHz, d_6-DMSO): δ 7.98 (s, 1H), 6.85 (t, J_{HF} = 54 Hz, 1H);
<u>^{13}*C NMR*</u> (126 MHz, d_6-DMSO) δ 160.3, 152.1, 146.5, 113.1 (t, J_{CF} = 235 Hz), 98.9;
<u>^{19}*F NMR*</u> (400 MHz, d_6-DMSO + TFA): δ -115;89 (d, J_{FH} = 53 Hz, 2F);
<u>*HRMS (m/z)*</u>: $[M+H]^+$ calcd. for $C_5H_6N_3OF_2$, 162.0479 ; found, 162.0481.

NB : As less than 1 mg of product was obtained, carbons assignments were determined by HSQC and HMBC analysis.
2 peaks at 9.48 and 8.48 ppm in the proton spectrum are observed, corresponding to the degradation of the difluoromethylated compound into the corresponding aldehyde.

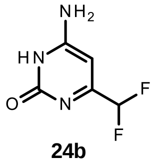

24b

<u>^{1}*H NMR*</u> (400 MHz, d_6-DMSO): δ 9.75 (s, 1H), 8.79 (s, 1H), 6.87 (t, J_{HF} = 54 Hz, 1H), 6.24 (s, 1H);
<u>^{13}*C NMR*</u> (126 MHz, d_6-DMSO) δ 160.52, 149.29 (t, J_{CF} = 25 Hz), 148.69, 109.23 (t, J_{CF} = 241 Hz), 91.61 (t, J_{CF} = 8 Hz);
<u>^{19}*F NMR*</u> (400 MHz, d_6-DMSO + TFA): δ -125.02 (d, J_{FH} = 53 Hz, 2F);
<u>*HRMS (m/z)*</u>: $[M+H]^+$ calcd. for $C_5H_6N_3OF_2$, 162.0479; found, 162.0485.

8-(difluoromethyl)-9H-purin-6-amine / CHF_2-Adenine (25)

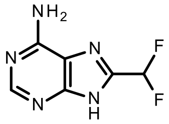

25

The general procedure B using adenine (0.40 mmol) yielded after purification (acidic mode, gradient 7) to 4.5 mg (6%) of the title compound as a colorless solid.

<u>^{1}*H NMR*</u> (400 MHz, d_6-DMSO): δ 8.50 (s, 1H), 7.31 (t, J_{HF} = 53 Hz, 1H);

^{13}C NMR (126 MHz, d$_6$-DMSO) δ 152.03, 150.05, 147.25, 145.46 (t, J_{CF} = 27 Hz), 117.52, 109.16 (t, J_{CF} = 234 Hz);
^{19}F NMR (400 MHz, d$_6$-DMSO + TFA): δ -118.33 (d, J_{FH} = 53 Hz, 2F);
HRMS (m/z): [M+H]$^+$ calcd. for $C_6H_5F_2N_5$, 186.0591; found, 186.0593.

(1S)-5-(difluoromethyl)-1-[(3R,4S,5R)-3,4-dihydroxy-5-(hydroxymethyl)tetrahydrofuran-2-yl]pyrimidine-2,4-dione / CHF$_2$-uridine (26)

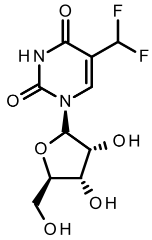

26

The general procedure B using uridine (0.40 mmol) yielded, after purification (acidic mode, gradient 7) to 2.5 mg (2.1%) of the title compound as a colorless oil.

^{1}H NMR (400 MHz, d$_6$-DMSO): δ 8.48 (s, 1H), 6.68 (t, J_{HF} = 54 Hz, 1H), 5.77 (d, J_{HH} = 4.5 Hz, 1H), 4.04 (t, J_{HH} = 5 Hz, 1H), 3.98 (t, J_{HH} = 5 Hz, 1H), 3.92-3.88 (m, 1H);
^{13}C NMR (126 MHz, d$_6$-DMSO) δ 160.98 (t, J_{CF} = 3 Hz), 150.25, 141.10 (t, J_{CF} = 7.5 Hz), 112.10 (t, J_{CF} = 234 Hz), 107.53 (t, J_{CF} = 23 Hz), 88.83, 84.97, 74.33, 69.58, 60.36;
^{19}F NMR (400 MHz, d$_6$-DMSO + TFA): δ -117.91 (dd, J_{FH} = 40 Hz, $J_{FH'}$ = 54 Hz, 2F);
HRMS (m/z): [M-H]$^-$ calcd. for $C_{10}H_{11}N_2O_6F_2$, 293.0585; found, 293.0590.

(1S)-4-amino-5-(difluoromethyl)-1-[(3R,4S,5R)-3,4-dihydroxy-5-(hydroxymethyl)tetrahydrofuran-2-yl]pyrimidin-2-one / CHF$_2$-cytidine (27)

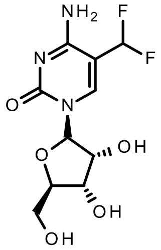

27

The general procedure B using cytidine (0.40 mmol) yielded after purification (SFC mode, isocratic 75/25 CO_2/EtOH) to 0.5 mg (1%) of the title compound as a colorless oil.

^{1}H NMR (500 MHz, d$_6$-DMSO): δ 8.5 (s, 1H), 6.81 (t, J_{HF} = 54 Hz), 5.74 (s, 1H), 3.99-3.93 (m, 2H), 3.91-3.86 (m, 1H), 3.72 (d, J_{HH} = 13 Hz, 1H), 3.57 (d, J_{HH} = 13Hz, 1H);
^{13}C NMR (126 MHz, d$_6$-DMSO): δ 160.3, 152.9, 142.9, 112.5 (t, J_{CF} = 222 Hz), 89.3, 83.7, 74.0, 68.3, 59.5;
^{19}F NMR (400 MHz, d$_6$-DMSO + TFA): δ -115.44 (dd, J_{HF} = 54 Hz, J_{HF} = 15 Hz, 2F);
HRMS (m/z): [M+H]$^+$ calcd. for $C_{10}H_{14}N_3O_5F_2$, 294.0802; found, 294.0897.

NB : The compound degrades easily, caution should be taken during the purification and the following evaporation.

As less than 1 mg of product was obtained, carbons assignments were determined by HSQC and HMBC analysis.

(3R,4S,5R)-2-[(9S)-6-amino-8-(difluoromethyl)purin-9-yl]-5-(hydroxymethyl) tetrahydrofuran-3,4-diol /CHF$_2$-Adenosine (28)

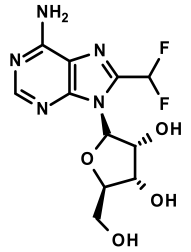

28

The general procedure B using adenosine (0.40 mmol) yielded after purification (acidic mode, gradient 7) to 4.2 mg (3.3%) of the title compound as a colorless solid.

^{1}*H NMR* (400 MHz, d$_6$-DMSO): δ 8.22 (s, 1H), 8.20 (s, 2H), 7.41 (t, J_{HF} = 52 Hz, 1H), 5.97 (d, J_{HH} = 7 Hz, 1H), 4.87 (dd, J_{HH} = 5 Hz, J_{HH}' = 7 Hz, 1H), 4.20 (dd, J_{HH} = 2 Hz, J_{HH}' = 5 Hz, 1H), 4.03 (m, 1H), 3.60 (m, 2H);
^{13}*C NMR* (126 MHz, d$_6$-DMSO) δ 164.16, 157.19, 154.25, 150.28, 118.57, 109.68 (t, J_{CF} = 237 Hz), 89.11, 97.13, 72.71, 71.05, 62.23;
^{19}*F NMR* (400 MHz, d$_6$-DMSO + TFA): δ -115.1 (dd, J_{FH} = 53Hz, J_{FF}' = 319 Hz, 1F), -118.6 (dd, J_{FH} = 53Hz, J_{FF}' = 319 Hz, 1F);
HRMS (m/z): $[M+H]^+$ calcd. for $C_{11}H_{14}N_5O_4F_2$, 318.1014; found, 318.1017.

(9S)-2-amino-8-(difluoromethyl)-9-[(3R,4S,5R)-3,4-dihydroxy-5-(hydroxymethyl) tetrahydrofuran-2-yl]-1H-purin-6-one / CHF$_2$-Guanosine (29)

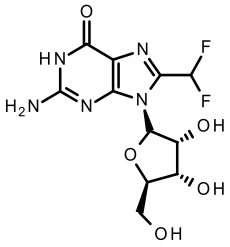

29

The general procedure B using guanosine (0.40 mmol) yielded after purification (acidic mode, gradient 7) to 5.2 mg (4%) of the title compound as a colorless solid.

^{1}*H NMR* (400 MHz, d$_6$-DMSO): δ 7.26 (t, J_{HF} = 52 Hz, 1H), 6.62 (s, 2H), 5.82 (d, J_{HH} = 6 Hz, 1H), 5.40 (d, J_{HH} = 6 Hz), 5.1-5.04 (m, 2H), 4.73-4.66 (m, 1H), 4.14 (s, 1H), 3.90 (q, J_{HH} = 4 Hz), 3.70-3.62 (m, 1H), 3.61-3.51 (m, 1H);
^{13}*C NMR* (126 MHz, d$_6$-DMSO) δ 157.02, 154.39, 152.29, 138.43 (t, J_{CF} = 26 Hz), 116.49, 109.51 (t, J_{CF} = 236 Hz), 88.04, 85.98, 72.06, 70.38, 61.71;
^{19}*F NMR* (400 MHz, d$_6$-DMSO + TFA): δ -110.55 (dd, J_{FH} = 54 Hz, J_{FF}' = 316 Hz, 1F), -115.32 (dd, J_{FH} = 54 Hz, J_{FF}' = 316 Hz, 1F);
HRMS (m/z): $[M+H]^+$ calcd. for $C_{11}H_{14}N_5O_5F_2$, 334.0963; found, 334.0964.

4-chloro-2-(difluoromethyl)-N-(4,5-dihydro-1H-imidazol-2-yl)-6-methoxy-pyrimidin-5-amine / CHF_2-moxonidine (30)

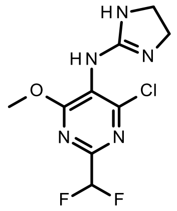

30

The general procedure B using 4-chloro-N-(4,5-dihydro-1H-imidazol-2-yl)-6-methoxy-pyrimidin-5-amine (0.40 mmol) yielded after purification (acidic mode, gradient 4) to 7.2 mg (7.9 %) of the title compound as a colorless solid.

<u>^{1}*H NMR*</u> (400 MHz, d_6-DMSO): δ 6.76 (t, J_{HF} = 54 Hz, 1H), 6.48 (s, 2H), 3.92 (s, 3H), 3.35 (s, 4H);
<u>^{13}*C NMR*</u> (126 MHz, d_6-DMSO): δ 163.80, 158.07, 149.58, 148.55 (t, J_{CF} = 25 Hz), 132.12, 111.87 (t, J_{CF} = 240 Hz), 54.52, 41.77;
<u>^{19}*F NMR*</u> (400 MHz, d_6-DMSO + TFA): δ -119.40 (d, J_{FH} = 53 Hz, 2F);
<u>*HRMS (m/z)*</u>: $[M+H]^+$ calcd. for $C_9H_{11}N_5OF_2Cl$, 278.0620; found, 278.0621.

(4R)-1-[[2-(difluoromethyl)-4-pyridyl]methyl]-4-(3,4,5-trifluorophenyl)pyrrolidin-2-one (31a), (4R)-1-[[3-(difluoromethyl)-4-pyridyl]methyl]-4-(3,4,5-trifluorophenyl)pyrrolidin-2-one (31b), (4R)-4-[2-(difluoromethyl)-3,4,5-trifluoro-phenyl]-1-(4-pyridylmethyl)pyrrolidin-2-one (31c)

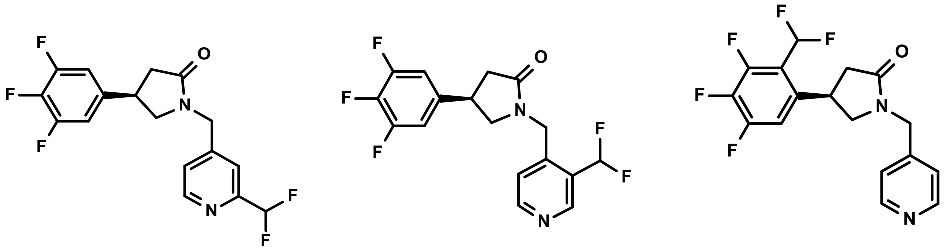

31a **31b** **31c**

The general procedure A using (4R)-1-(4-pyridylmethyl)-4-(3,4,5-trifluorophenyl)pyrrolidin-2-one **(39)** (0.30 mmol) yielded after purification (acidic mode, gradient 1, then basic mode, gradient 8) to 5 mg (16%) of **31a**, 400 µg (1.5%) of **31b** and 250 µg of **31c** (1%) as colorless solids.

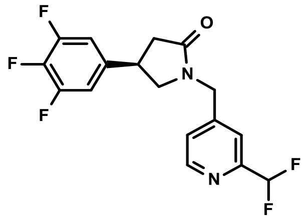

31a

1H NMR (400 MHz, d_6-DMSO): δ 8.65 (d, J_{HH} = 5 Hz, 1H), 7.55 (s, 1H), 7.46 (d, J_{HH} = 4.6 Hz, 1H), 7.33 (dd, J_{HH} = 6.8 Hz, J'_{HH} = 8.5 Hz, 2H), 6.95 (t, J_{HF} = 54 Hz, 1H), 4.61 (d, J_{HH} = 15 Hz, 1H), 4.49 (d, J_{HH} = 15 Hz, 1H), 3.69 (m, 2H), 3.29 (m, 1H), 2.76 (dd, J_{HH} = 7 Hz, J'_{HH} = 15 Hz, 1H), 2.60 (dd, J_{HH} = 7 Hz, J'_{HH} = 15Hz, 1H);
13C NMR (126 MHz, d_6-DMSO) δ 173.52, 152.68 (t, J_{CF} = 24 Hz), 150.61 (ddd, J_{CF} = 4 Hz, J'_{CF} = 10 Hz, J''_{CF} = 248 Hz), 150.37, 148.55, 140.01 (m), 137.98 (td, J_{CF} = 16 Hz, J'_{CF} = 248 Hz), 124.96, 119.51 (t, J_{CF} = 4 Hz), 114.19 (t, J_{CF} = 238 Hz), 112.41 (dd, J_{CF} = 4 Hz, J'_{CF} = 17 Hz), 53.50, 44.99, 37.73, 36.87;
19F NMR (400 MHz, d_6-DMSO + TFA): δ -116.92 (d, J_{FH} = 53 Hz, 2F), -136.72 (dd, J_{FH} = 8 Hz, J'_{FF} = 22 Hz, 2H), -165.69 (tt, J_{FH} = 8 Hz , J'_{FF} = 22 Hz, 1H);
HRMS (m/z): $[M+H]^+$ calcd. for $C_{17}H_{14}N_2OF_5$, 357.1026; found, 357.1030.

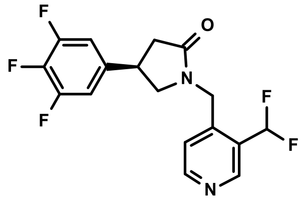

31b

1H NMR (400 MHz, d_6-DMSO): δ 8.77 (s, 1H), 7.38 (m, 2H), 7.31 (t, J_{HF} = 54 Hz, 1H), 4.72 (d, J_{HH} = 15Hz, 1H), 4.52 (d, J_{HH} = 15Hz, 1H), 3.68 (m, 2H), 2.75 (dd, J_{HH} = 8 Hz, J'_{HH} = 15 Hz, 1H), 2.31 (dd, J_{HH} = 8 Hz, J'_{HH} = 15 Hz, 1H);
13C NMR (126 MHz, d_6-DMSO) δ 173.7, 152.9, 147.6, 145.0, 136.9, 127.5, 123.0, 114.3, 112.6, 53.4, 42.0, 37.8, 36.9;
19F NMR (400 MHz, d_6-DMSO + TFA): δ -115.66 (d, J_{FH} = 55 Hz, 2F), -138.37 (dd, J_{FH} = 9 Hz, J'_{FF} = 21 Hz, 2H), -167.29 (tt, J_{FH} = 7 Hz , J'_{FF} = 22 Hz, 1H);
HRMS (m/z): $[M+H]^+$ calcd. for $C_{17}H_{14}N_2OF_5$, 357.1026; found, 357.1027.

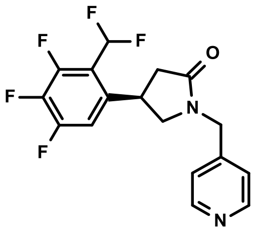

31c

^{1}H NMR (400 MHz, d$_6$-DMSO): δ 8.54 (d, J_{HH} = 5 Hz, 2H), 8.53 (s, 1H), 7.62 (m, 1H), 7.42 (t, J_{HF} = 52 Hz, 1H), 7.28 (d, J_{HH} = 5 Hz, 2H), 4.52 (d, J_{HH} = 14 Hz, 1H), 4.41 (d, J_{HH} = 14 Hz, 1H), 4.09 (t, J_{HH} = 8 Hz, 1H), 3.62 (t, J_{HH} = 8 Hz, 1H), 2.75 (dd, J_{HH} = 8.5 Hz, J'_{HH} = 16 Hz, 1H), 2.60 (d, J_{HH} = 9 Hz, 1H);
^{13}C NMR (126 MHz, d$_6$-DMSO) δ 172.2, 149.4, 145.9, 145.7, 122.2, 117.1, 111.8, 94.9, 53.1, 44.4, 31.9;
^{19}F NMR (400 MHz, d$_6$-DMSO + TFA): δ -113.73 (dd, J_{FH} = 52 Hz, J_{FF} = 11Hz, 2F), -132.53 (q, J_{FF} = 11 Hz, 1H), -141.10 (m, 1H), -164.52 (td, J_{FH} = 7 Hz, J_{FF} = 22 Hz, 1H);
HRMS (m/z): $[M+H]^+$ calcd. for $C_{17}H_{14}N_2OF_5$, 357.1026; found, 357.1026.

NB : As less than 1 mg of product was obtained for compounds **31b** and **31c**, carbons assignments were determined by HSQC and HMBC analysis.

d. Synthesis of the different precursors

*i. Synthesis of moxonidine analogue (**36**)*

36 was synthetized using a 2 steps approach procedure, previously reported by B. A. Czeskis for the synthesis of [^{14}C]moxonidine[3].

1-[2-[(4,6-dichloropyrimidin-5-yl)amino]-4,5-dihydroimidazol-1-yl]ethenone (35)

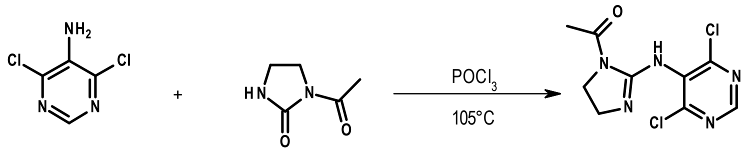

33 34 35

To a suspension of 4,6-dichloropyrimidin-5-amine **33** (1 g, 5.9 mmol) in $POCl_3$ (10mL) was added 1-acetylimidazolidin-2-one **34** (781 mg, 5.9 mmol, 1 equiv.). The reaction was stirred at 105˚C. After 3h, the crude mixture was cooled down to room temperature, and $POCl_3$ was evaporated under reduced pressure. The residue was treated with ice water, and aqueous NaOH was added until obtention of a pH>10. Then, the mixture was extracted twice with DCM. The combined organic layers, were then washed with brine, dried over anhydrous sodium sulfate (Na_2SO_4) and concentrated under reduced pressure. Purification on silica gel chromatography (isocratic: 50/50 heptane/ethyl acetate) provided compound **35** (0.60 g, 2.2 mmol, yield = 38%) as a colorless solid.
^{1}H NMR (400 MHz, d$_6$-DMSO): δ 8.50 (s, 1H), 7.54 (s, 1H), 3.90 (t, J_{HH} = 7 Hz, 2H), 3.38 (t, J_{HH} = 3 Hz, 3H);
^{13}C NMR (126 MHz, d$_6$-DMSO): δ 169.48, 153.37, 150.38, 150.14, 139.56, 43.77, 38.00, 24.71;
HRMS (m/z): $[M+H]^+$ calcd. for $C_9H_{10}N_5OCl_2$, 274.0262; found, 274.0269.

4-chloro-N-(4,5-dihydro-1H-imidazol-2yl)-6-methoxy-pyrimidin-5-amine (36)

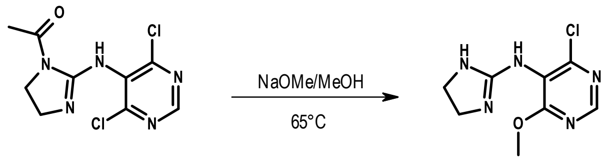

35 36

Sodium methoxide in MeOH (0.5 N, 3 mL, 1.55 mmol, 1.1 equiv.) was added to **35** (450 mg, 1.3 mmol) The reaction was refluxed at 65°C. After 3h, the crude mixture was cooled down, the formed precipitate was filtered and washed twice with H_2O to afford compound **36** (0.275 g, 1.2 mmol, yield = 92%) as a colorless solid.

^{1}H NMR (400 MHz, d_6-DMSO): δ 8.13 (s, 1H), 6.29 (s, 1H), 3.88 (s, 3H), 3.34 (s, 4H);
^{13}C NMR (126 MHz, d_6-DMSO): δ 163.50, 157.94, 150.22, 147.80, 130.49, 54.10, 41.79;
HRMS (m/z): $[M+H]^+$ calcd. for $C_8H_{11}N_5OCl$, 228.0652 ; found, 228.0657.

ii. *Synthesis of SV2A-PET **(39)***

(4R)-1-(4-pyridylmethyl)-4-(3,4,5-trifluorophenyl)pyrrolidin-2-one (39)
39 was synthetized in one step starting from **37** and **38** (the synthesis of an analogue of **38** is described in the patent WO2014/012563[4]).

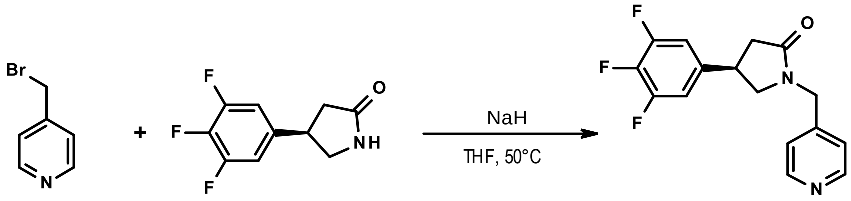

To a solution of (4R)-4-(3,4,5-trifluorophenyl)pyrrolidin-2-one **38** (1 g, 4.64 mmol) and 4-(bromomethyl)pyridine **37** (0.8 g, 4.64 mmol, 1 equiv.) in THF (5 mL) was added NaH (371 mg, 9.3 mmol, 2 equiv., 60% in oil) portionwise. The reaction was stirred at 50°C. After 1h, the crude mixture was filtered over celite and evaporated under reduced pressure. The residue was taken in DCM (20mL), H_2O was added (20 mL), and the aqueous phase was extracted with DCM (3 x 20 mL). The combined organic layers were dried over anhydrous sodium sulfate (Na_2SO_4), filtered and concentrated under reduced pressure. Purification on silica gel chromatography (gradient: starting from 100% DCM until 90/10 DCM/MeOH) to afford compound **39** (0.80 g, 2.6 mmol, yield = 56%) as a yellow solid.

^{1}H NMR (400 MHz, d_6-DMSO): δ 8.54 (d, J_{HH} = 5 Hz, 2H), 7.34 (dd, J_{HH} = 7 Hz, J'_{HH} = 9 Hz, 2H), 7.27 (d, J_{HH} = 5 Hz, 2H), 4.54 (d, J_{HH} = 16 Hz, 1H), 4.40 (d, J_{HH} = 16 Hz, 1H), 3.67 (m, 2H), 3.28 (t, J_{HH} = 7 Hz, 1H), 2.74 (dd, J_{HH} = 8 Hz, J'_{HH} = 8 Hz, 1H), 2.60 (dd, J_{HH} = 8 Hz, J'_{HH} = 16 Hz);
^{13}C NMR (126 MHz, d_6-DMSO) δ 173.32, -150.55 (ddd, J_{CF} = 3 Hz, J'_{CF} = 9 Hz, J''_{CF} = 248 Hz), 150.25, 146.33, 139.97 (m), 137.22 (ddd, J_{CF} = 16 Hz, J'_{CF} = 31 Hz, J''_{CF} = 248 Hz), 122.96, 112.44 (dd, J_{CF} = 5 Hz, J'_{CF} = 16 Hz), 53.38, 44.97, 37.86, 36.94;
^{19}F NMR (400 MHz, d_6-DMSO + TFA): δ -137.16 (dd, J_{FF} = 22 Hz, J_{FH} = 9 Hz, 2H), -166.07 (td, J_{FF} = 22 Hz, J_{FH} = 7 Hz);
HRMS (m/z): $[M+H]^+$ calcd. for $C_{16}H_{14}N_2OF_3$, 307.1058 ; found, 307.1060.

2. Radioactive Chemistry
a. Generality

No-carrier-added [^{18}F]fluoride was produced via the ^{18}O(p,n)^{18}F nuclear reaction by bombardment of ^{18}O-enriched water (>95%) with 18 MeV protons using a cyclone 18/18 (IBA) . ^{18}O-enriched water was purchased from Rotem or ABX. At the end of bombardment (EOB), the activity was transferred to the hot lab cell with helium pressure through Teflon tubing (~50 m).
Radioactivity was measured in a dose calibrator (Veenstrat). All the radiochemical yields are decay corrected.

First experiments were realized with a low level of radioactivity (37-185 MBq (1- 5 mCi)). For synthesis at higher level (111 GBq (3 Ci)), an automated FASTlab™ module from GE Healthcare was used.

Thin layer chromatography (TLC) were realized silica gel Polygram® SIL G/UV_{254} pre-coated TLC sheets eluted with MeOH (100%). The same eluent was used for all the radioactive analyses. The radioactive spots were quantitatively detected on a Berthold TLC scanner (model AR200). The TLC identity of all the labelled compounds was confirmed by UPLC after injection and co-injections on the same analytical system (see above) of the corresponding ^{19}F-fluorinated references.

Sep-Pak cartridges (Light ^{t}C18 (360 mg, 55-105 μm) and Accell™ Plus QMA Carbonate Plus Light cartridges (46 mg, 37-55 μm) were obtained from Waters (Milford, USA). The ^{t}C18 SPE were preconditioned beforehand with MeCN (3 mL) and H_2O (6 mL).

UPLC analyses were carried out on an ACQUITY UPLC® system (Waters) equipped with a PDA UV (200-400 nm) and a gamma–ray NaI detectors. The system was controlled by the Empower software. The ACQUITY UPLC® CSH™ C18 column (2.1 x 100 mm, 1.7 μm; Waters), heated at 45°C, was eluted in gradient mode with a mixture consisting of MeCN/H_2O (0,05 % HCOOH) (See Table S1).

Table S1 : UPLC gradient for ^{18}F-labeled compounds

Time (min)	H_2O (0.05% HCOOH) (%)	MeCN (%)	Flow (mL/min)
0	100	0	0.5
0.5	100	0	0.5
6	25	75	0.5
7	100	0	0.5
8	100	0	0.5

The semi-preparative HPLC purification was conducted on a X-Terra® RP18 HPLC column (10 x 250 mm, 10 µm, Waters), connected to a stand alone HPLC. The loop of a motorized rheodyne valve was lined to the outlet of the synthesizer module. The Waters system (600 pump, 996 PDA UV detector (190-400 nm) was controlled by the Empower software. The radioactive elution profile was monitored with a custom-made Geiger-Müller (GM) radioactivity detector. The column was eluted with an isocratic mixture of MeCN/H_2O (40/60 (*v*/*v*)) at a flow rate of 5 mL min^{-1}.

Flow reactions for difluoromethylation were performed using the Futurechem FlowStart EVO system, with a microchip of 100 µL and a 2 W LED of 470 nm.

b. Automated radiosynthesis of [^{18}F]3

The whole radiosynthesis of **[^{18}F]3** was performed on a FASTlab™ synthesizer from *GE Healthcare*. The reagents and solvents used for the radiosynthesis of **[^{18}F]3** were placed in small sealed vials according to a process previously reported in our laboratory[5]. Reagents were prepared and positioned on the FASTlab™ manifold as described and illustrated in Figure S2.

The enriched Oxygene-18 water containing [^{18}F]fluoride was directly recovered from the cyclotron target (V6) onto the FASTlab synthesizer and trapped on an ion exchange resin (QMA Carbonate Cartridge; from V5 to V4) and the [^{18}O]H_2O was recovered in a separate vial (V1) (Figure 2). The [^{18}F]fluoride was eluted into the reactor through a central tubing (V8) with 750 μL of a Kryptofix® ($K_{2.2.2.}$, 7.5 mg in 600 μL of MeCN) and K_2CO_3 (1.4 mg in 150 μL of H_2O) solution. The eluent was azeotropically evaporated under vacuum and nitrogen flow by heating at 105°C and 120°C. Afterwards, 1 mL of 2-((bromofluoromethyl)thio)benzo[*d*]thiazole (**1**) in MeCN (11,1 mg; 0,04 mmol; 1.1 mL) was then transferred to the dry potassium [^{18}F]fluoride/$K_{2.2.2.}$ complex through the central tubing of the reactor (V8) and heated to 120 °C for 5 min (labeling). After labeling, the reaction medium containing the 2-((di[^{18}F]fluoromethyl)thio)benzo[*d*]thiazole (**[^{18}F]2**) was diluted three times in syringe S2 (V11) with H_2O (~12 mL) (V15), and the labeled compound **[^{18}F]2** was trapped on a ^{t}C18 cartridge (from V17 to V18). The reactor was washed with H_2O (~4 mL), and this solution was passed through the cartridge. An solution containing $NaIO_4$ (51,3 mg; 0,24 mmol) and $RuCl_3 \cdot xH_2O$ (1,7 mg; 0,008 mmol) in H_2O (4 mL) (V14) was passed through the ^{t}C18 cartridge and the oxidation of **[^{18}F]2** was performed on it for 5 min at room temperature. Thereafter, the crude labeled compound **[^{18}F]3** was eluted from the ^{t}C18 cartridge (from V18 to V17; reverse flow elution) with MeCN (2 mL; syringe S3, V24) and recovered into the reactor via its central tubing. After dilution with H_2O (4 mL) the resulting solution was transferred with syringe S2 (V11) into the semi-preparative HPLC loop (V9; 6 mL) through a Sterifix® Paed filter (0.2 μm). The reactor was then washed with water (~2 mL), and the aqueous solution was transferred into the HPLC loop. The semi-preparative HPLC purification was performed using MeCN/H_2O (isocratic, 40/60 (*v*/*v*) at 5 mLmin^{-1}). The HPLC peak corresponding to **[^{18}F]3** was collected (retention time = 16.5-19.5 min) in a sealed vial containing water (~30 mL). Afterwards, **[^{18}F]3** was pumped (from V10), 6 mL by 6 mL, with the syringe S2 (V11) and further passed through a preconditioned ^{t}C18 cartridge (from V21 to V22). Finally, **[^{18}F]3** was eluted into the outlet vial (V20) with reverse flow of DMSO (1 mL, syringe S3 (V24)).

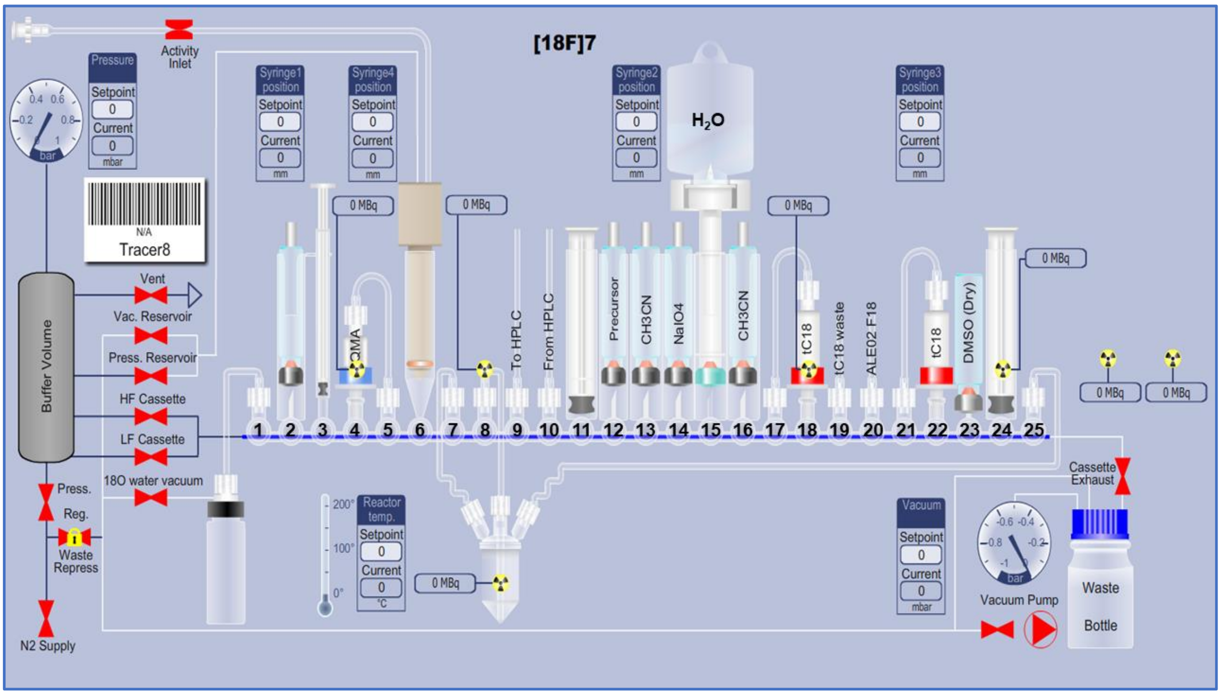

Figure S2 : Layout of the FASTLAB cassette for the radiosynthesis of [^{18}F]3.

c. Optimization of the labeling and oxidative steps

The conditions screened for the labeling and oxidative steps are summarized in the following table (Table S2) and each radiochemical experiment was conducted three times (n=3).
The Radio-LC and a LC of the references are also shown after the first table to confirm the formation of the desired product (Figures S3-6).

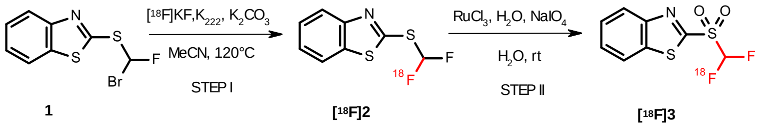

Table S2 : Optimization of the reaction conditions for the preparation of [^{18}F]3

Entry	Reaction	Deviation from usual conditions[1,2]	RCY(%)
**1**	I	none	15.2 ± 0.3
**2**	I	85°C	12.7 ± 0.2
**3**	I	85°C, DCE	7,2 ± 0,5
**4**	I	85°C, DMSO	0
**5**	I	Precursor 1 (80 µmol)	5.9 ± 2.4
**6**	I	$Et_4N^+HCO_3^-$	12.8 ± 1.1
**7**	II	none	13.4 ± 0.4
**8**	II	$NaIO_4$ (0.12 mmol)	9.6 ± 0.9

[1] Usual conditions for I : 1 (40 µmol), K_{222} (10 µmol), K_2CO_3 (20 µmol), MeCN, 120°C, 5min
[2] Usual conditions for II : $NaIO_4$ (240 µmol), $RuCl_3.H_20$ (80 µmol), H_20, rt, 5 min

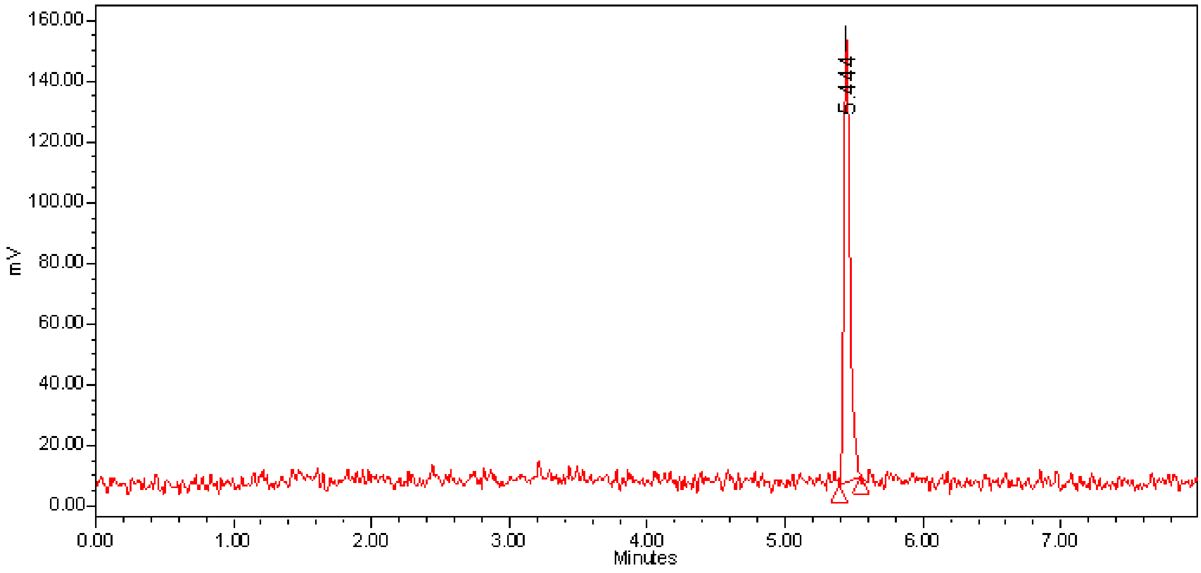

Figure S3 : UPLC radio-chromatogram of [^{18}F]2.

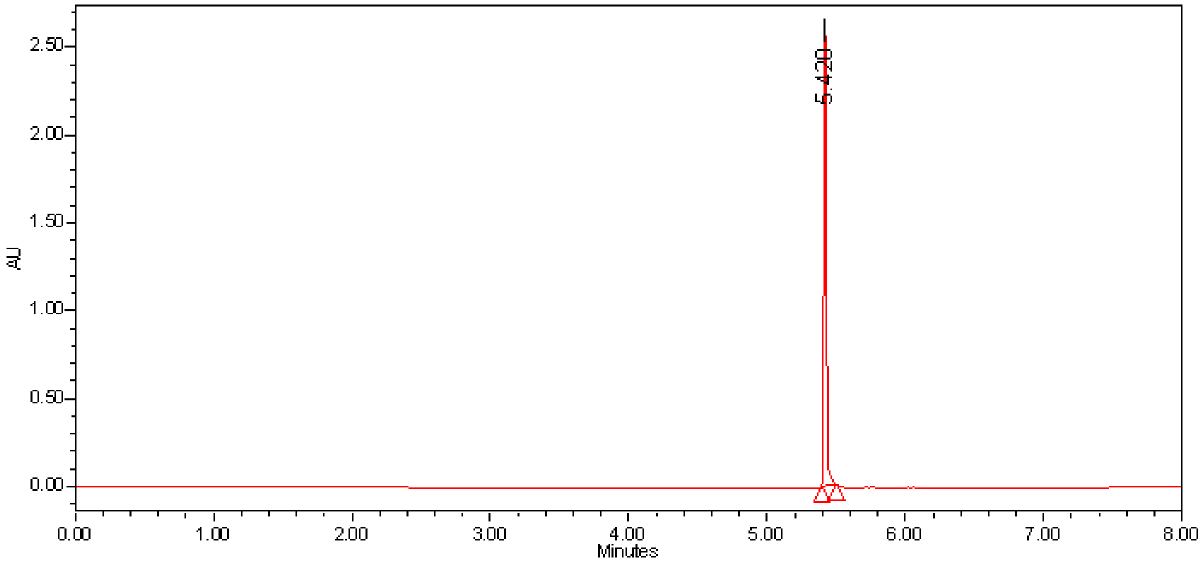

Figure S4 : UPLC UV-chromatogram (254 nm) of an authentic reference 2.

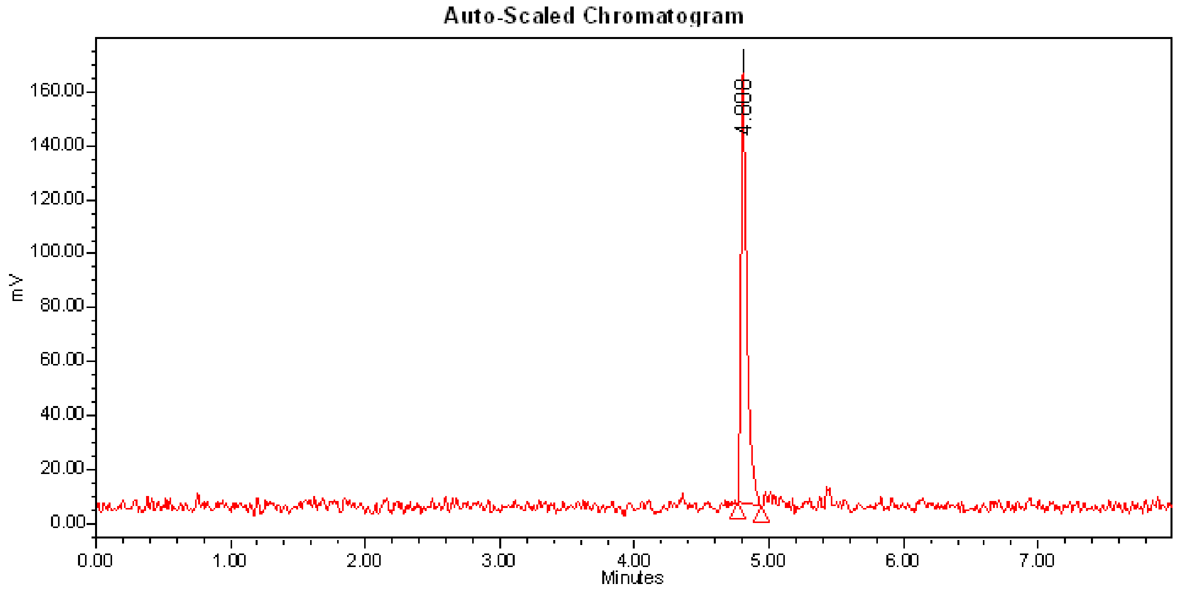

Figure S5 : UPLC radio-chromatogram of [^{18}F]3

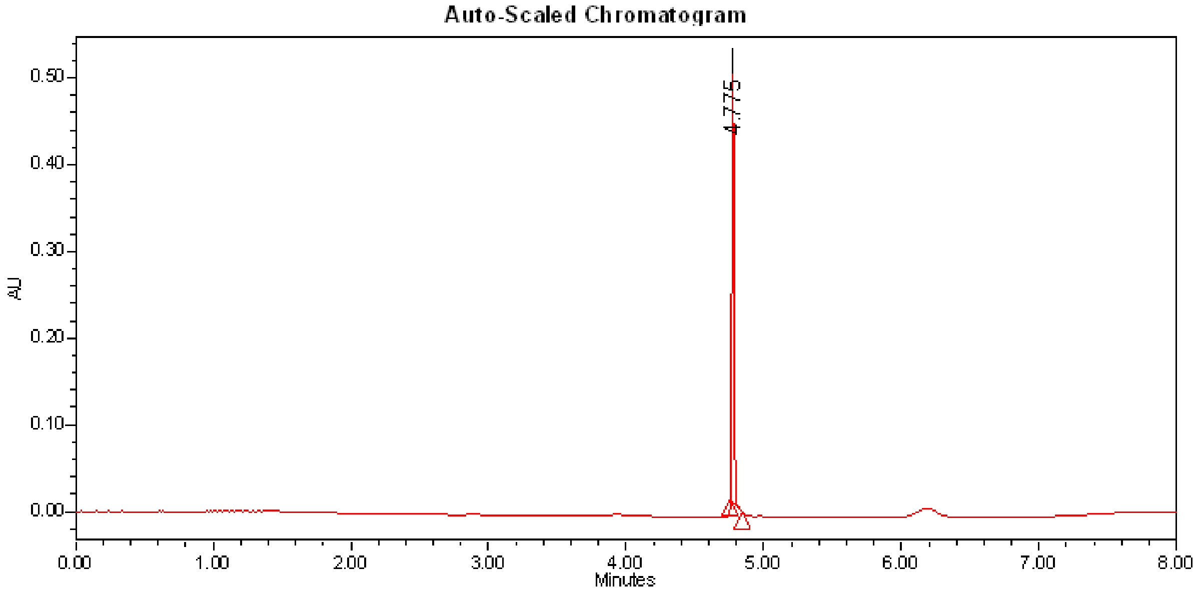

Figure S6 : UPLC UV-chromatogram (239 nm) of an authentic reference 3

For each experiment, the RCY (decay-corrected) of **[^{18}F]2** and **[^{18}F]3** was determined after SPE purification on an aliquot of the final solution according to the following formula taking into account the TLC and HPLC radiochemical purity of the isolated product.

$$RCY\ (\%, dc) = \frac{radioTLC\ purity\ (\%) \times radioUPLC\ purity\ (\%) \times activity\ of\ crude\ solution\ (dc)}{starting\ activity\ (dc)} \times 100\%$$

d. Isolation and determination of molar activity of the sulfone [^{18}F]3

The fully automated synthesis of the ^{18}F-labeled sulfone was realized with the FastLab module as described in section 2b using the best conditions reported in section 2c (Table 2, entry 7). The molar activity of **[^{18}F]3** was determined on a aliquot of the DMSO solution (5 µL). After UPLC elution, the radioactive peak associated to the non-radioactive sulfone was collected and counted and the UV area of the peak determined (□ = 239 nm). The decay-corrected activity was calculated and the corresponding amount of **3** was determined using the calibration curve previously obtained with the non-radioactive reference (Figure S7). A molar activity between 74 and 185 GBq/µmol (2 and 5 Ci/µmol) was calculated.

These analyses were performed with the UPLC system and the conditions reported above.

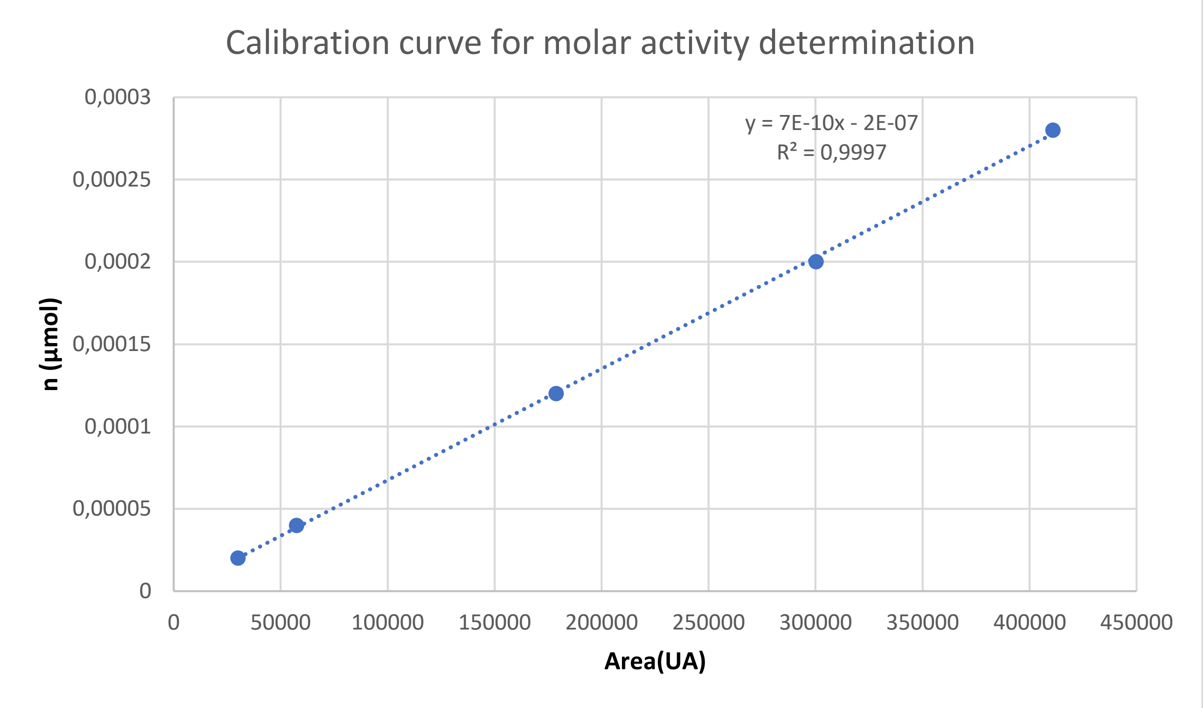

Figure S7 : Calibration curve of 3

For the following photoredox reaction, aliquots of the recovered DMSO solution of **[^{18}F]3** were used.

e. Optimization of the photochemical reaction

2-amino-8-(difluoromethyl)-7-(2-hydroxyethoxymethyl)-9H-purin-6-ol / CHF^{18}F-Acyclovir [^{18}F]5

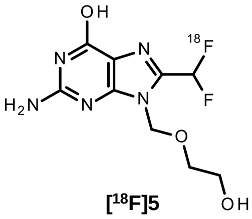

[^{18}F]5

A solution of acyclovir (4.5mg, 20 µmol), [Ir(ppy)$_3$] (0.01 µmol) in DMSO (200 µL) was prepared. Then **[^{18}F]3** in DMSO (around 37 MBq/ 1mCi) was added and the solution was injected through a 100 µL microchip, pumped with DMSO at a flow rate of 50 µL/min (residence time of 2 min) and irradiated under blue LED (470nm, 2W), at a temperature of 35°C (see the instrument in Figure S8). The exited solution was analysed by Radio-TLC and Radio-UPLC for radiochemical yield determination.

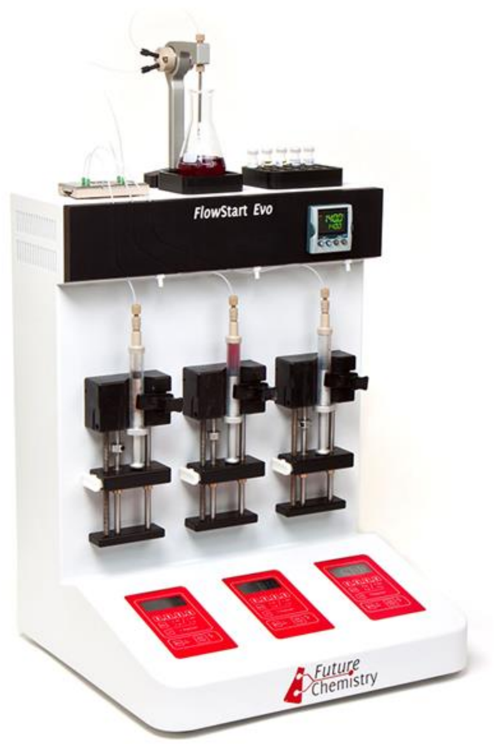

Figure S8 : Flow chemistry instrument used for ^{18}F-difluoromethylation; Futurechemistry

Radiochemical yields were determined according the following formula and are not decay corrected.

$$RCY\ (\%) = \frac{LC\ purity(\%) * TLC\ purity(\%)}{100}$$

For instance :

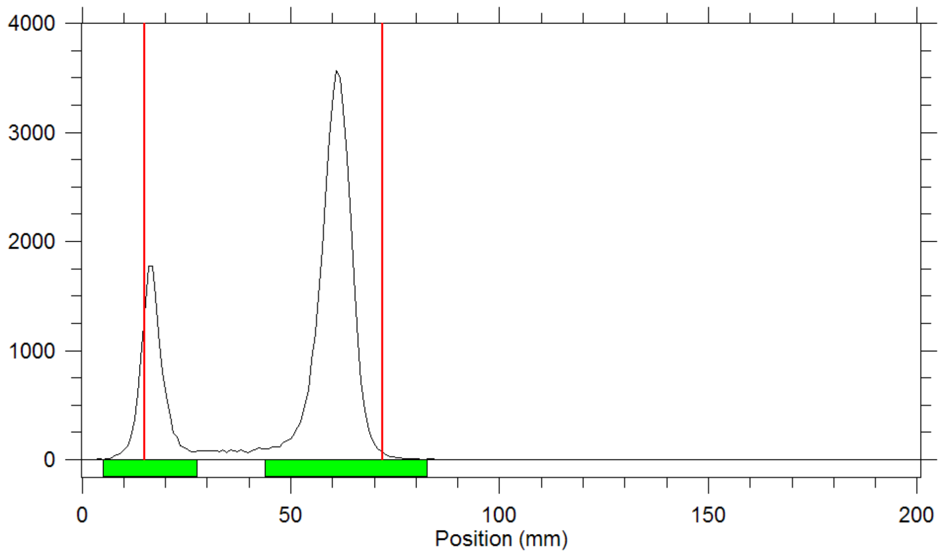

Figure S9: Radio TLC of [^{18}F]5

Table S3 : Results Radio-TLC [^{18}F]5

Rf	*Ratio*

0.03<br>0.79	26 (impurity/byproduct)<br>74 (product)

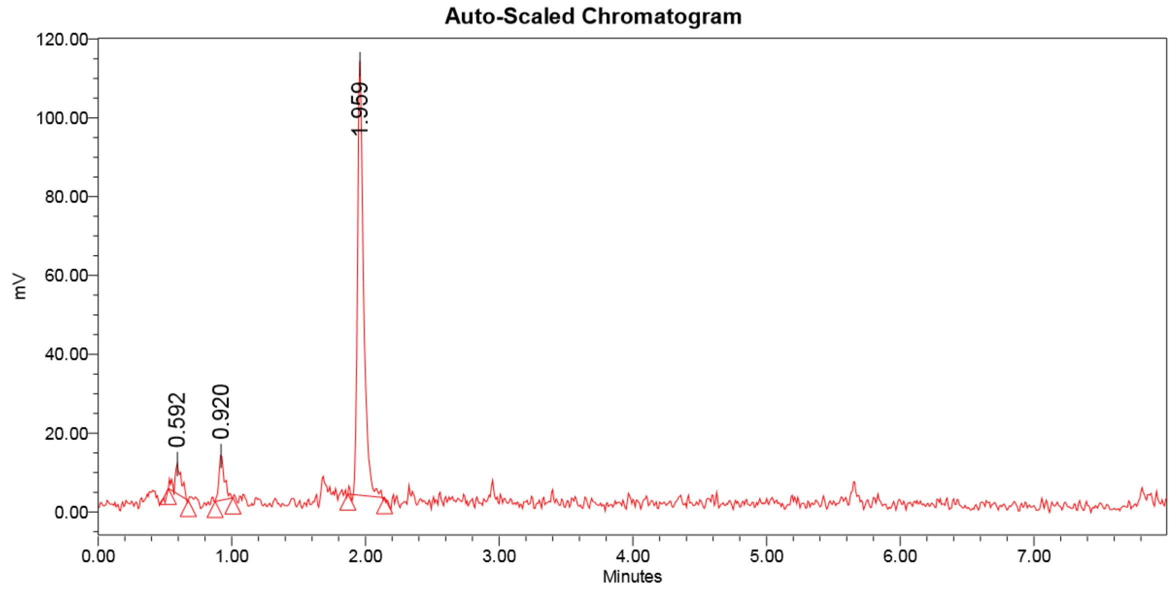

Figure S10 : UPLC radio-chromatogram of [^{18}F]5

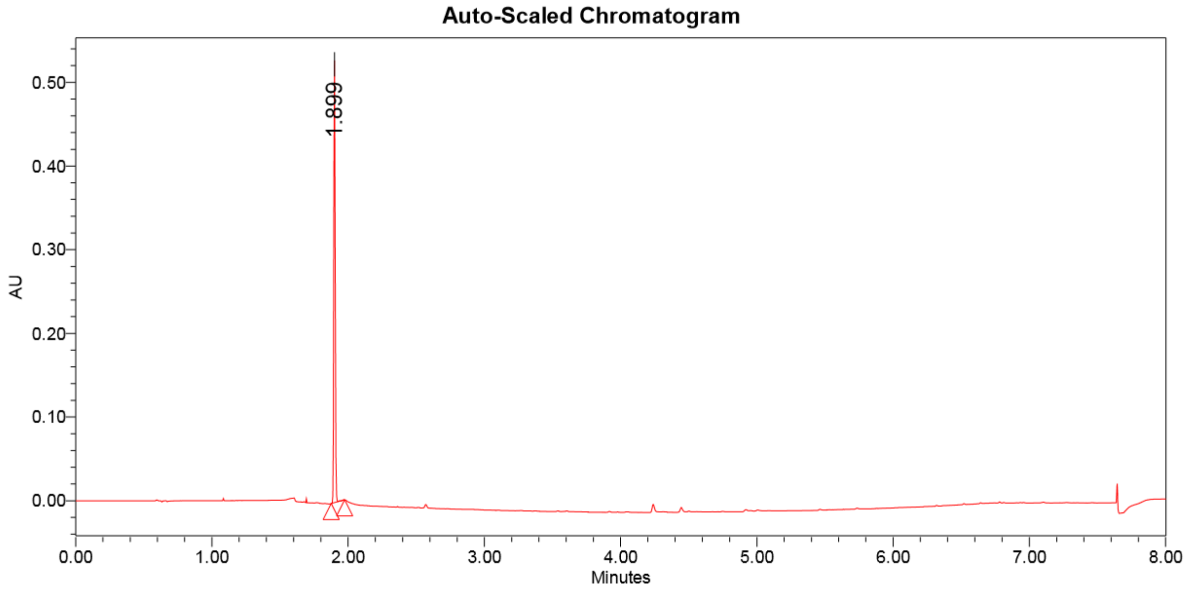

Figure S11 : UPLC UV-chromatogram of an authentic reference 5

$$RCY\ (\%) = \frac{LC\ purity(\%) * TLC\ purity(\%)}{100}$$

$$RCY\ (\%) = \frac{100 * 74}{100}$$

$$RCY\ (\%) = 74\%\ (ndc)$$

NB : In some cases, some peaks at 0.6 and 0.9 min can be observed on the radio-UPLC chromatograms. Those two peaks were collected and analysed by radio-TLC. Their Rf being

of 0, they were not taking into account for the determination of the LC purity (as they were already taken into account in the determination of the TLC purity).

The results of the different optimization tests are summarized in the following table :

Conditions : Acyclovir (20 µmol) , [Ir(ppy)$_3$] (0.01 µmol), **[^{18}F]3** (37 MBq), DMSO (200 µL), 2min, 35°C, blue LED (2 W)

Table S4 : Screening of experimental conditions for the ^{18}F-difluoromethylation reaction

Entry	Deviation from usual conditions	RCY(%, ndc)
**1**	none	70 ± 7
**2**	55°C	51 ± 10
**3**	DMF	44 ± 1
**4**	30s	60 ± 8
**5**	Ir(ppy)$_3$ (0.001 µmol)	42
**6**	Benzophenone[1]	47 ± 5
**7**	Ru(bpy)$_3$	0
**8**	H_2O (50 µL)	45 ± 10
**9**	After HPLC purification	42 ± 4[2]

[1] benzophenone (10 µmol), 365nm, [2] Isolated Radiochemical Yield (n=4)

Only a few solvents allows a complete solubilization of the substrate, DMSO gives better results than DMF.
The temperature is also an important parameter: too high temperature leads to more degradation.
The best conditions for the ^{18}F-difluoromethylation are 35°C, [Ir(ppy)$_3$] (0.01 µmol), 2 minutes of residence time and DMSO as solvent (see entry 1, Table S4). These conditions were applied to the scope.

f. Isolation and Molar activity of CHF^{18}FAcyclovir [^{18}F]5

After completion of the photoredox reaction that was realized as described in the previous section, H_2O (5 mL) was added. The purification of **[^{18}F]5** was then conducted at 5 mL/min on the semi preparative HPLC column described above. The eluent was a mixture of H_2O (95%) and MeCN (5%). Based on the amount of sulfone used for the photochemistry reaction, the RCY of **[^{18}F]5** purified by HPLC was of 42 ± 4 % (dc).

Then following the same process as for compound **3**, a calibration curve (Figure S12) was realized with **5** to determine the molar activity of the product.

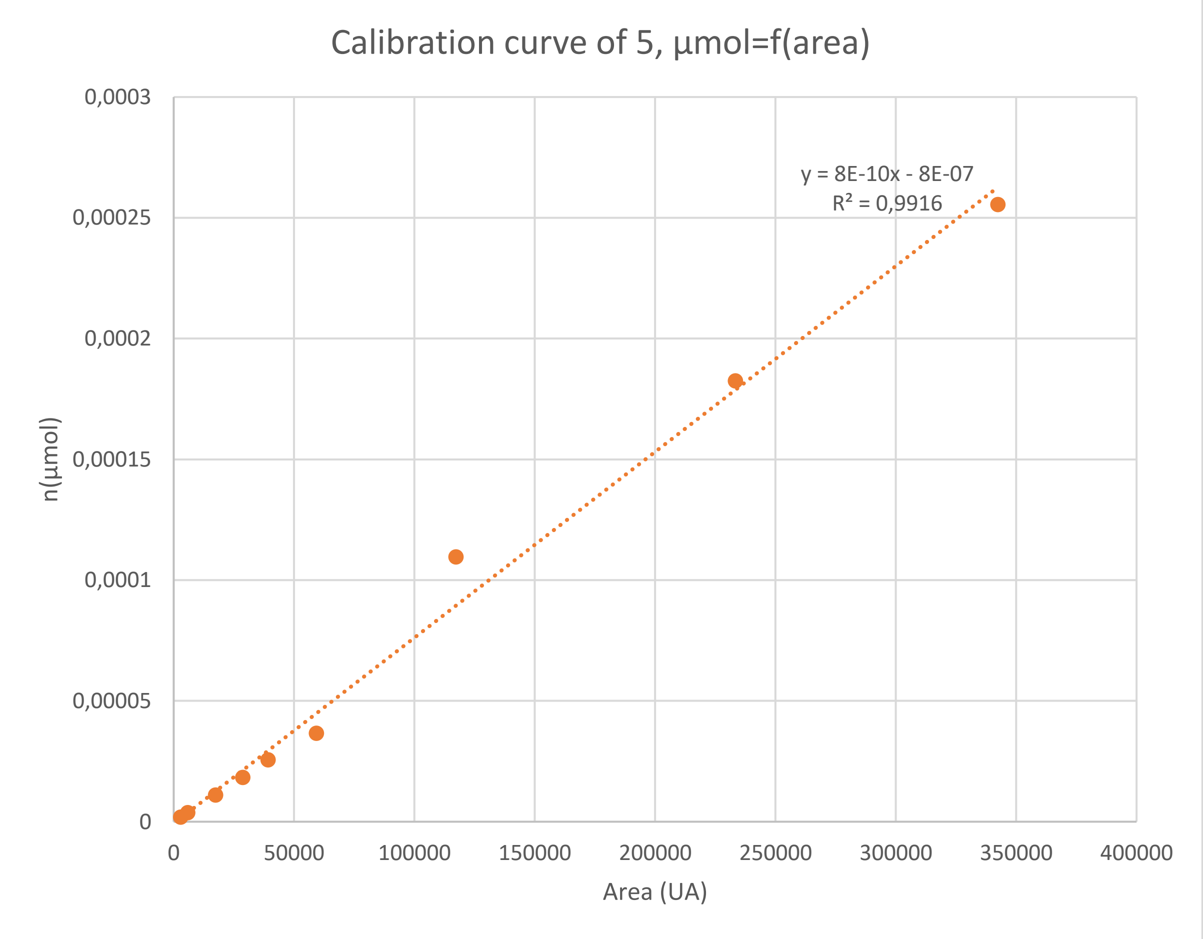

Figure S12 : Calibration curve of 5

The molar activity (dc) was of 44.4 ± 11.1 GBq/µmol (1.2 ± 0.3 Ci/µmol).

g. Mechanistic studies

The proposed mechanism of the reaction is represented in the following figure: (Figure S13)

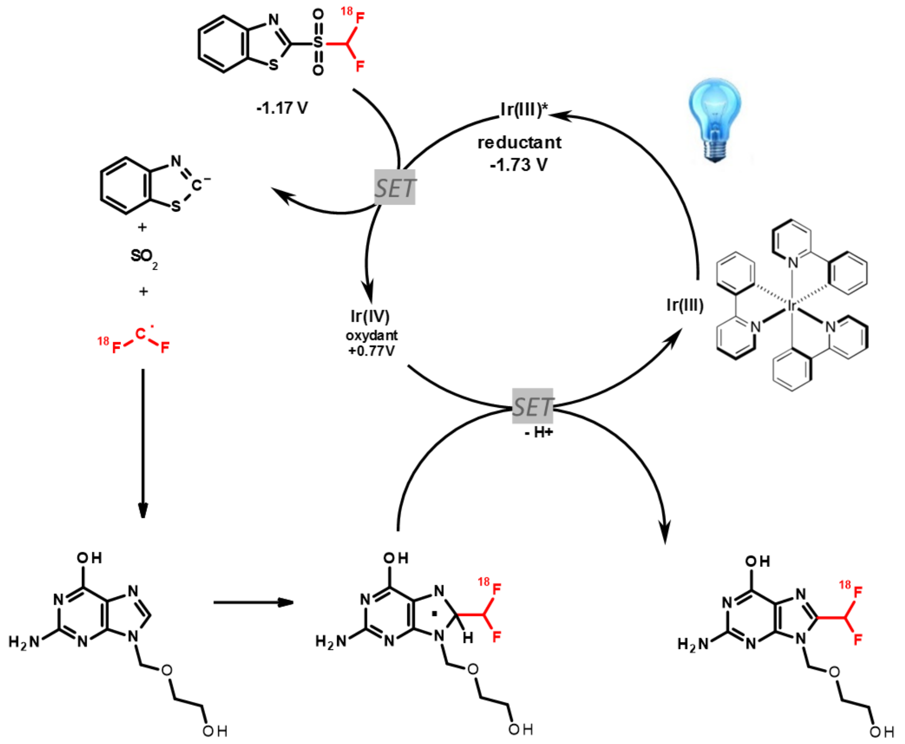

Figure S13 : Proposed mechanism

No product was obtained in the absence of light or photocatalyst (entries 1 and 2, Table S5) and in presence of TEMPO (entry 3), suggesting that radical species are involved, as proposed in the mechanism.

Table S5 : Tests for mechanistic studies

Entry	Deviation from usual conditions	RCY (%)
**1**	No catalyst	0
**2**	No light	0
**3**	TEMPO	0

h. General procedure for ^{18}F-difluoromethylation

A solution of the substrate (20 µmol), [Ir(ppy)$_3$] (0.01 µmol) in DMSO (200 µL) was prepared. Then the **[^{18}F]3** in DMSO (around 37 MBq/1 mCi) was added. The solution was injected in a 100 µL microchip, pumped with DMSO at a flow rate of 50 µL/min (residence time of 2 min) and irradiated under blue LED (470 nm, 2 W), at a temperature of 35°C .

The exited solution was analyzed by Radio-TLC and Radio-UPLC for radiochemical yield determination.

i. Scope

4-(difluoromethyl)-1H-indole [^{18}F]6

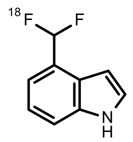

[^{18}F]6

General procedure using indole (2.3 mg, 20 µmol) yielded to 18 ± 1% as RCY (ndc, on crude product) of the title compound (see Table S6 and Figures S14 and S15 for radio LC of the labelled compound and LC analysis of the reference).

Table S6 : Radiochemical yield of [^{18}F]6

Reaction	Radio-TLC purity (%)	Radio-LC purity (%)	Radiochemical Yield (%)
**1**	60	32	19
**2**	59	30	18
**3**	61	30	18
**Radiochemical Yield + Deviation (%)**			18 ± 1

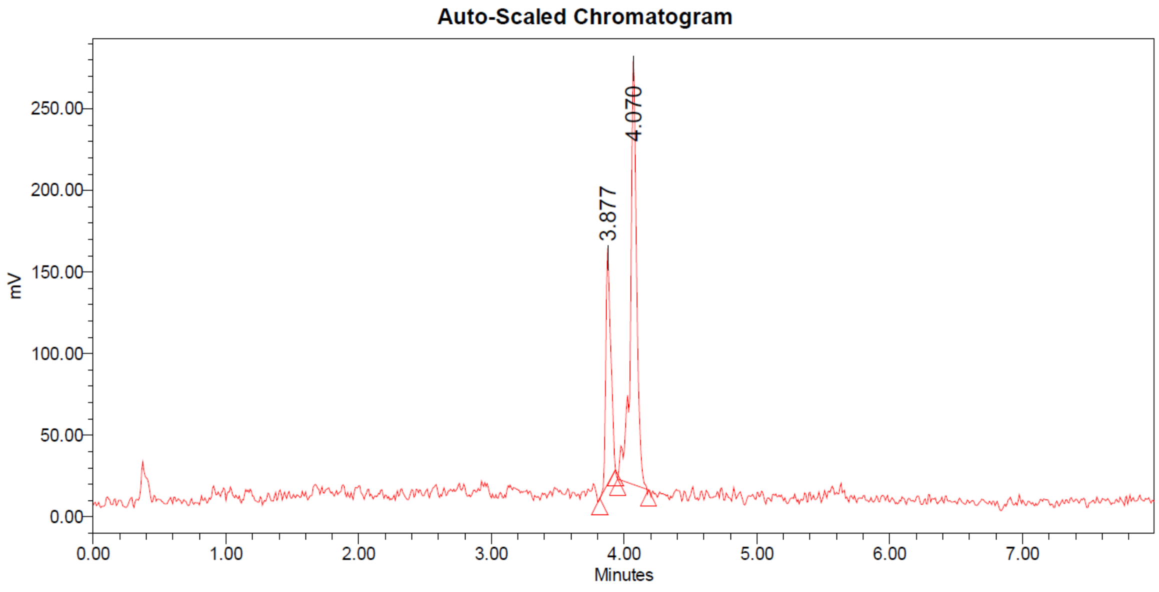

Figure S14 : UPLC radio-chromatogram of [^{18}F]6

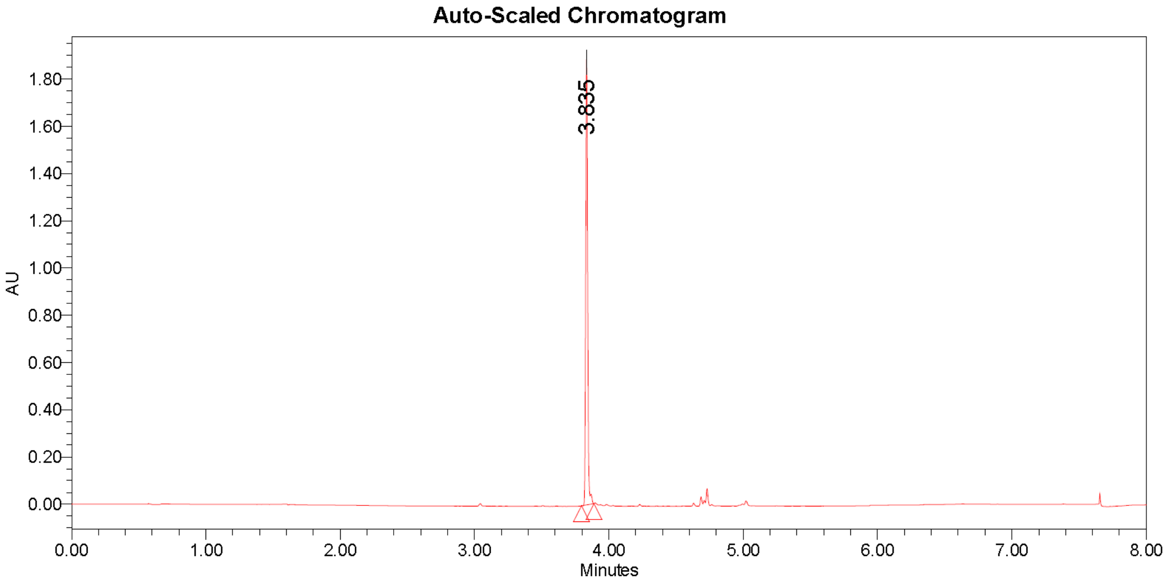

Figure S15 : UPLC UV-chromatogram of an authentic reference 6

2-(difluoromethyl)-1H-benzimidazole [^{18}F]7a; 4-(difluoromethyl)-1H-benzimidazole [^{18}F]7b ;

5-(difluoromethyl)-1H-benzimidazole [^{18}F]7c

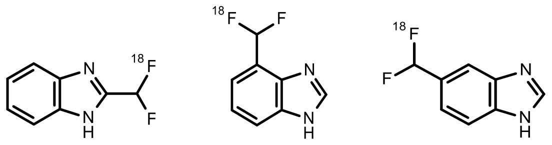

[^{18}F]7a **[^{18}F]7b** **[^{18}F]7c**

General procedure using benzimidazole (2.4 mg, 20 µmol) yielded respectively to 20 ± 1%, 41 ± 5 % and 10 ± 4 % as RCYs (ndc, on crude product) of the title compounds **[^{18}F]7a**, **[^{18}F]7b** and **[^{18}F]7c** (see Table S7 and Figures S16 to S18 for radio LC of the labelled compound and LC analysis of the references).

Table S7 : Radiochemical yield of [^{18}F]7

Reaction	Radio-TLC purity (%)	Radio-LC purity (%)			Radiochemical Yield (%)		
	a+b+c	a	b	c	a	b	c
**1**	72	27	65	8	19	47	6
**2**	64	32	55	13	21	36	8
**3**	70	27	53	20	19	37	14
**Radiochemical Yield + Deviation (%)**					20 ± 1	41 ± 5	10 ± 4

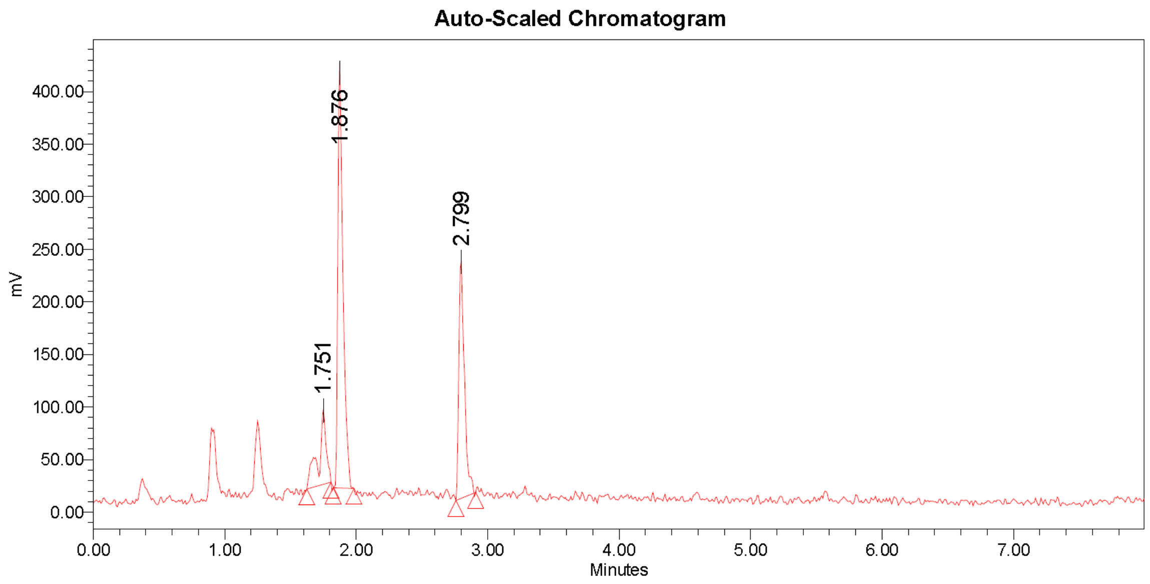

Figure S16 : UPLC radio-chromatogram of [^{18}F]7

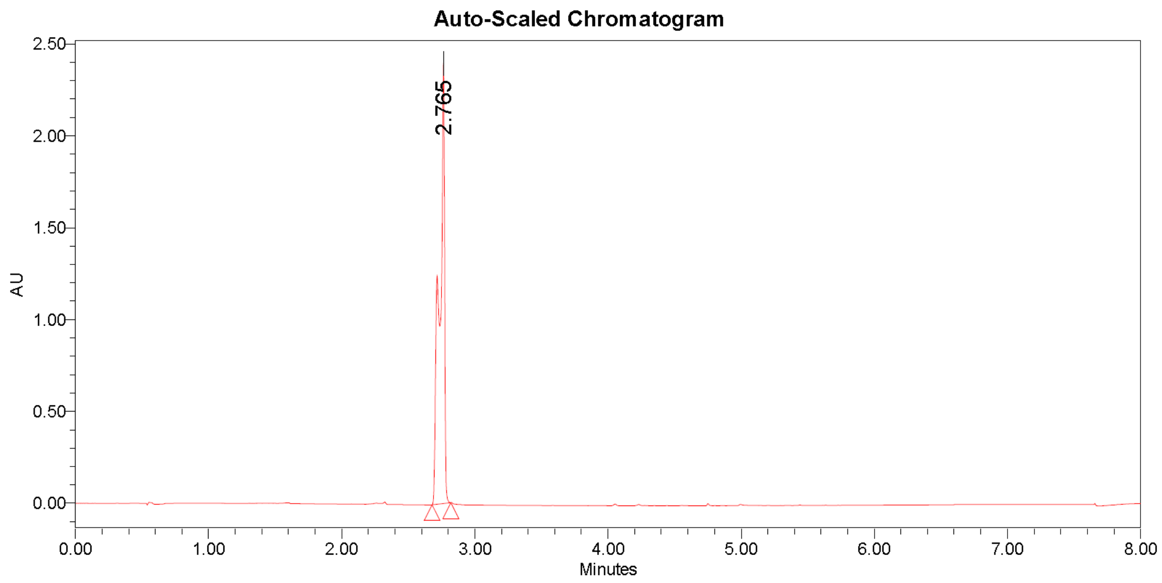

Figure S17 : UPLC UV-chromatogram of an authentic reference 7a

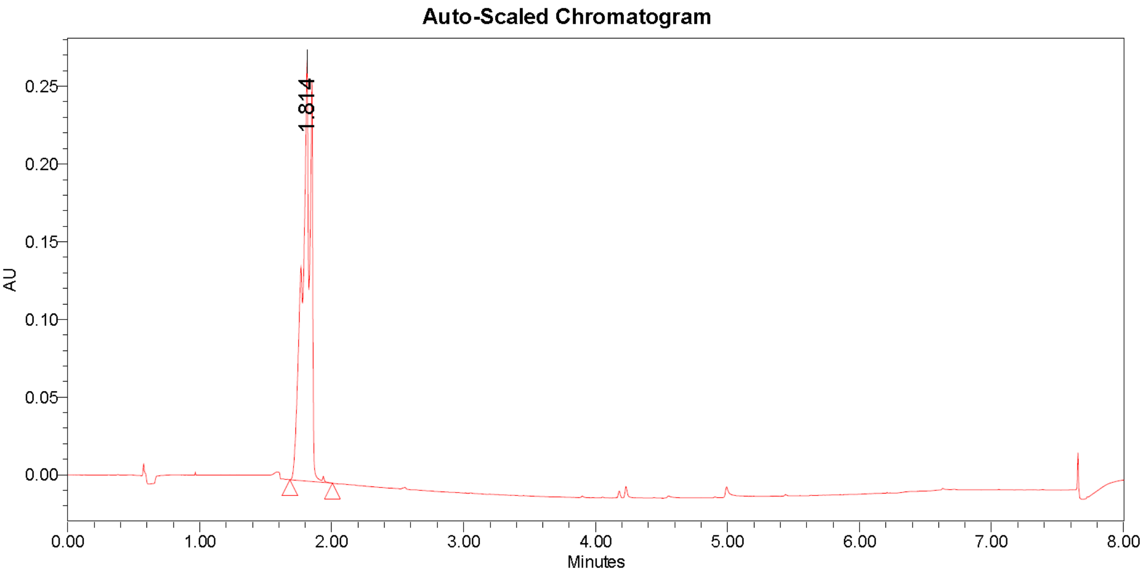

Figure S18 : UPLC UV-chromatogram of an authentic reference 7b

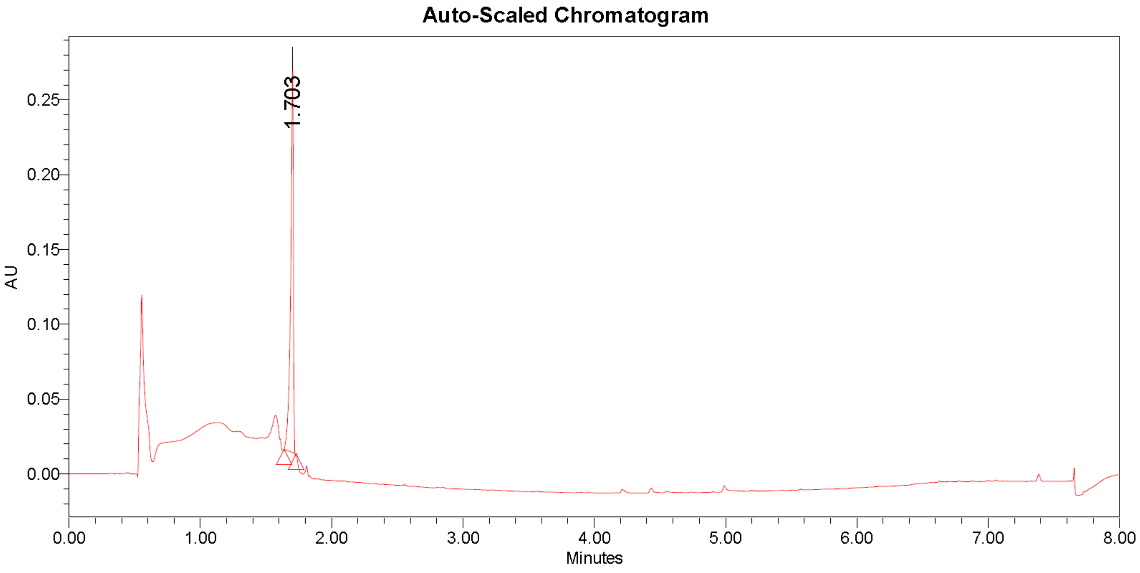

Figure S19 : UPLC UV-chromatogram of an authentic reference 7c

2-(difluoromethyl)-4-methyl-1H-pyrrolo[2,3-b]pyridine [^{18}F]8a; 6-(difluoromethyl)-4-methyl-1H-pyrrolo[2,3-b]pyridine [^{18}F]8b

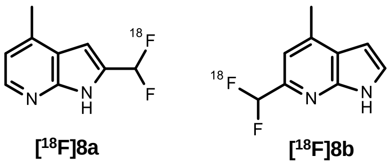

[^{18}F]8a [^{18}F]8b

General procedure using 4-methyl-1H-pyrrolo[2,3-b]pyridine (2.6 mg, 20 µmol) yielded respectively to 61 ± 1% and 7% as RCYs (ndc, on crude product) of the title compounds

[^{18}F]8a and **[^{18}F]8b** (see Table S8 and Figures S20 to S22 for radio LC of the labelled compound and LC analysis of the references).

Table S8 : Radiochemical yield of [^{18}F]8

Reaction	Radio-TLC purity (%)	Radio-LC purity (%)		Radiochemical Yield (%)	
	a+b	a	b	a	b
**1**	65	90	10	59	7
**2**	69	90	10	62	7
**3**	68	90	10	61	7
**Radiochemical Yield + Deviation (%)**				61 ± 1	7

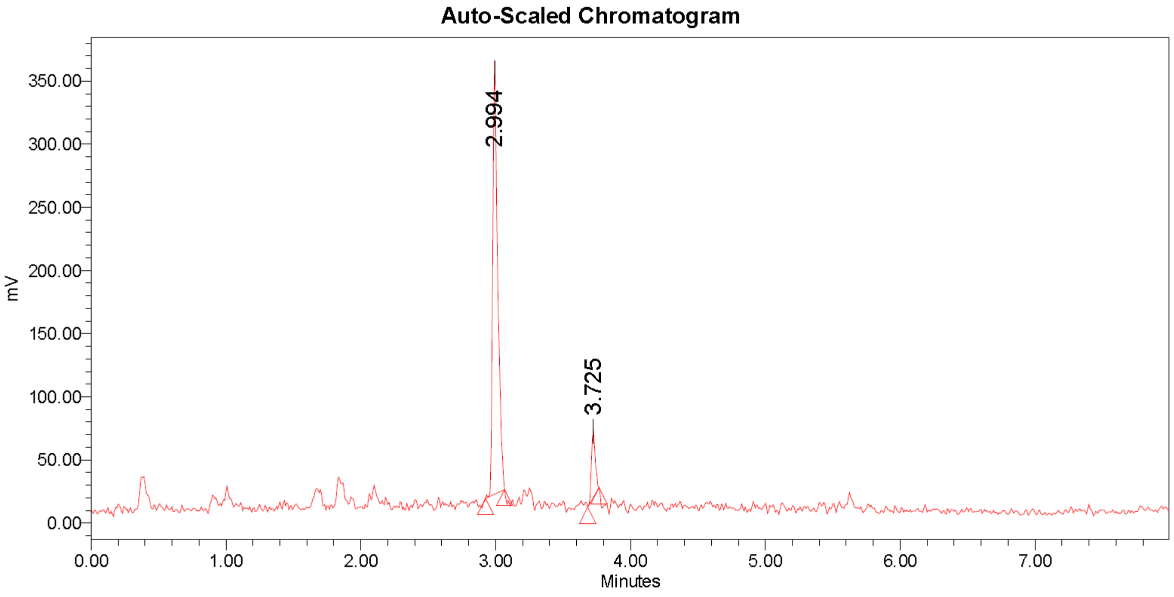

Figure S20 : UPLC radio-chromatogram of [^{18}F]8

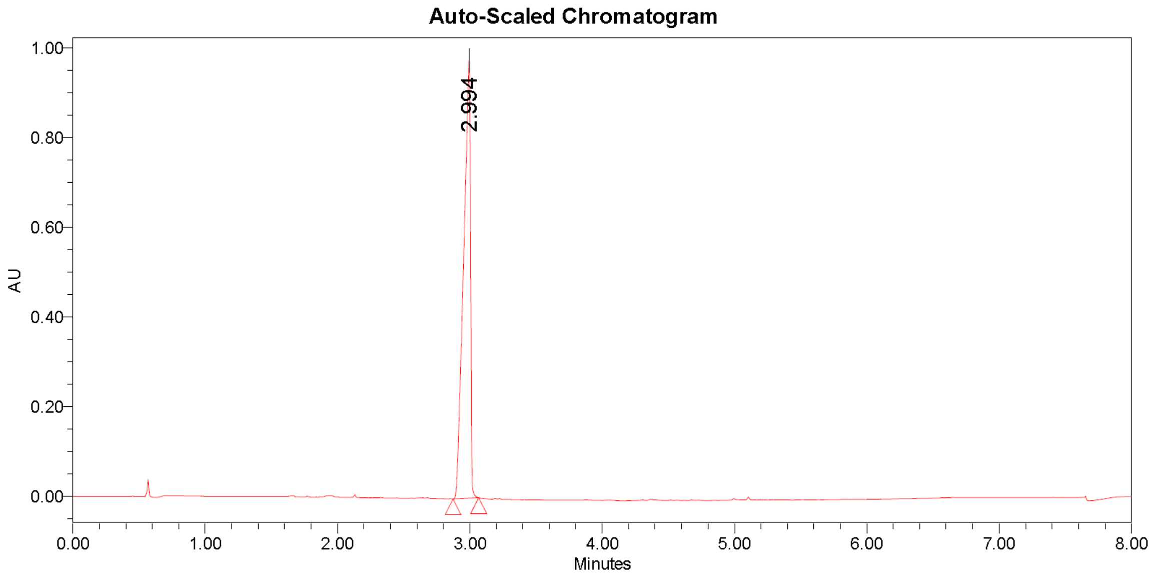

Figure S21 : UPLC UV-chromatogram of an authentic reference 8a

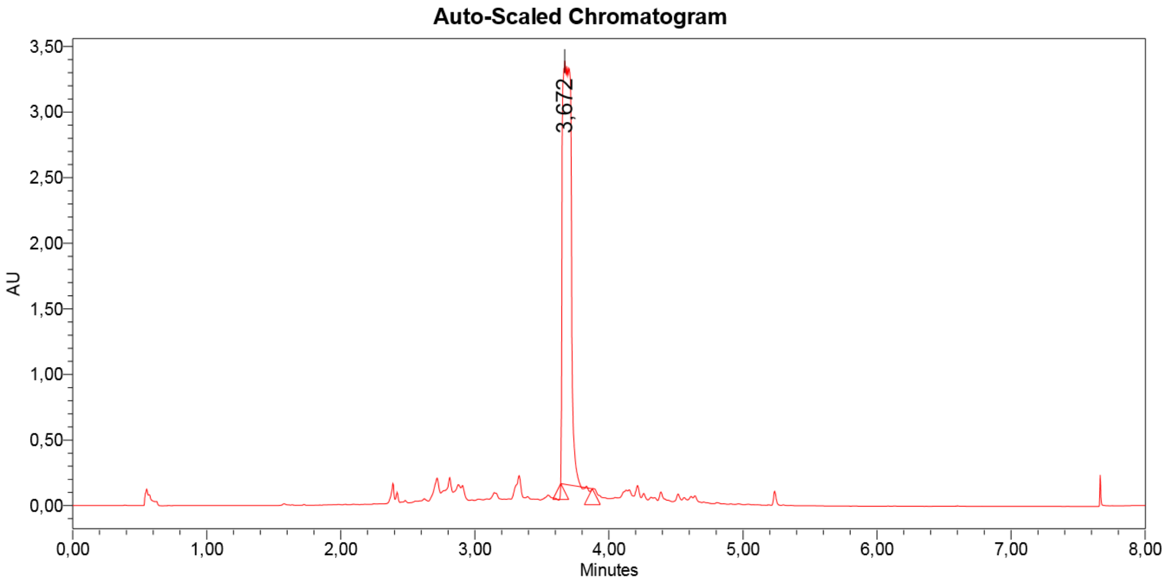

Figure S22 : UPLC UV-chromatogram of an authentic reference 8b

4-(difluoromethyl)-6-methyl-1H-pyrazolo[3,4-b]pyridine [^{18}F]9a; 3-(difluoromethyl)-6-methyl-1H-pyrazolo[3,4-b]pyridine [^{18}F]9b

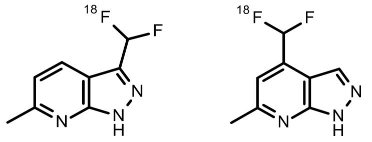

[^{18}F]9a **[^{18}F]9b**

General procedure using 6-methyl-1H-pyrazolo[3,4-b]pyridine (2.2 mg, 20 µmol) yielded respectively to 20 ± 1% and 54 ± 2% as RCYs (ndc, on crude product) of the title compounds **[^{18}F]9a** and **[^{18}F]9b** (see Table S9 and Figures S23 to S25 for radio LC of the labelled compound and LC analysis of the references).

Table S9 : Radiochemical yield of [^{18}F]9

Reaction	Radio-TLC purity (%)	Radio-LC purity (%)		Radiochemical Yield (%)	
	a + b	a	b	a	b
**1**	74	25	75	19	56
**2**	73	29	71	21	52
**3**	75	28	72	20	54
**Radiochemical Yield + Deviation (%)**				20 ± 1	54 ± 2

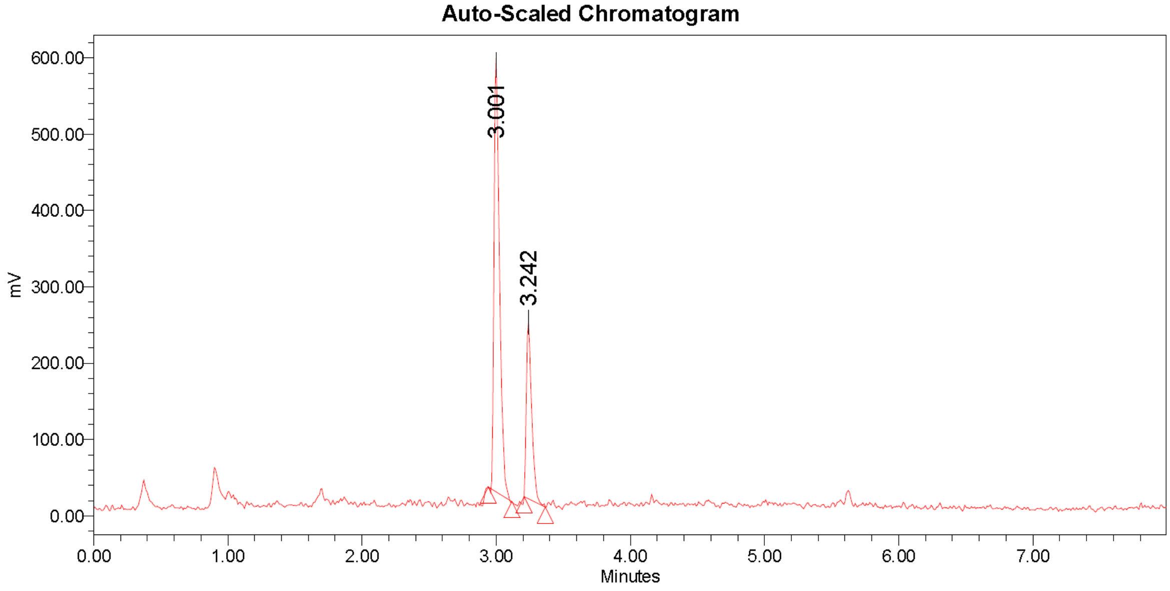

Figure S23 : UPLC radio-chromatogram of [^{18}F]9

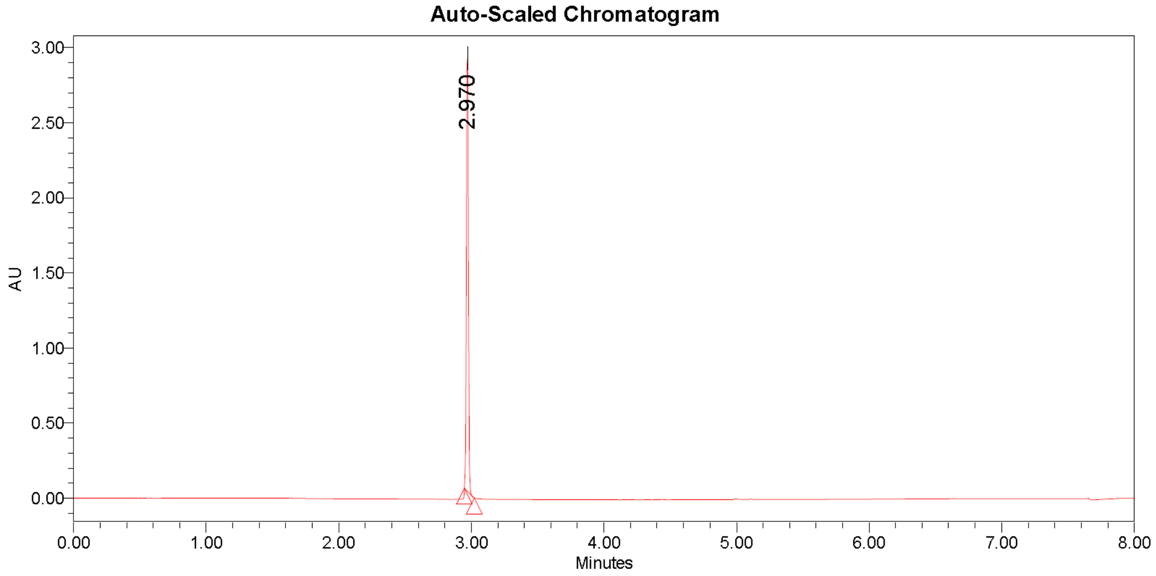

Figure S24 : UPLC UV-chromatogram of an authentic reference 9a

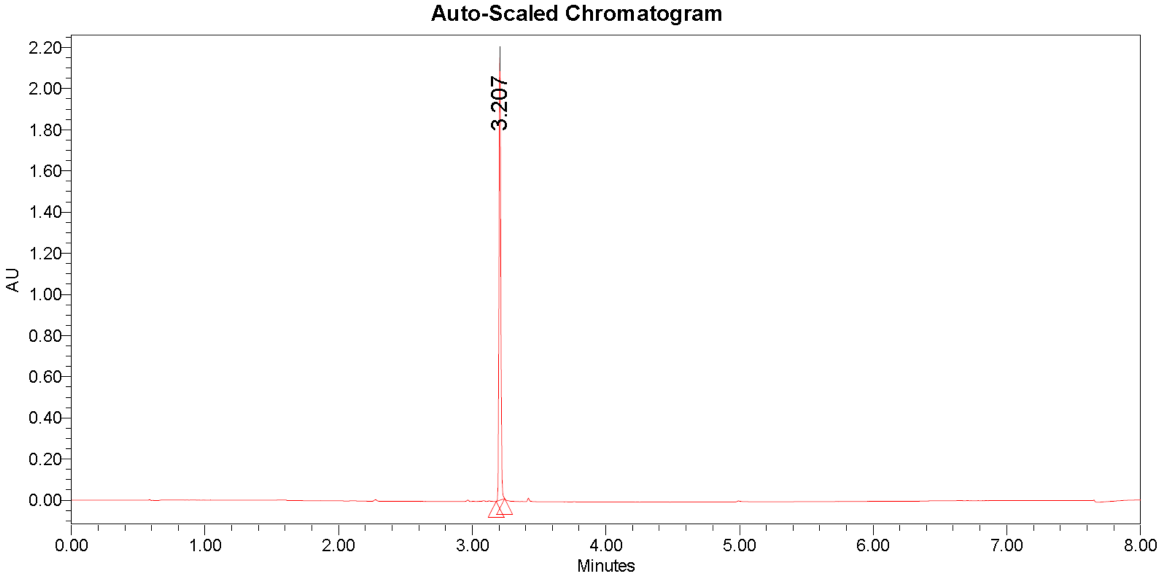

Figure S25 : UPLC UV-chromatogram of an authentic reference 9b

2-[3,5-dichloro-2-(difluoromethyl)-4-pyridyl]-N,N-dimethyl-acetamide [^{18}F]10

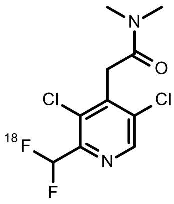

[^{18}F]10

General procedure using 2-(3,5-dichloro-4-pyridyl)-N,N-dimethyl-acetamide (4.7 mg, 20 µmol) yielded to 57 ± 5% as RCY (ndc, on crude product) of the title compound (see Table S10 and Figures S26 and S27 for radio LC of the labelled compound and LC analysis of the reference).

Table S10 : Radiochemical yield of [^{18}F]10

Reaction	Radio-TLC purity (%)	Radio-LC purity (%)	Radiochemical Yield (%)
**1**	75	82	62
**2**	71	73	52
**3**	65	93	60
**Radiochemical Yield + Deviation (%)**			57 ± 5

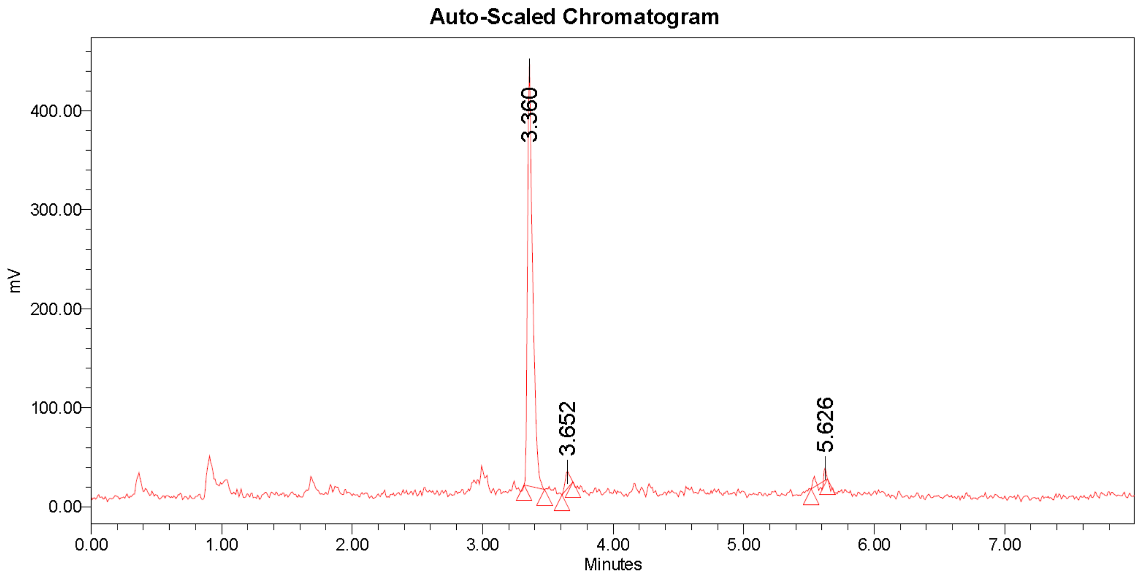

Figure S26 : UPLC radio-chromatogram of [^{18}F]10

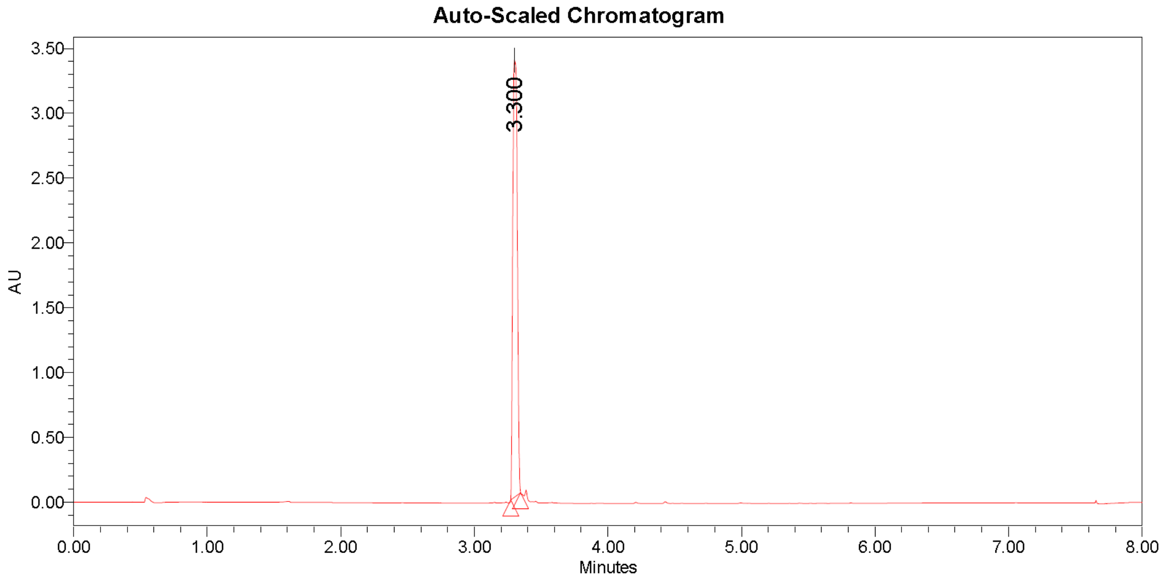

Figure S27 : UPLC UV-chromatogram of an authentic reference 10

3-chloro-1-(difluoromethyl)-6,7-dihydro-5H-cyclopenta[c]pyridine-4-carbonitrile [^{18}F]11

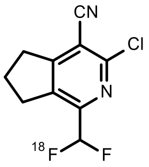

[^{18}F]11

General procedure using 3-chloro-6,7-dihydro-5H-cyclopenta[c]pyridine-4-carbonitrile (3.6 mg, 20 µmol) yielded to 45 ± 2% as RCY (ndc, on crude product) of the title compound (see

Table S11 and Figures S28 and S29 for radio LC of the labelled compound and LC analysis of the reference).

Table S11 : Radiochemical yield of [^{18}F]11

Reaction	Radio-TLC purity (%)	Radio-LC purity (%)	Radiochemical Yield (%)
**1**	50	95	47
**2**	43	100	43
**3**	46	100	46
**Radiochemical Yield + Deviation (%)**			45 ± 2

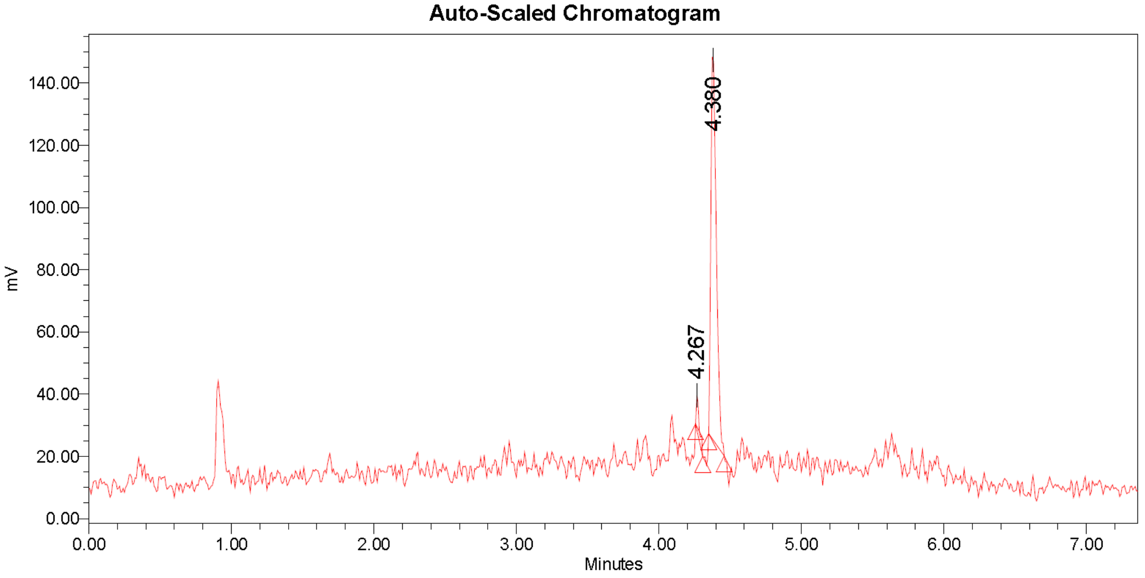

Figure S28 : UPLC radio-chromatogram of [^{18}F]11

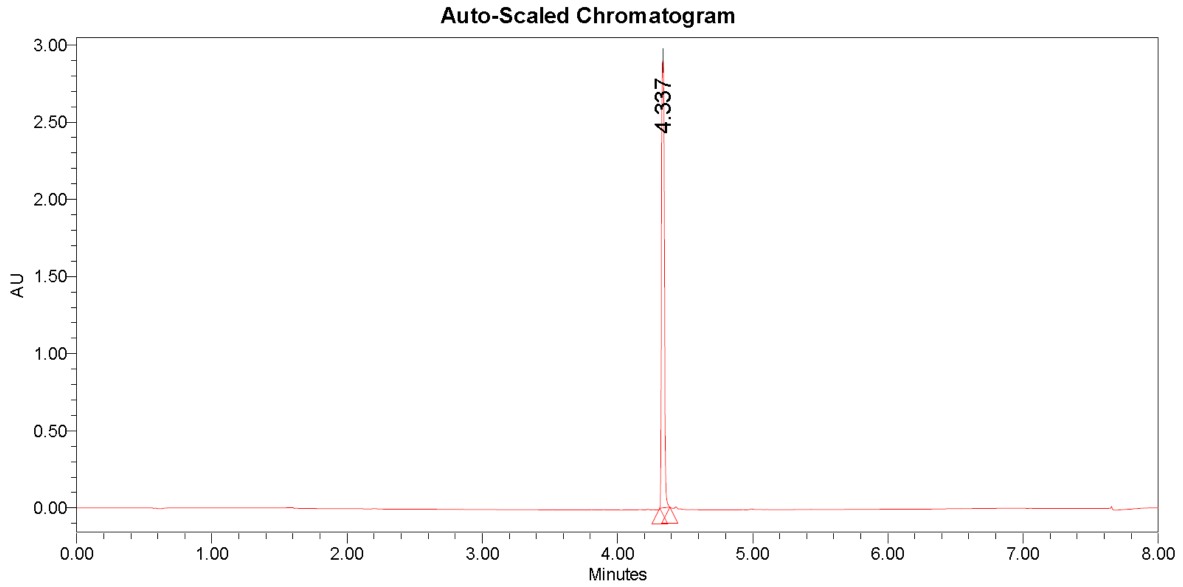

Figure S29 : UPLC UV-chromatogram of an authentic reference 11

2-chloro-4-(difluoromethyl)-6,7-dihydro-5H-cyclopenta[b]pyridine-3-carbonitrile [^{18}F]12

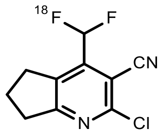

[^{18}F]12

General procedure using 2-chloro-6,7-dihydro-5H-cyclopenta[b]pyridine-3-carbonitrile (3.6 mg, 20 µmol) yielded to 42 ± 13% as RCY (ndc, on crude product) of the title compound (see Table S12 and Figures S30 and S31 for radio LC of the labelled compound and LC analysis of the reference).

Table S12 : Radiochemical yield of [^{18}F]12

Reaction	Radio-TLC purity (%)	Radio-LC purity (%)	Radiochemical Yield (%)
**1**	45	100	45
**2**	47	62	29
**3**	63	87	55
**Radiochemical Yield + Deviation (%)**			42 ± 13

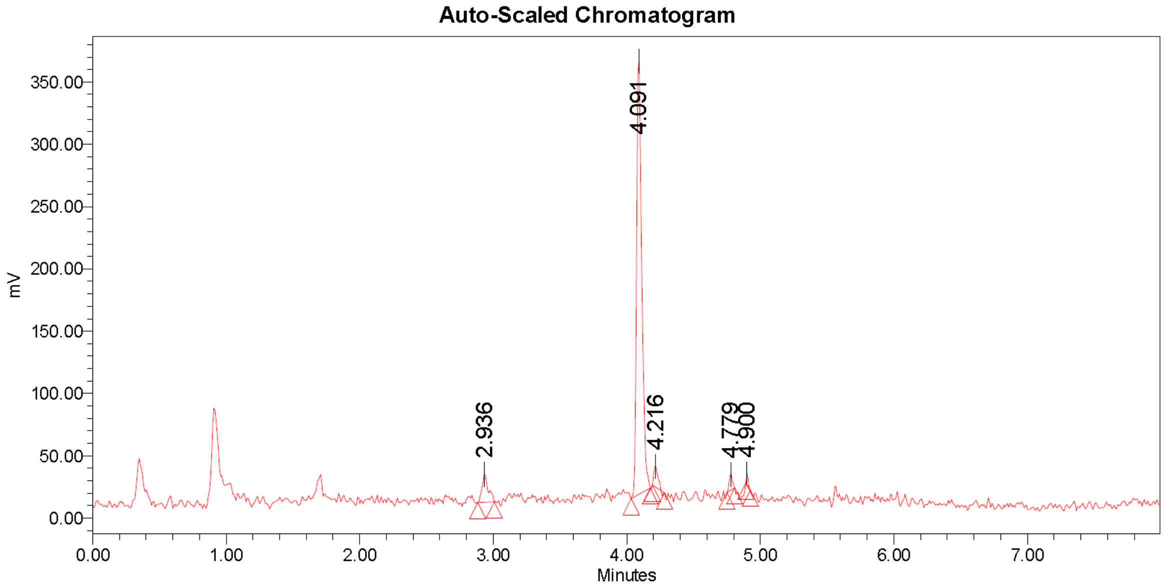

Figure S30 : UPLC radio-chromatogram of [^{18}F]12

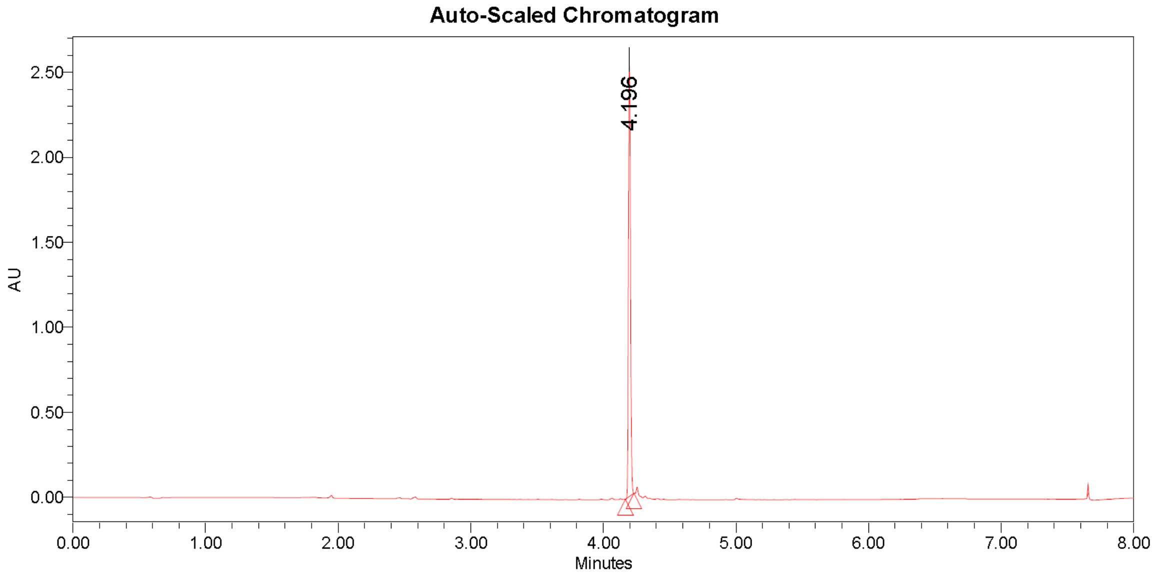

Figure S31 : UPLC UV-chromatogram of an authentic reference 12

4-(difluoromethyl)-2-tetrahydropyran-4-yl-pyrimidin-5-amine [^{18}F]13

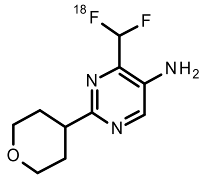

[^{18}F]13

General procedure using 2-tetrahydropyran-4-ylpyrimidin-5-amine (3.6 mg, 20 µmol) yielded to 69 ± 3% as RCY (ndc, on crude product) of the title compound (see Table S13 and Figures S32 and S33 for radio LC of the labelled compound and LC analysis of the reference).

Table S13 : Radiochemical yield of [^{18}F]13

Reaction	Radio-TLC purity (%)	Radio-LC purity (%)	Radiochemical Yield (%)
**1**	71	100	71
**2**	67	98	66
**3**	72	99	71
**Radiochemical Yield + Deviation (%)**			69 ± 3

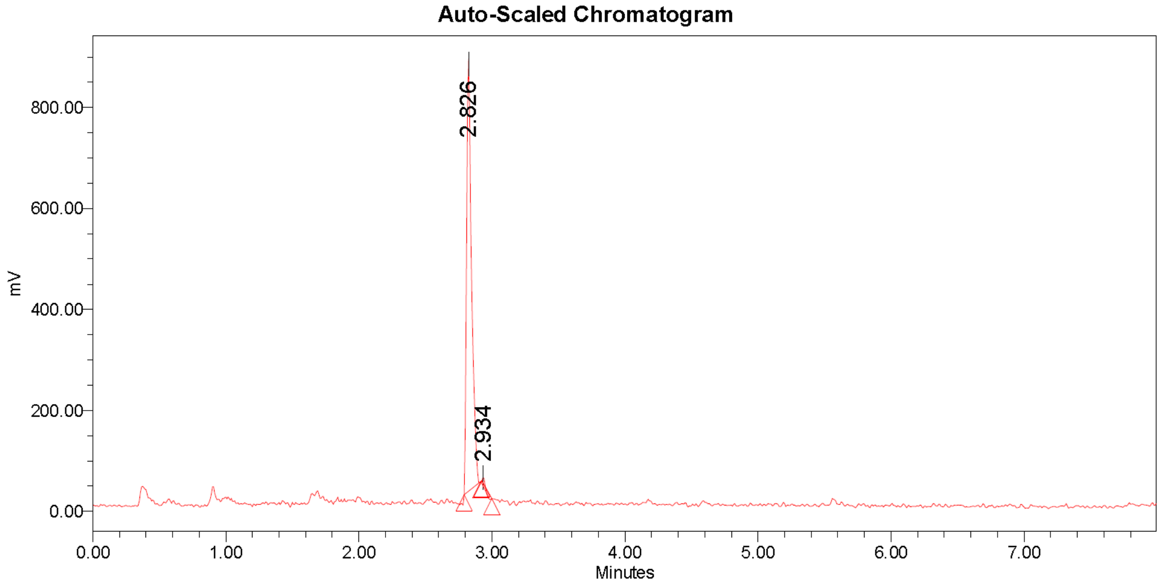

Figure S32 : UPLC radio-chromatogram of [^{18}F]13

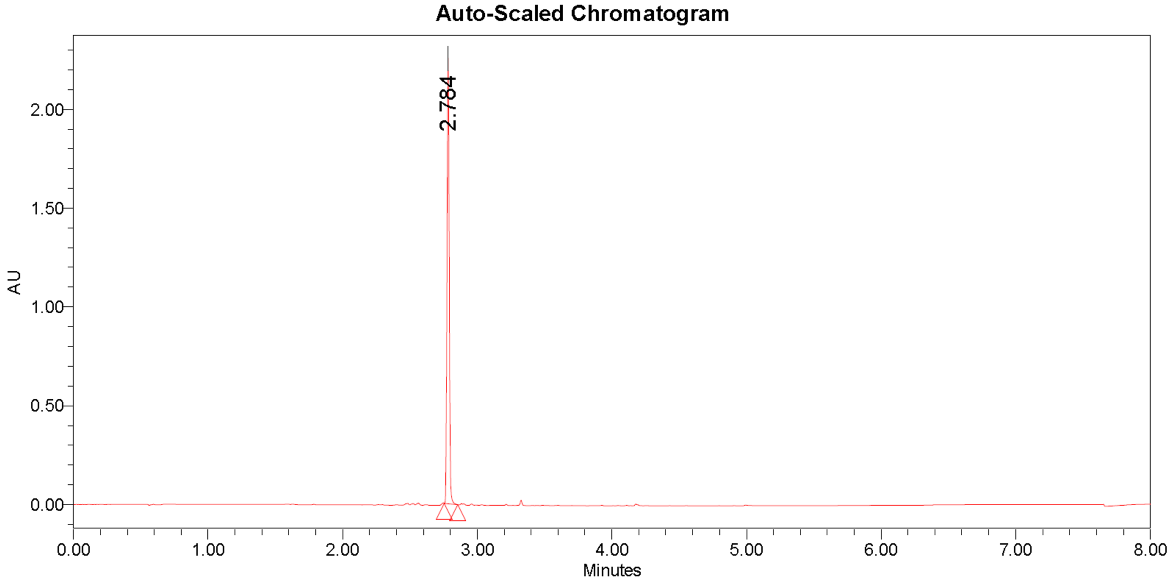

Figure S33 : UPLC UV-chromatogram of an authentic reference 13

4-chloro-2-(difluoromethyl)-6-methoxy-pyrimidin-5-amine [^{18}F]14

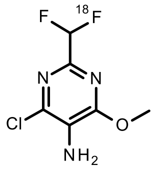

[^{18}F]14

General procedure using 4-chloro-6-methoxy-pyrimidin-5-amine (3.2 mg, 20 µmol) yielded to 57 ± 6 % as RCY (ndc, on crude product) of the title compound (see Table S14 and Figures S34 and S35 for radio LC of the labelled compound and LC analysis of the reference).

Table S14 : Radiochemical yield of [^{18}F]14

Reaction	Radio-TLC purity (%)	Radio-LC purity (%)	Radiochemical Yield (%)
**1**	57	90	51
**2**	69	90	62
**3**	76	85	64
**Radiochemical Yield + Deviation (%)**			57 ± 6

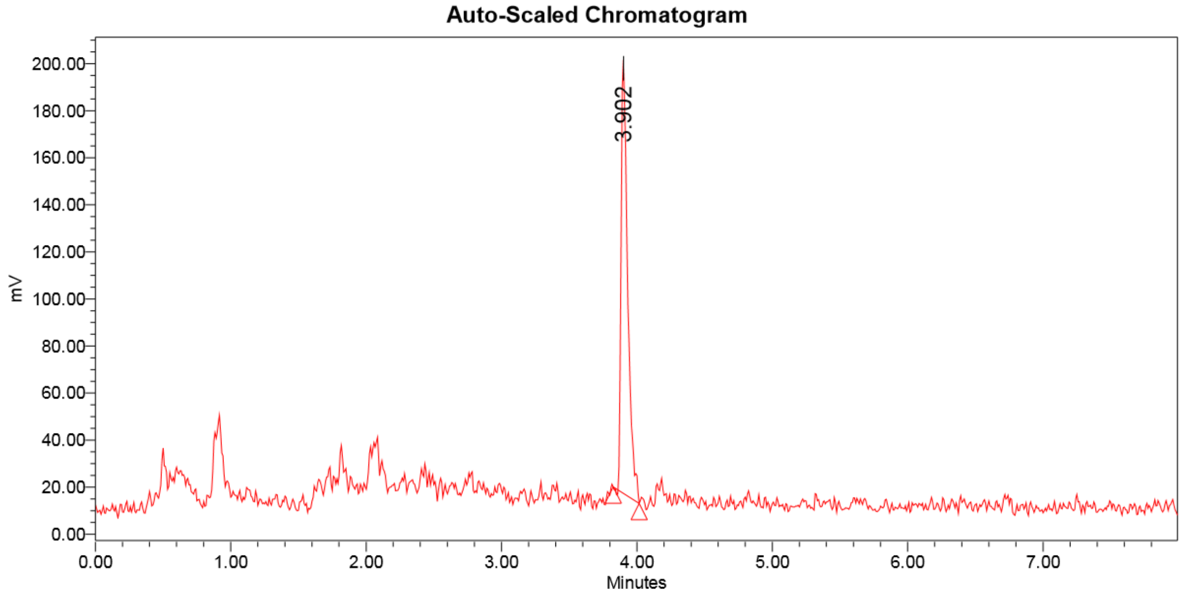

Figure S34 : UPLC radio-chromatogram of [^{18}F]14

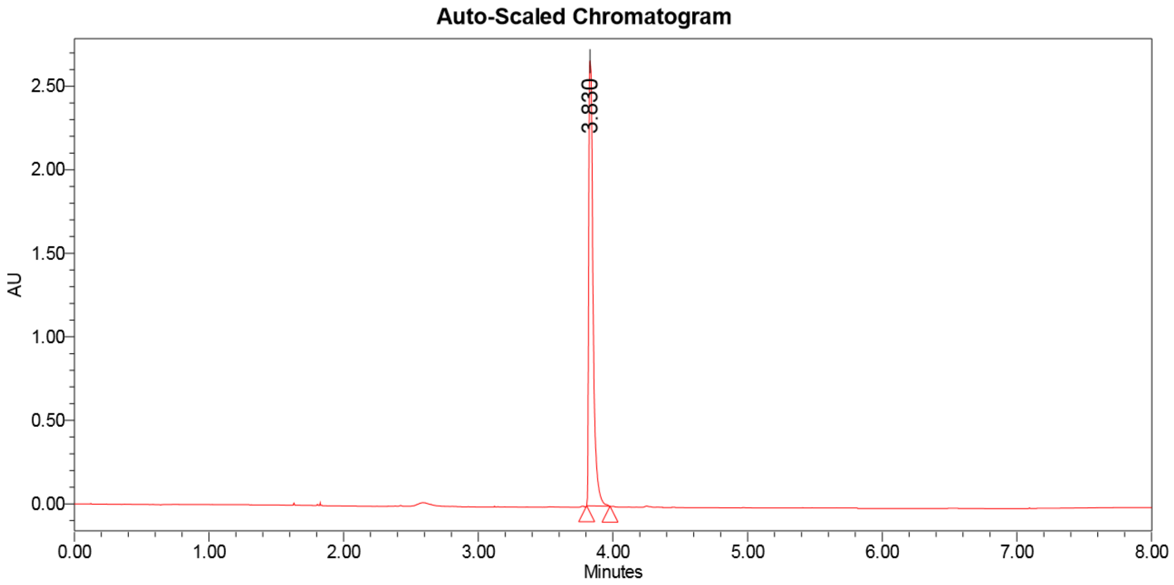

Figure S35 : UPLC UV-chromatogram of an authentic reference 14

4-(difluoromethyl)-5-methyl-pyrimidin-2-amine [^{18}F]15

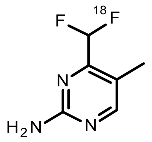

[^{18}F]15

General procedure using 5-methylpyrimidin-2-amine (2.2 mg, 20 µmol) yielded to 43 ± 3% as RCY (ndc, on crude product) of the title compound (see Table S15 and Figures S36 and S37 for radio LC of the labelled compound and LC analysis of the reference).

Table S15 : Radiochemical yield of [^{18}F]15

Reaction	Radio-TLC purity (%)	Radio-LC purity (%)	Radiochemical Yield (%)
**1**	41	100	41
**2**	42	100	42
**3**	46	100	46
**Radiochemical Yield + Deviation (%)**			43 ± 3

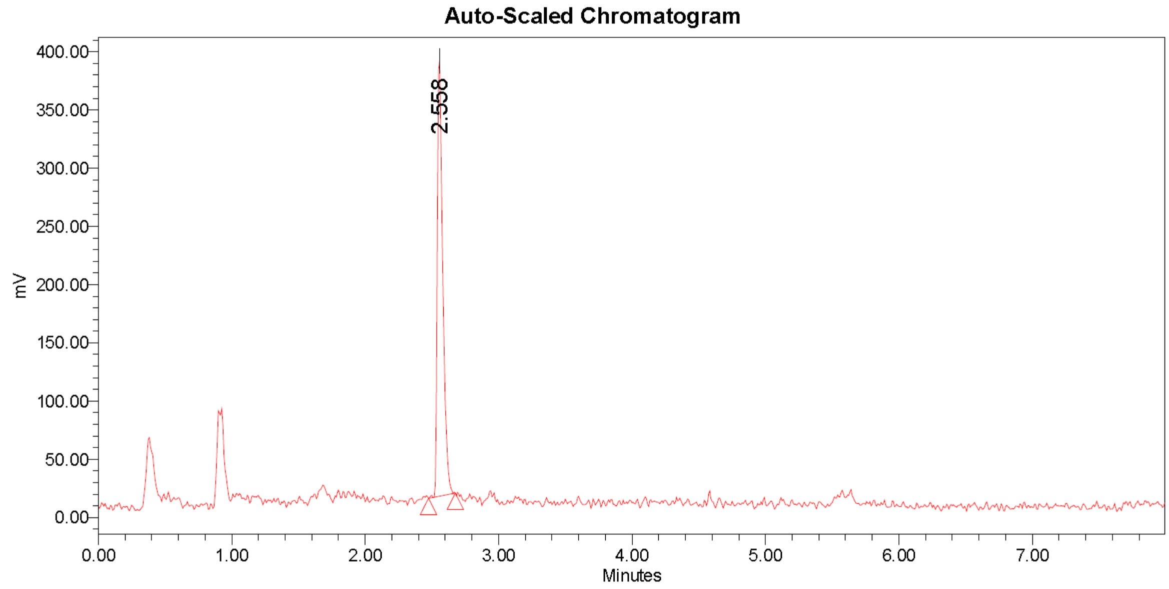

Figure S36 : UPLC radio-chromatogram of [^{18}F]15

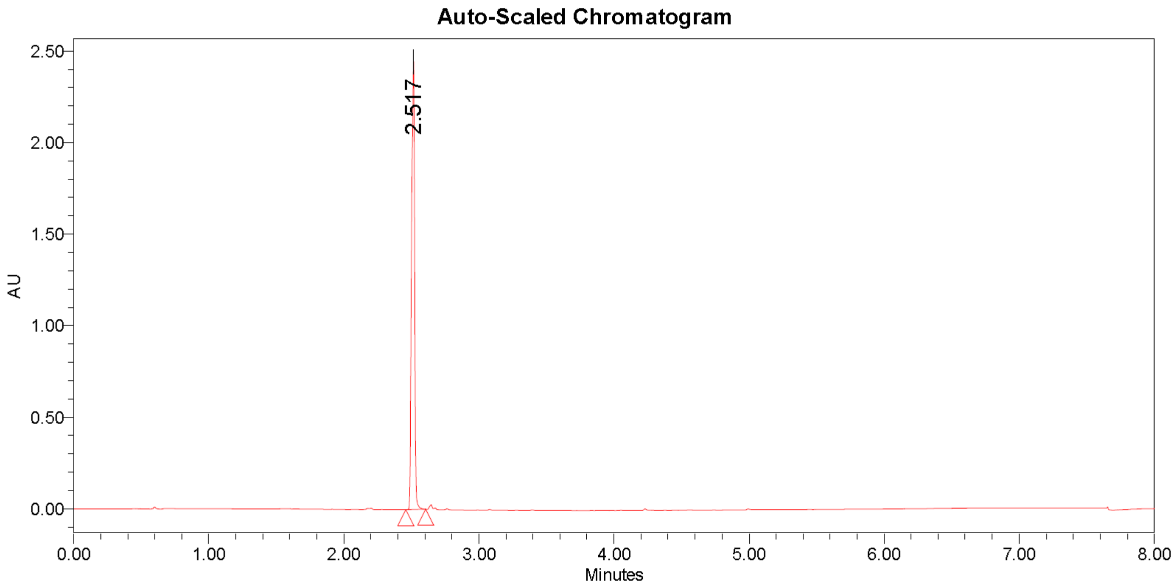

Figure S37 : UPLC UV-chromatogram of an authentic reference 15

4-(difluoromethyl)-2-methyl-6,8-dihydro-5H-pyrido[2,3-d]pyrimidin-7-one [^{18}F]16

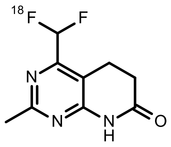

[^{18}F]16

General procedure using 2-methyl-6,8-dihydro-5H-pyrido[2,3-d]pyrimidin-7-one (3.3 mg, 20 µmol), with a residence time of 4 min and [Ir(ppy)$_3$] (0.05 µmol) yielded to 32 ± 4% as RCY (ndc, on crude product) of the title compound (see Table S16 and Figures S38 and S39 for radio LC of the labelled compound and LC analysis of the reference).

Table S16 : Radiochemical yield of [^{18}F]16

Reaction	Radio-TLC purity (%)	Radio-LC purity (%)	Radiochemical Yield (%)
**1**	53	66	36
**2**	59	46	27
**3**	55	60	33
**Radiochemical Yield + Deviation (%)**			32 ± 4

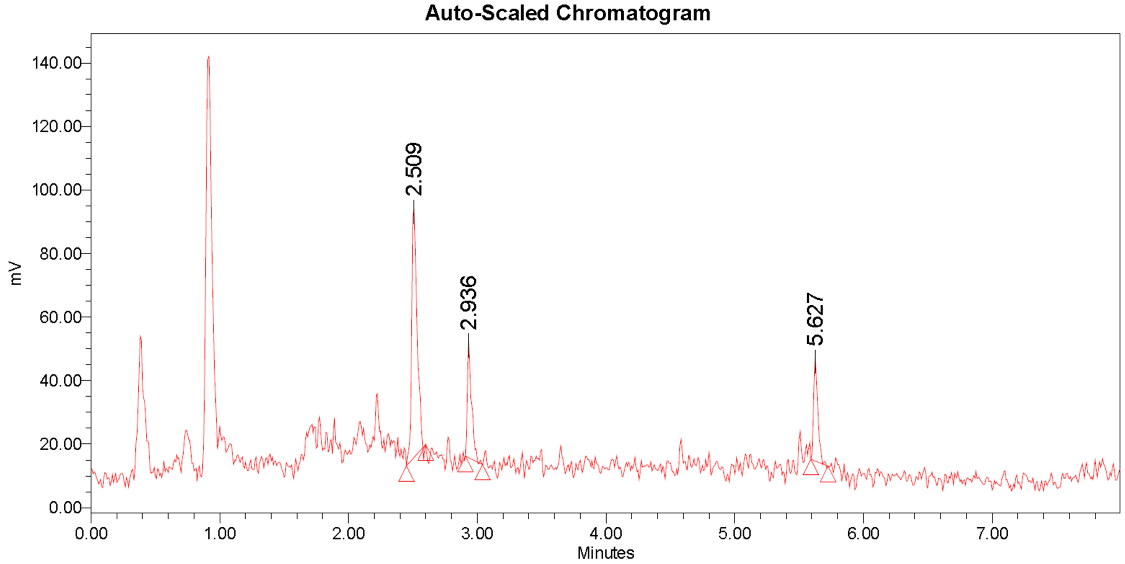

Figure S38 : UPLC radio-chromatogram of [^{18}F]16

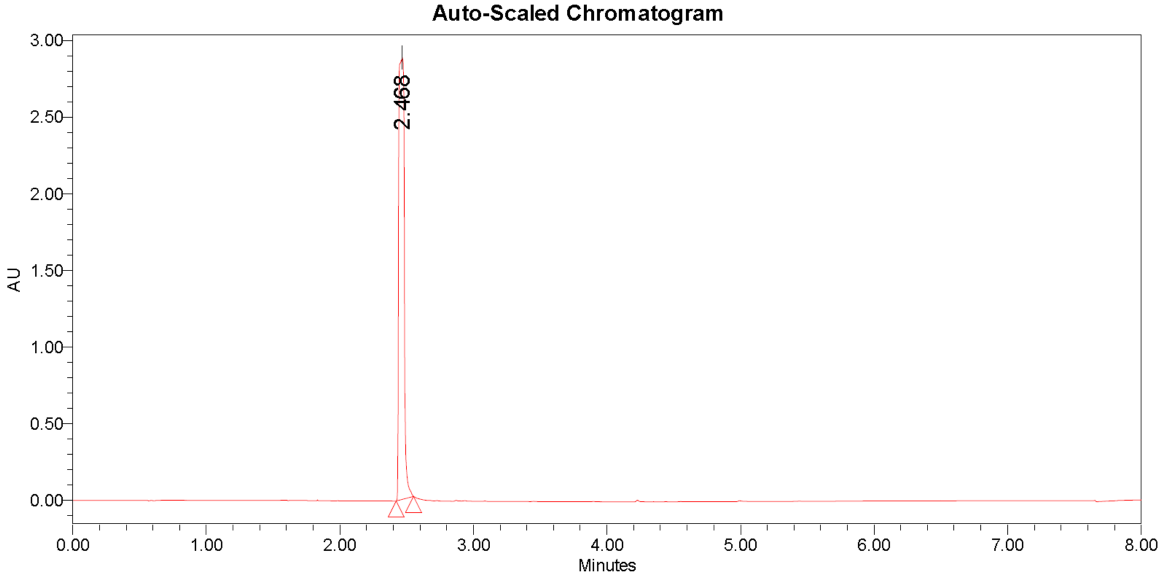

Figure S39 : UPLC UV-chromatogram of an authentic reference 16

5-(difluoromethyl)-1H-pyrimido[4,5-d]pyridazine-2,4-dione, 8-(difluoromethyl)-1H-pyrimido[4,5-d]pyridazine-2,4-dione [^{18}F]17

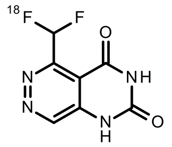

[^{18}F]17

General procedure using 1H-pyrimido[4,5-d]pyridazine-2,4-dione (3.3 mg, 20 µmol) with a residence time of 4 min and [Ir(ppy)$_3$] (0.05 µmol) yielded to 75 ± 1% as RCY (ndc, on crude product) of the title compound (see Table S17 and Figures S40 and S41 for radio LC of the labelled compound and LC analysis of the reference).

Table S17 : Radiochemical yield of [^{18}F]17

Reaction	Radio-TLC purity (%)	Radio-LC purity (%)	Radiochemical Yield (%)
**1**	79	96	76
**2**	76	96	74
**3**	77	96	74
**Radiochemical Yield + Deviation (%)**			75 ± 1

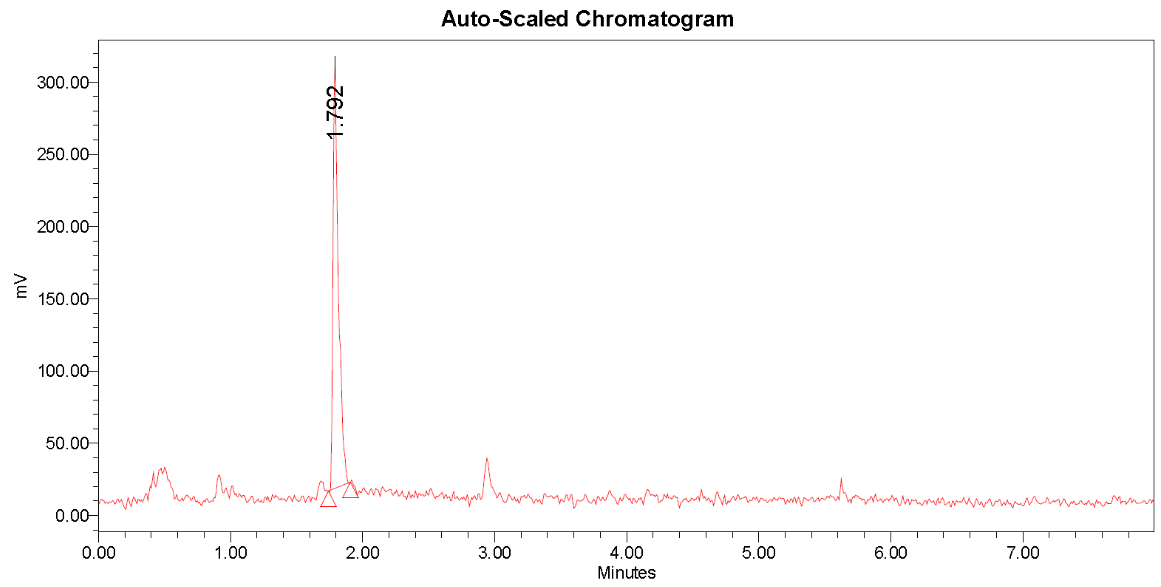

Figure S40 : UPLC radio-chromatogram of [^{18}F]17

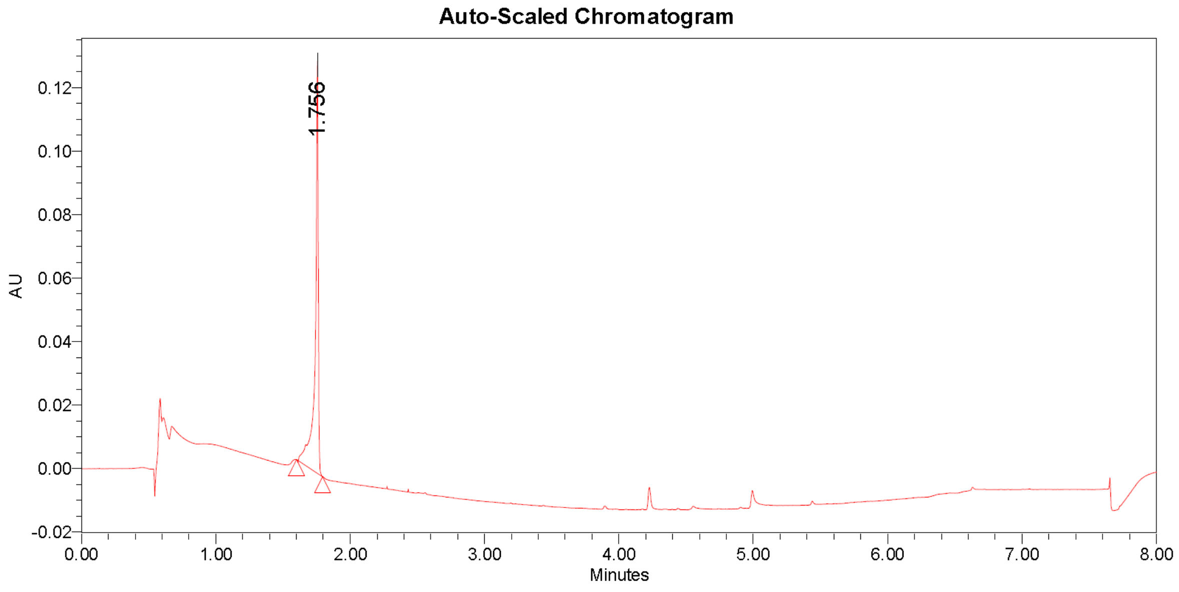

Figure S41 : UPLC UV-chromatogram of an authentic reference 17

Methyl 2-[3,5-dichloro-6-(difluoromethyl)-1-tetrahydropyran-2-yl-pyrazolo[3,4-b]pyridin-4-yl]acetate [^{18}F]18

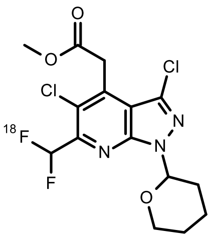

[^{18}F]18

General procedure using methyl 2-(3,5-dichloro-1-tetrahydropyran-2-yl-pyrazolo[3,4-b]pyridin-4-yl)acetate (6.9 mg, 20 µmol), with a residence time of 4 min and [Ir(ppy)$_3$] (0.05 µmol) yielded to 38 ± 5% as RCY (ndc, on crude product) of the title compound (see Table S18 and Figures S42 and S43 for radio LC of the labelled compound and LC analysis of the reference).

Table S18 : Radiochemical yield of [^{18}F]18

Reaction	Radio-TLC purity (%)	Radio-LC purity (%)	Radiochemical Yield (%)
**1**	74	60	44
**2**	76	43	33
**3**	69	58	40
**Radiochemical Yield + Deviation (%)**			38 ± 5

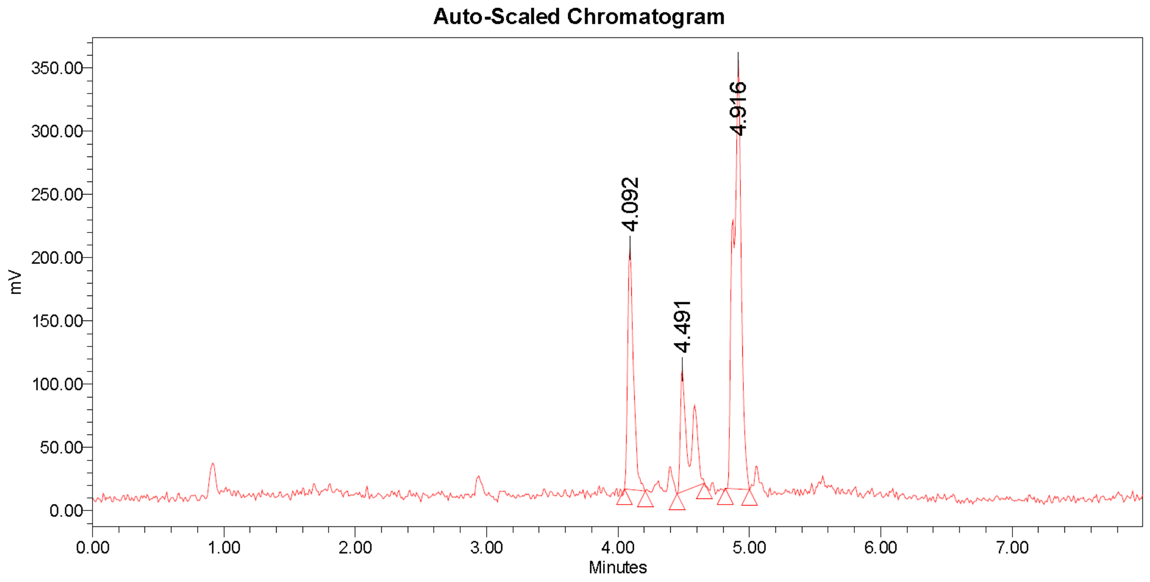

Figure S42 : UPLC radio-chromatogram of [^{18}F]18

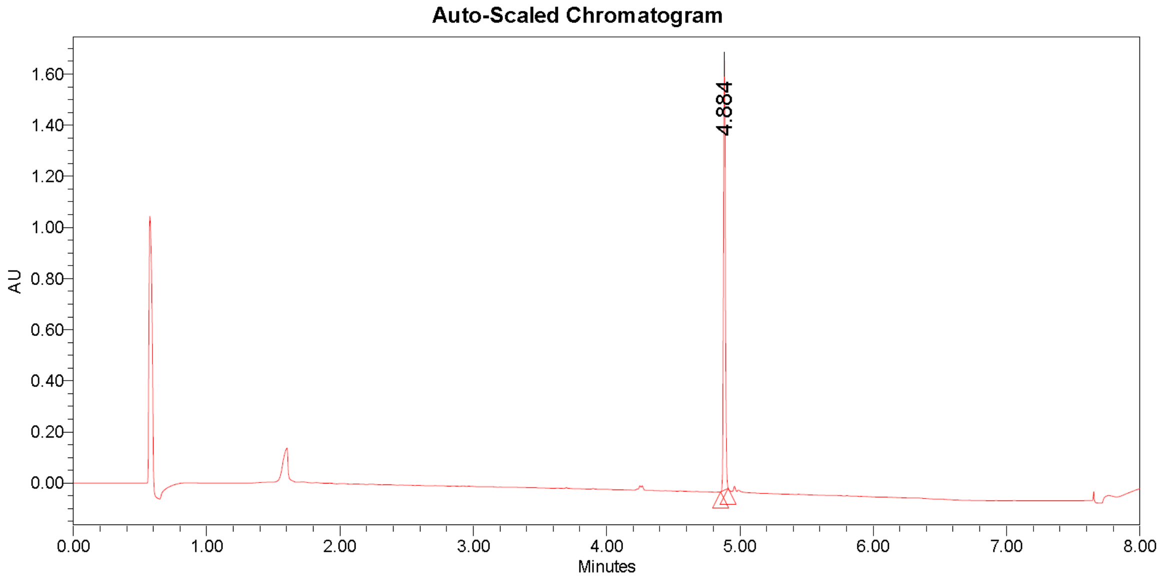

Figure S43 : UPLC UV-chromatogram of an authentic reference 18

4-(difluoromethyl)-6-methyl-2-methylsulfanyl-8H-pyrimido[4,5-d]pyrimidine-5,7-dione [^{18}F]19

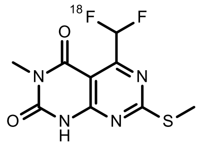

[^{18}F]19

General procedure using 6-methyl-2-methylsulfanyl-8H-pyrimido[4,5-d]pyrimidine-5,7-dione (4.5 mg, 20 µmol) yielded to 43 ± 3 % as RCY (ndc, on crude product) of the title compound (see Table S19 and Figures S44 and S45 for radio LC of the labelled compound and LC analysis of the reference).

Table S19 : Radiochemical yield of [^{18}F]19

Reaction	Radio-TLC purity (%)	Radio-LC purity (%)	Radiochemical Yield (%)
**1**	76	60	46
**2**	70	60	42
**3**	67	60	40
**Radiochemical Yield + Deviation (%)**			43 ± 3

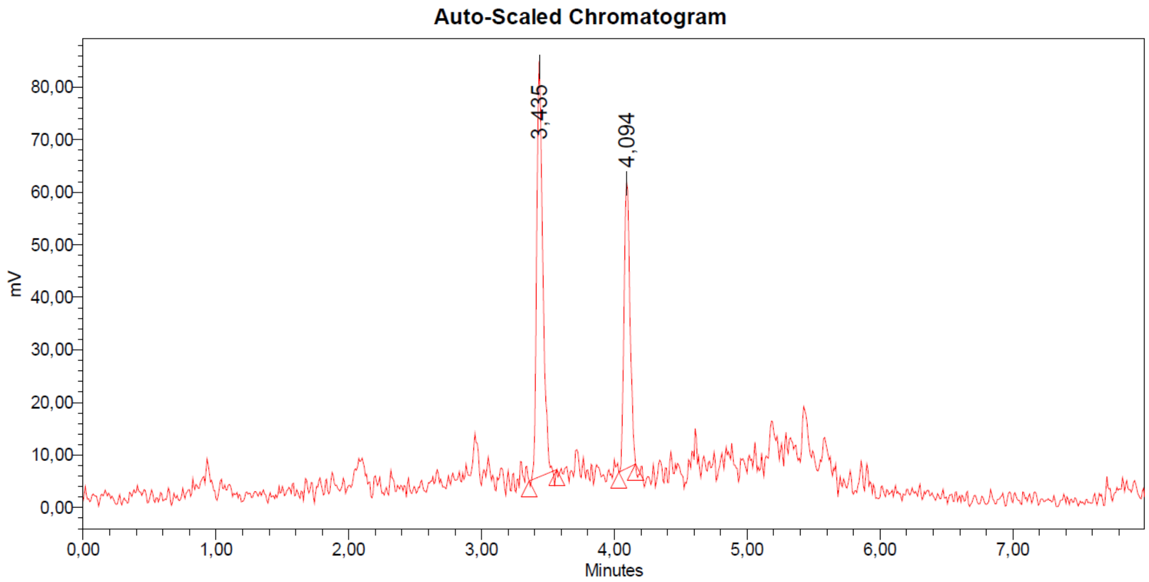

Figure S44 : UPLC radio-chromatogram of [^{18}F]19

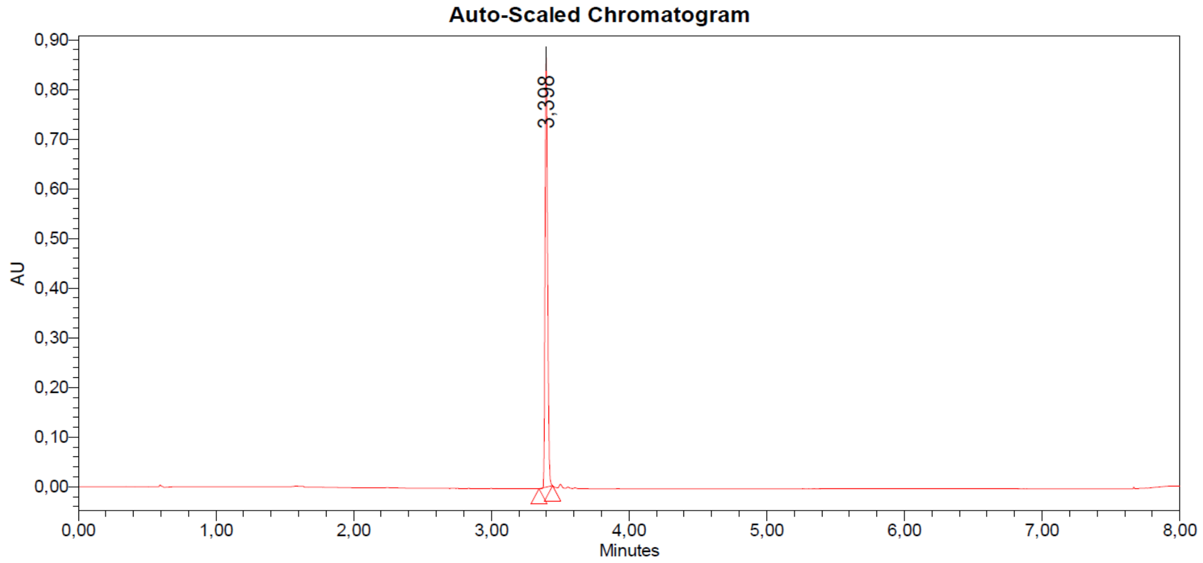

Figure S45 : UPLC UV-chromatogram of an authentic reference 19

8-(difluoromethyl)-1,3,7-trimethyl-purine-2,6-dione/ CHF^{18}F-caffeine [^{18}F]20

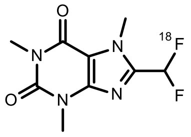

[^{18}F]20

General procedure using caffeine (3.9 mg, 20 µmol) yielded to 51 +/- 1% as RCY (ndc, on crude product) of the title compound (see Table S20 and Figures S46 and S47 for radio LC of the labelled compound and LC analysis of the reference).

Table S20 : Radiochemical yield of [^{18}F]20

Reaction	Radio-TLC purity (%)	Radio-LC purity (%)	Radiochemical Yield (%)
**1**	50	100	50
**2**	50	100	50
**3**	52	100	52
**Radiochemical Yield + Deviation (%)**			51 ± 1

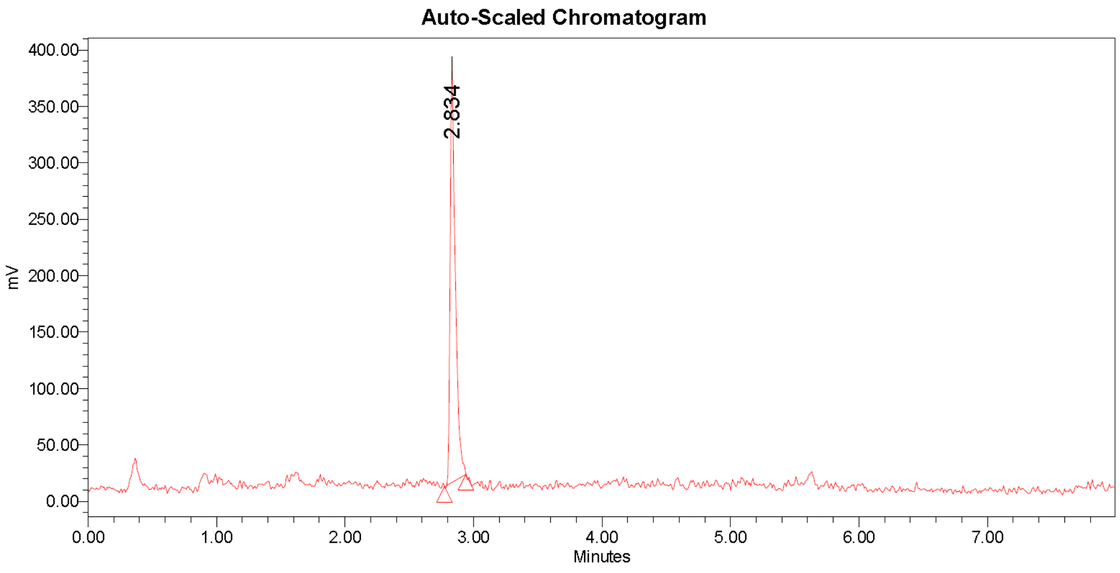

Figure S46 : UPLC radio-chromatogram of [^{18}F]20

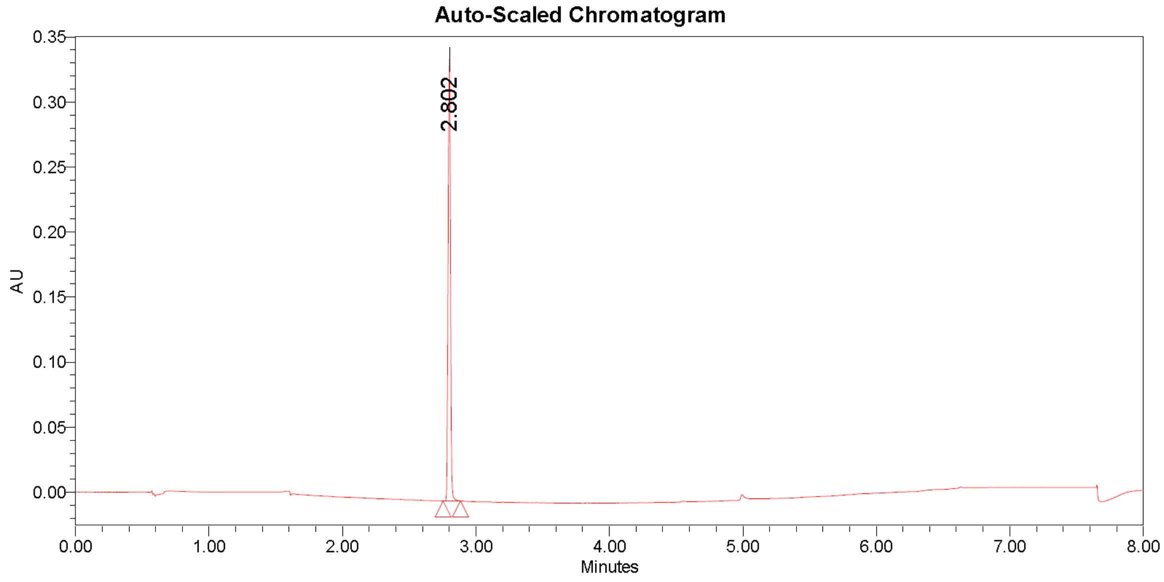

Figure S47 : UPLC UV-chromatogram of an authentic reference 20

8-(difluoromethyl)-1,3-dimethyl-7H-purine-2,6-dione / CHF^{18}F-theophylline [^{18}F]21

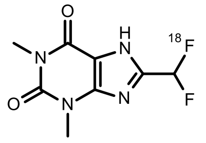

[^{18}F]21

General procedure using theophylline (3.6 mg, 20 µmol) yielded to 42 ± 6% as RCY (ndc, on crude product) of the title compound (see Table S21 and Figures S48 and S49 for radio LC of the labelled compound and LC analysis of the reference).

Table S21 : Radiochemical yield of [^{18}F]21

Reaction	Radio-TLC purity (%)	Radio-LC purity (%)	Radiochemical Yield (%)
**1**	51	96	48
**2**	43	83	36
**3**	57	74	42
**Radiochemical Yield + Deviation (%)**			42 ± 6

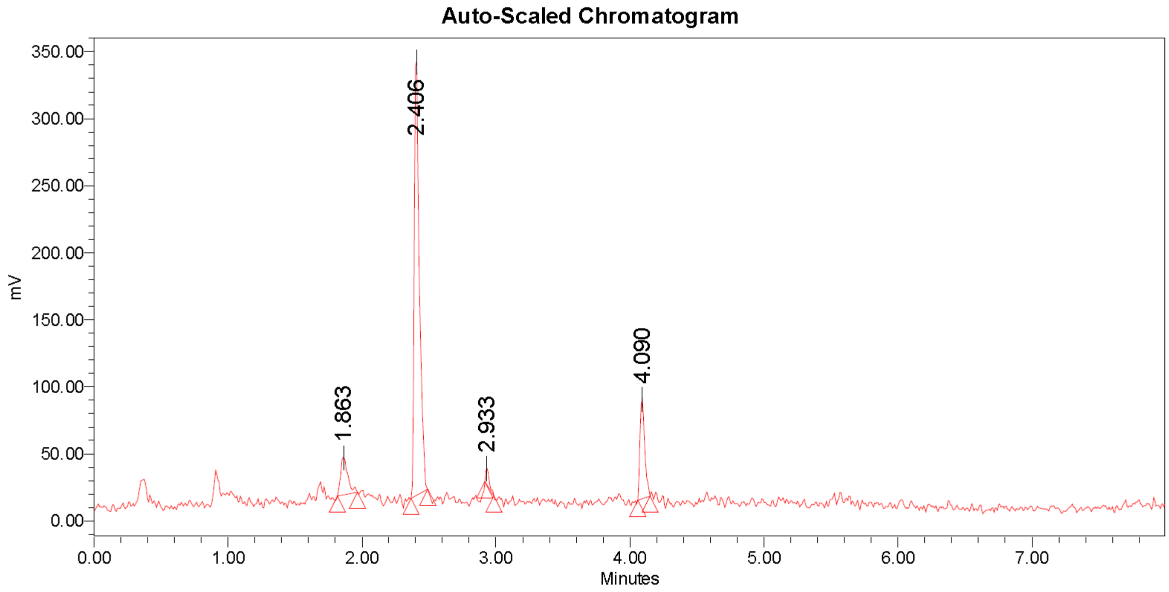

Figure S48 : UPLC radio-chromatogram of [^{18}F]21

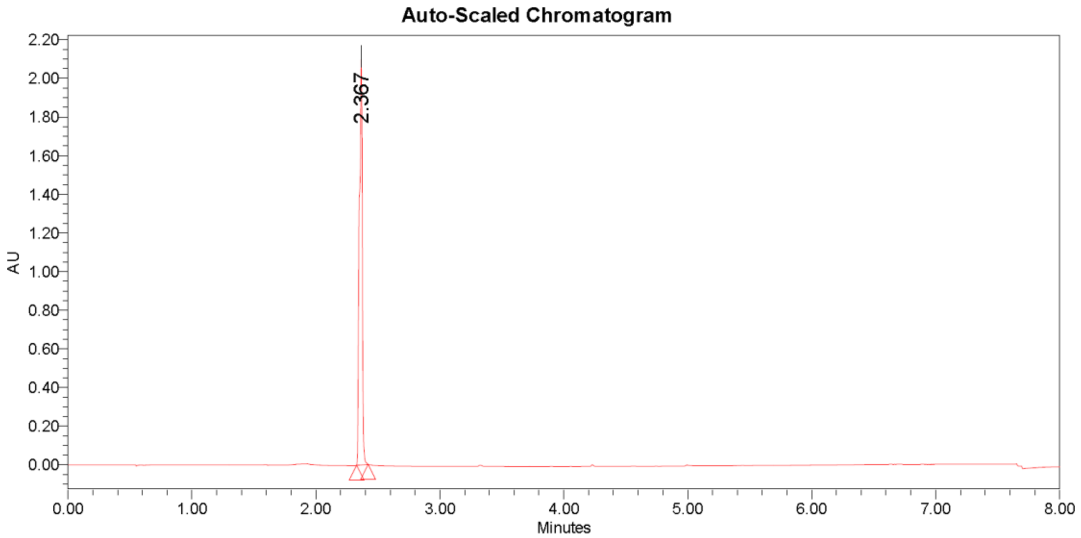

Figure S49 : UPLC UV-chromatogram of an authentic reference 21

8-(difluoromethyl)-3,7-dimethyl-1-(5-oxohexyl)purine-2,6-dione / CHF^{18}F-pentoxyfilline [^{18}F]22

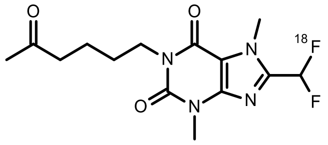

[^{18}F]22

General procedure using pentoxyfilline (5.6 mg, 20 µmol) yielded to 30 ± 5 % as RCY (ndc, on crude product) of the title compound (see Table S22 and Figures S50 and S51 for radio LC of the labelled compound and LC analysis of the reference).

Table S22 : Radiochemical yield of [^{18}F]22

Reaction	Radio-TLC purity (%)	Radio-LC purity (%)	Radiochemical Yield (%)
**1**	34	100	34
**2**	43	70	31
**3**	30	85	25
**Radiochemical Yield + Deviation (%)**			30 ± 5

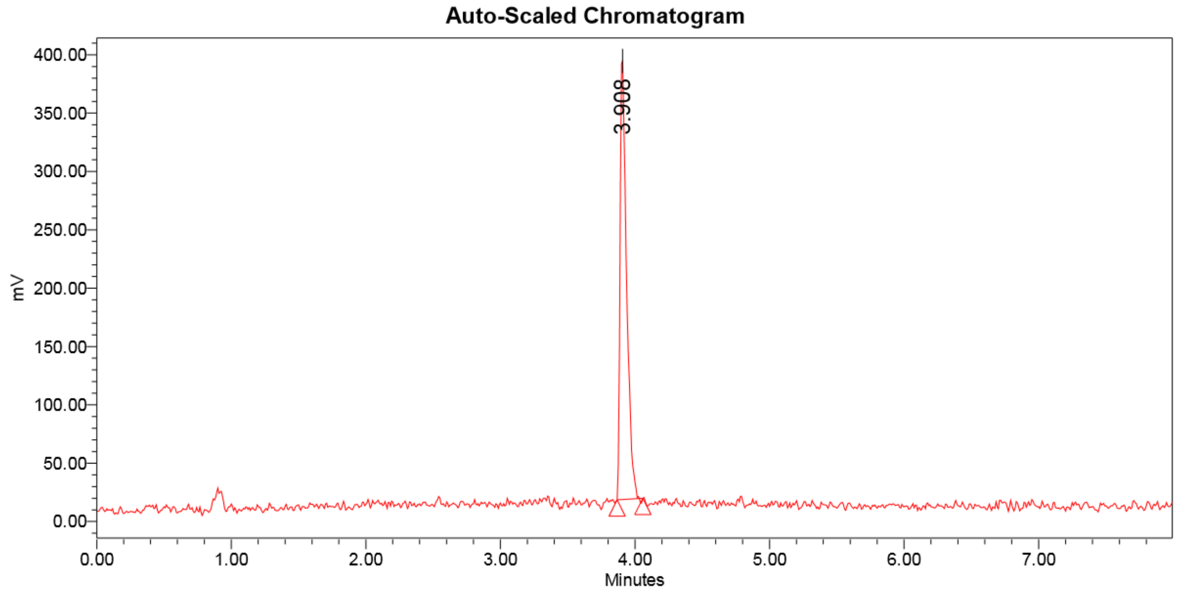

Figure S50: UPLC radio-chromatogram of [^{18}F]22

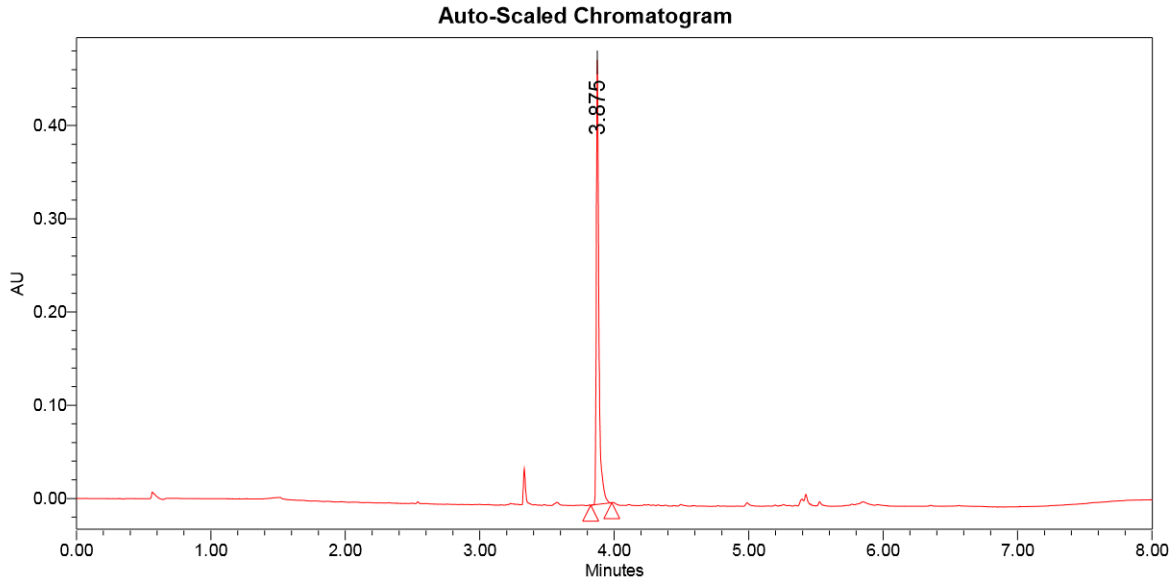

Figure S51 : UPLC UV-chromatogram of an authentic reference 22

5-(difluoromethyl)-1,3-dimethyl-pyrimidine-2,4-dione / CHF^{18}F-dimethyl-uracil [^{18}F]23

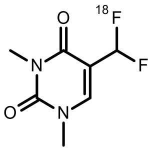

[^{18}F]23

General procedure using dimethyl-uracil (2.8 mg, 20 µmol) yielded to 54 ± 1% as RCY (ndc, on crude product) of the title compound (see Table S23 and Figures S52 and S53 for radio LC of the labelled compound and LC analysis of the reference).

Table S23 : Radiochemical yield of [^{18}F]23

Reaction	Radio-TLC purity (%)	Radio-LC purity (%)	Radiochemical Yield (%)
**1**	78	70	55
**2**	70	75	53
**3**	75	70	53
**Radiochemical Yield + Deviation (%)**			54 ± 1

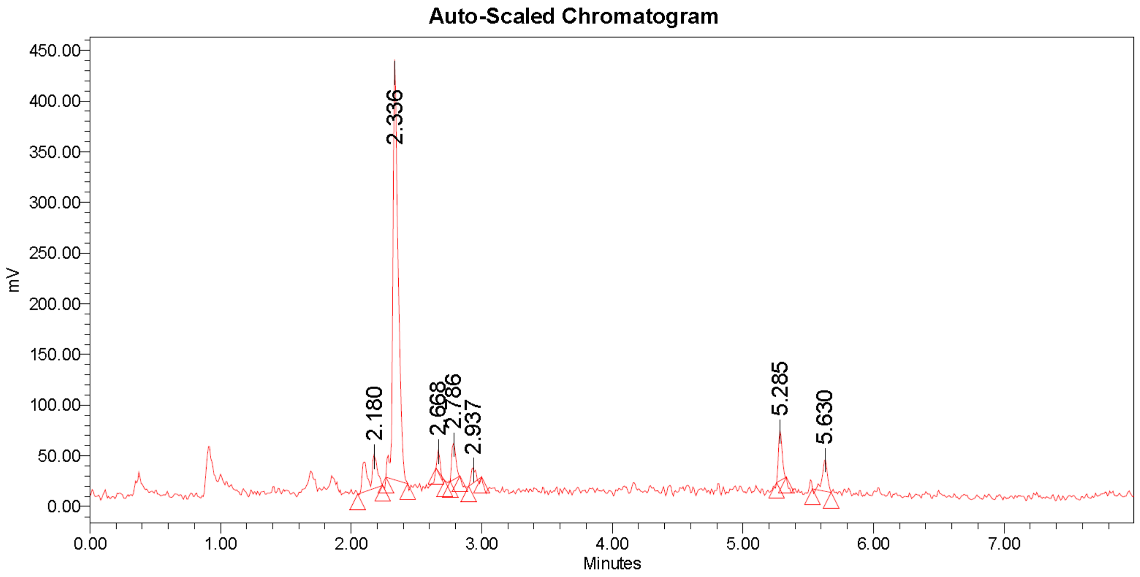

Figure S52 : UPLC radio-chromatogram of [^{18}F]23

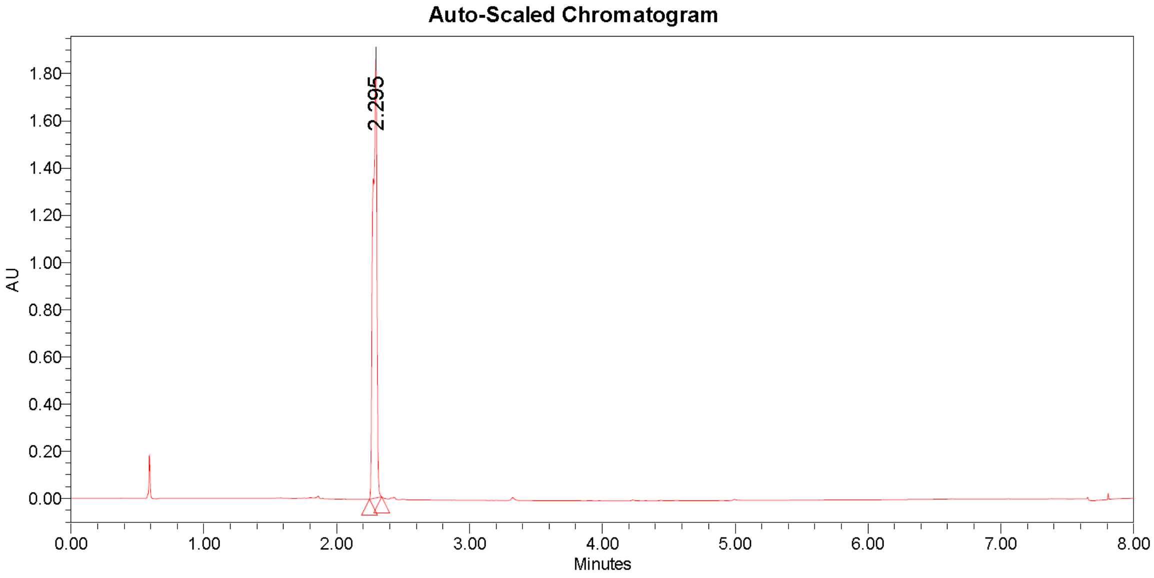

Figure S53 : UPLC UV-chromatogram of an authentic reference 23

6-amino-5-(difluoromethyl)-1H-pyrimidin-2-one [^{18}F]24a and 6-amino-4-(difluoromethyl)-1H-pyrimidin-2-one [^{18}F]24b/ CHF^{18}F -cytosine [^{18}F]24

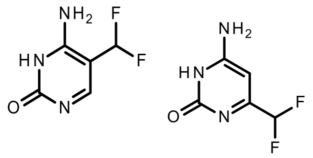

[^{18}F]24a [^{18}F]24b

General procedure using cytosine (3.2 mg, 20 µmol) yielded to 60 ± 2 % as RCY (ndc, on crude product) of the title compounds. The two isomers were not distincted in UPLC analyses, the ratio was then not determined. (see Table S24 and Figures S54 and S55 for radio LC of the labelled compound and LC analysis of the reference).

Table S24 : Radiochemical yield of [^{18}F]24

Reaction	Radio-TLC purity (%)	Radio-LC purity (%)	Radiochemical Yield (%)
**1**	58	100	58
**2**	57	100	57
**3**	62	100	62
**Radiochemical Yield + Deviation (%)**			60 ± 2

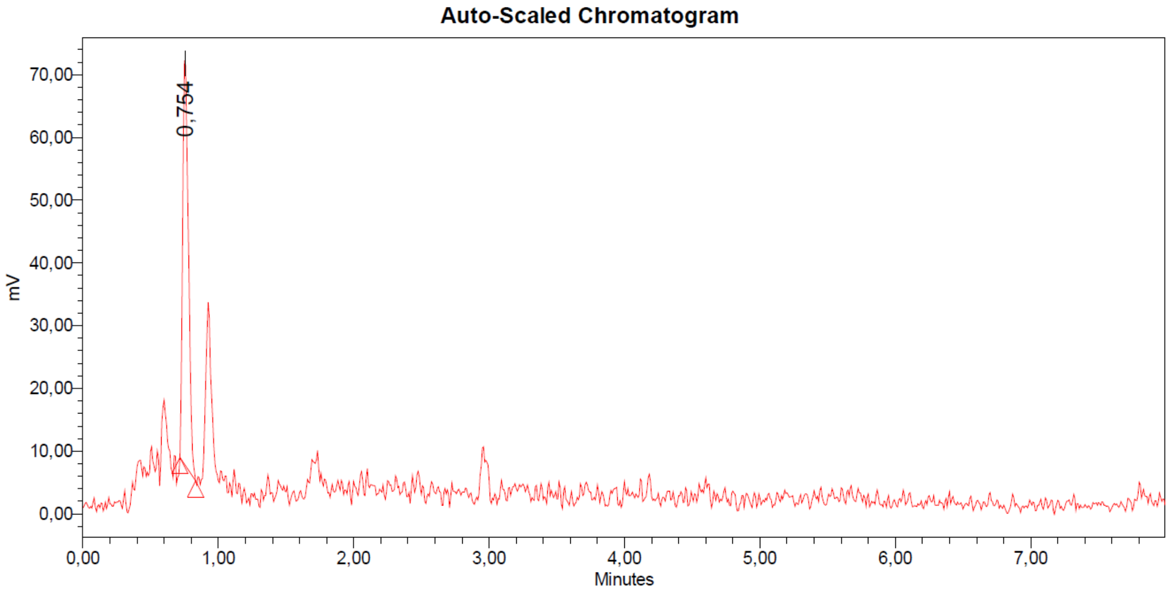

Figure S54 : UPLC radio-chromatogram of [^{18}F]24

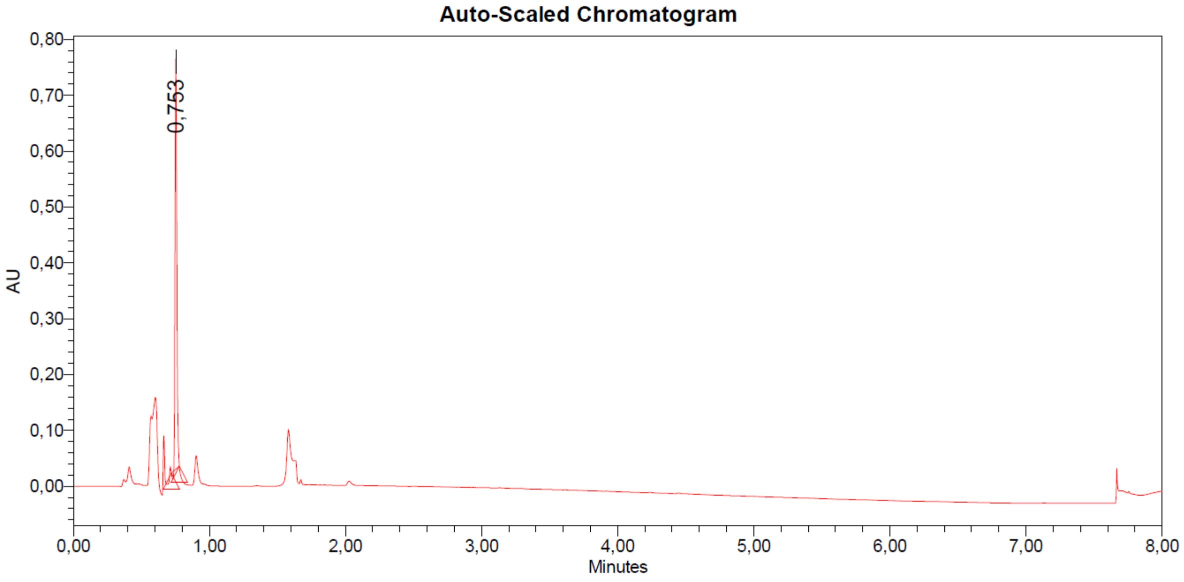

Figure S55 : UPLC UV-chromatogram of an authentic reference 24a and 24b

8-(difluoromethyl)-9H-purin-6-amine / CHF^{18}F-adenine [^{18}F]25

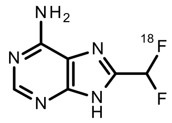

[^{18}F]25

General procedure using adenine (2.7 mg, 20 µmol) yielded to 65 ± 6 % as RCY (ndc, on crude product) of the title compound (see Table S25 and Figures S56 and S57 for radio LC of the labelled compound and LC analysis of the reference).

Table S25 : Radiochemical yield of [^{18}F]25

Reaction	Radio-TLC purity (%)	Radio-LC purity (%)	Radiochemical Yield (%)
**1**	76	94	71
**2**	65	100	65
**3**	59	100	59
**Radiochemical Yield + Deviation (%)**			65 ± 6

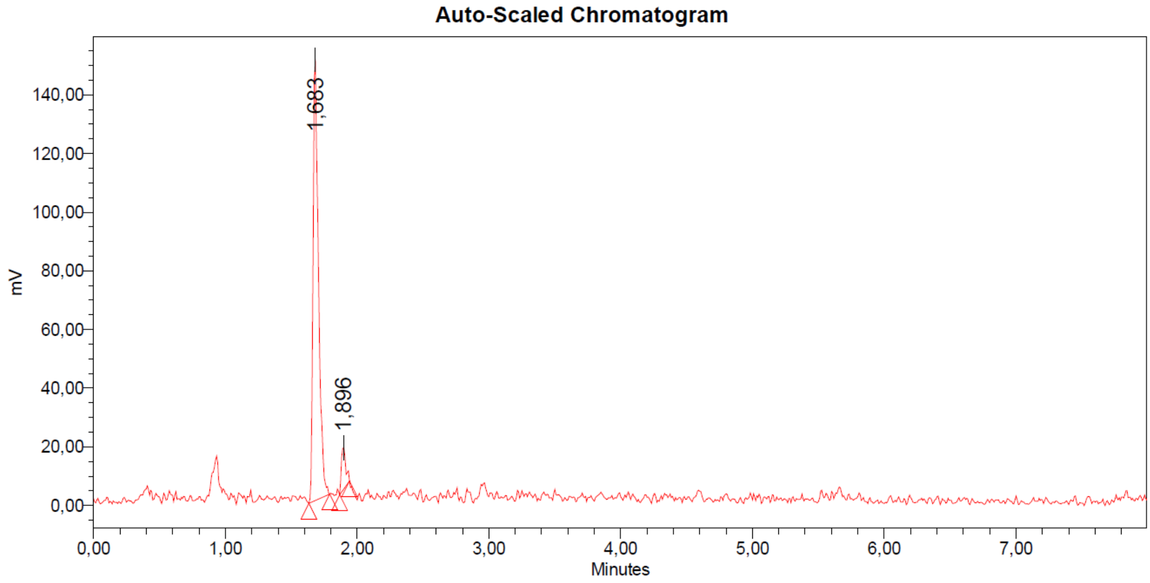

Figure S56 : UPLC radio-chromatogram of [^{18}F]25

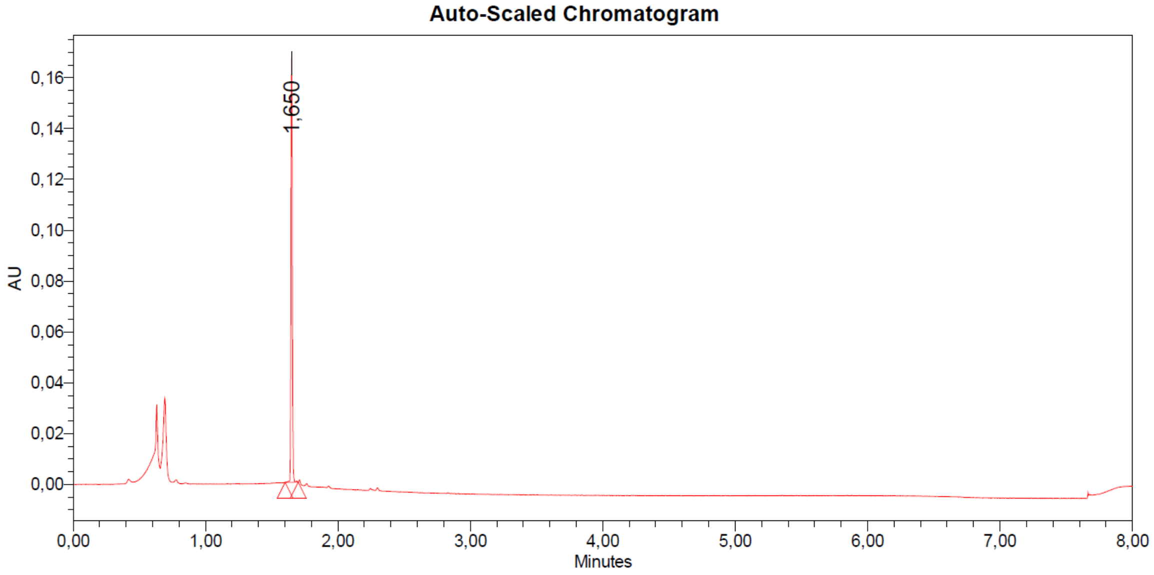

Figure S57 : UPLC UV-chromatogram of an authentic reference 25

(1S)-5-(difluoromethyl)-1-[(3R,4S,5R)-3,4-dihydroxy-5-(hydroxymethyl)tetrahydrofuran-2-yl]pyrimidine-2,4-dione / CHF^{18}F-uridine [^{18}F]26

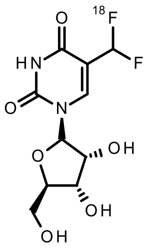

[^18^F]26

General procedure using uridine (4.8 mg, 20 µmol) yielded to 71 ± 4 % as RCY (ndc, on crude product) of the title compound (see Table S26 and Figures S58 and S59 for radio LC of the labelled compound and LC analysis of the reference).

Table S26 : Radiochemical yield of [^{18}F]26

Reaction	Radio-TLC purity (%)	Radio-LC purity (%)	Radiochemical Yield (%)
**1**	75	100	75
**2**	76	100	76
**3**	67	100	67
**Radiochemical Yield + Deviation (%)**			71 ± 4

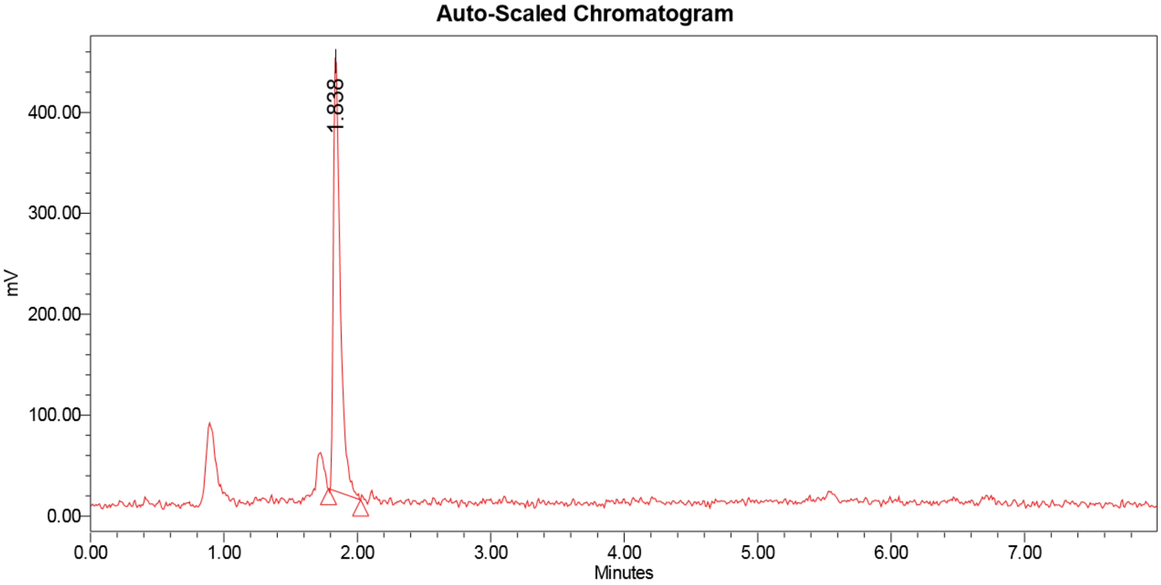

Figure S58 : UPLC radio-chromatogram of [^{18}F]26

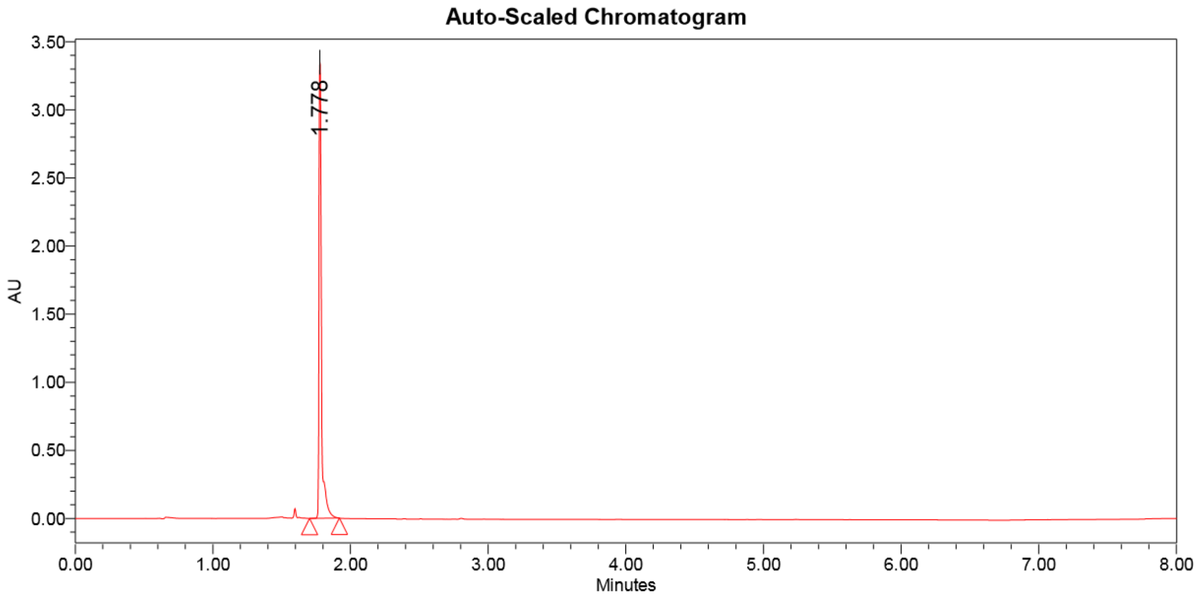

Figure S59 : UPLC UV-chromatogram of an authentic reference 26

(1S)-4-amino-5-(difluoromethyl)-1-[(3R,4S,5R)-3,4-dihydroxy-5-(hydroxymethyl)tetrahydrofuran-2-yl]pyrimidin-2-one / CHF^{18}F-cytidine [^{18}F]27

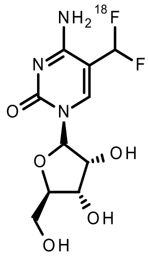

[^{18}F]27

General procedure using cytidine (4.8 mg, 20 µmol) yielded to 65 ± 2 % as RCY (ndc, on crude product) of the title compound (see Table S27 and Figures S60 and S61 for radio LC of the labelled compound and LC analysis of the reference).

Table S27 : Radiochemical yield of [^{18}F]27

Reaction	Radio-TLC purity (%)	Radio-LC purity (%)	Radiochemical Yield (%)
**1**	64	100	64
**2**	67	100	67
**3**	66	100	66
**Radiochemical Yield + Deviation (%)**			65 ± 2

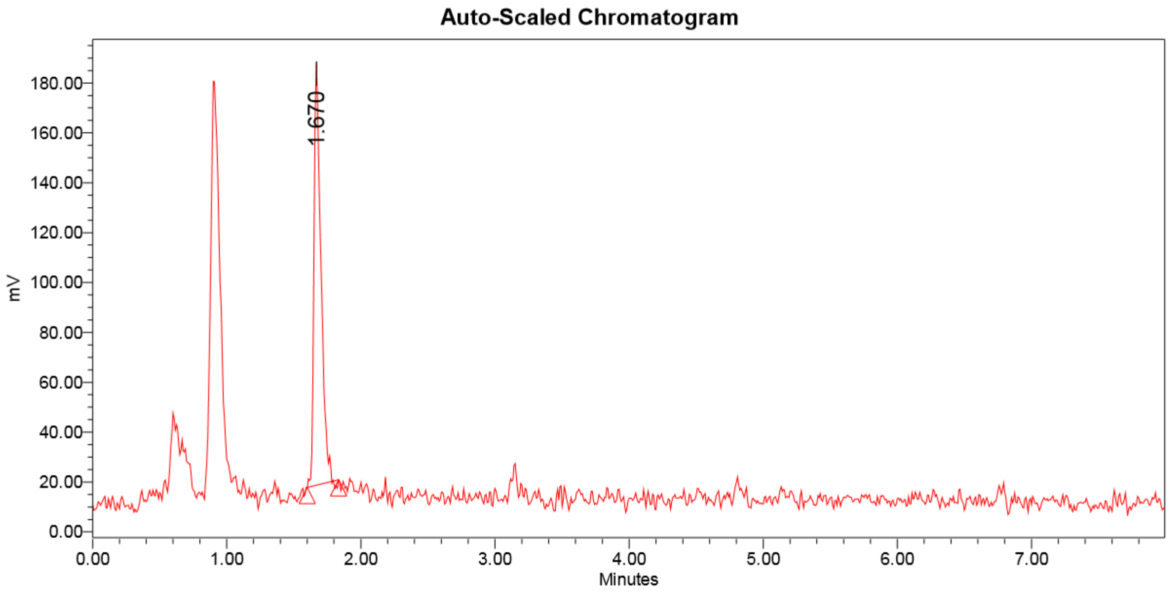

Figure S60 : UPLC radio-chromatogram of [^{18}F]27

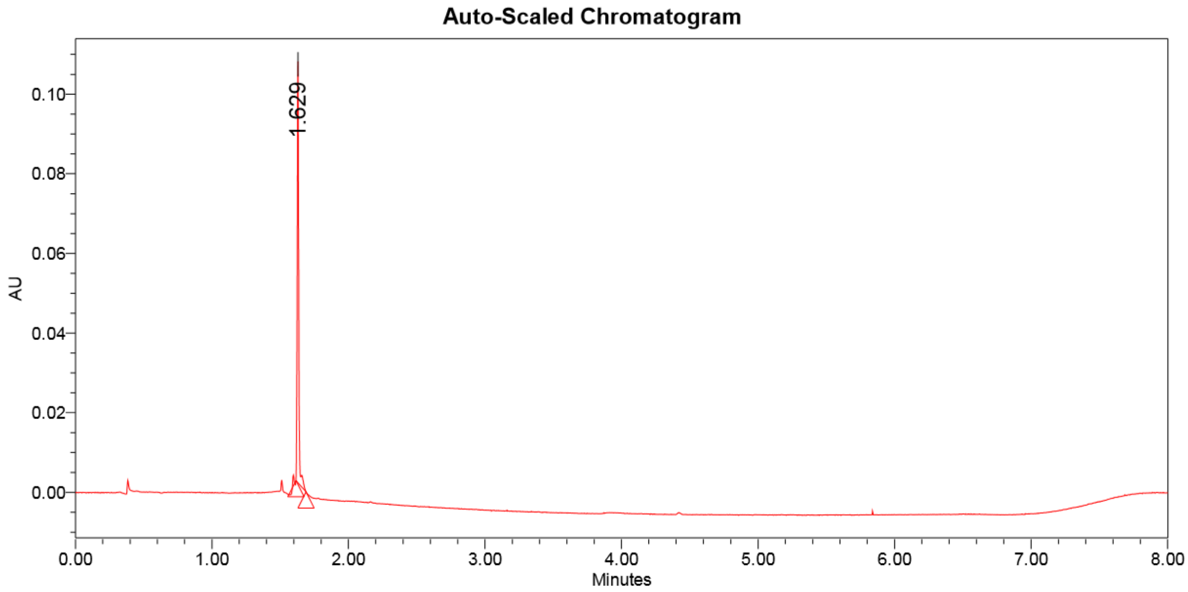

Figure S61 : UPLC UV-chromatogram of an authentic reference 27

(3R,4S,5R)-2-[(9S)-6-amino-8-(difluoromethyl)purin-9-yl]-5-(hydroxymethyl) tetrahydrofuran-3,4-diol / CHF^{18}F-Adenosine [^{18}F]28

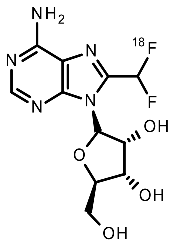

[18F]28

General procedure using adenosine (5.3 mg, 20 µmol) yielded to 59 ± 5% as RCY (ndc, on crude product) of the title compound (see Table S28 and Figures S62 and S63 for radio LC of the labelled compound and LC analysis of the reference).

Table S28 : Radiochemical yield of [18F]28

Reaction	Radio-TLC purity (%)	Radio-LC purity (%)	Radiochemical Yield (%)
**1**	64	97	62
**2**	59	100	59
**3**	55	100	55
**Radiochemical Yield + Deviation (%)**			58 ± 4

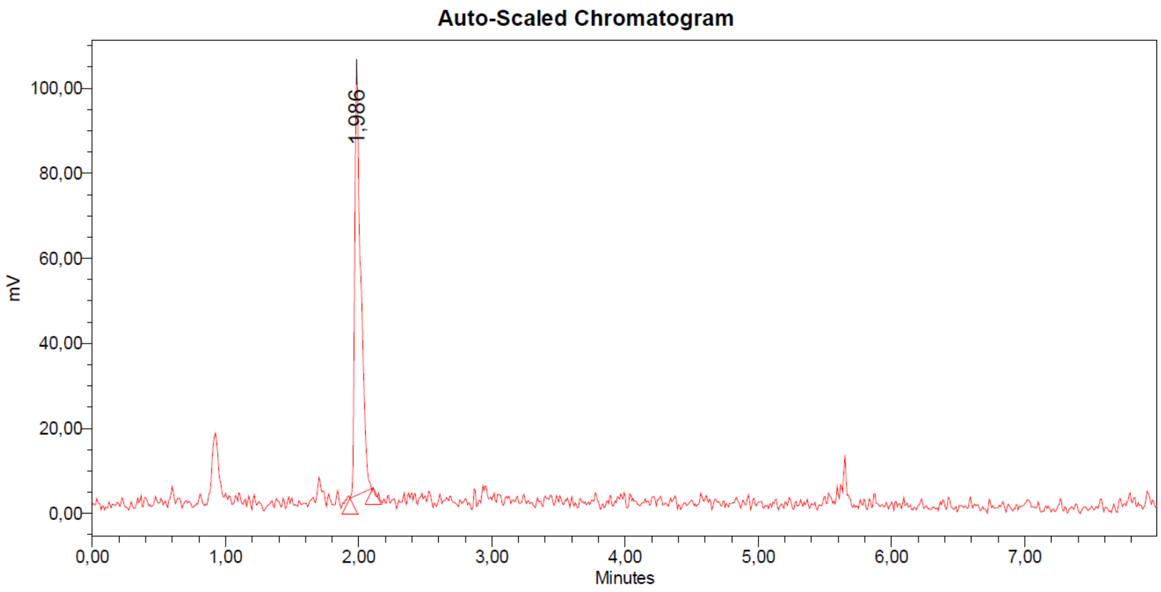

Figure S62 : UPLC radio-chromatogram of [18F]28

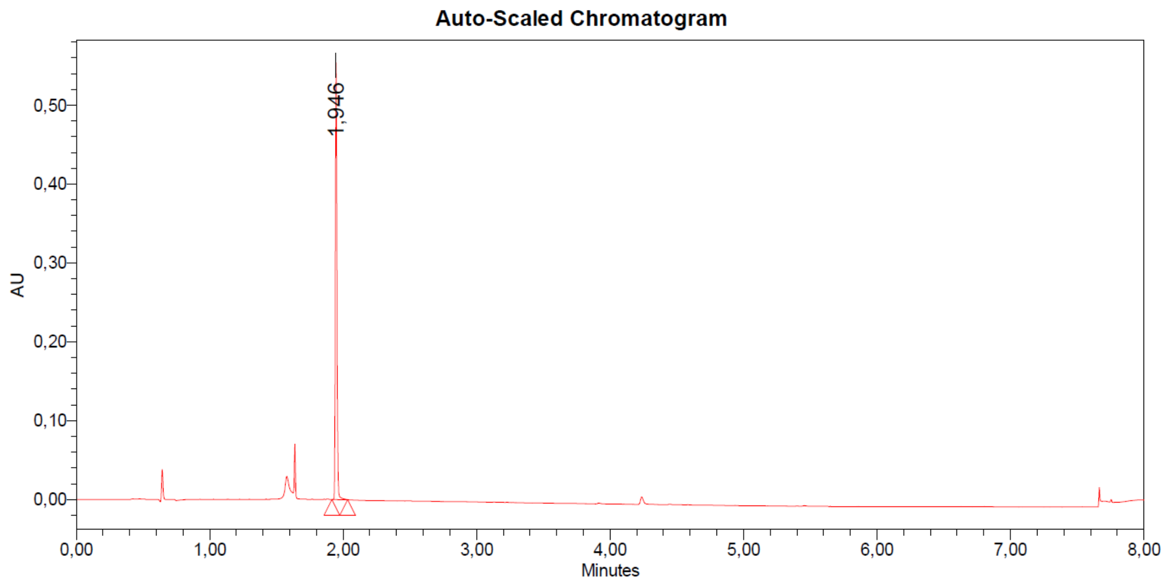

Figure S63 : UPLC UV-chromatogram of an authentic reference 28

(9S)-2-amino-8-(difluoromethyl)-9-[(3R,4S,5R)-3,4-dihydroxy-5-(hydroxymethyl) tetrahydrofuran-2-yl]-1H-purin-6-one / CHF^{18}F-guanosine [^{18}F]29

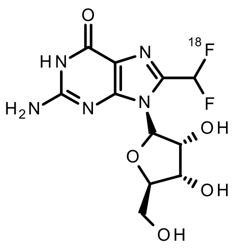

[18F]29

General procedure using guanosine (5.7 mg, 20 µmol) yielded to 62 ± 6% as RCY (ndc, on crude product) of the title compound (see Table S29 and Figures S64 and S65 for radio LC of the labelled compound and LC analysis of the reference).

Table S29 : Radiochemical yield of [^{18}F]29

Reaction	Radio-TLC purity (%)	Radio-LC purity (%)	Radiochemical Yield (%)
**1**	61	100	61
**2**	68	100	68
**3**	56	100	56
**Radiochemical Yield + Deviation (%)**			62 ± 6

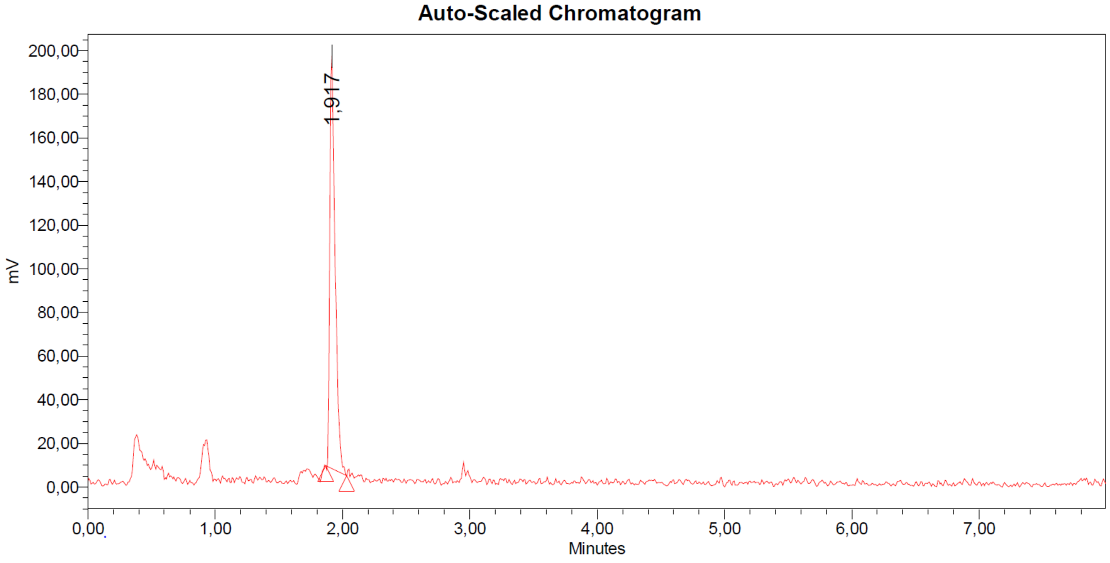

Figure S64 : UPLC radio-chromatogram of [^{18}F]29

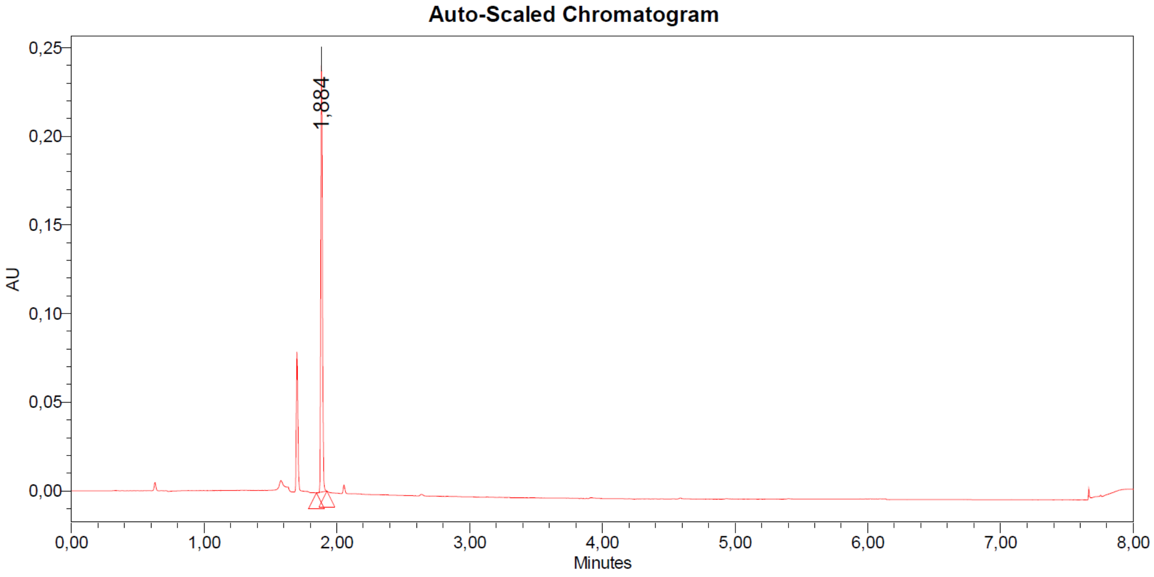

Figure S65 : UPLC UV-chromatogram of an authentic reference 29

4-chloro-2-(difluoromethyl)-N-(4,5-dihydro-1H-imidazol-2-yl)-6-methoxy-pyrimidin-5-amine / CHF^{18}F-moxonidine analogue [^{18}F]30

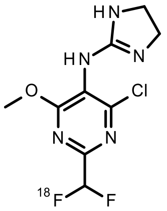

[^{18}F]30

General procedure using moxonidine analogue (4.5 mg, 20 µmol) yielded to 65 ± % 4 as RCY (ndc, on crude product) of the title compound (see Table S30 and Figures S66 and S67 for radio LC of the labelled compound and LC analysis of the reference).

Table S30 : Radiochemical yield of [^{18}F]30

Reaction	Radio-TLC purity (%)	Radio-LC purity (%)	Radiochemical Yield (%)
**1**	63	100	63
**2**	69	100	69
**3**	61	100	61
**Radiochemical Yield + Deviation (%)**			65 ± 4

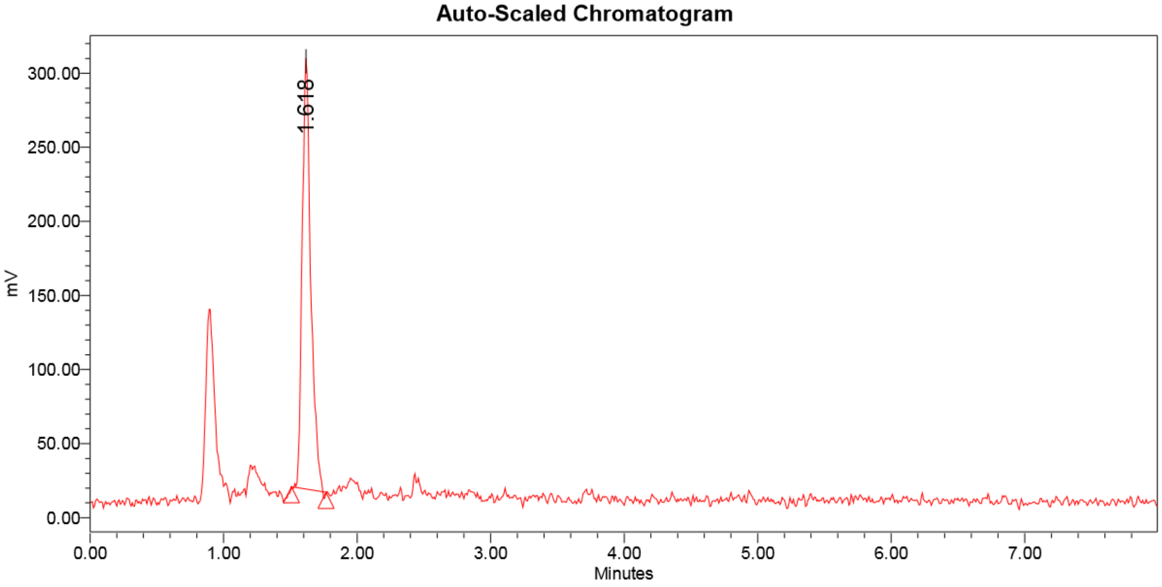

Figure S66 : UPLC radio-chromatogram of [^{18}F]30

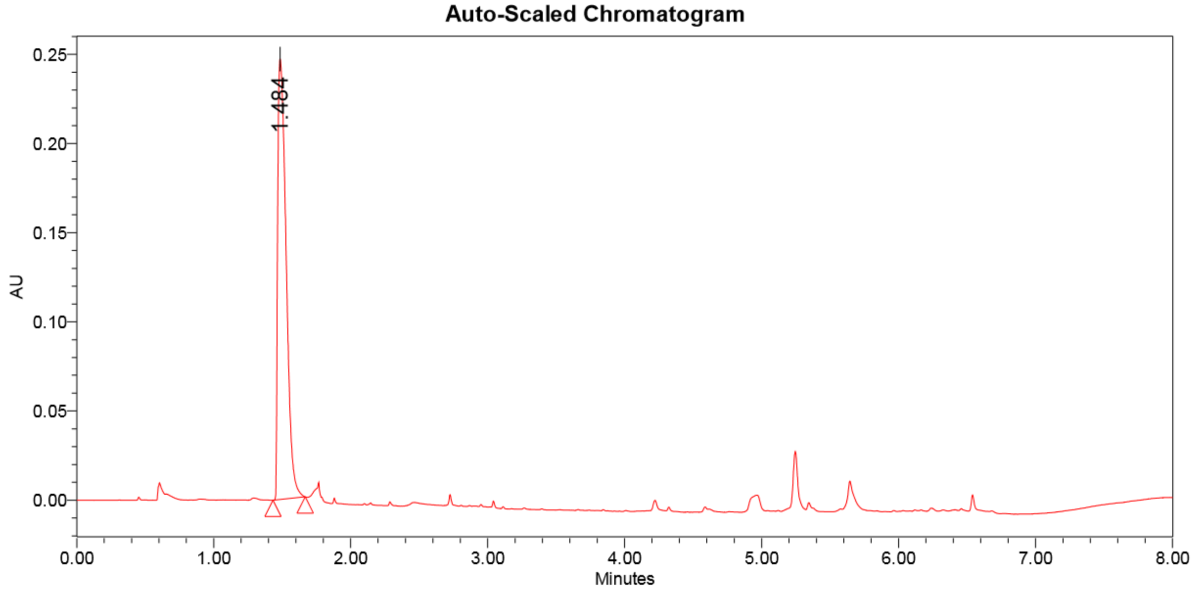

Figure S67 : UPLC UV-chromatogram of an authentic reference 30

(4R)-1-[[2-(difluoromethyl)-4-pyridyl]methyl]-4-(3,4,5-trifluorophenyl)pyrrolidin-2-one [^{18}F]31a, (4R)-1-[[3-(difluoromethyl)-4-pyridyl]methyl]-4-(3,4,5-trifluorophenyl)pyrrolidin-2-one [^{18}F]31b, (4R)-4-[2-(difluoromethyl)-3,4,5-trifluoro-phenyl]-1-(4-pyridylmethyl)pyrrolidin-2-one [^{18}F]31c

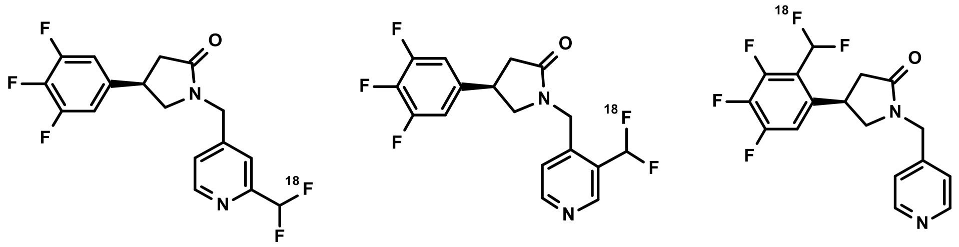

[^{18}F]31a [^{18}F]31b [^{18}F]31c

General procedure using compound **39** (6.1 mg, 20 µmol) yielded respectively to 14.8%, 3.8% and 7.1% as RCYs (ndc, on crude product) of **[^{18}F]31a, [^{18}F]31b** and **[^{18}F]31c** (see Table S31 and Figures S68 to S71 for radio LC of the labelled compound and LC analysis of the reference).

Table S31 : Radiochemical yield of [^{18}F]31

Reaction	Radio-TLC purity (%)	Radio-LC purity (%)			Radiochemical Yield (%)		
	a+b+c	a	b	c	a	b	c
**1**	80	20	5	10	16	4	8
**2**	75	18	6	9	13.5	4.5	7
**3**	78	19	4	8	14.8	3.1	6.2
**Radiochemical Yield + Deviation (%)**					14.8 ± 0.3	3.8 ± 0.7	7.1 ± 0.9

Different analyses conditions were used, to ensure a better separation of the different isomers.
The new analytic gradient is disclosed in the table below : (Table S32)

Table S32 : Gradient used for SVA PET tracer compounds

Time (min)	H_2O (0.05% HCOOH) (%)	MeCN (%)	Flow (mL/min)
0	100	0	0.5
0.5	100	0	0.5
0.6	70	30	0.5
10	65	35	0.5
10.1	0	100	0.5
11	0	100	0.5
12	100	0	0.5
13	100	0	0.5

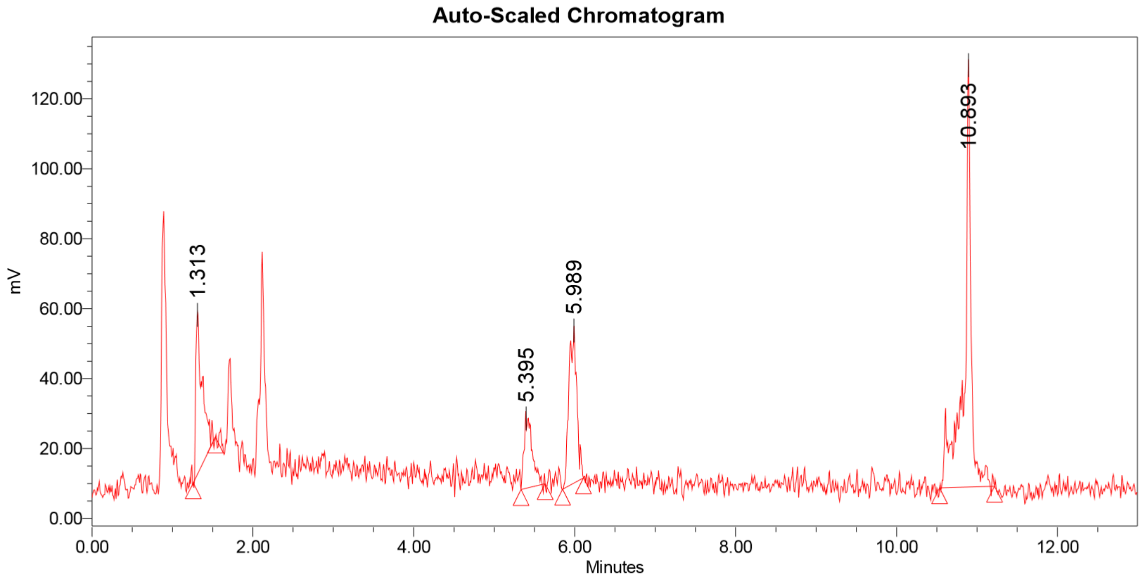

Figure S68 : UPLC radio-chromatogram of [^{18}F]31

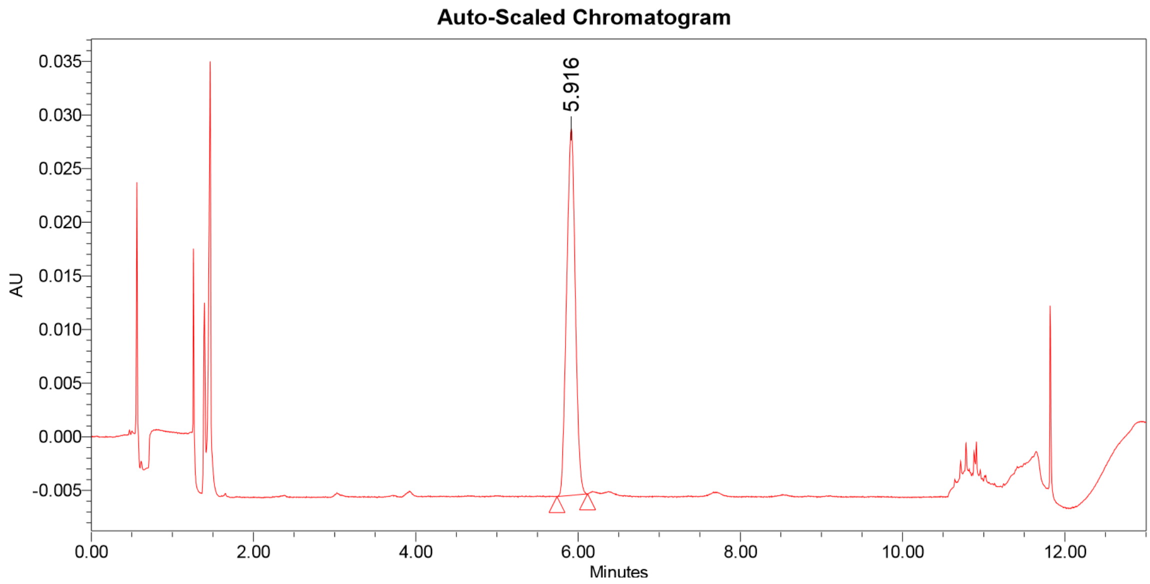

Figure S69 : UPLC UV-chromatogram of an authentic reference 31a

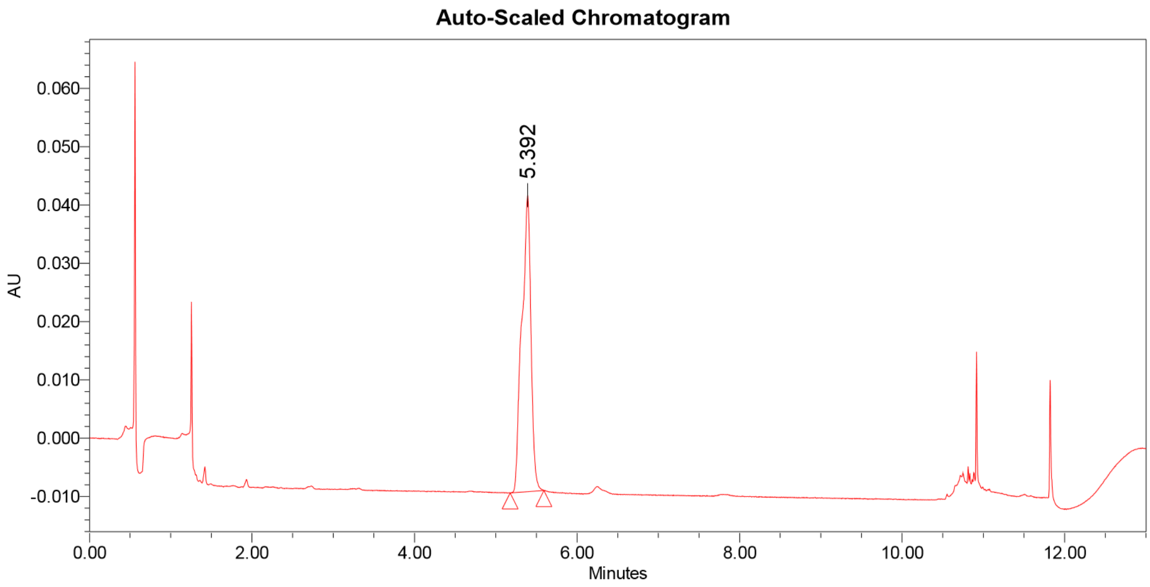

Figure S70 : UPLC UV-chromatogram of an authentic reference 31b

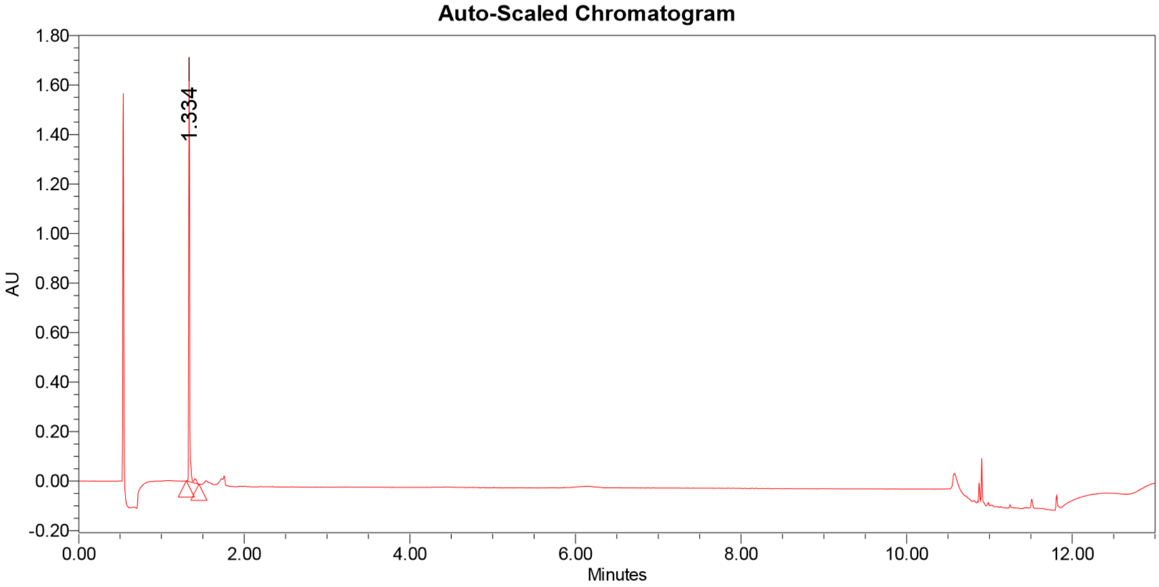

Figure S71 : UPLC UV-chromatogram of an authentic reference 31c

Then a Semi-Prep HPLC, using a mixture of MeCN/H_2O (1/1) as eluent was performed to recover **[^{18}F]31a** and **[^{18}F]31b** with respective isolated RCYs dc of 4.2 ± 0.3 % and 1.5 ± 0.1 % (n=3). Starting from 7 mCi, 0.1 mCi of **[^{18}F]31b** was isolated after 20 minutes (photoredox reaction + purification).

3. Comparison batch fluorine-19/flow fluorine-18 conditions

Some of the substrates were also tested in non-radioactive chemistry to able a comparison between non-radioactive and radioactive chemistry and put in evidence the difference of reactivity between both conditions. The general procedure and the results are reported just below.

a. General procedure for batch fluorine-19 conditions

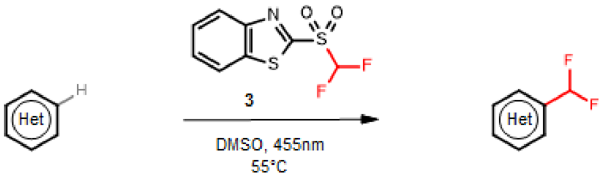

A solution of the substrate (0.10 mmol), 2-((Difluoromethyl)sulfonyl)benzo[*d*]thiazole **3** (37 mg, 0.15 mmol, 1.5 equiv.), and [Ir(ppy)$_3$] (3.3 mg, 0.005 mmol, 5 mol%) in DMSO (0.5 mL) was prepared. The vial was put over a LED (455nm, 1 W, 55°C, see equipment below, Figure S72) and stirred for 24 hours.

2-((Difluoromethyl)sulfonyl)benzo[*d*]thiazole **3** (37 mg, 0.15 mmol, 1.5 equiv.), and [Ir(ppy)$_3$] (3.3 mg, 0.005 mmol, 5 mol%) were added again to the reaction, and the crude mixture was irradiated for another 24 hours.
Yields were determined by ^{19}F NMR after 24 and 48h reaction time.

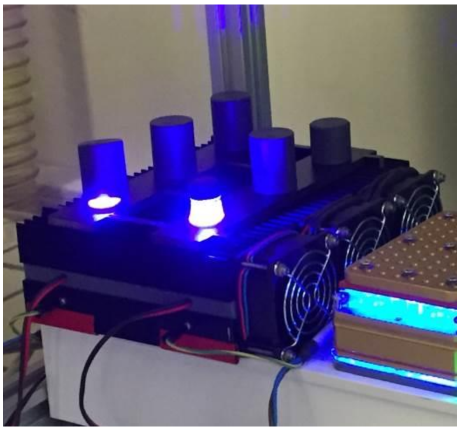

Figure S72 : 1W LED (455nm) equipment used for non-radioactive batch conditions

b. Results

The general procedure was applied to 10 compounds of the scope and the results are presented in the following table (Table S33) :

Table S33 : Comparative table for ^{19}F vs ^{18}F conditions
[1] See part 1.c.i/iii for general procedures and results, [2]See part 2h/i for general procedures and results, NA : Non available

Molecule	^{19}F-batch conditions		^{19}F-Flow Pseudo PET conditions[1]	^{18}F flow conditions[2]
	^{19}F-NMR yield 24h (%)	^{19}F-NMR yield 48h (%)	Isolated yield (%)	RCY (on crude product, %) n=3
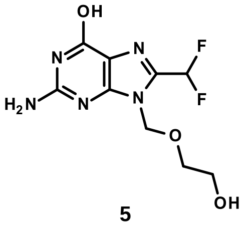 **5**	30	30	15	71 ± 5

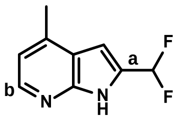<br>8	13<br>a/b : 3/1	12<br>a/b : 2/1	NA	67 ± 2<br>a/b : 90/10
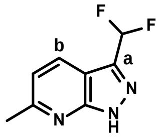<br>9	20<br>a/b :<br>30/70	45<br>a/b : 1/2	6.5<br>a/b: 2/1	74 ± 2<br>a/b : 30/70
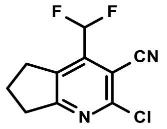<br>12	10	15	NA	43 ± 10
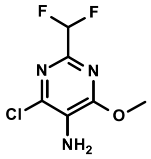<br>14	20	25	NA	57 ± 7
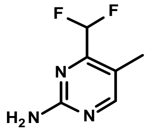<br>15	32	30	26	43 ± 3
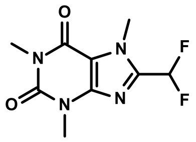<br>20	34	45	4	50 ± 1
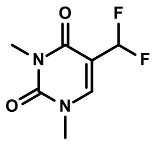<br>23	13	13	5	54 ± 1
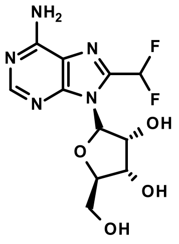<br>28	10	10	NA	59 ±5

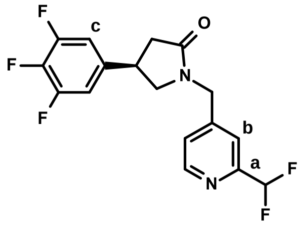<br><br>**31**	10<br>a/b/c :<br>4/1/2	15<br>a/b/c :<br>4/1/2	18<br>a/b/c : 16/1/1	25 ± 3<br>a/b/c : 4/1/2

It is interesting to note that in most cases, yields are way lower in non-radioactive chemistry (molecules **5**, **8**, **9**, **12**, **14**, **23**, **28**) while similar results are obtained with both fluorine-19 conditions.

Noteworthy, adding new portion of the difluoromethylating agent (**3**), and catalyst doesn't lead to much yield improvement suggesting the formation of a poisoning reagent preventing the reaction to continue.

Finally, the isomers ratio can be slightly different between all conditions. All these results clearly showed an improved reactivity of the reaction using fluorine-18.

4. References

[1] Y. Fujiwara, J. A. Dixon, R. A. Rodriguez, R. D. Baxter, D. D. Dixon, M. R. Collins, D. G. Blackmond, P. S. Baran, *J. Am. Chem. Soc.* **2012**, *134*, 1494.

[2] A. Sakamoto, H. Kashiwagi, K. Maruoka, *Org. Lett*. **2017**, *19*, 5126.

[3] B. A. Czeskis, *J. Label. Comp. Radiopharm.* **2004**, *47*, 699.

[4] B. Kenda, E. Jnoff, *International patent*, WO2014/012563 A1 (2014).

[5] C. Lemaire, L. Libert, X. Franci, J.-L. Genon, S. Kuci, F. Giacomelli, A. Luxen, *J. Label. Comp. Radiopharm.* **2015**, *58*, 281.

5. NMR spectra

a. ^{1}H, ^{13}C and ^{19}F NMR of 1

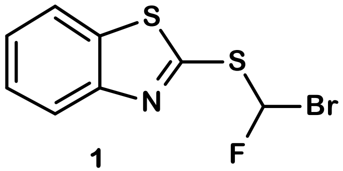
S
N
S
Br
F

1

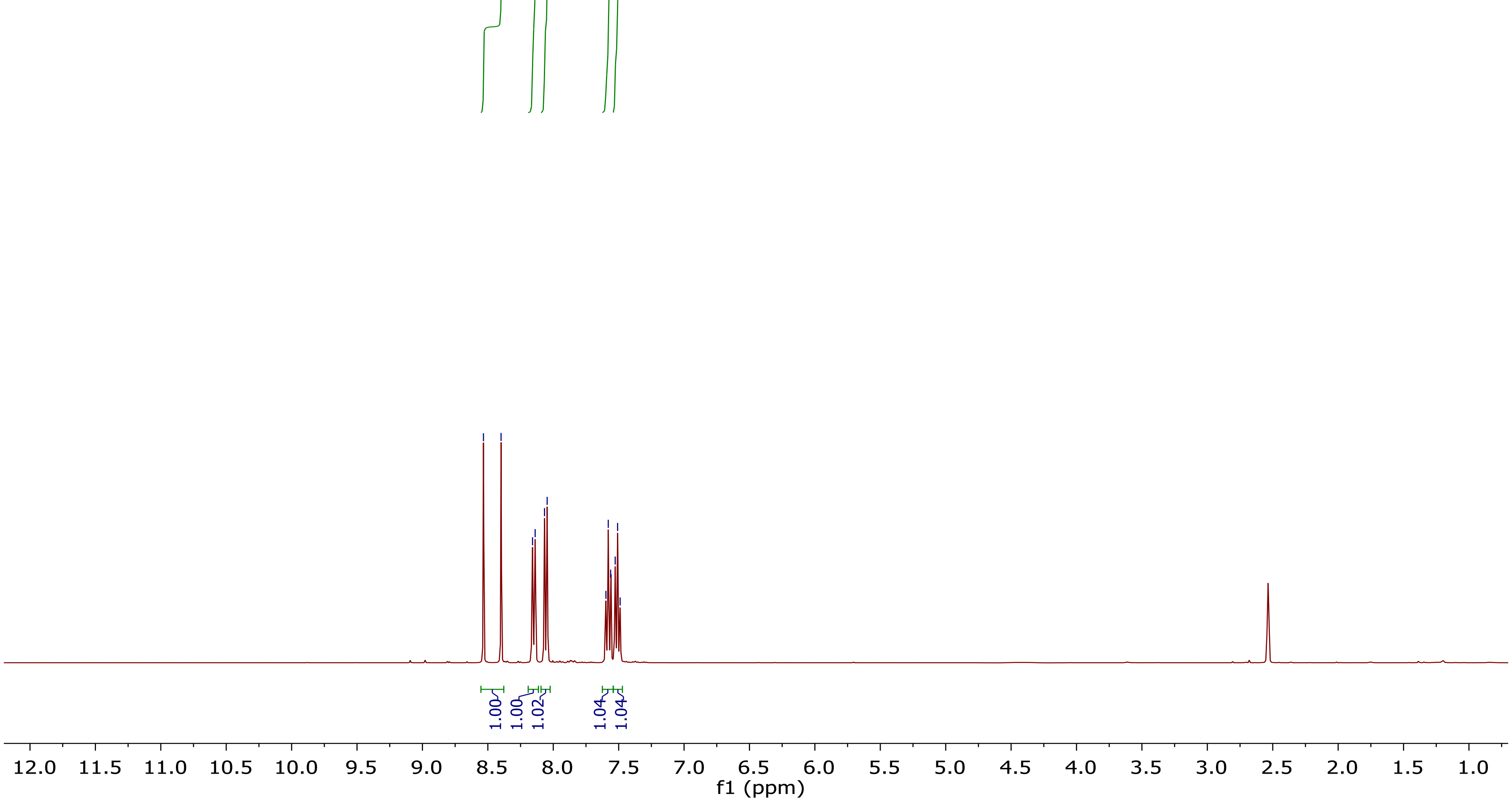
8.53
8.40
8.16
8.14
8.07
8.05
7.60
7.58
7.56
7.53
7.51
7.49
1.00
1.00
1.02
1.04
1.04
12.0 11.5 11.0 10.5 10.0 9.5 9.0 8.5 8.0 7.5 7.0 6.5 6.0 5.5 5.0 4.5 4.0 3.5 3.0 2.5 2.0 1.5 1.0
f1 (ppm)

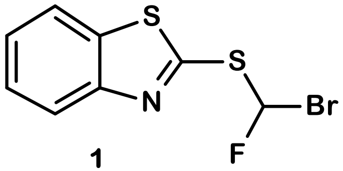
S
S
N
Br
F

1

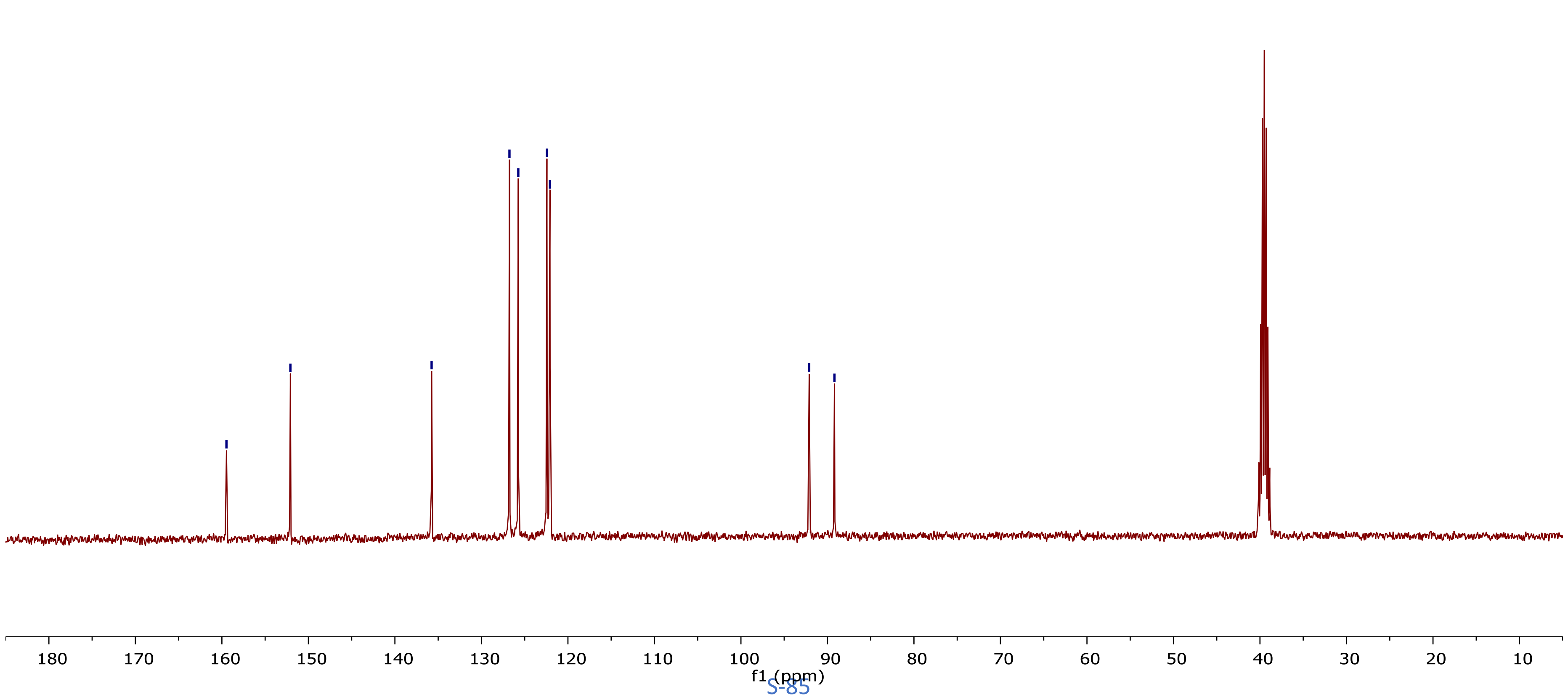
159.47
152.08
135.76
126.76
125.74
122.43
122.08
92.11
89.19
180
170
160
150
140
130
120
110
100
90
80
70
60
50
40
30
20
10
f1 (ppm)

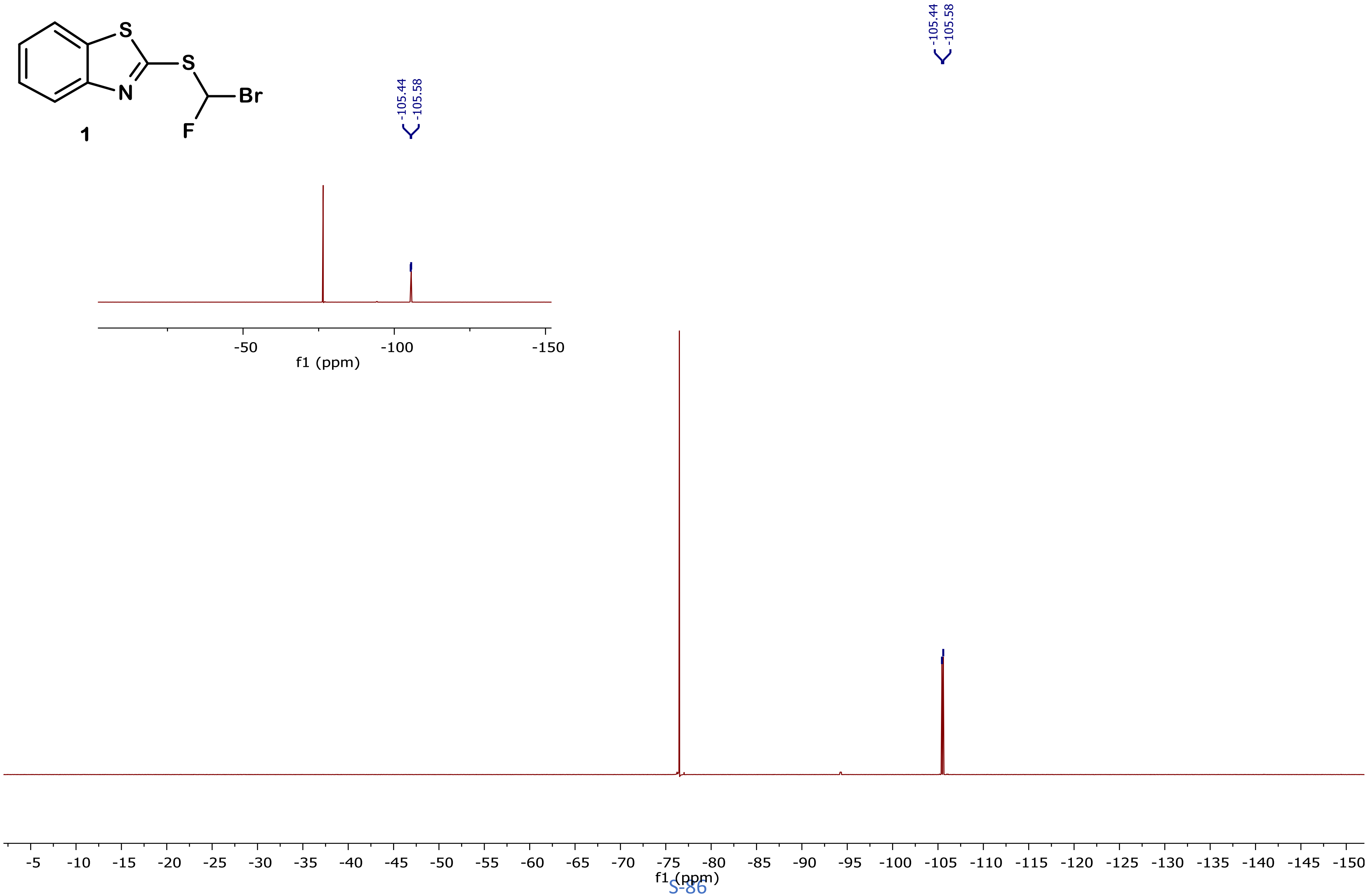
S
N
S
Br
F
1
-105.44
-105.58
-50
-100
-150
f1 (ppm)
-105.44
-105.58
-5 -10 -15 -20 -25 -30 -35 -40 -45 -50 -55 -60 -65 -70 -75 -80 -85 -90 -95 -100 -105 -110 -115 -120 -125 -130 -135 -140 -145 -150
f1 (ppm)

b. ^{1}H, ^{13}C and ^{19}F NMR of 2

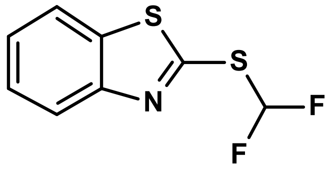

2

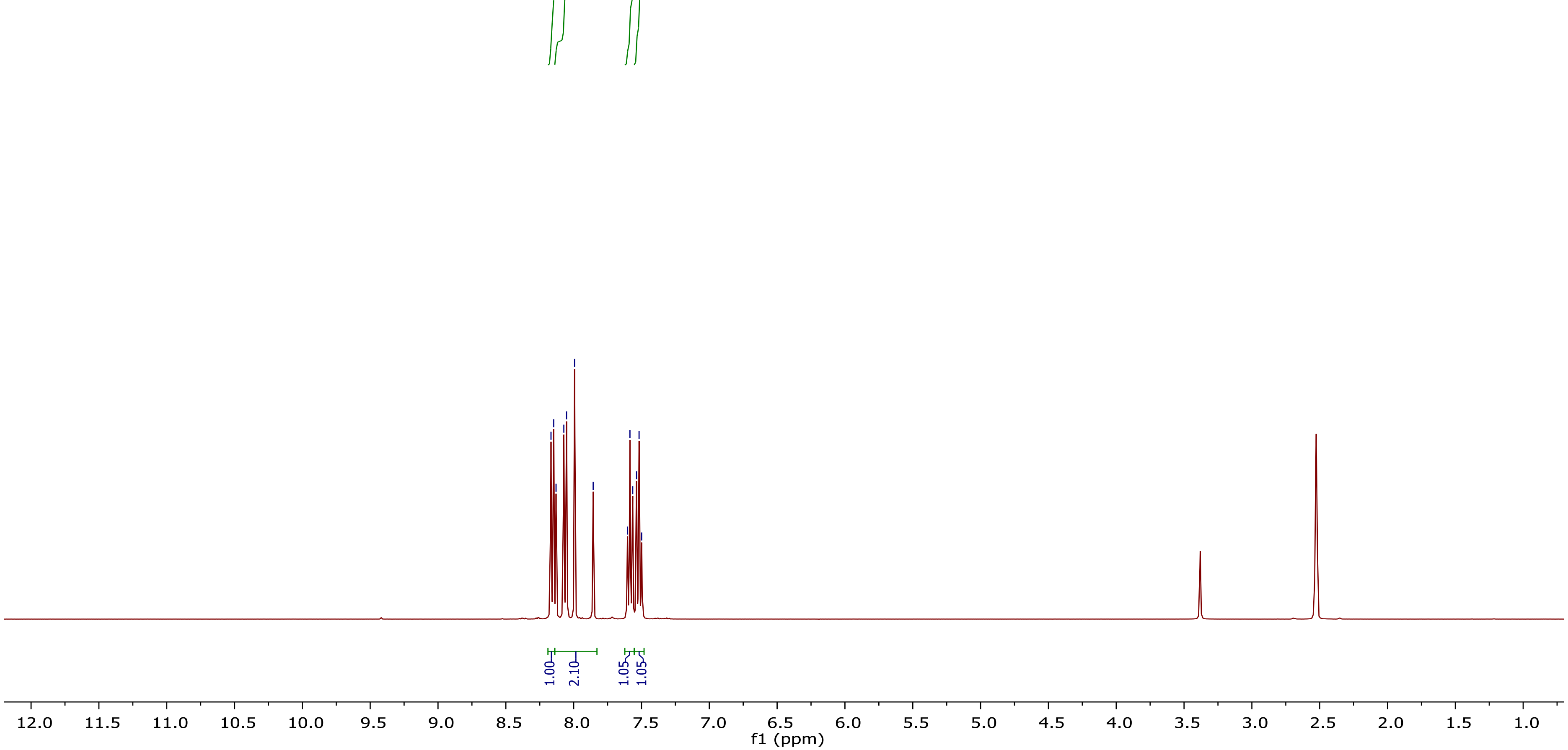
8.17
8.15
8.13
8.07
8.05
7.99
7.86
7.60
7.58
7.56
7.54
7.52
7.50
1.00
2.10
1.05
1.05
f1 (ppm)

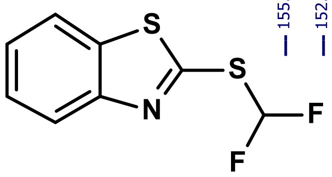

2

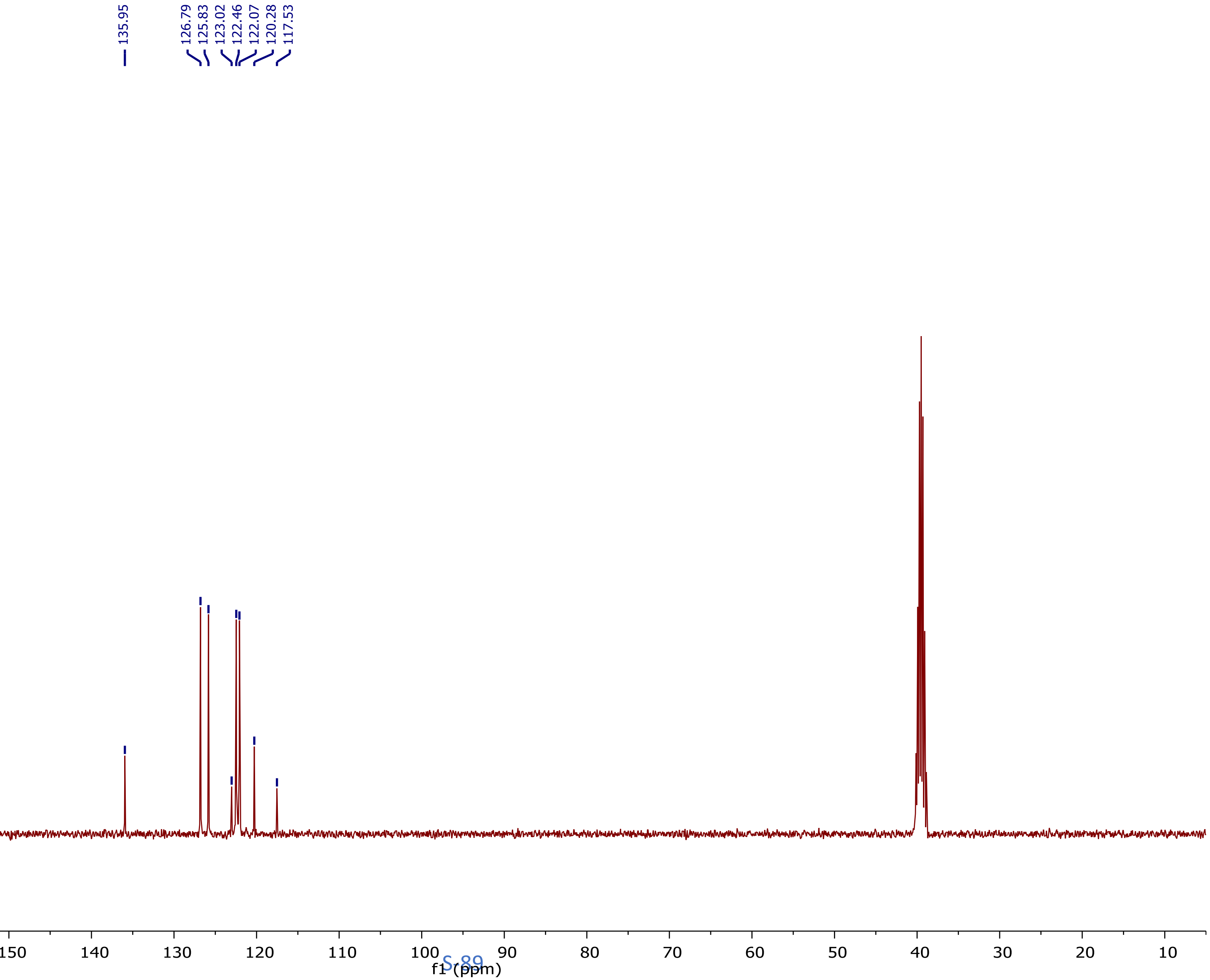
155.94
152.28
135.95
126.79
125.83
123.02
122.46
122.07
120.28
117.53
180
170
160
150
140
130
120
110
100
90
80
70
60
50
40
30
20
10
f1 (ppm)

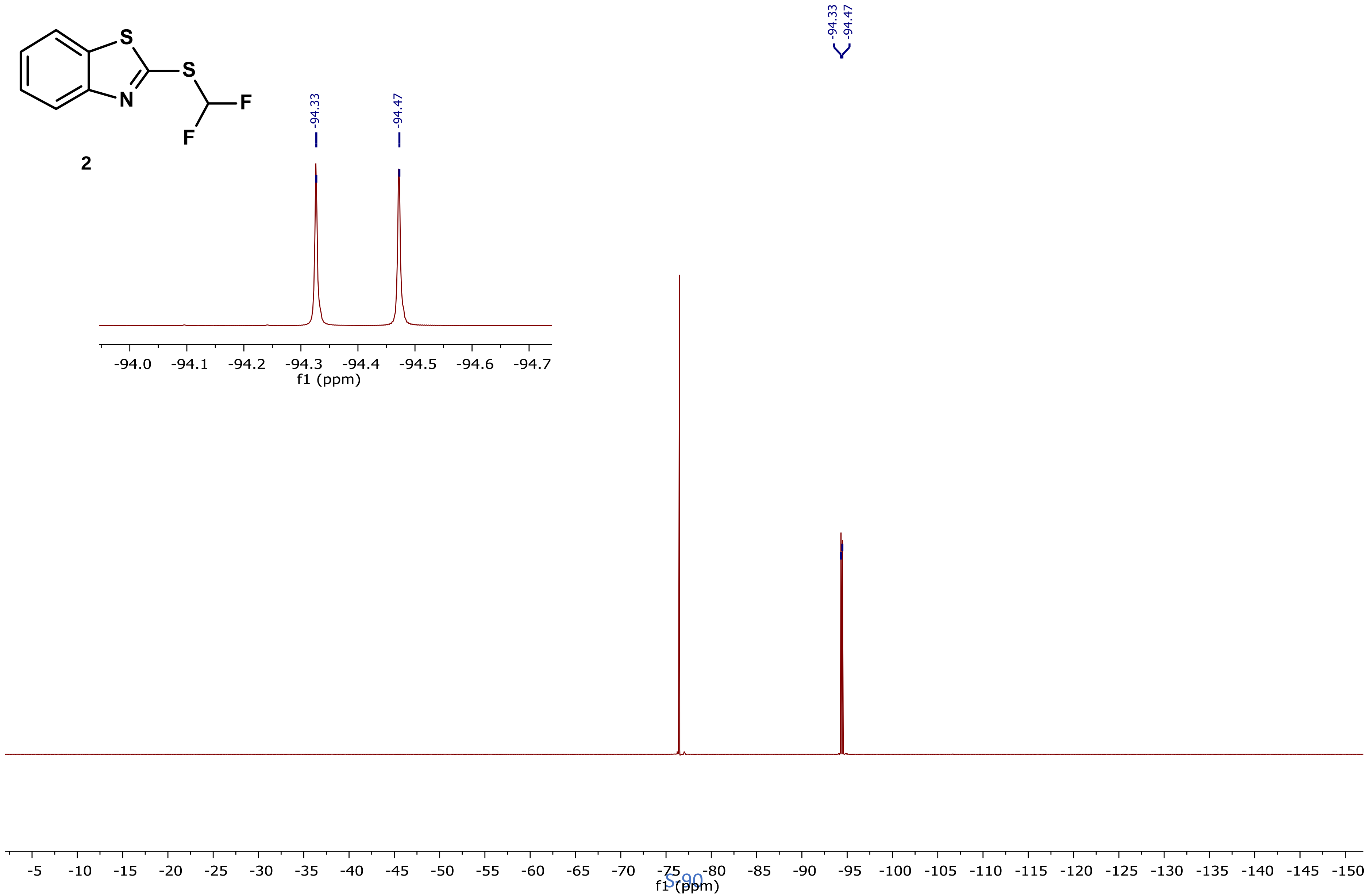
S
S
N
F
F
2
-94.33
-94.47
-94.0 -94.1 -94.2 -94.3 -94.4 -94.5 -94.6 -94.7
f1 (ppm)
-94.33
-94.47
-5 -10 -15 -20 -25 -30 -35 -40 -45 -50 -55 -60 -65 -70 -75 -80 -85 -90 -95 -100 -105 -110 -115 -120 -125 -130 -135 -140 -145 -150
f1 (ppm)

c. ^{1}H, ^{13}C and ^{19}F NMR of 3

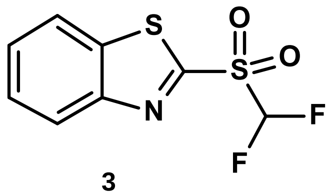

3

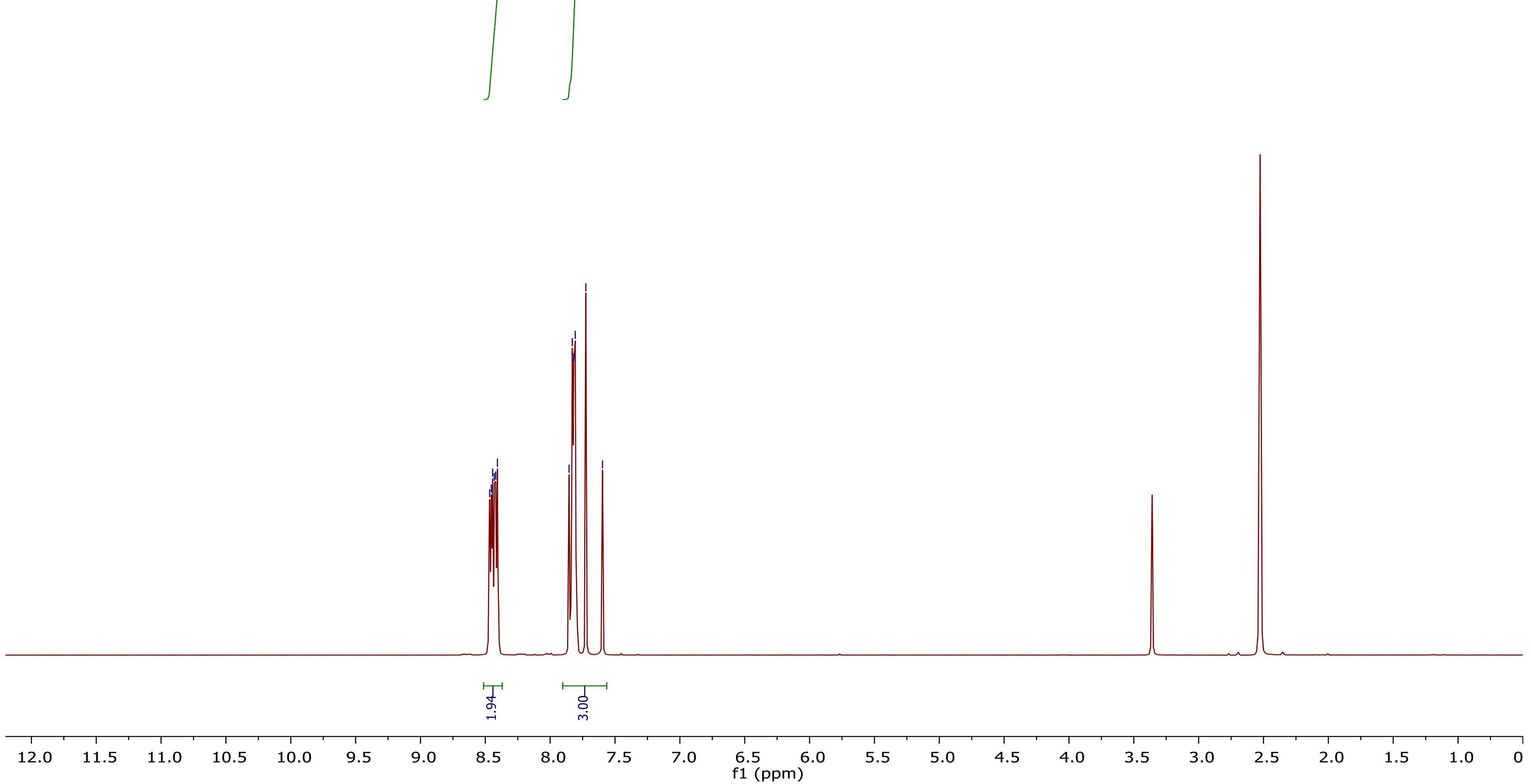
8.47
8.45
8.44
8.43
8.42
8.41
7.85
7.83
7.82
7.81
7.73
7.60
1.94
3.00
12.0
11.5
11.0
10.5
10.0
9.5
9.0
8.5
8.0
7.5
7.0
6.5
6.0
5.5
5.0
4.5
4.0
3.5
3.0
2.5
2.0
1.5
1.0
0
f1 (ppm)

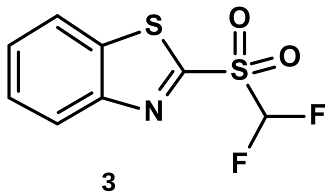
S
O
O
N
F
F

3

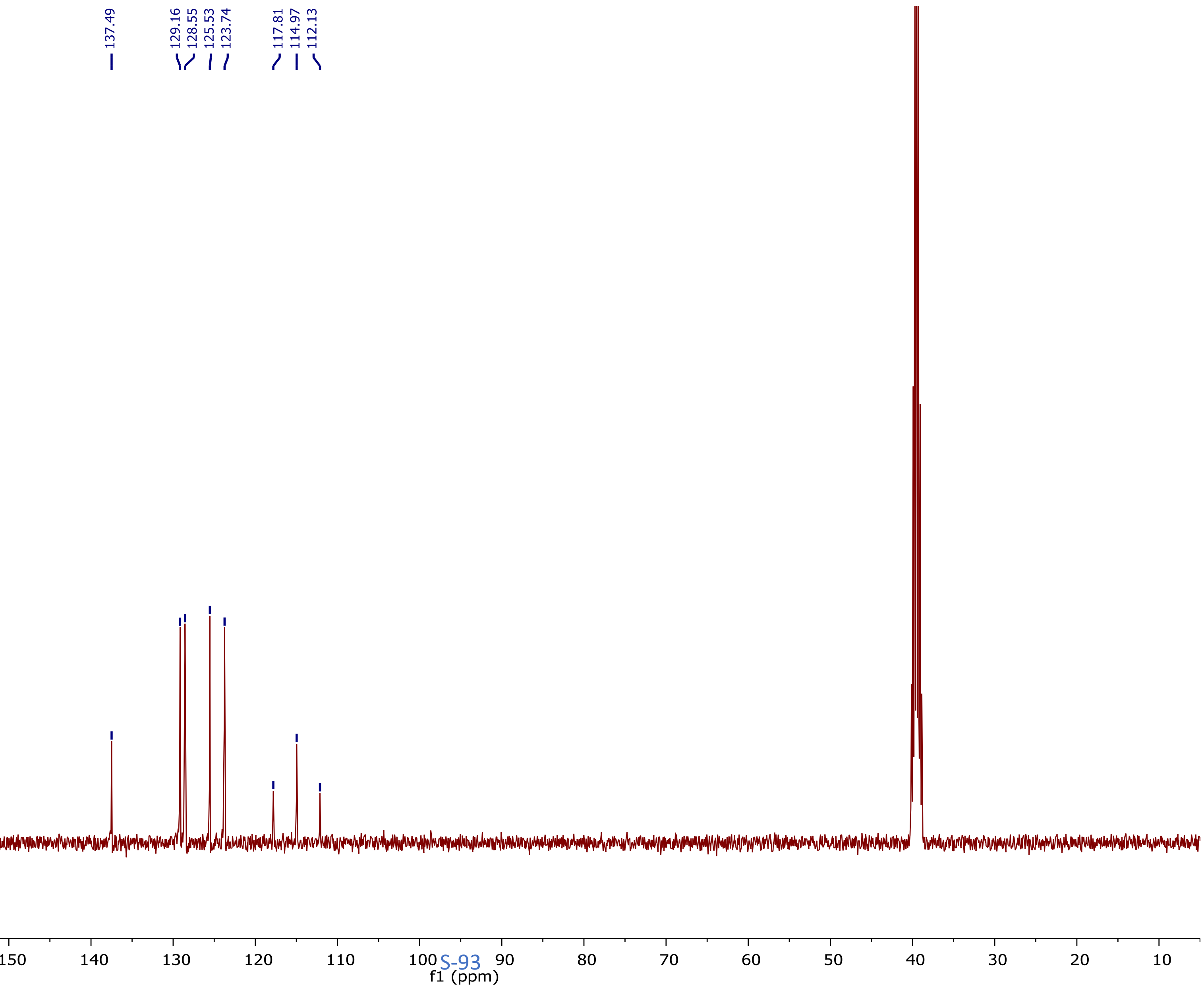
159.01
152.41
137.49
129.16
128.55
125.53
123.74
117.81
114.97
112.13
180
170
160
150
140
130
120
110
100
90
80
70
60
50
40
30
20
10
f1 (ppm)

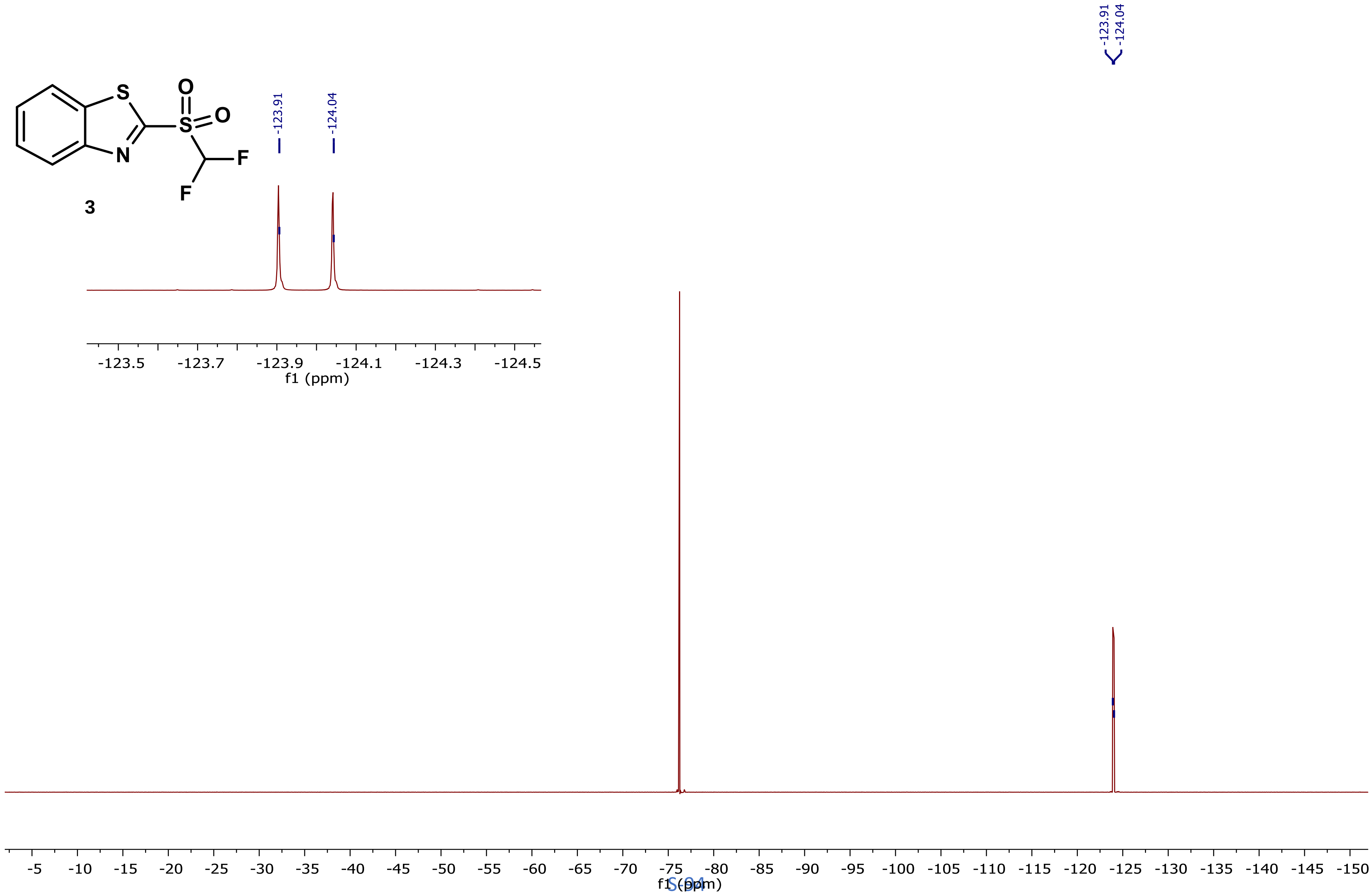

-123.91
-124.04
S
O
O
N
F
F
-123.91
-124.04
-123.5
-123.7
-123.9
-124.1
-124.3
-124.5
f1 (ppm)
-5 -10 -15 -20 -25 -30 -35 -40 -45 -50 -55 -60 -65 -70 -75 -80 -85 -90 -95 -100 -105 -110 -115 -120 -125 -130 -135 -140 -145 -150
f1 (ppm)

3

d. ^{1}H, ^{13}C and ^{19}F NMR of 5

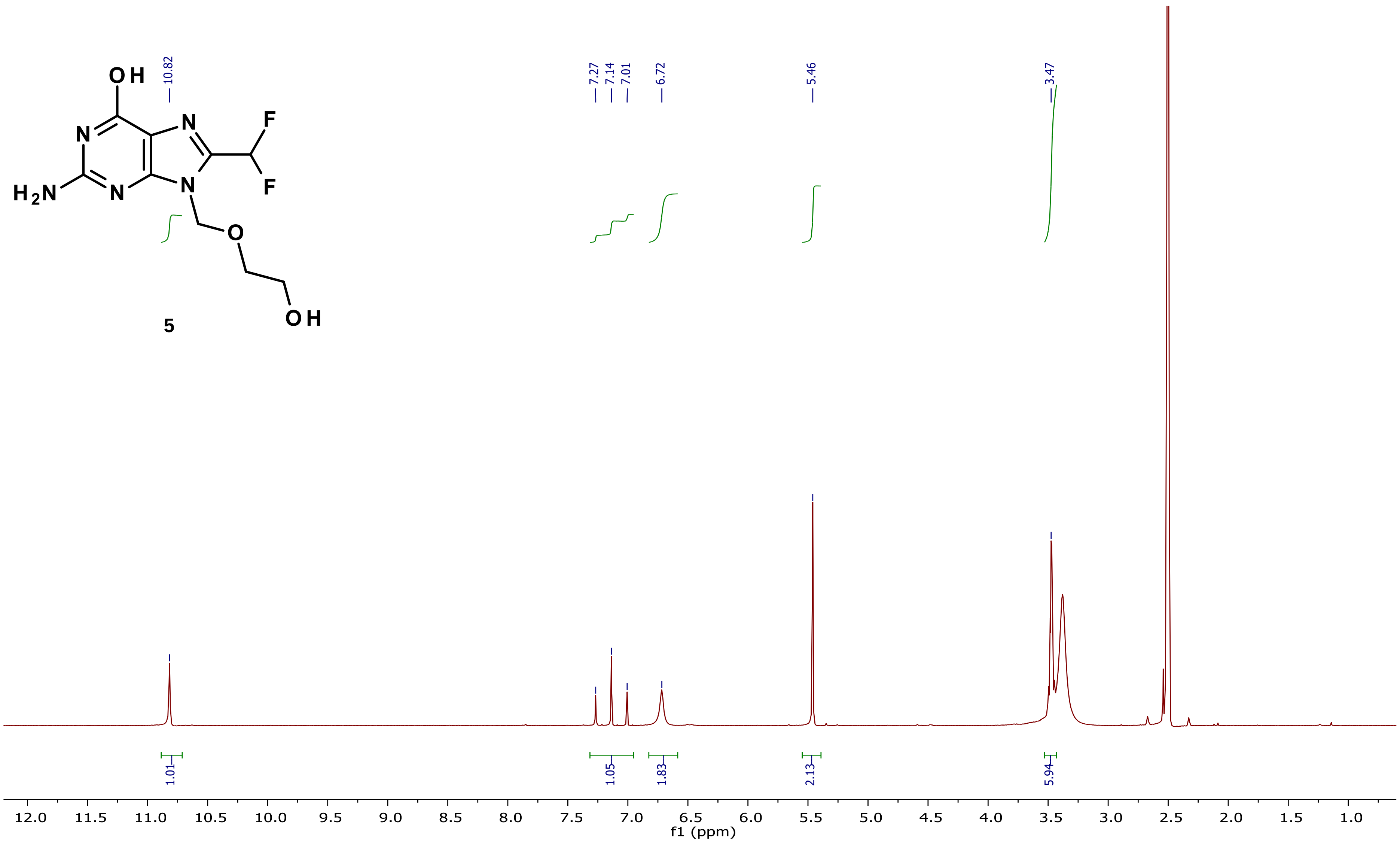

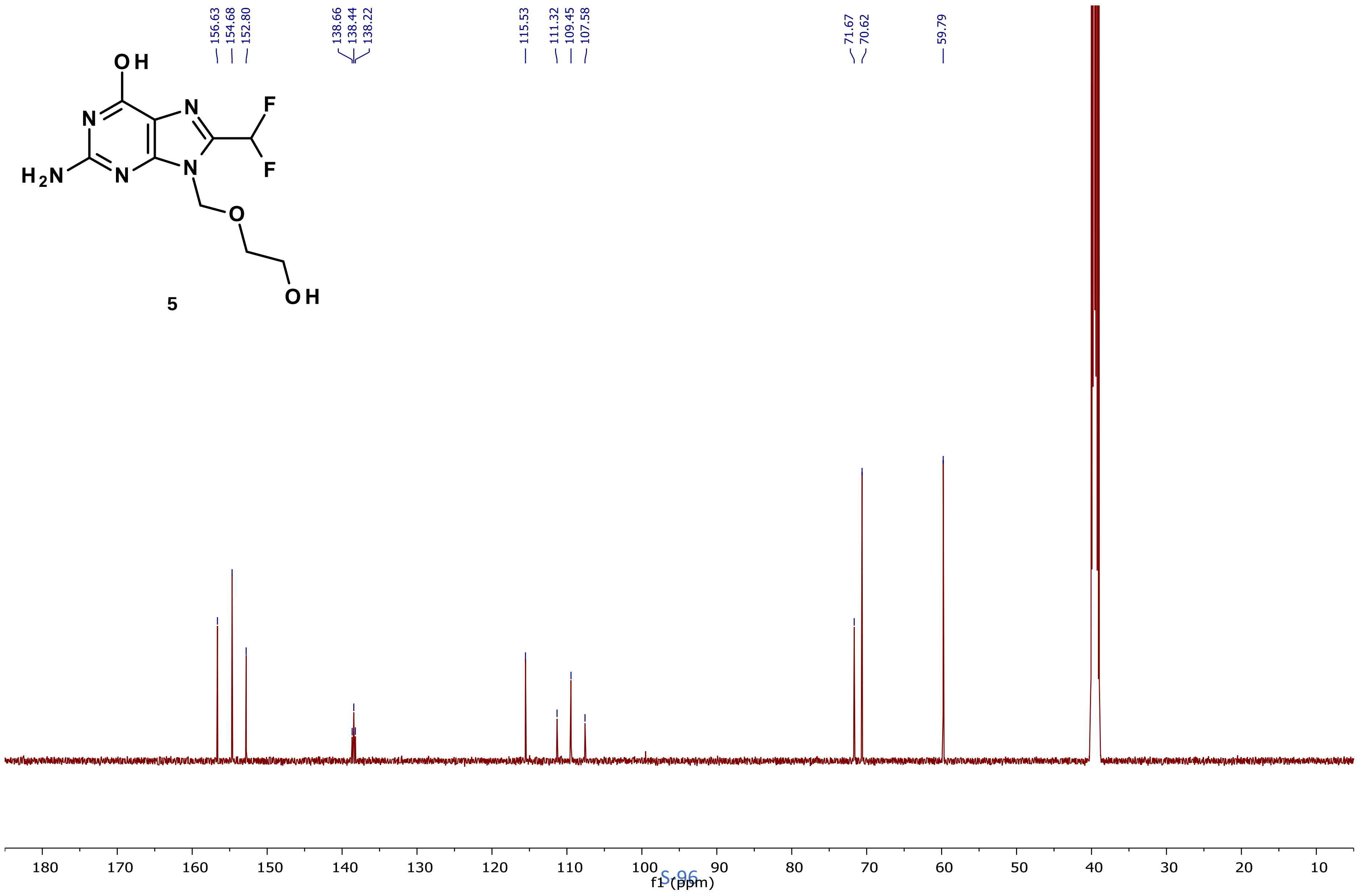
156.63
154.68
152.80
138.66
138.44
138.22
115.53
111.32
109.45
107.58
71.67
70.62
59.79
OH
N
N
H2N
N
N
F
F
O
OH
5
180
170
160
150
140
130
120
110
100
90
80
70
60
50
40
30
20
10
f1 (ppm)

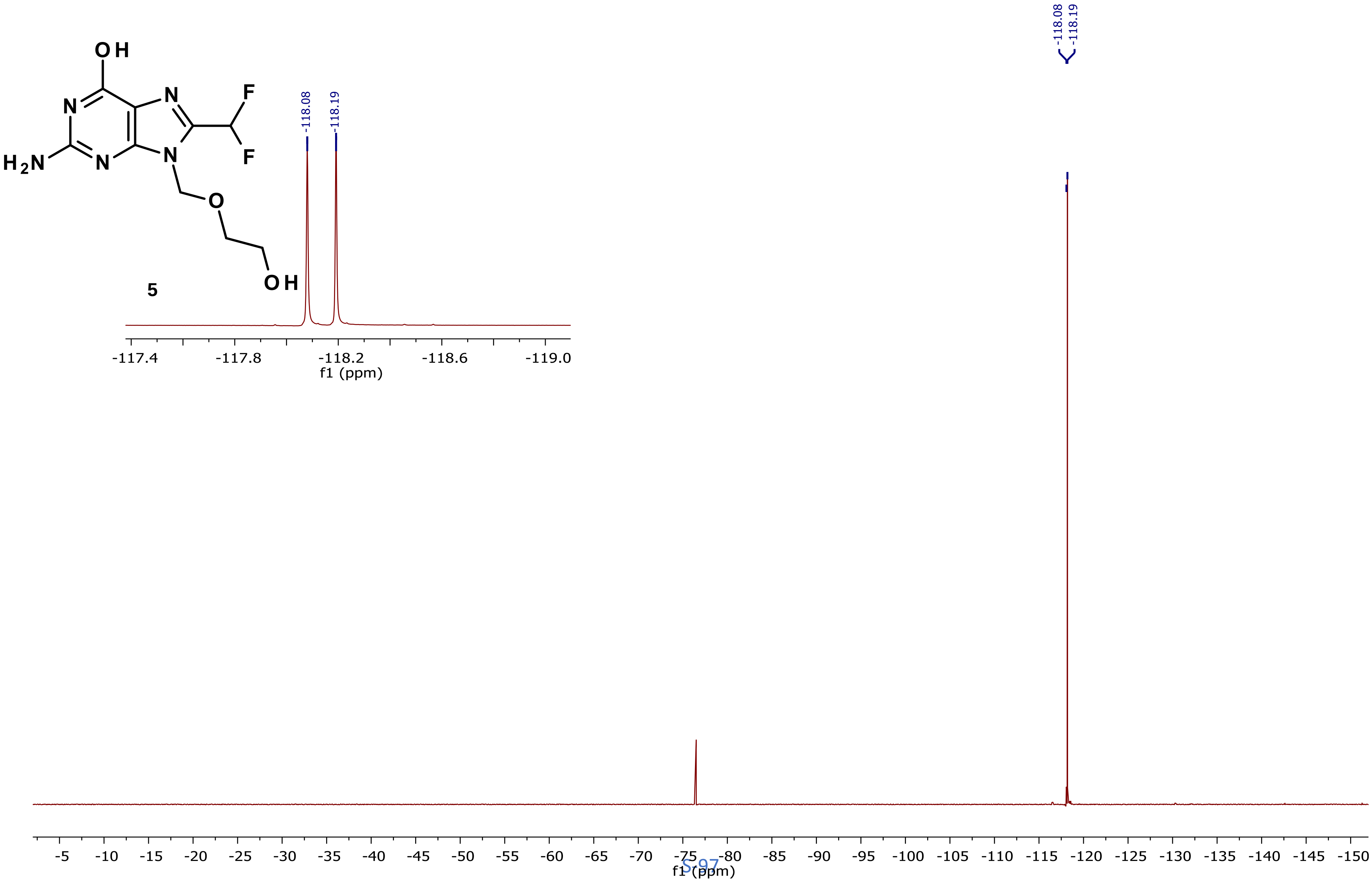
OH
N
N
F
H2N
N
N
F
O
OH
5
-118.08
-118.19
-117.4
-117.8
-118.2
-118.6
-119.0
f1 (ppm)
-5 -10 -15 -20 -25 -30 -35 -40 -45 -50 -55 -60 -65 -70 -75 -80 -85 -90 -95 -100 -105 -110 -115 -120 -125 -130 -135 -140 -145 -150
f1 (ppm)

e. ^{1}H, cosy, NOE, ^{13}C and ^{19}F NMR of 6

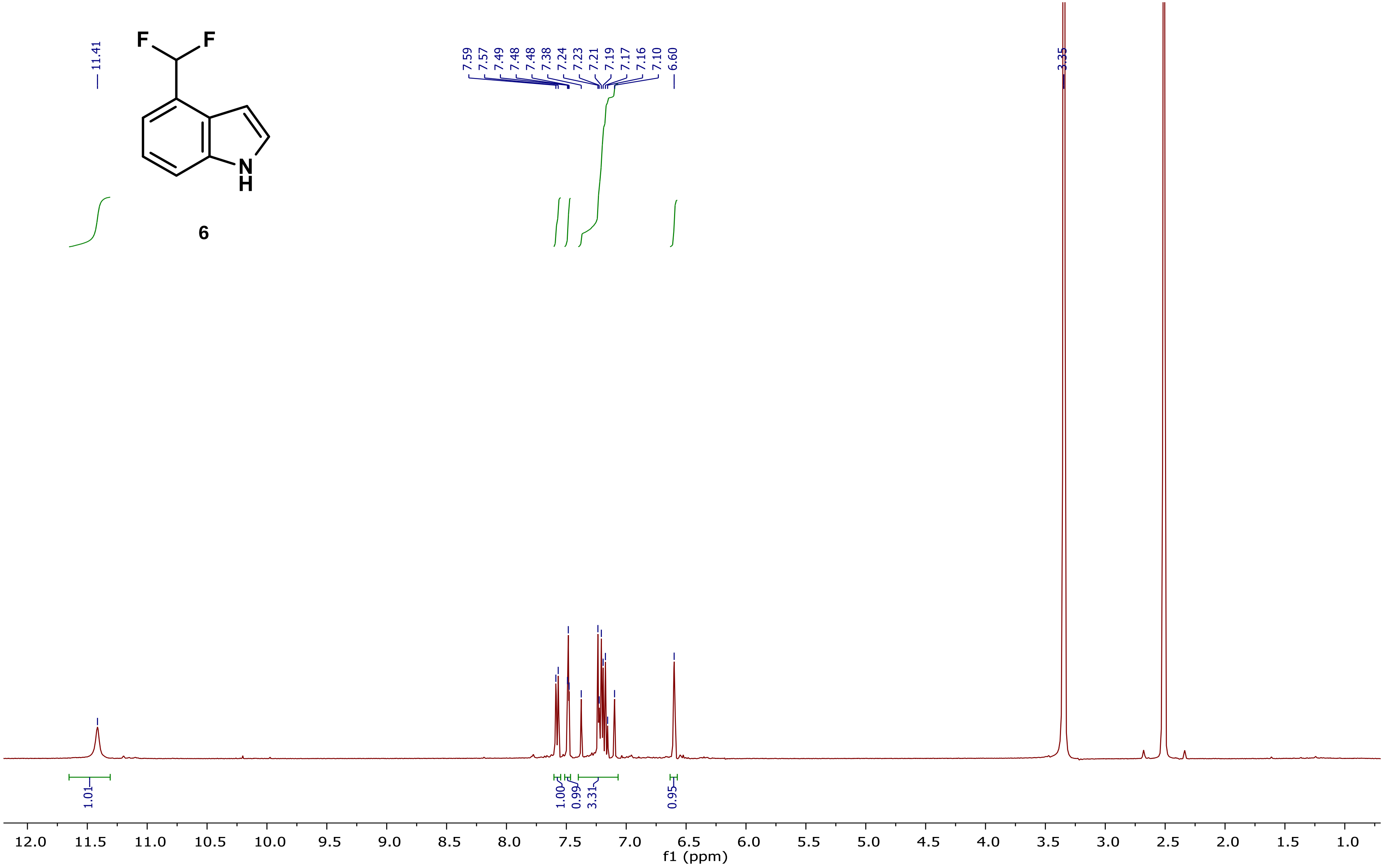

6

11.41
7.59
7.57
7.49
7.48
7.48
7.38
7.24
7.23
7.21
7.19
7.17
7.16
7.10
6.60
3.35
1.01
1.00
0.99
3.31
0.95
12.0 11.5 11.0 10.5 10.0 9.5 9.0 8.5 8.0 7.5 7.0 6.5 6.0 5.5 5.0 4.5 4.0 3.5 3.0 2.5 2.0 1.5 1.0
f1 (ppm)

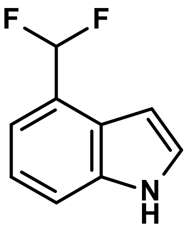

6

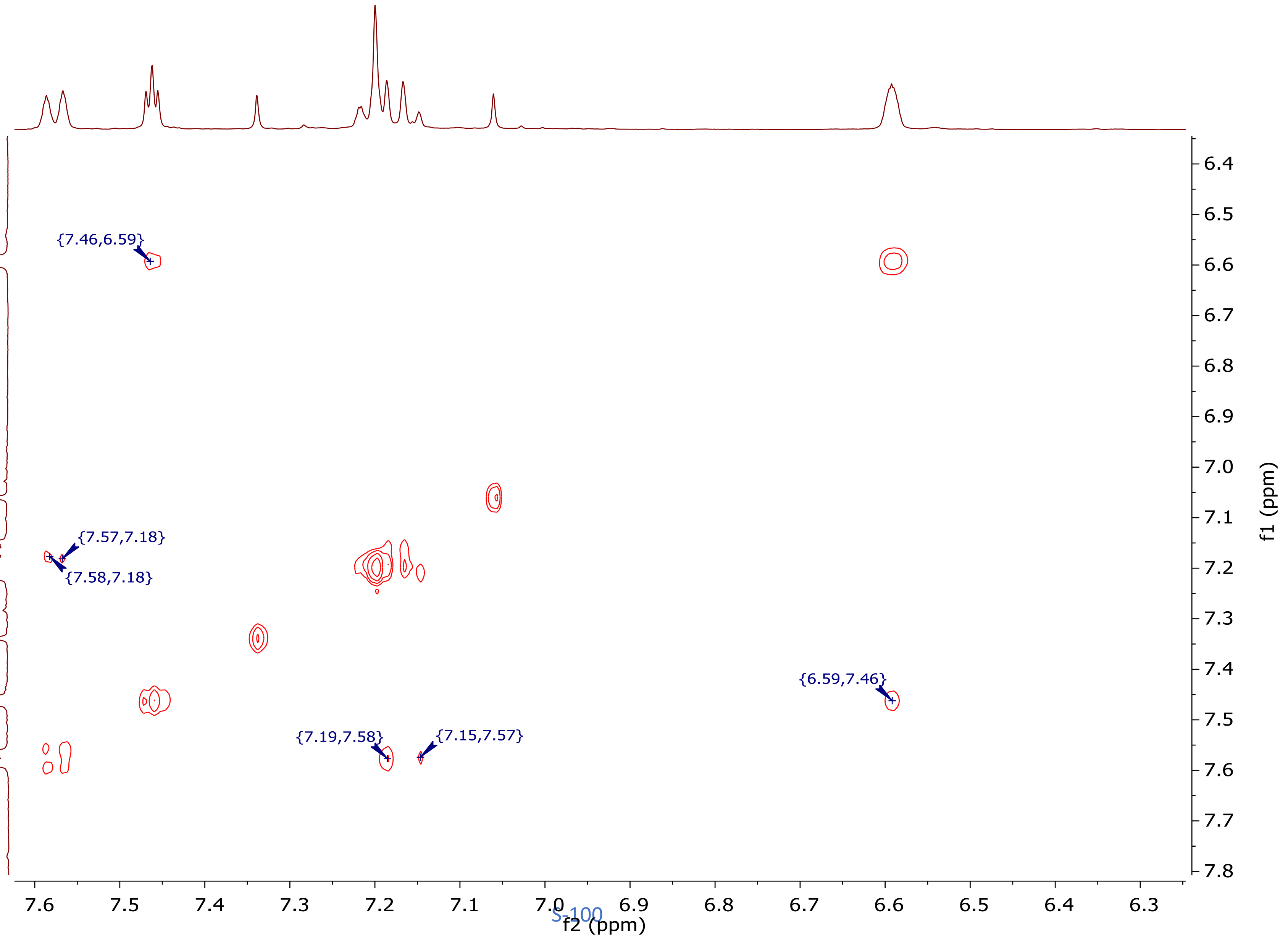

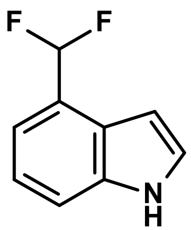
F
F
N
H

6

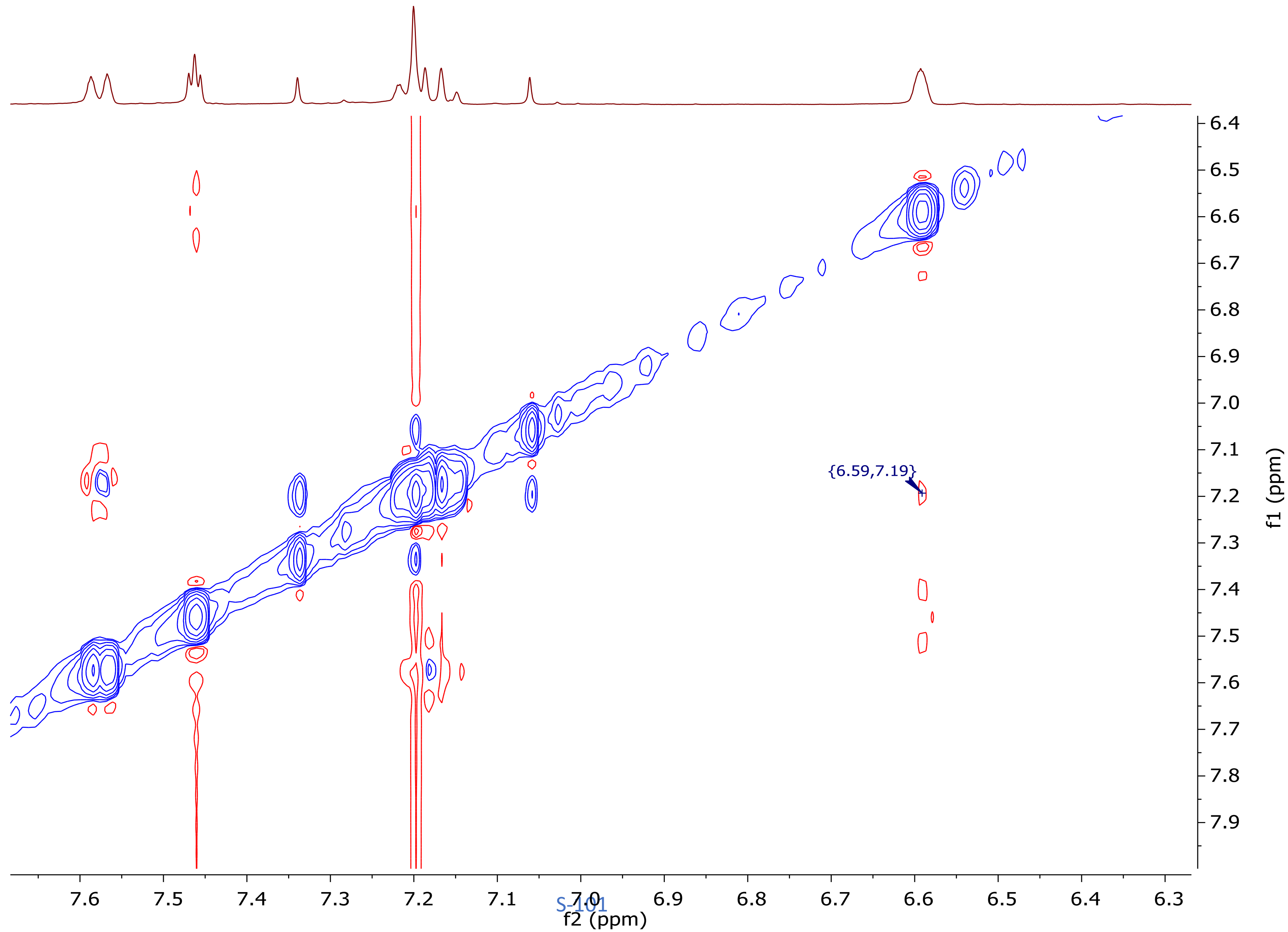
{6.59,7.19}
f1 (ppm)
f2 (ppm)
7.6
7.5
7.4
7.3
7.2
7.1
7.0
6.9
6.8
6.7
6.6
6.5
6.4
6.3
7.9

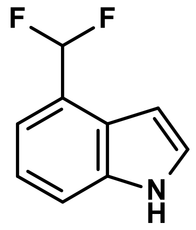

6

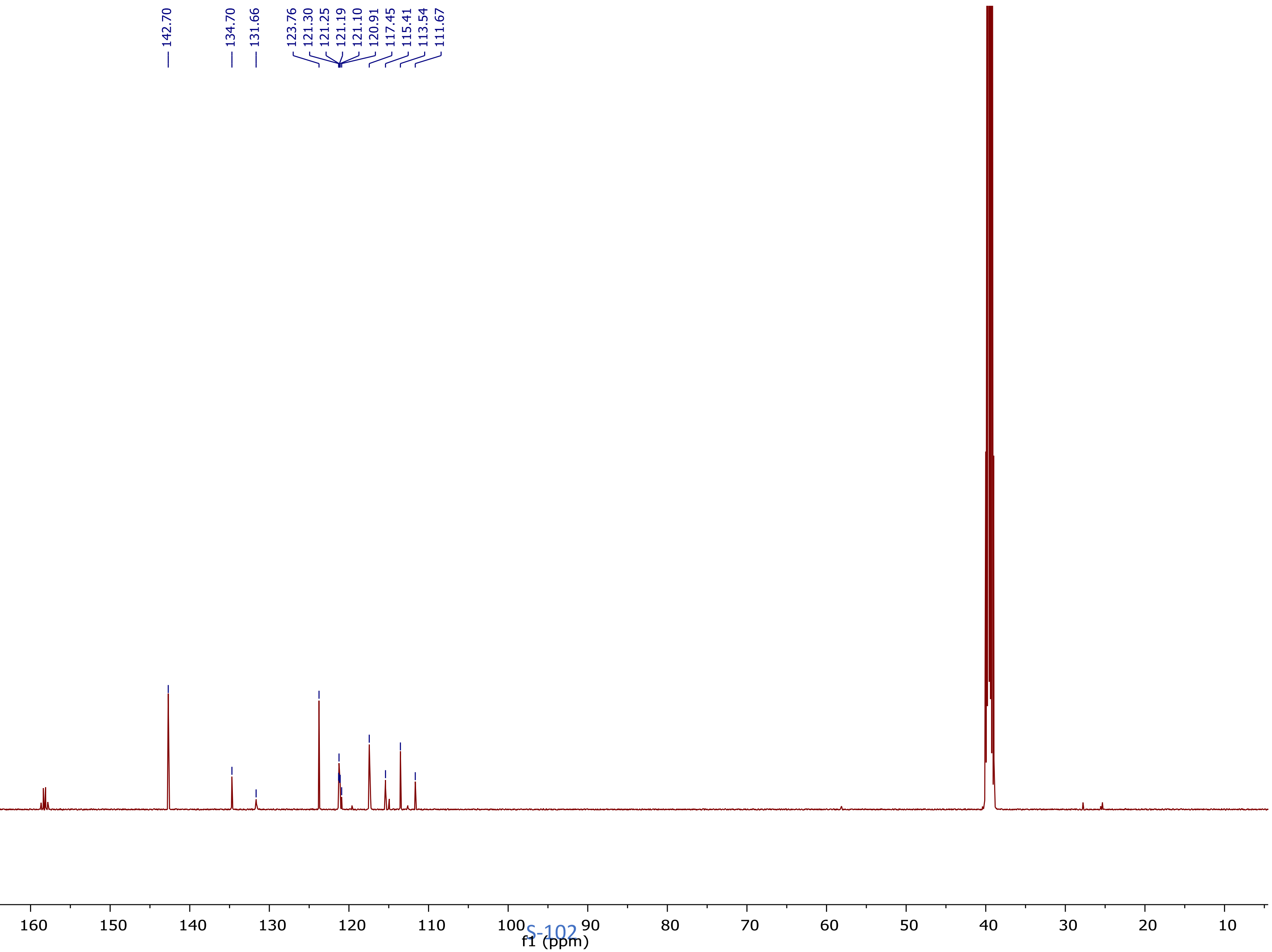
142.70
134.70
131.66
123.76
121.30
121.25
121.19
121.10
120.91
117.45
115.41
113.54
111.67
180
170
160
150
140
130
120
110
100
90
80
70
60
50
40
30
20
10
f1 (ppm)

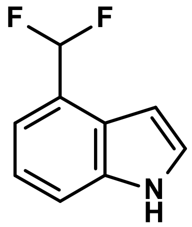
F
F
N
H

6

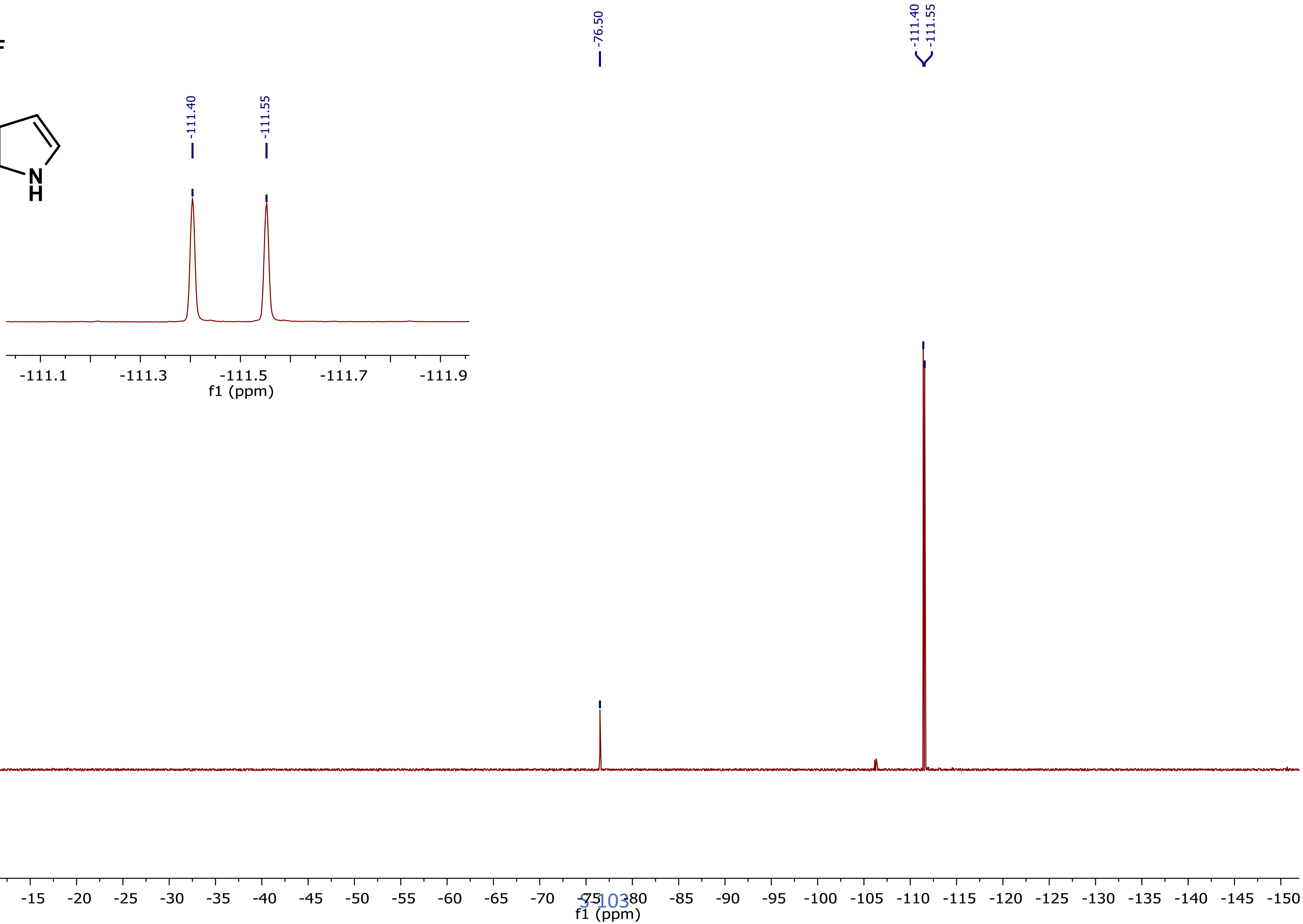
-76.50
-111.40
-111.55
-111.1
-111.3
-111.5
-111.7
-111.9
f1 (ppm)
-5 -10 -15 -20 -25 -30 -35 -40 -45 -50 -55 -60 -65 -70 -75 -80 -85 -90 -95 -100 -105 -110 -115 -120 -125 -130 -135 -140 -145 -150
f1 (ppm)

f. ^{1}H, ^{13}C and ^{19}F NMR of 7a

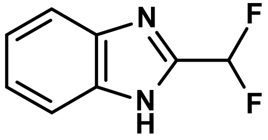

7a

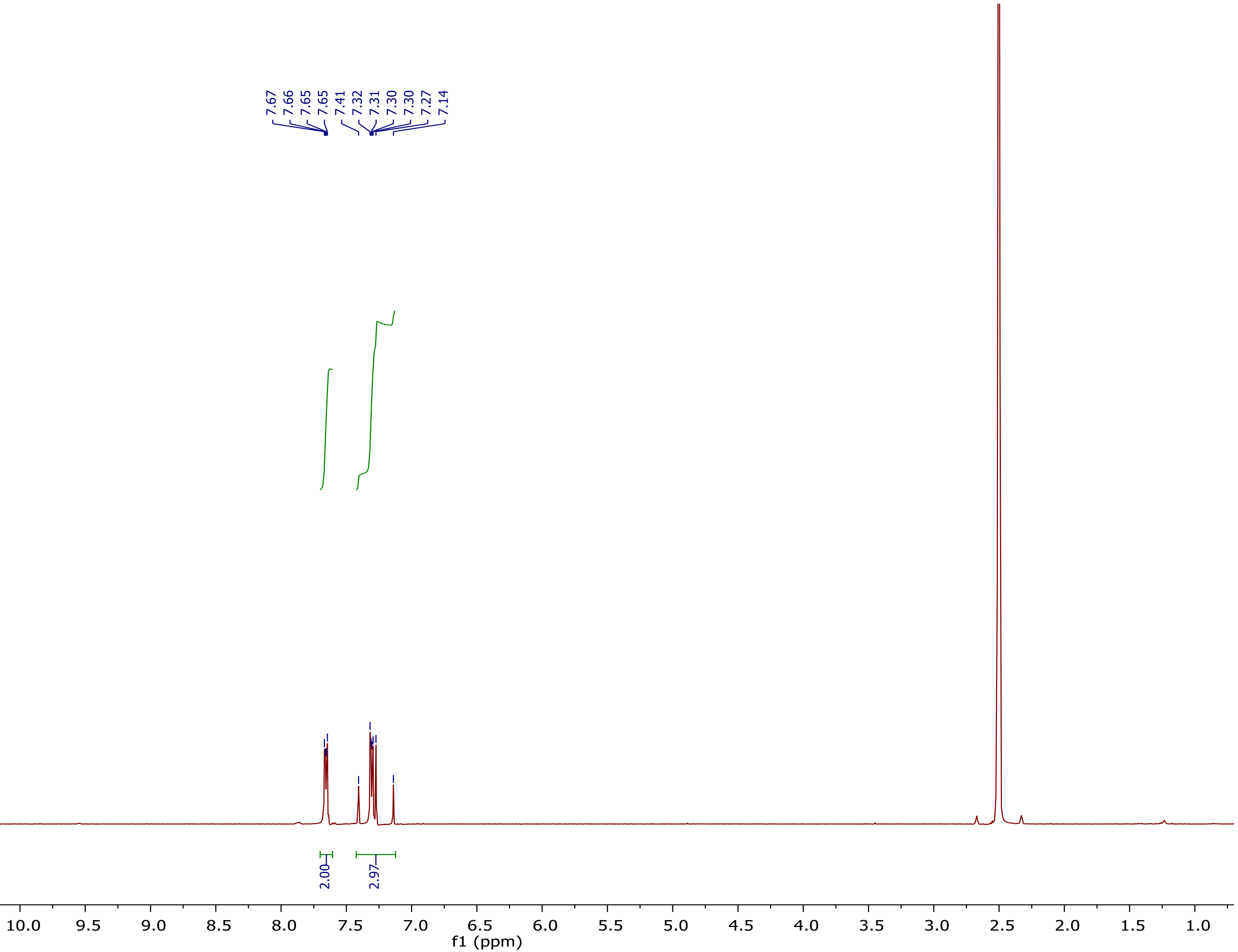
7.67
7.66
7.65
7.65
7.41
7.32
7.31
7.30
7.30
7.27
7.14
2.00
2.97
12.0 11.5 11.0 10.5 10.0 9.5 9.0 8.5 8.0 7.5 7.0 6.5 6.0 5.5 5.0 4.5 4.0 3.5 3.0 2.5 2.0 1.5 1.0
f1 (ppm)

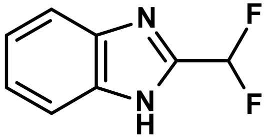
N
N
H
F
F

7a

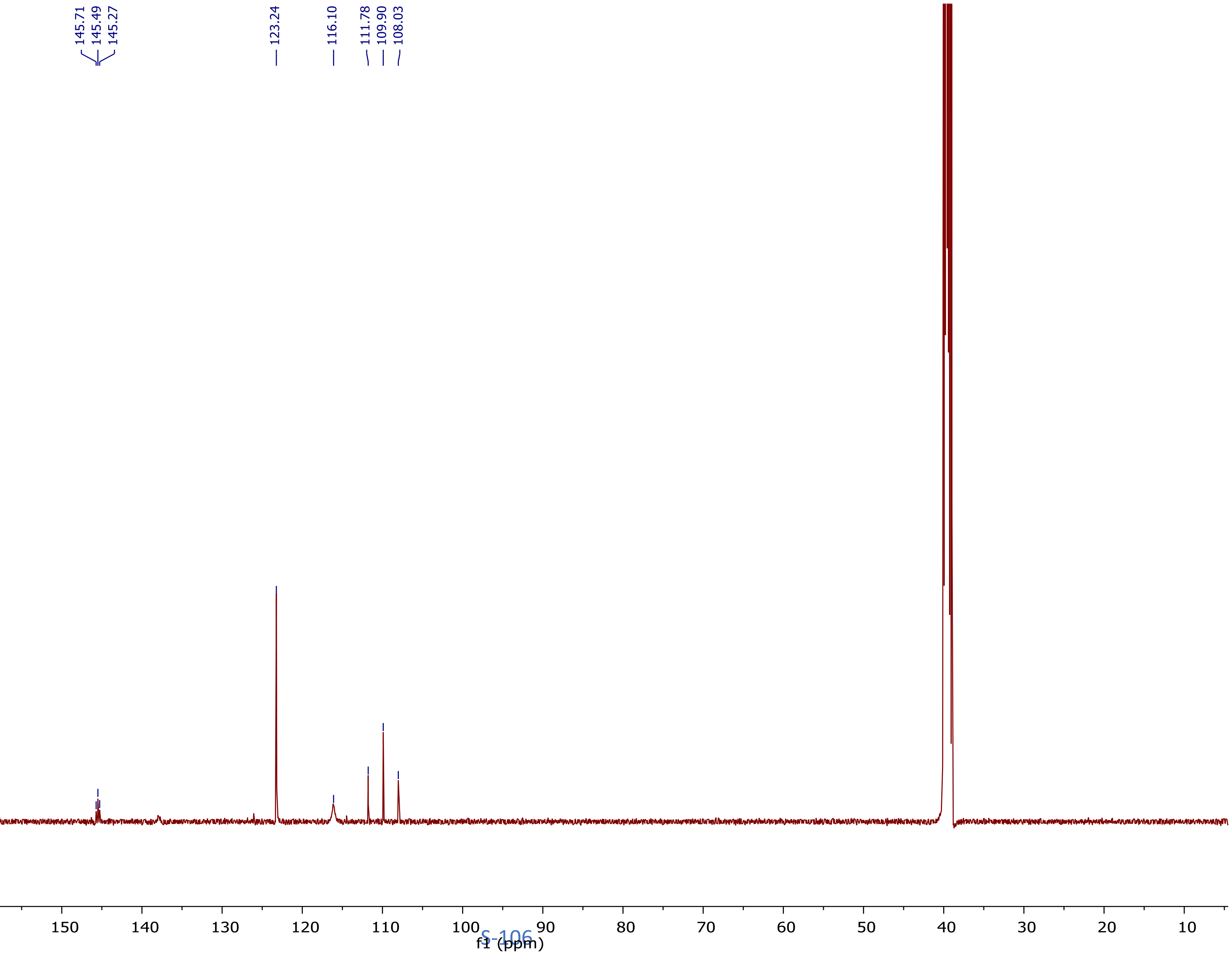
145.71
145.49
145.27
123.24
116.10
111.78
109.90
108.03
180
170
160
150
140
130
120
110
100
90
80
70
60
50
40
30
20
10
f1 (ppm)

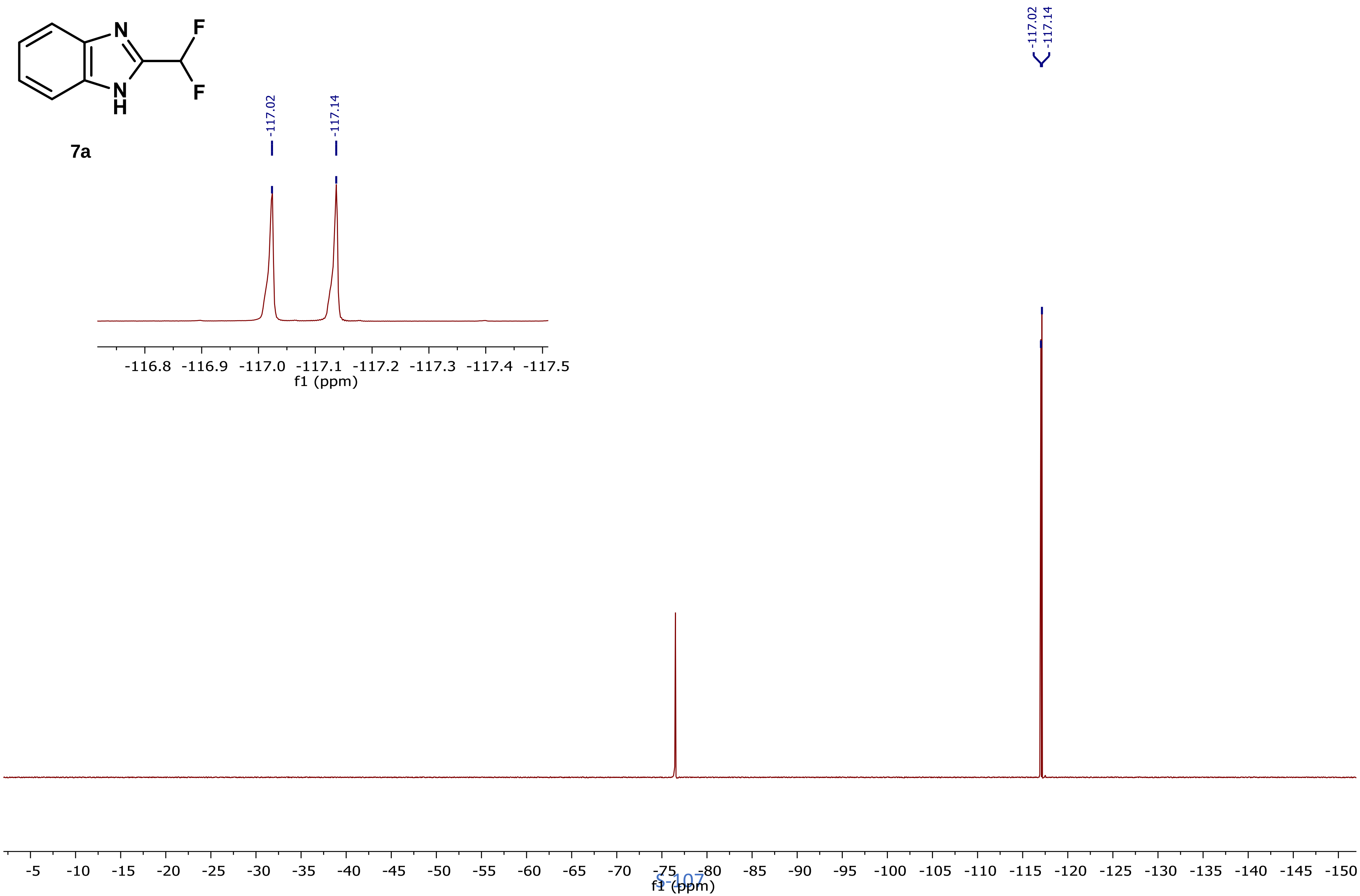

N
F
N
H
F
7a
-117.02
-117.14
-116.8 -116.9 -117.0 -117.1 -117.2 -117.3 -117.4 -117.5
f1 (ppm)
-5 -10 -15 -20 -25 -30 -35 -40 -45 -50 -55 -60 -65 -70 -75 -80 -85 -90 -95 -100 -105 -110 -115 -120 -125 -130 -135 -140 -145 -150
f1 (ppm)

g. ^{1}H, ^{13}C and ^{19}F NMR of 7b

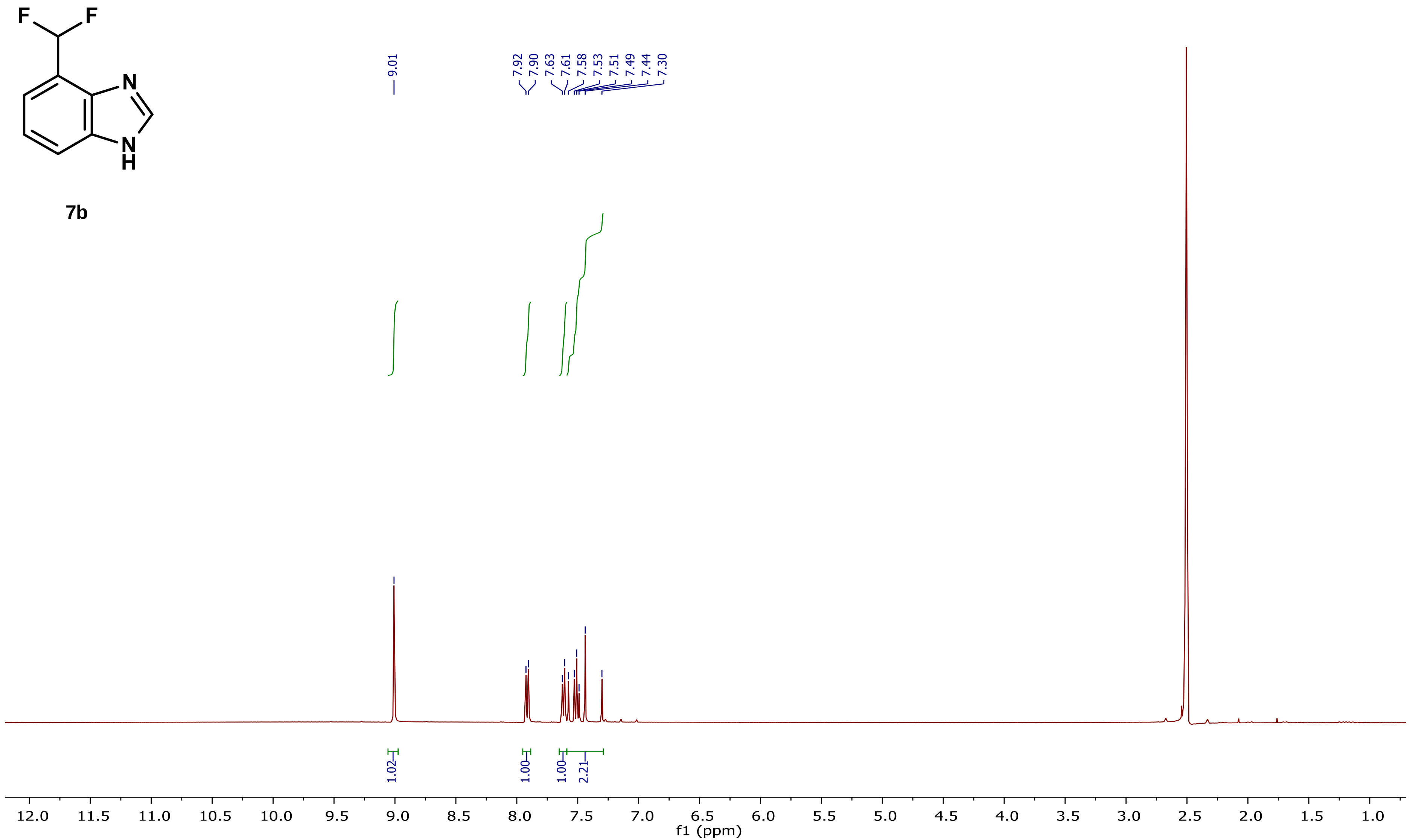

7b
9.01
7.92
7.90
7.63
7.61
7.58
7.53
7.51
7.49
7.44
7.30
1.02
1.00
1.00
2.21
12.0
11.5
11.0
10.5
10.0
9.5
9.0
8.5
8.0
7.5
7.0
6.5
6.0
5.5
5.0
4.5
4.0
3.5
3.0
2.5
2.0
1.5
1.0
f1 (ppm)

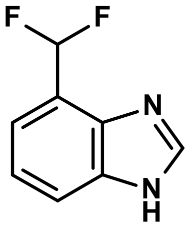

7b

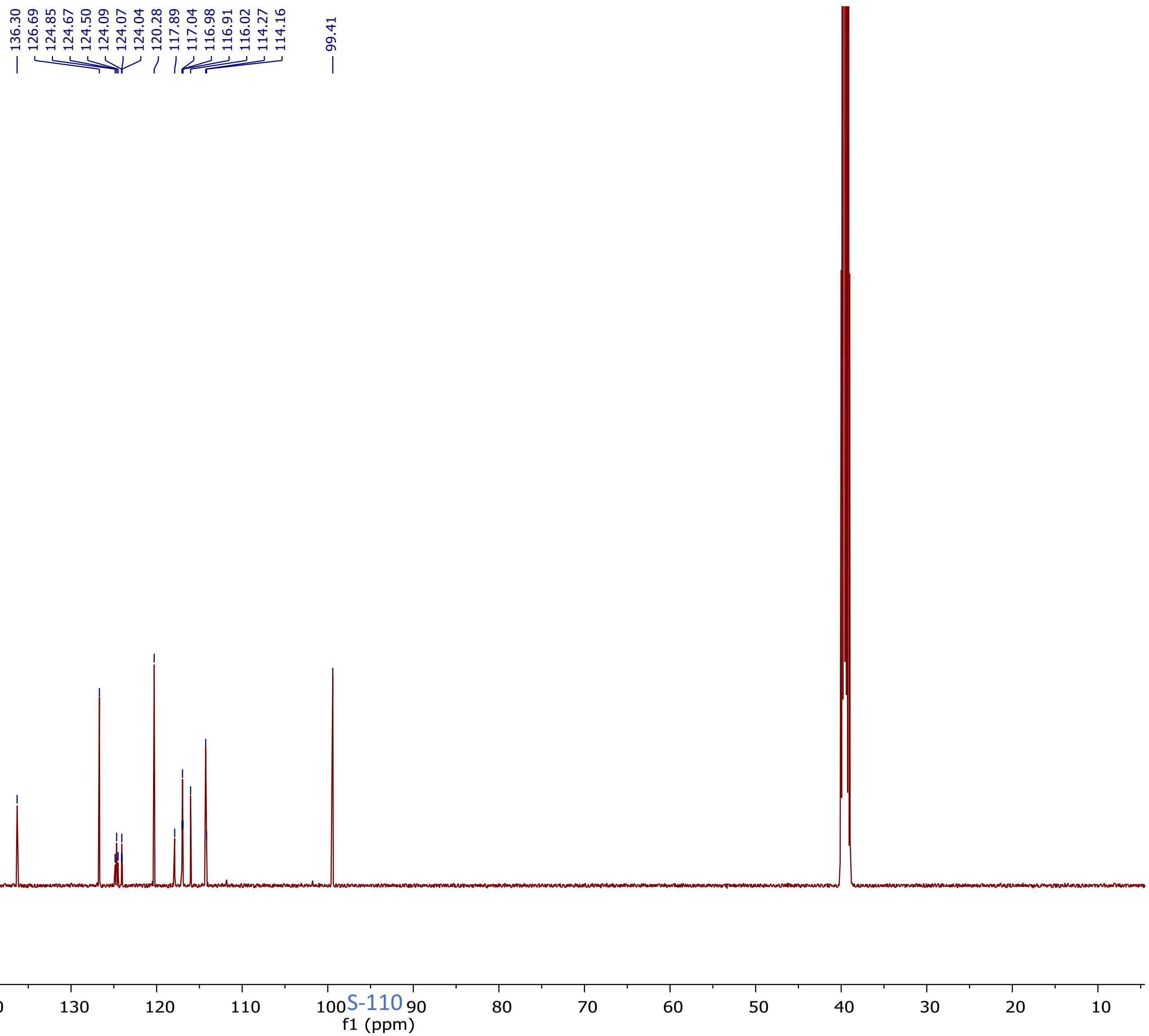
136.30
126.69
124.85
124.67
124.50
124.09
124.07
124.04
120.28
117.89
117.04
116.98
116.91
116.02
114.27
114.16
99.41
180
170
160
150
140
130
120
110
100
90
80
70
60
50
40
30
20
10
f1 (ppm)

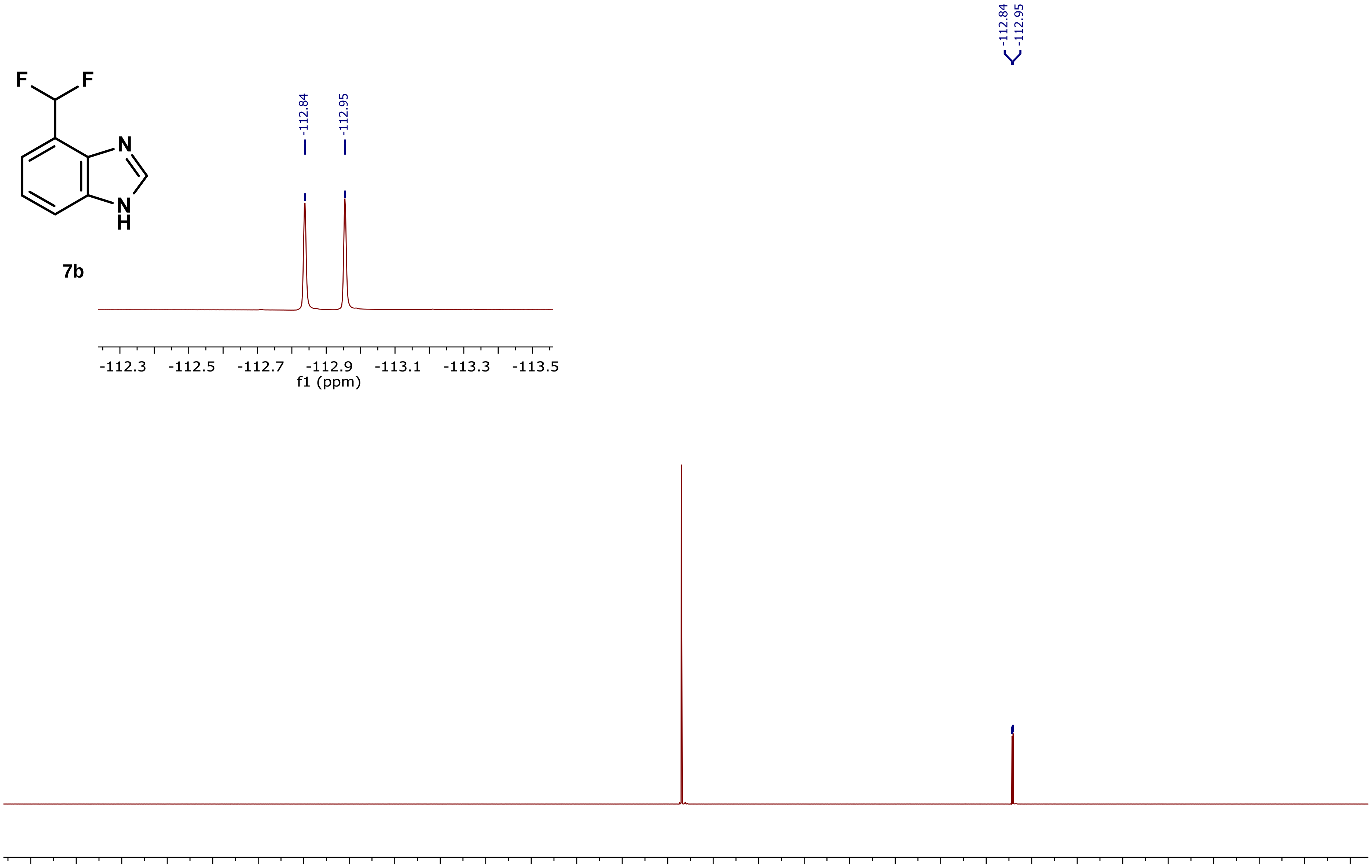
7b
-112.84
-112.95
-112.3 -112.5 -112.7 -112.9 -113.1 -113.3 -113.5
f1 (ppm)
-5 -10 -15 -20 -25 -30 -35 -40 -45 -50 -55 -60 -65 -70 -75 -80 -85 -90 -95 -100 -105 -110 -115 -120 -125 -130 -135 -140 -145 -150
f1 (ppm)

h. ^{1}H, ^{13}C and ^{19}F NMR of 7c

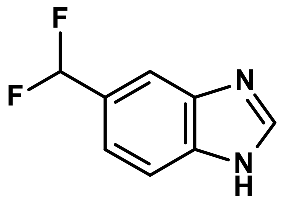

7c

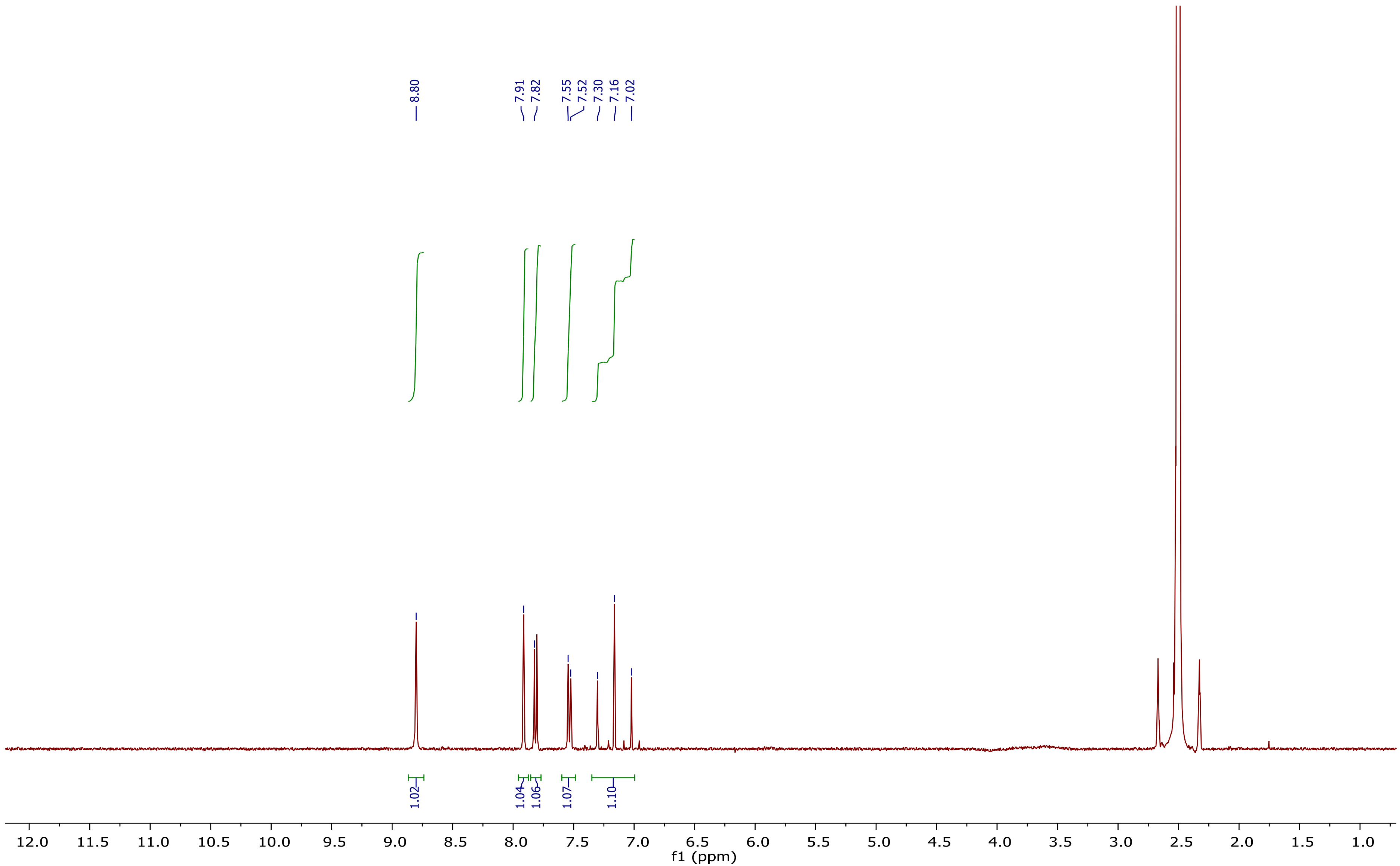
8.80
7.91
7.82
7.55
7.52
7.30
7.16
7.02
1.02
1.04
1.06
1.07
1.10
12.0 11.5 11.0 10.5 10.0 9.5 9.0 8.5 8.0 7.5 7.0 6.5 6.0 5.5 5.0 4.5 4.0 3.5 3.0 2.5 2.0 1.5 1.0
f1 (ppm)

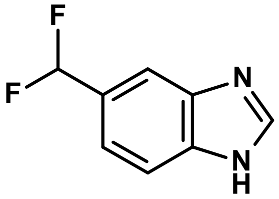

7c

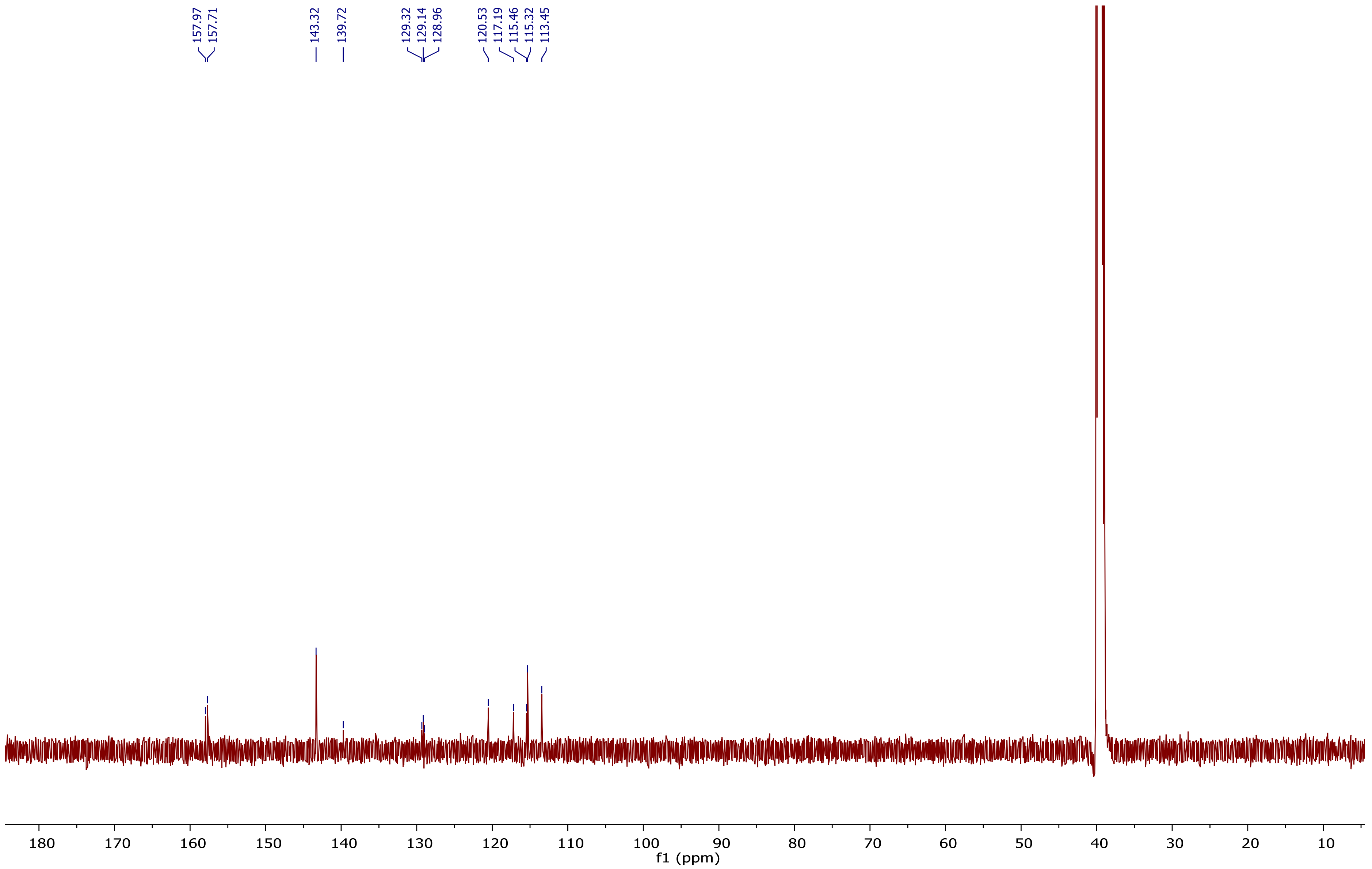
157.97
157.71
143.32
139.72
129.32
129.14
128.96
120.53
117.19
115.46
115.32
113.45
180
170
160
150
140
130
120
110
100
90
80
70
60
50
40
30
20
10
f1 (ppm)

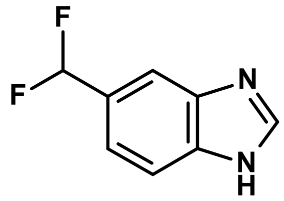
F
F
N
N
H

7c

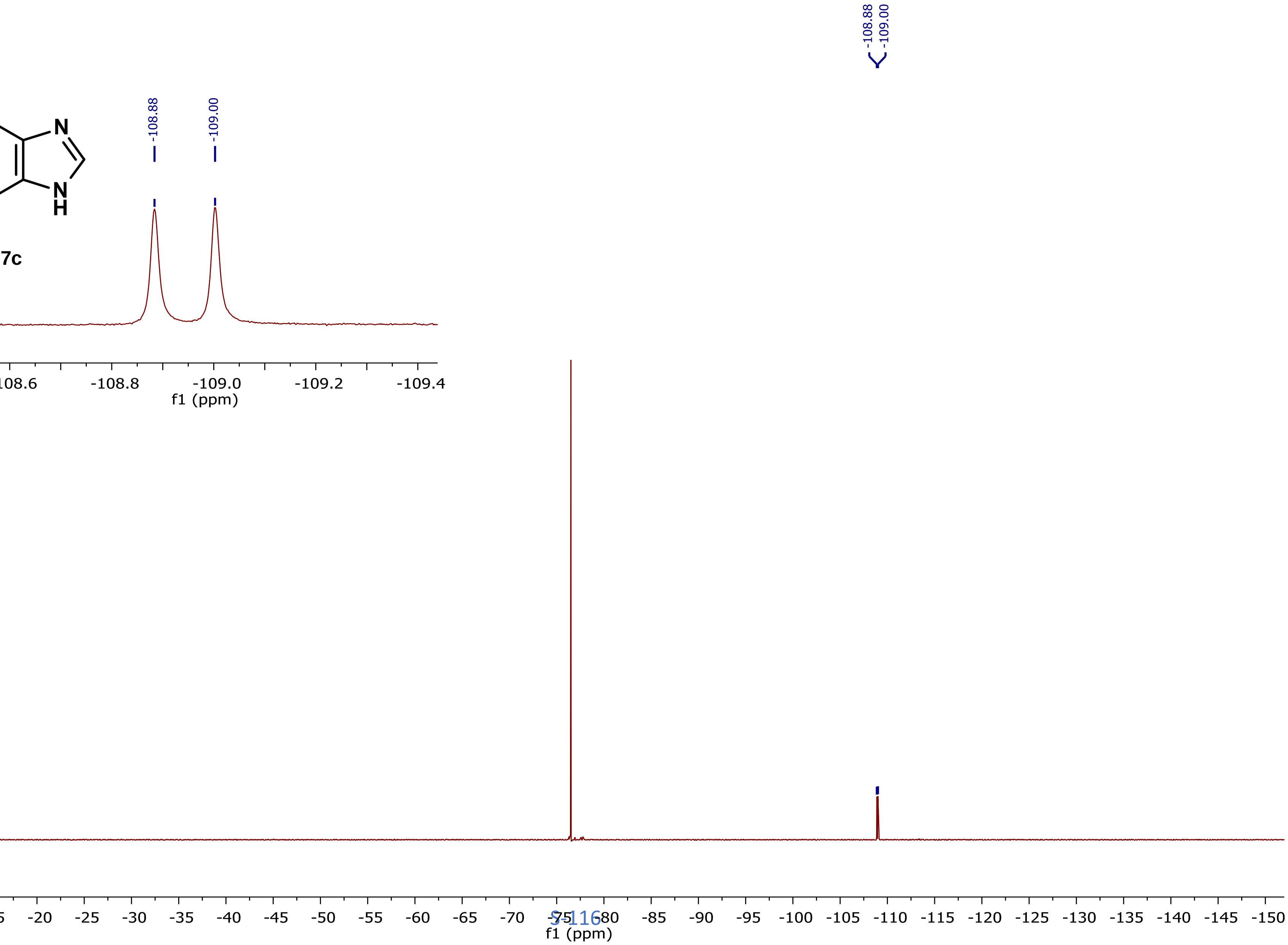
-108.88
-109.00
-108.6
-108.8
-109.0
-109.2
-109.4
f1 (ppm)
-5 -10 -15 -20 -25 -30 -35 -40 -45 -50 -55 -60 -65 -70 -75 -80 -85 -90 -95 -100 -105 -110 -115 -120 -125 -130 -135 -140 -145 -150
f1 (ppm)

i. ^{1}H, cosy, ^{13}C and ^{19}F NMR of 8a

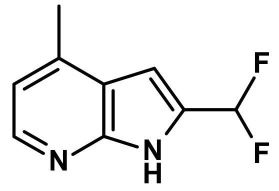
F
F
N
N
H

8a

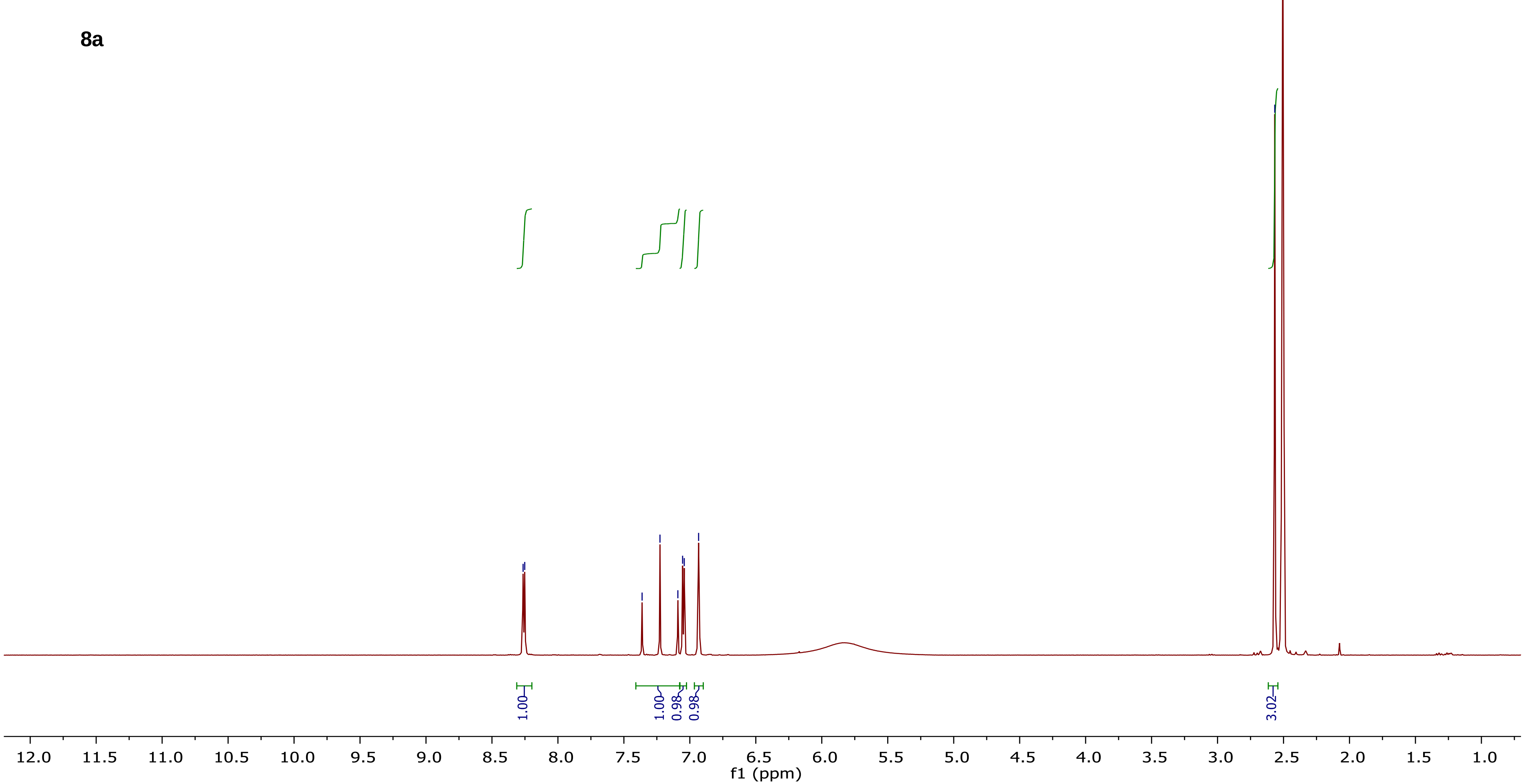
8.26
8.25
7.36
7.23
7.09
7.05
7.04
6.93
2.57
1.00
1.00
0.98
0.98
3.02
12.0
11.5
11.0
10.5
10.0
9.5
9.0
8.5
8.0
7.5
7.0
6.5
6.0
5.5
5.0
4.5
4.0
3.5
3.0
2.5
2.0
1.5
1.0
f1 (ppm)

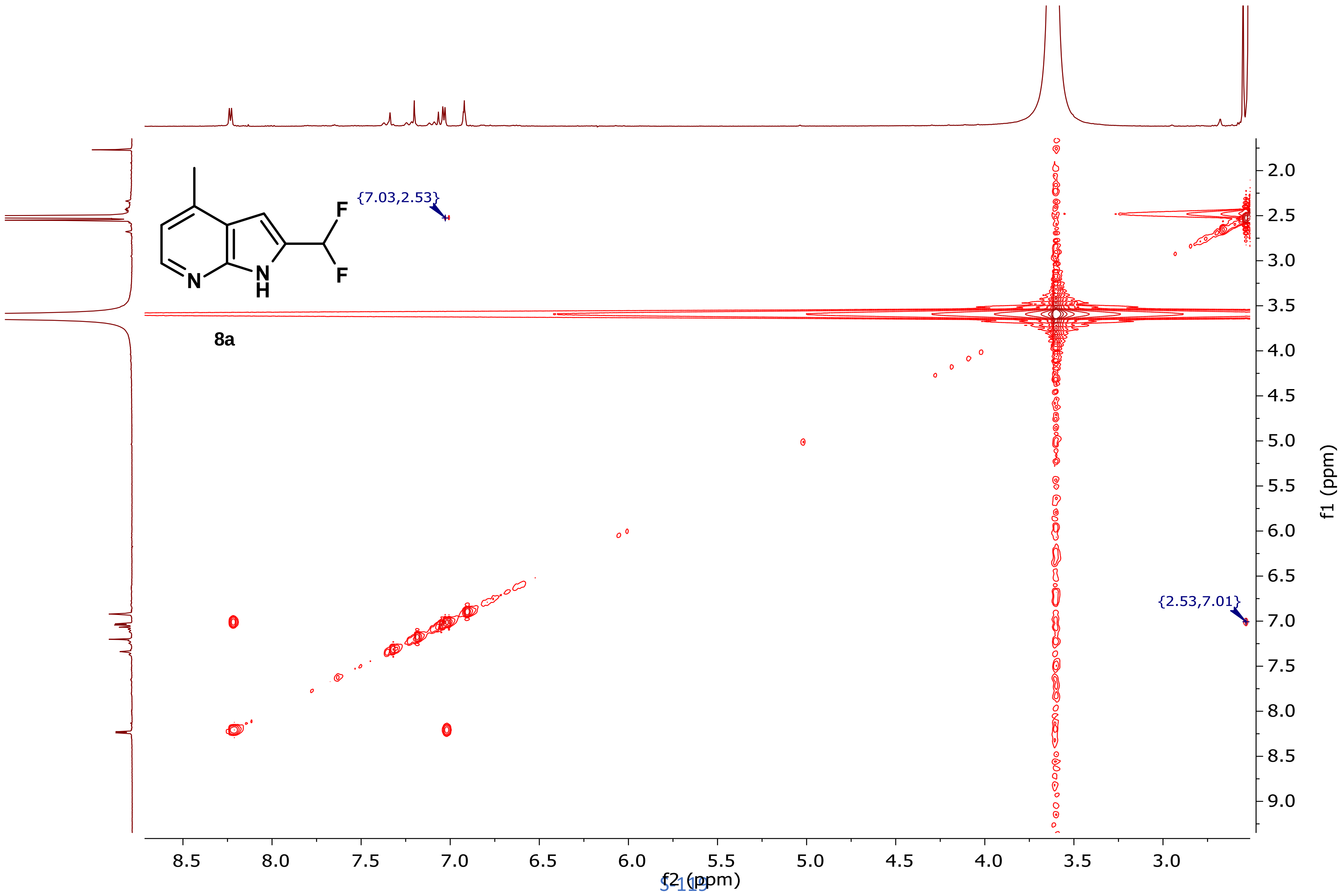
F
F
N
N
H
8a
{7.03,2.53}
{2.53,7.01}
2.0
2.5
3.0
3.5
4.0
4.5
5.0
5.5
6.0
6.5
7.0
7.5
8.0
8.5
9.0
f1 (ppm)
8.5
8.0
7.5
7.0
6.5
6.0
5.5
5.0
4.5
4.0
3.5
3.0
f2 (ppm)

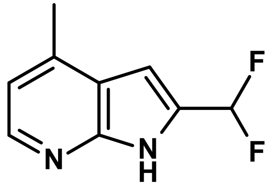

8a

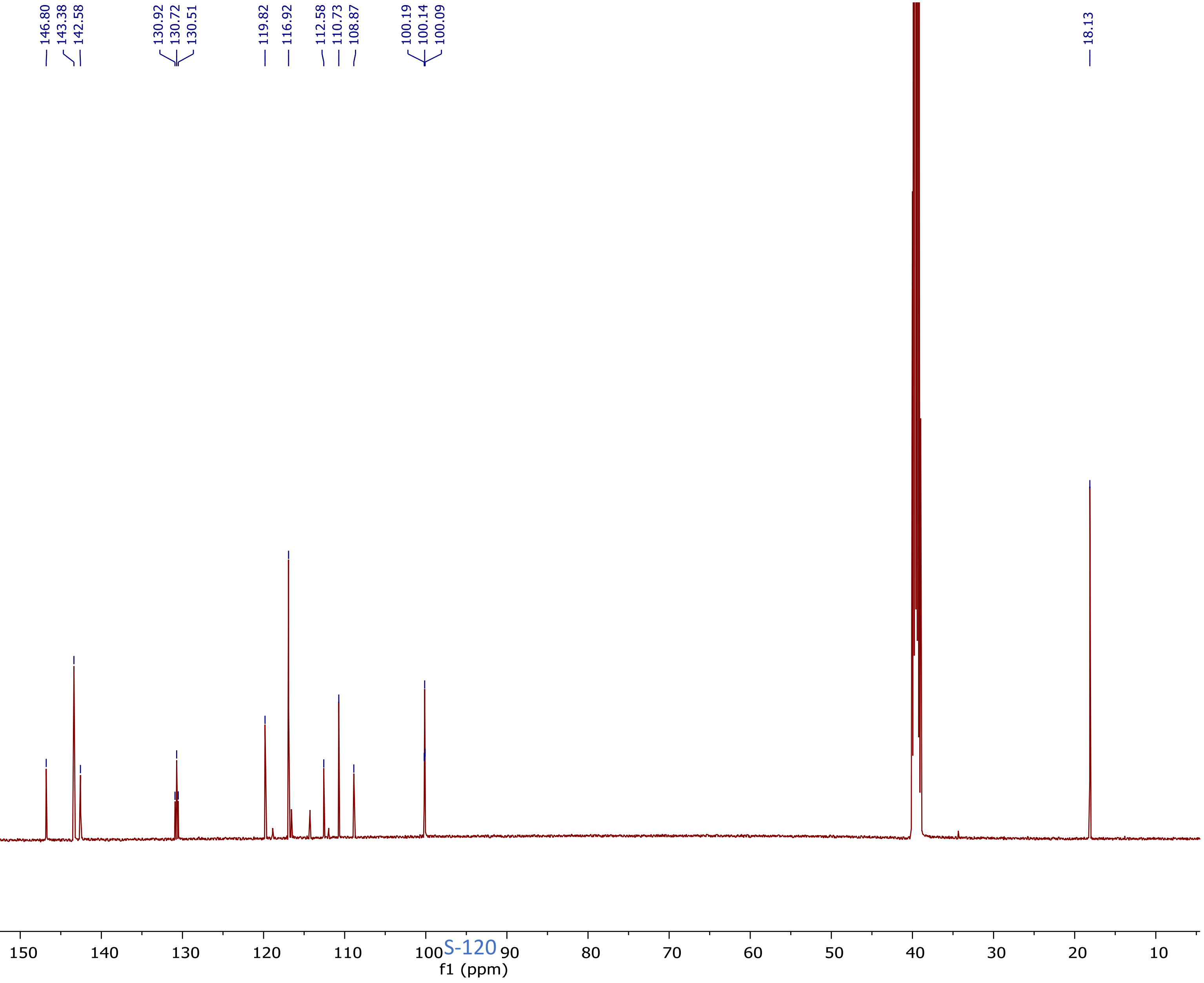
146.80
143.38
142.58
130.92
130.72
130.51
119.82
116.92
112.58
110.73
108.87
100.19
100.14
100.09
18.13
180
170
160
150
140
130
120
110
100
90
80
70
60
50
40
30
20
10
f1 (ppm)

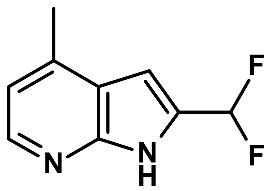

8a

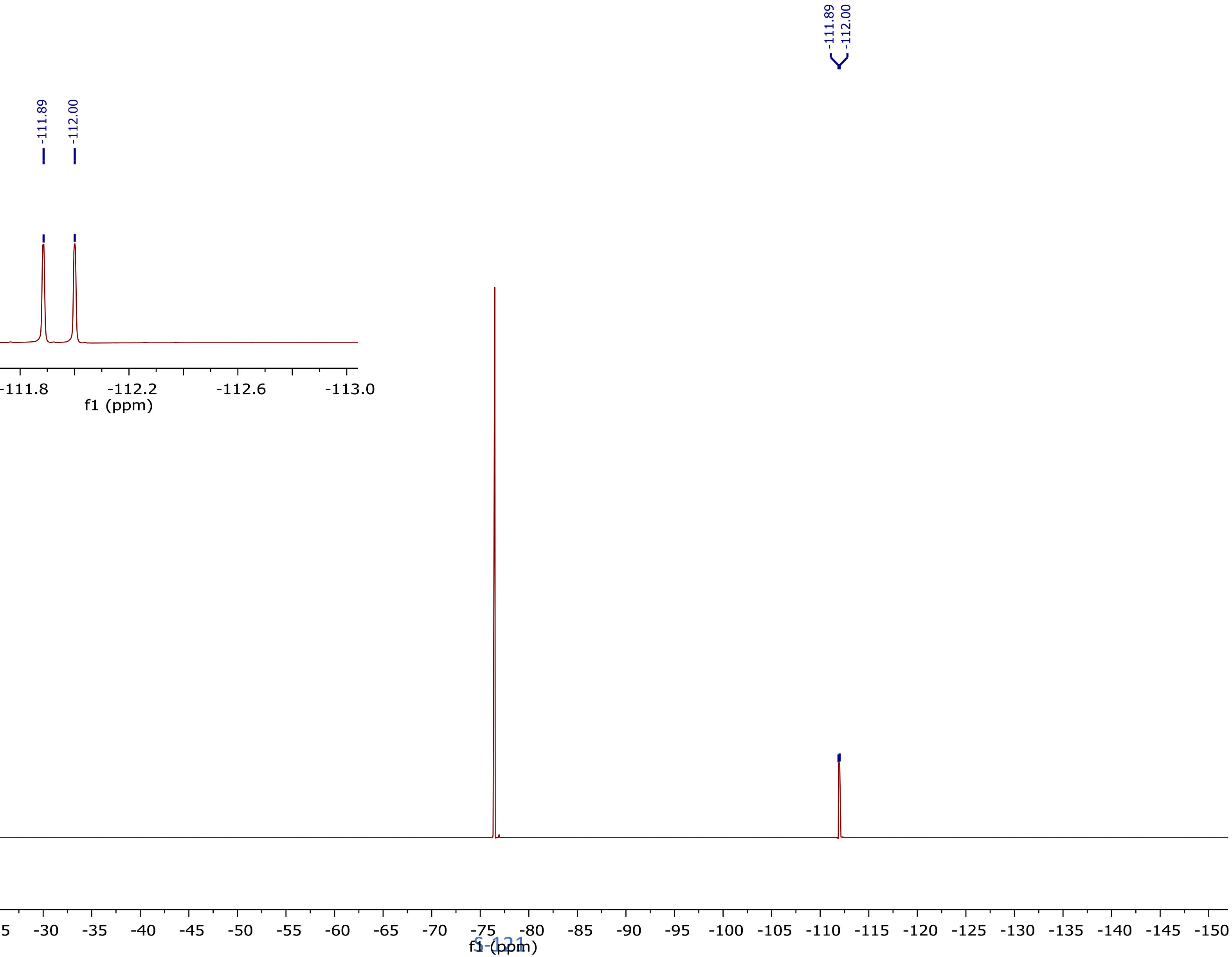
-111.89
-112.00
-111.4
-111.8
-112.2
-112.6
-113.0
f1 (ppm)
-5
-10
-15
-20
-25
-30
-35
-40
-45
-50
-55
-60
-65
-70
-75
-80
-85
-90
-95
-100
-105
-110
-115
-120
-125
-130
-135
-140
-145
-150
f1 (ppm)

j. ^{1}H, cosy, ^{13}C and ^{19}F NMR of 8b

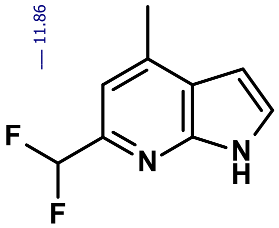

8b

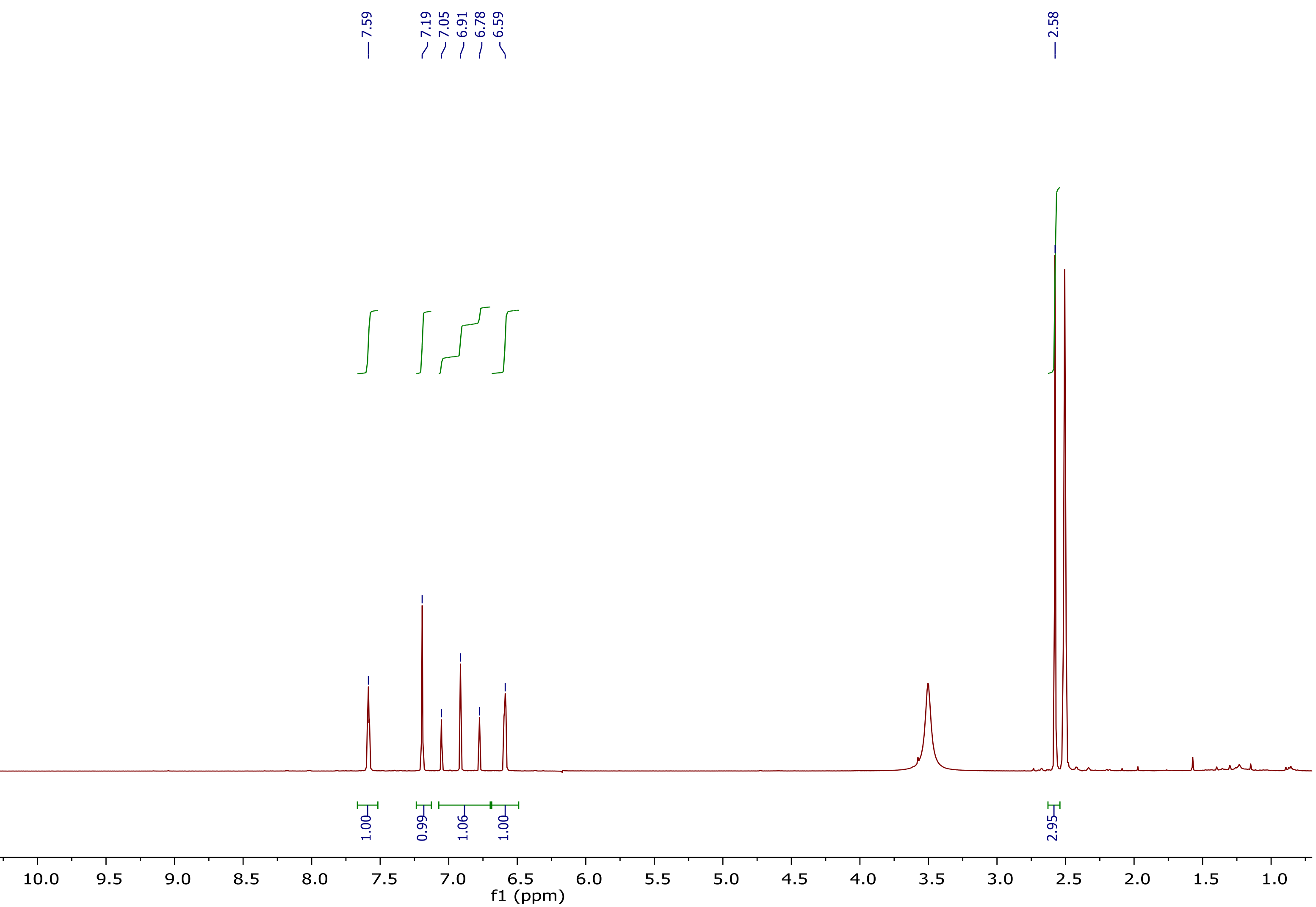
11.86
7.59
7.19
7.05
6.91
6.78
6.59
2.58
0.94
1.00
0.99
1.06
1.00
2.95
12.0 11.5 11.0 10.5 10.0 9.5 9.0 8.5 8.0 7.5 7.0 6.5 6.0 5.5 5.0 4.5 4.0 3.5 3.0 2.5 2.0 1.5 1.0
f1 (ppm)

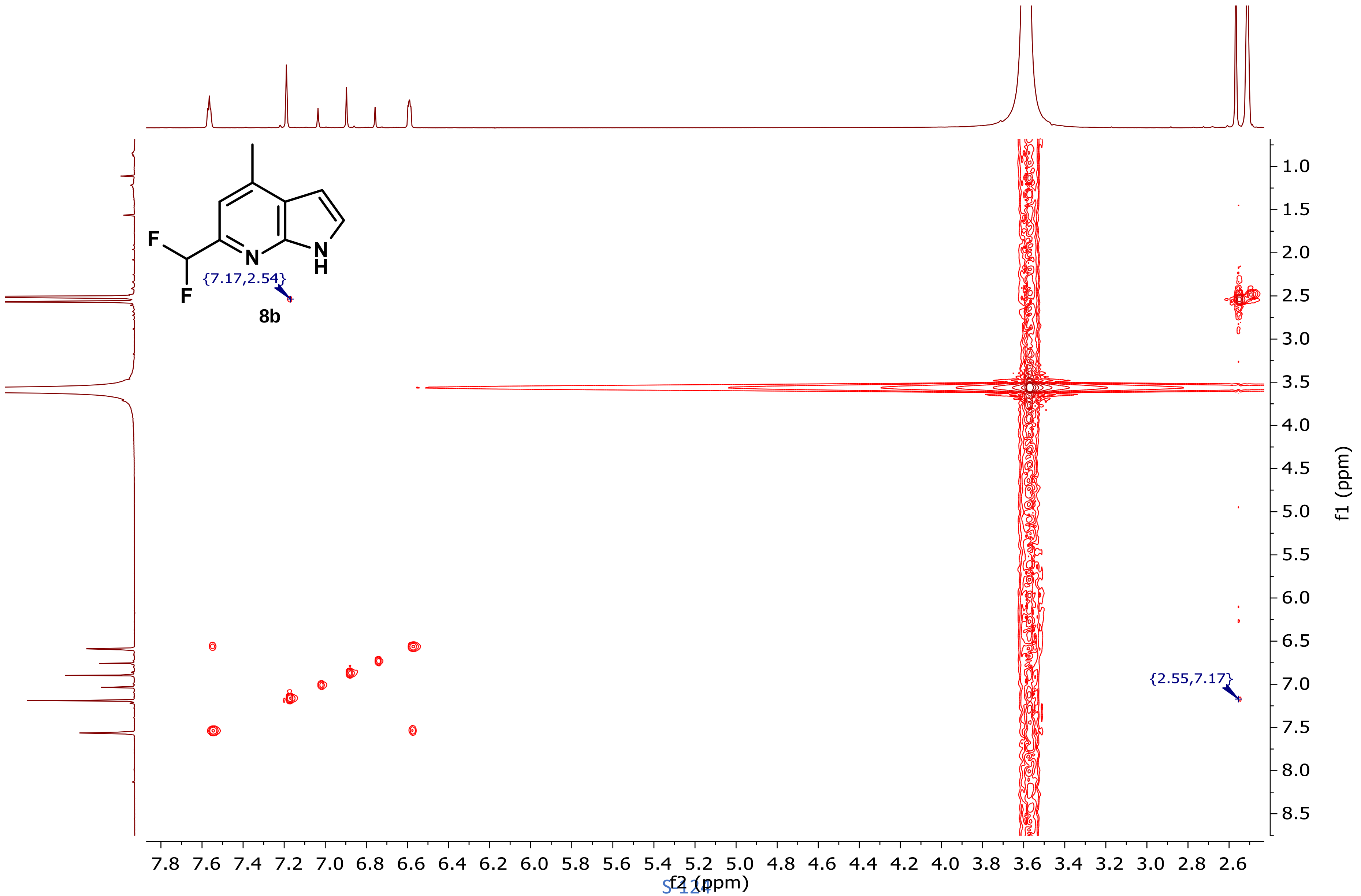
F
F
N
N
H
8b
{7.17,2.54}
{2.55,7.17}
7.8 7.6 7.4 7.2 7.0 6.8 6.6 6.4 6.2 6.0 5.8 5.6 5.4 5.2 5.0 4.8 4.6 4.4 4.2 4.0 3.8 3.6 3.4 3.2 3.0 2.8 2.6
f2 (ppm)
1.0 1.5 2.0 2.5 3.0 3.5 4.0 4.5 5.0 5.5 6.0 6.5 7.0 7.5 8.0 8.5
f1 (ppm)

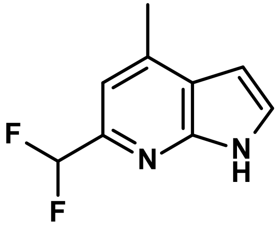

8b

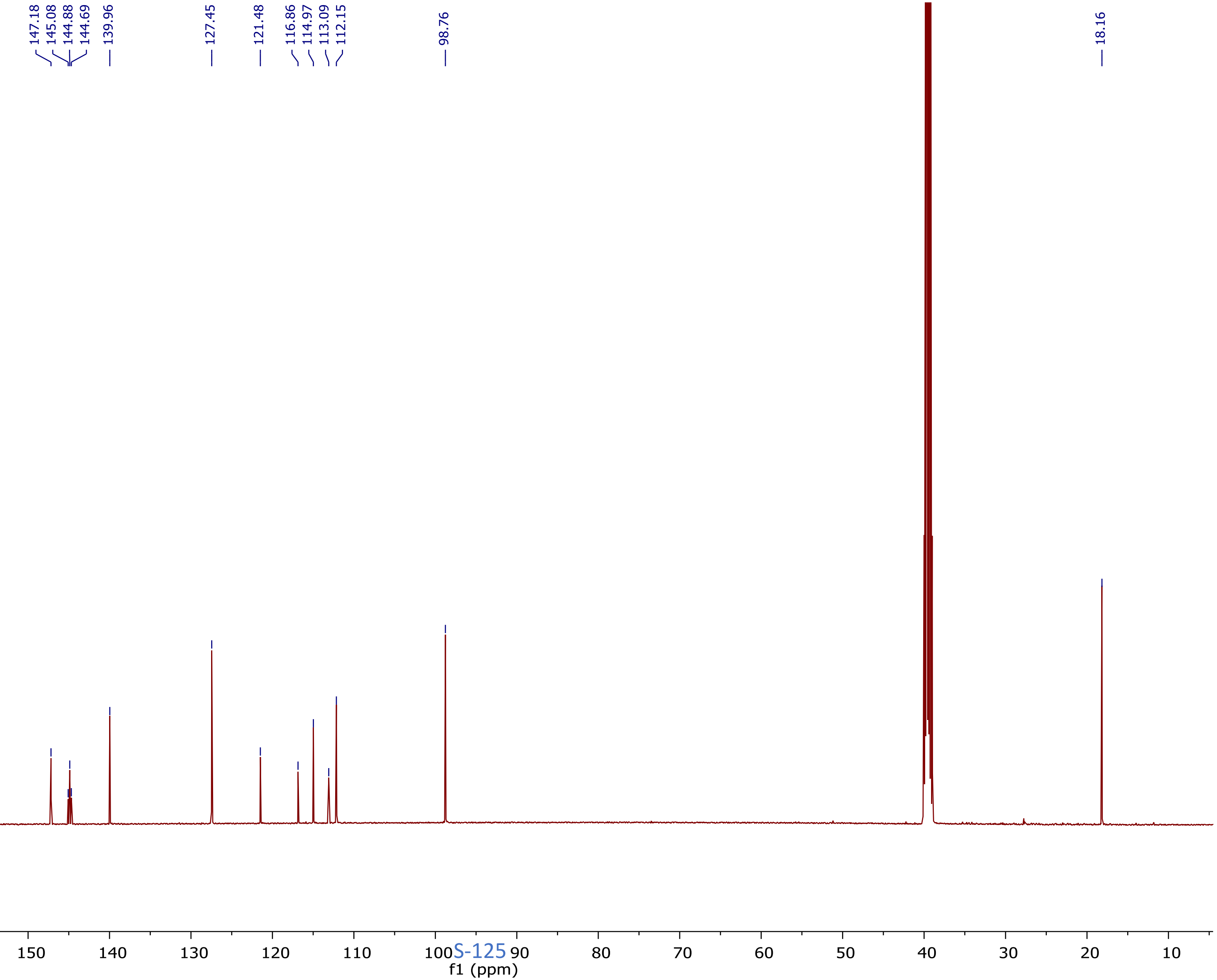
147.18
145.08
144.88
144.69
139.96
127.45
121.48
116.86
114.97
113.09
112.15
98.76
18.16
180
170
160
150
140
130
120
110
100
90
80
70
60
50
40
30
20
10
f1 (ppm)

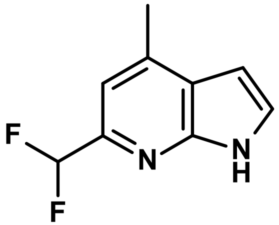
F
F
N
N
H

8b

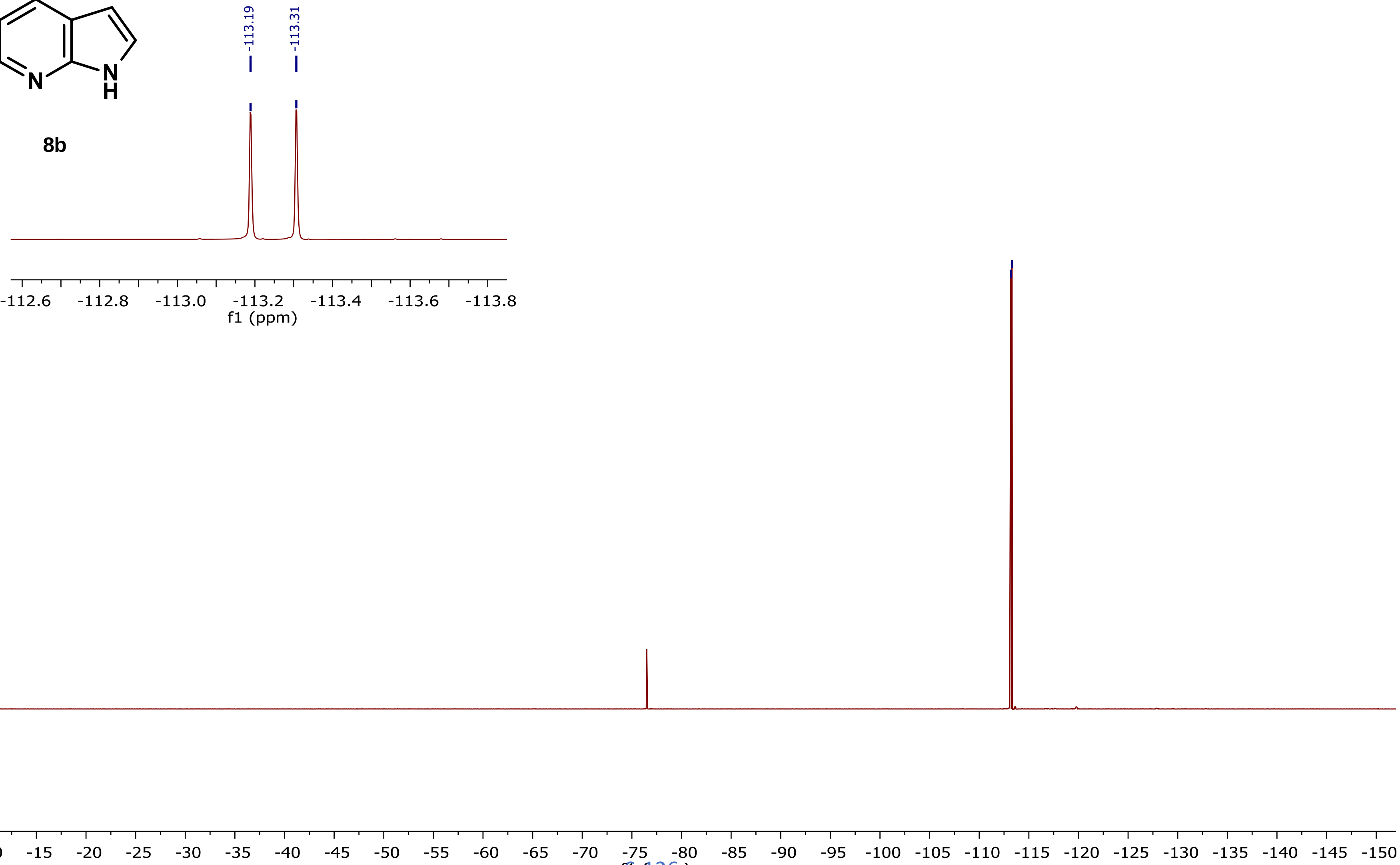
-113.19
-113.31
-112.6 -112.8 -113.0 -113.2 -113.4 -113.6 -113.8
f1 (ppm)
-5 -10 -15 -20 -25 -30 -35 -40 -45 -50 -55 -60 -65 -70 -75 -80 -85 -90 -95 -100 -105 -110 -115 -120 -125 -130 -135 -140 -145 -150
f1 (ppm)

k. ^{1}H, ^{13}C and ^{19}F NMR of 9a

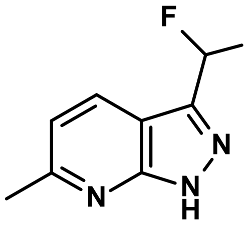

9a

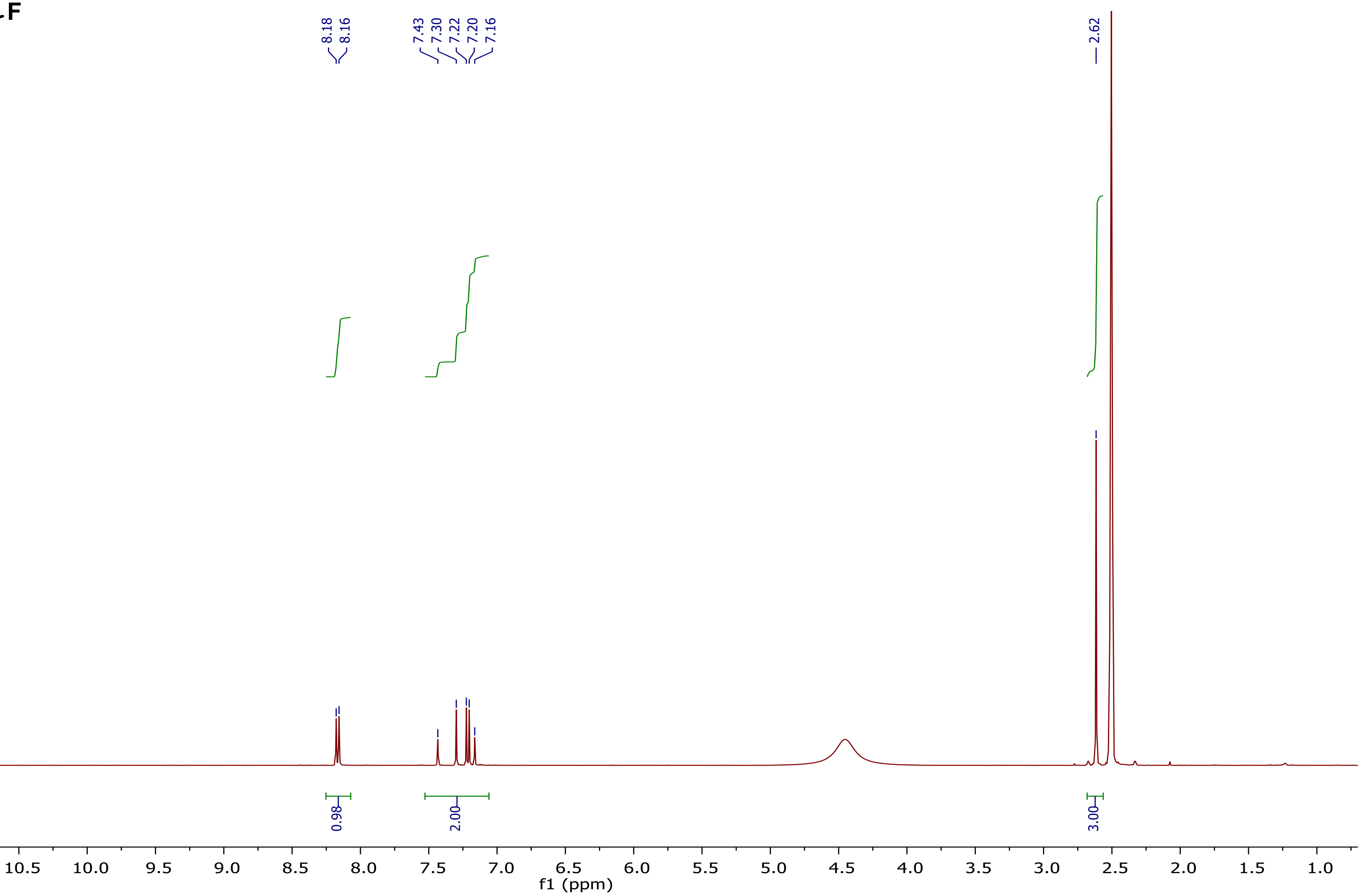

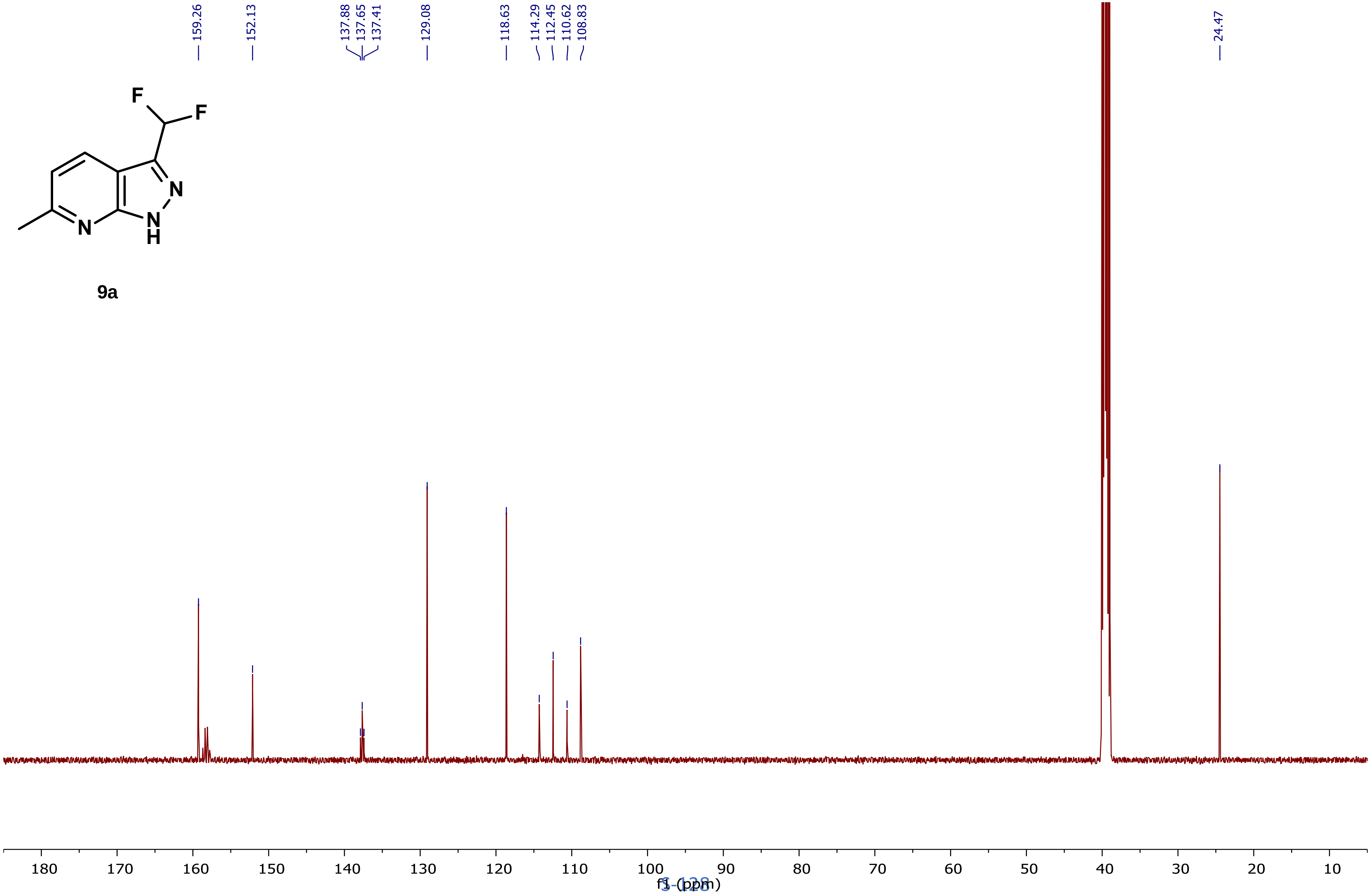
F
F
N
N
H
N
9a
159.26
152.13
137.88
137.65
137.41
129.08
118.63
114.29
112.45
110.62
108.83
24.47
180
170
160
150
140
130
120
110
100
90
80
70
60
50
40
30
20
10
f1 (ppm)

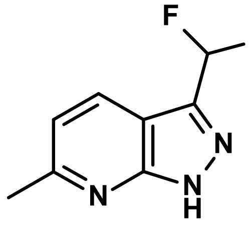
F
F
N
N
H
N

9a

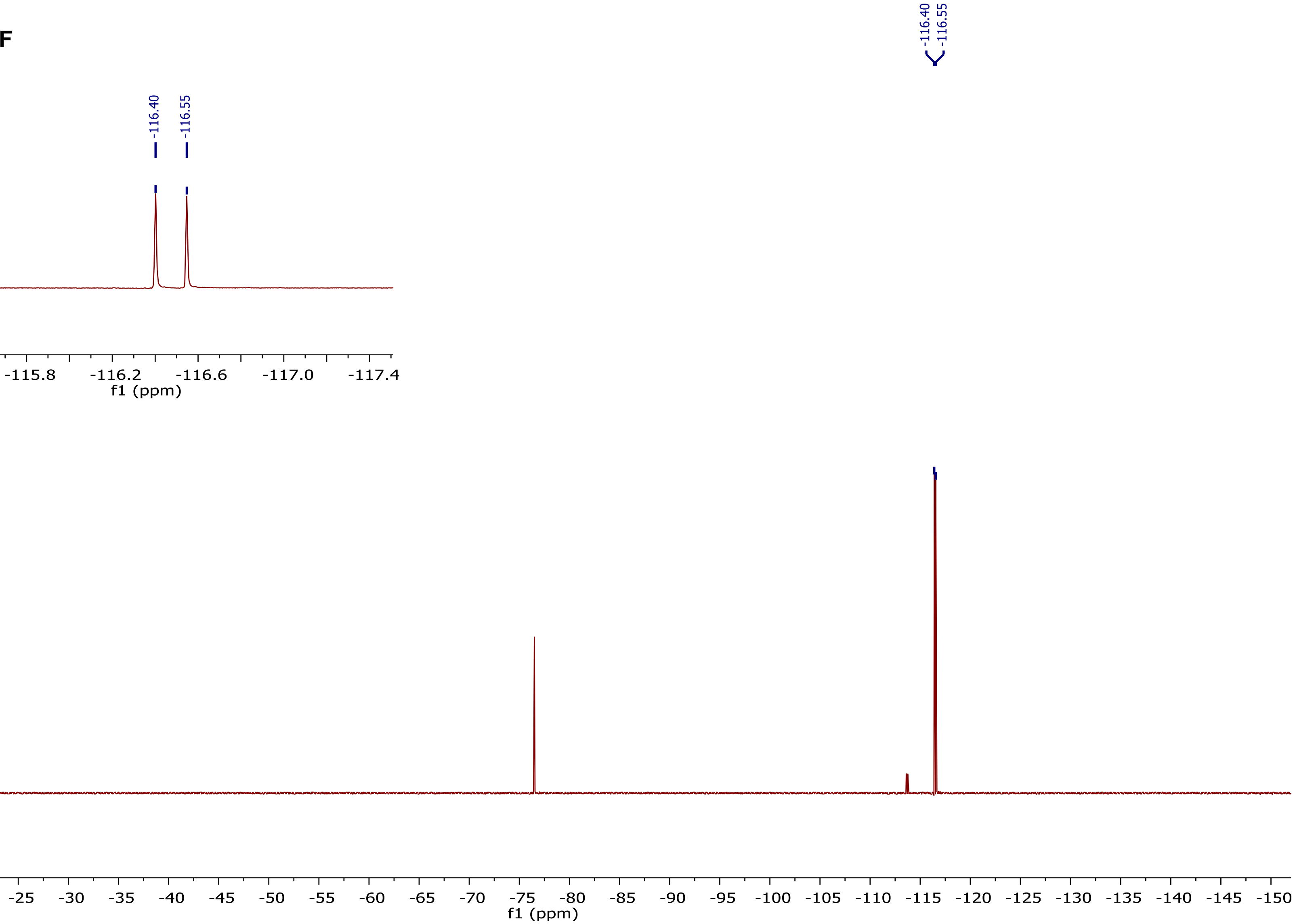
-116.40
-116.55
-115.4
-115.8
-116.2
-116.6
-117.0
-117.4
f1 (ppm)
-5
-10
-15
-20
-25
-30
-35
-40
-45
-50
-55
-60
-65
-70
-75
-80
-85
-90
-95
-100
-105
-110
-115
-120
-125
-130
-135
-140
-145
-150
f1 (ppm)

I. ^{1}H, ^{13}C and ^{19}F NMR of 9b

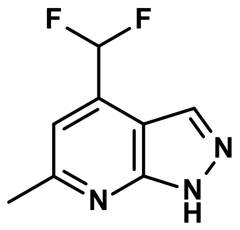

9b

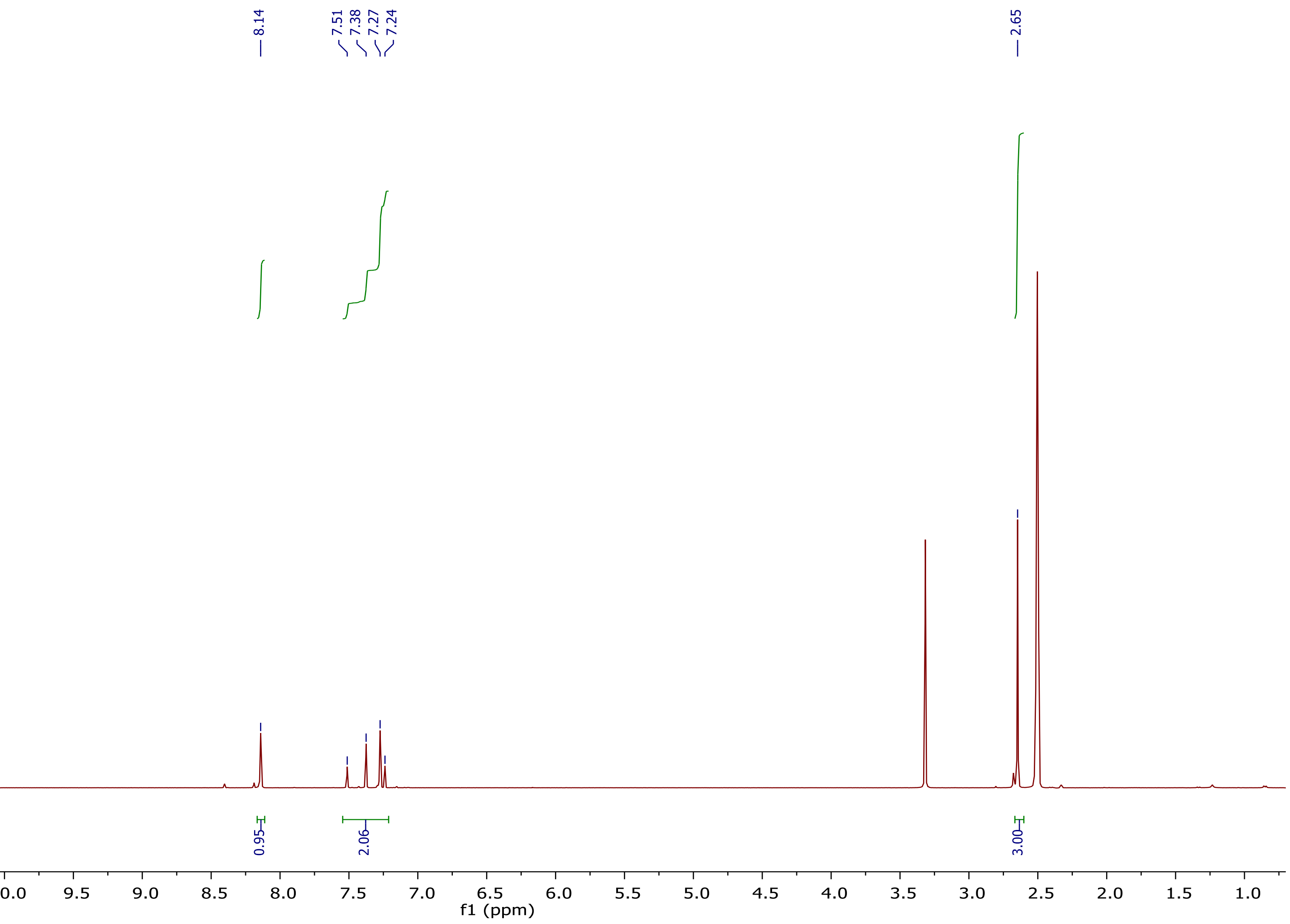
8.14
7.51
7.38
7.27
7.24
2.65
0.95
2.06
3.00
12.0
11.5
11.0
10.5
10.0
9.5
9.0
8.5
8.0
7.5
7.0
6.5
6.0
5.5
5.0
4.5
4.0
3.5
3.0
2.5
2.0
1.5
1.0
f1 (ppm)

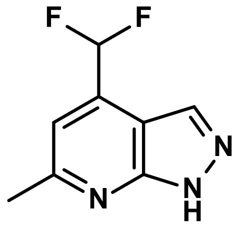

9b

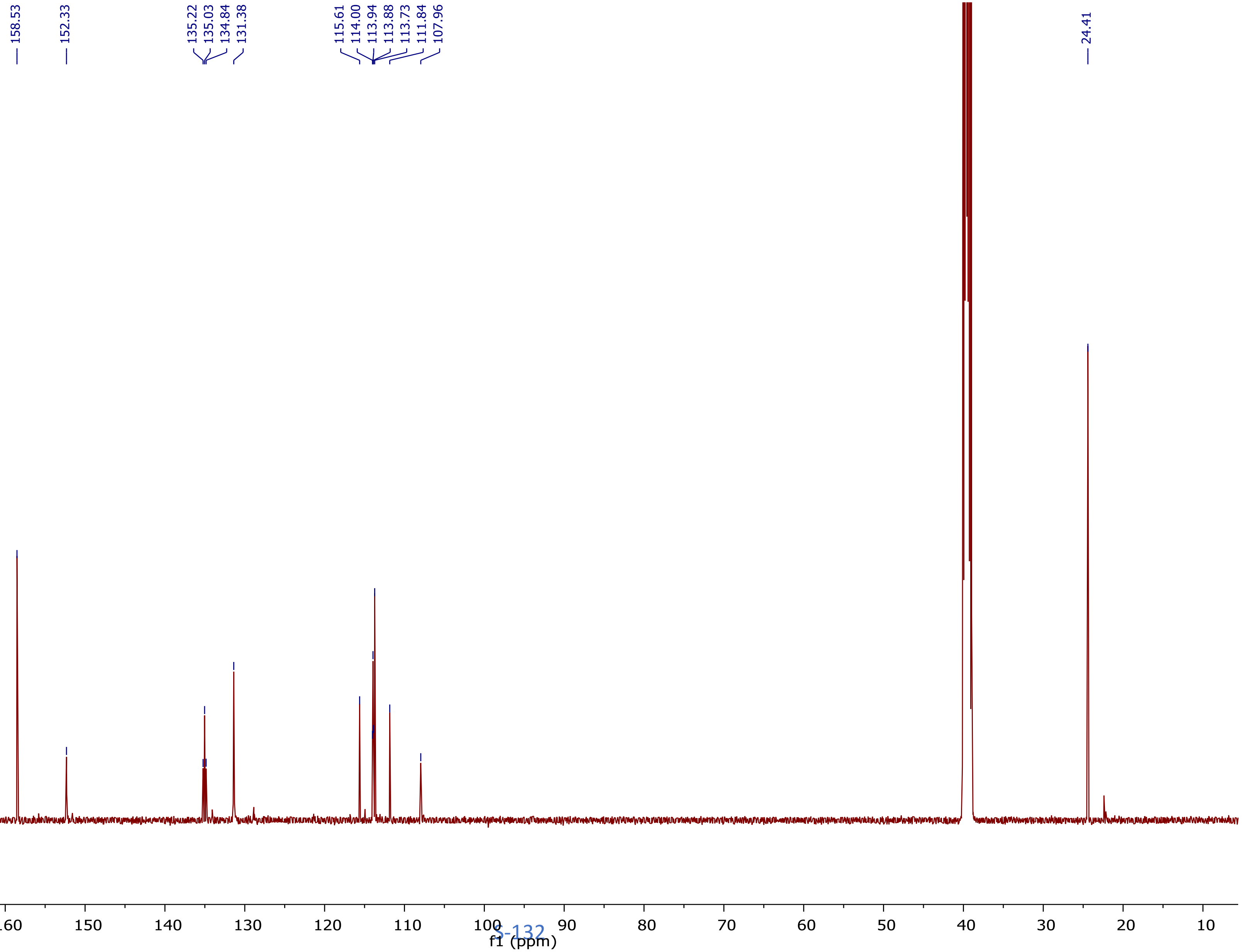
158.53
152.33
135.22
135.03
134.84
131.38
115.61
114.00
113.94
113.88
113.73
111.84
107.96
24.41
180
170
160
150
140
130
120
110
100
90
80
70
60
50
40
30
20
10
f1 (ppm)

9b

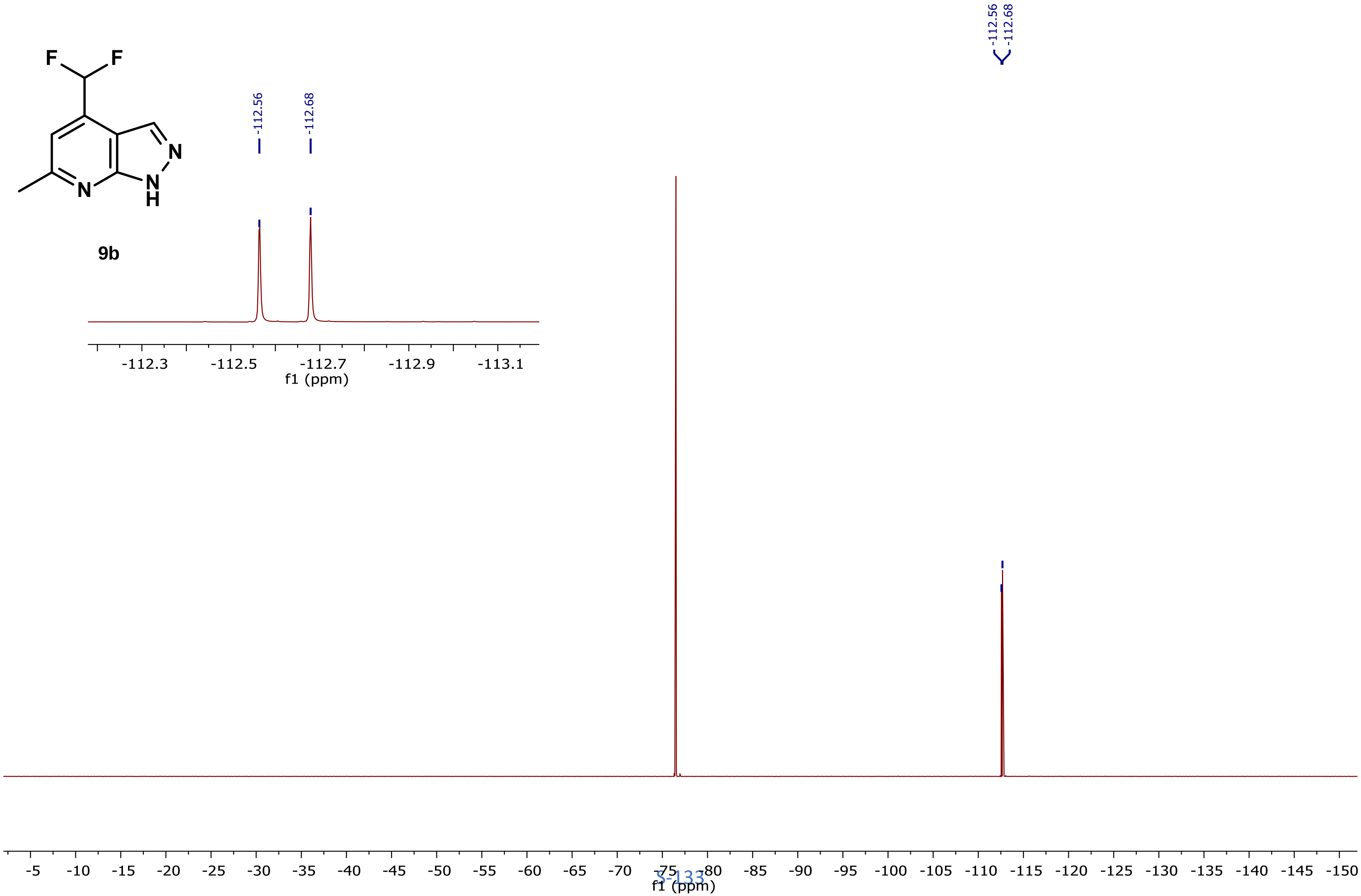
-112.56
-112.68
-112.3
-112.5
-112.7
-112.9
-113.1
f1 (ppm)
-5 -10 -15 -20 -25 -30 -35 -40 -45 -50 -55 -60 -65 -70 -75 -80 -85 -90 -95 -100 -105 -110 -115 -120 -125 -130 -135 -140 -145 -150
f1 (ppm)

m. ^{1}H, ^{13}C and ^{19}F NMR of 10

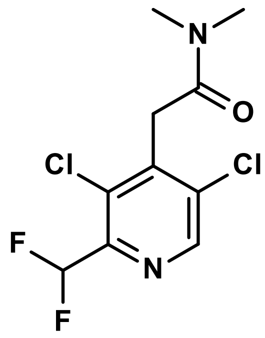

10

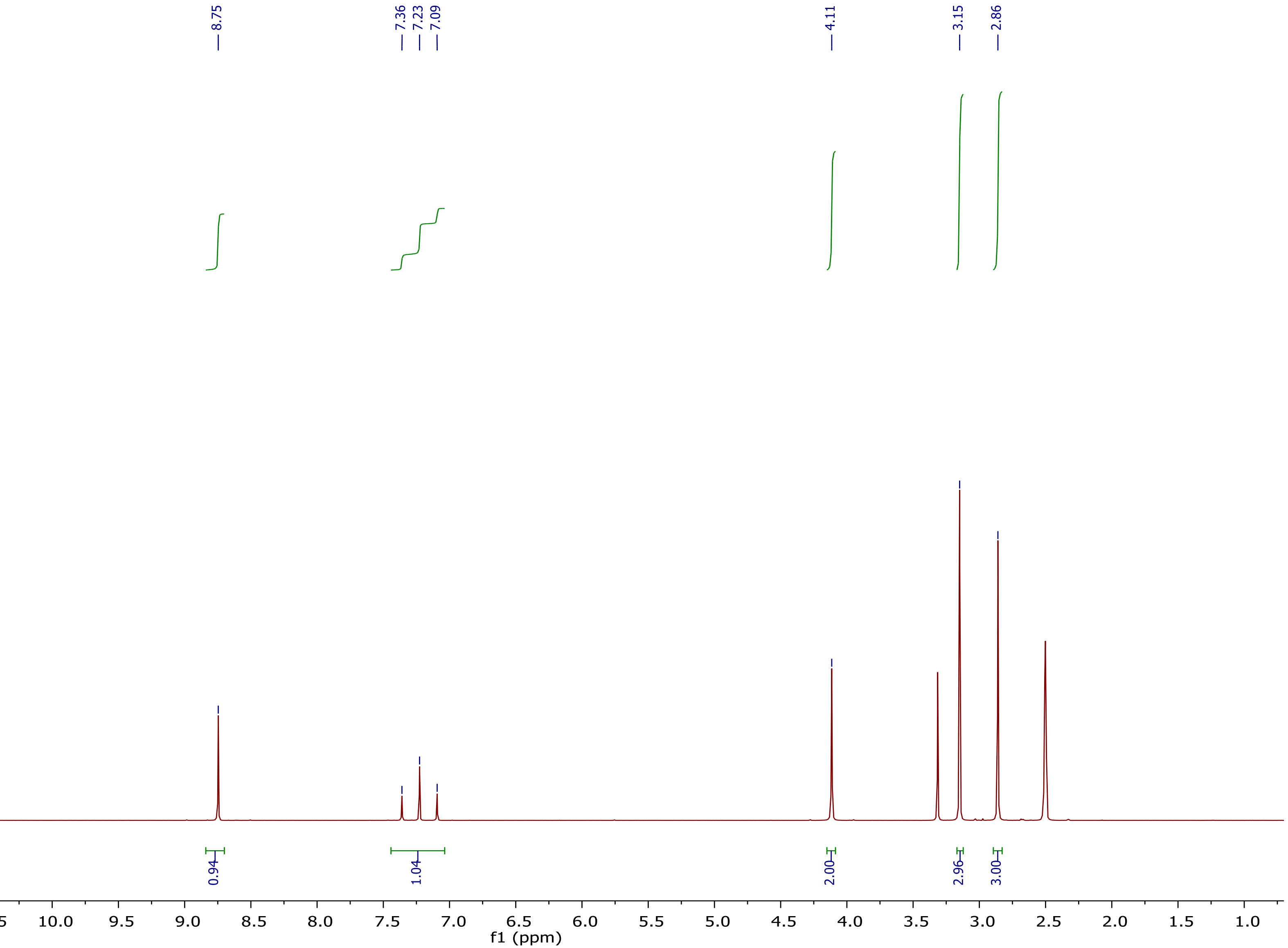
8.75
7.36
7.23
7.09
4.11
3.15
2.86
0.94
1.04
2.00
2.96
3.00
12.0
11.5
11.0
10.5
10.0
9.5
9.0
8.5
8.0
7.5
7.0
6.5
6.0
5.5
5.0
4.5
4.0
3.5
3.0
2.5
2.0
1.5
1.0
f1 (ppm)

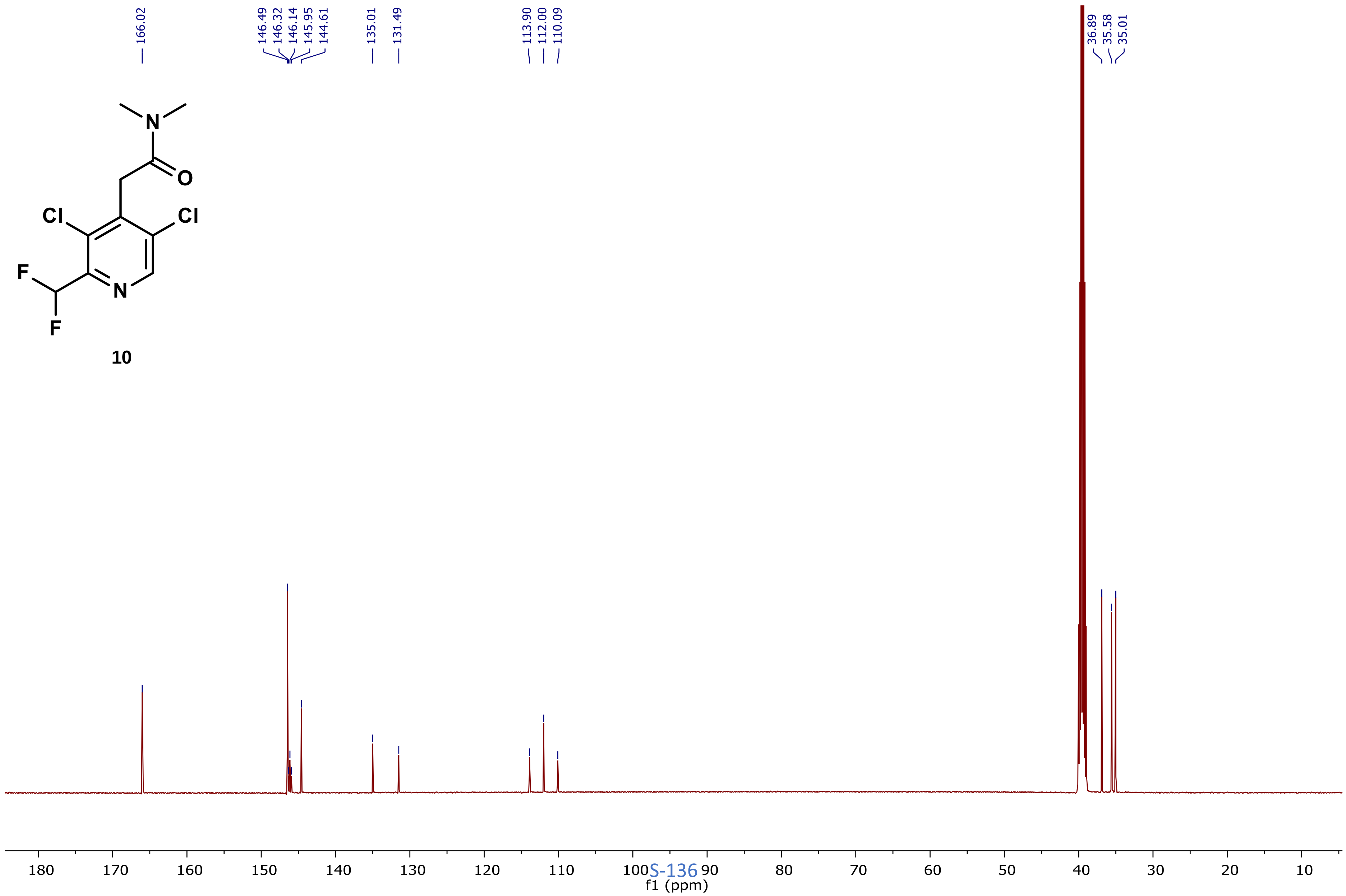

166.02
146.49
146.32
146.14
145.95
144.61
135.01
131.49
113.90
112.00
110.09
36.89
35.58
35.01
180
170
160
150
140
130
120
110
100
90
80
70
60
50
40
30
20
10
f1 (ppm)

10

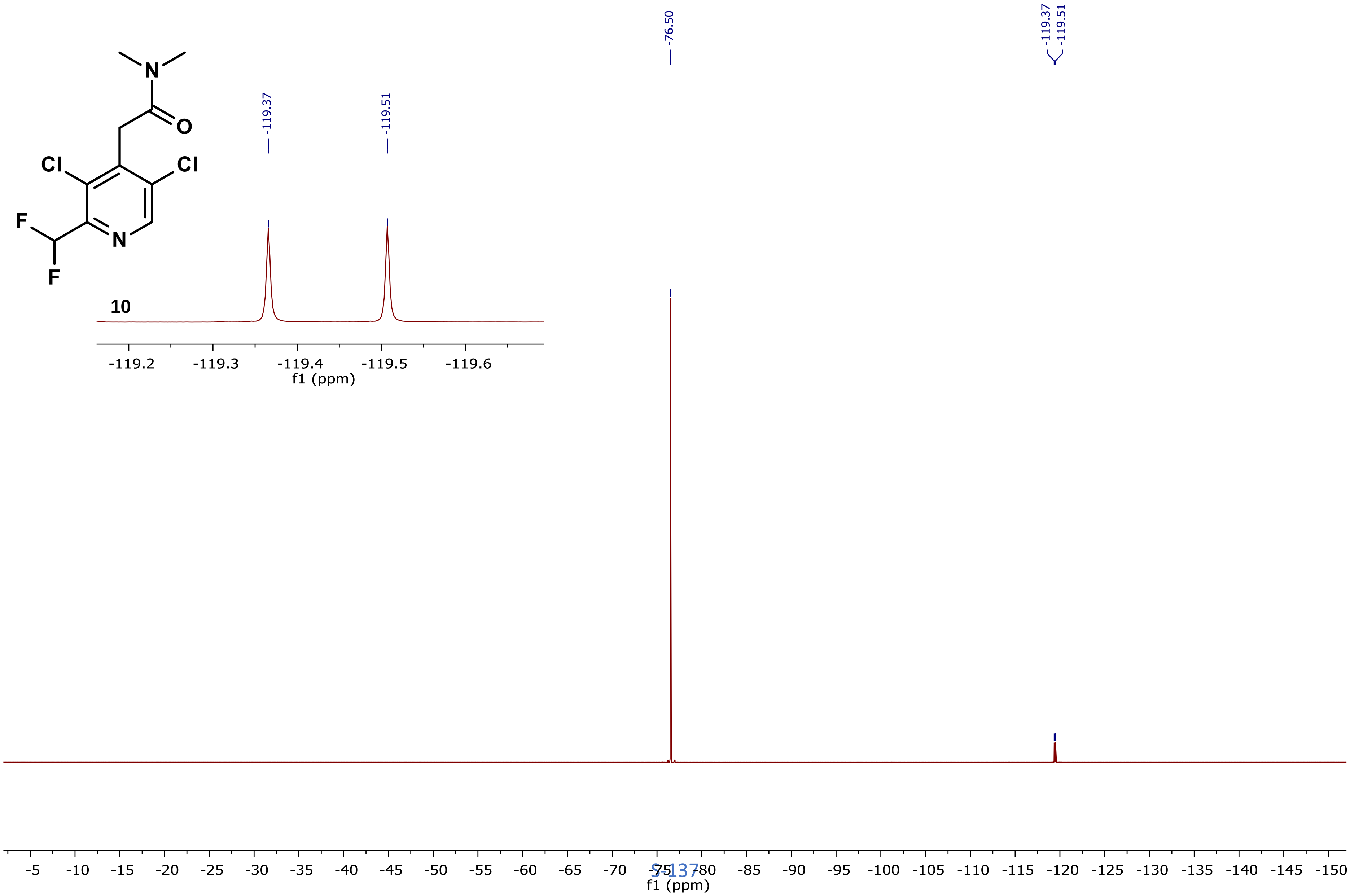
-76.50
-119.37
-119.51
Cl
Cl
F
F
N
N
O
10
-119.2
-119.3
-119.4
-119.5
-119.6
f1 (ppm)
-5
-10
-15
-20
-25
-30
-35
-40
-45
-50
-55
-60
-65
-70
-75
-80
-85
-90
-95
-100
-105
-110
-115
-120
-125
-130
-135
-140
-145
-150
f1 (ppm)

n. ^{1}H, ^{13}C and ^{19}F NMR of 11

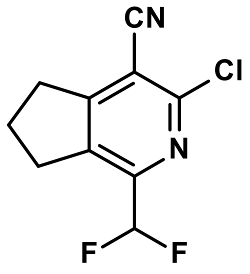
CN
Cl
N
F
F

11

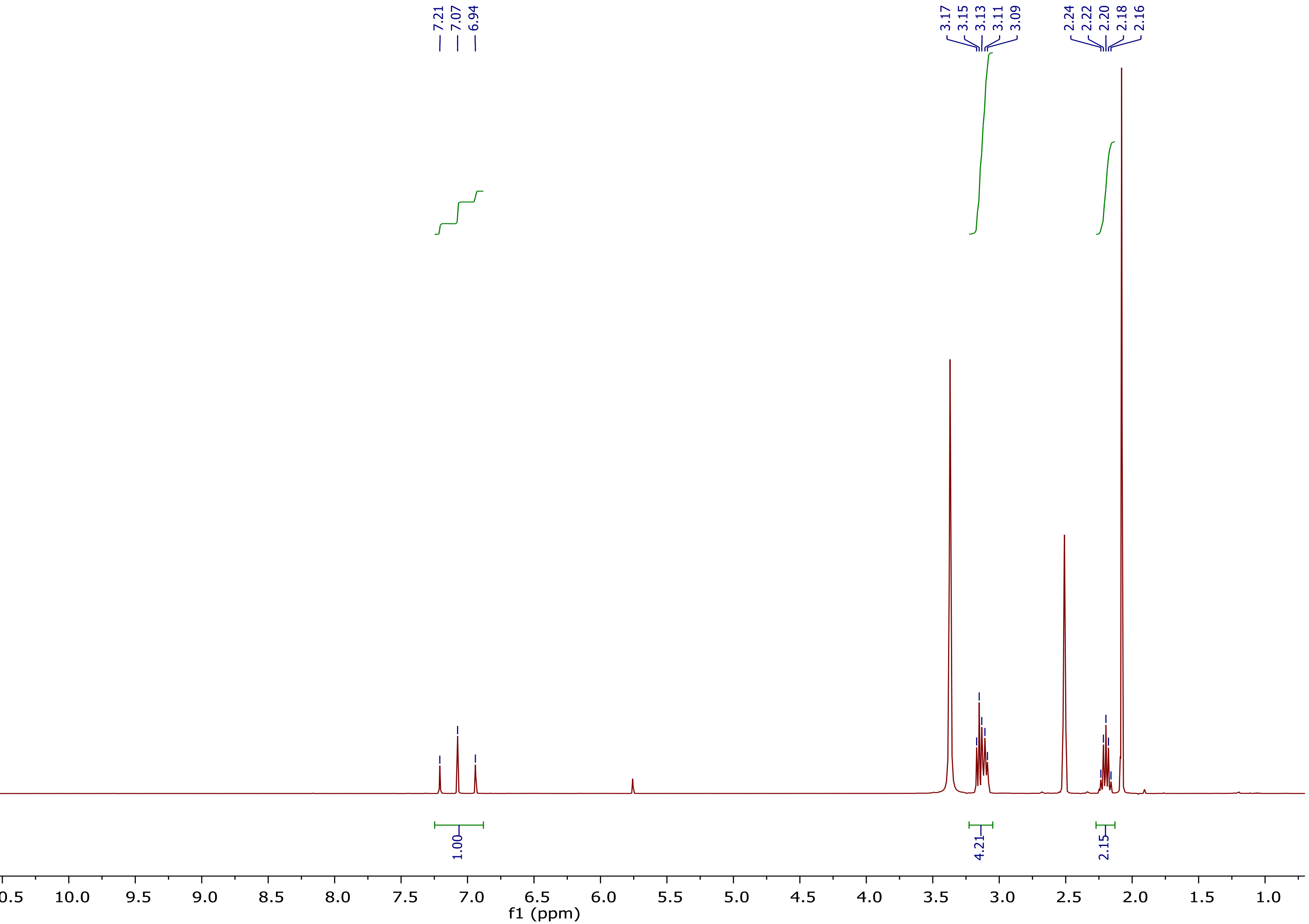
7.21
7.07
6.94
3.17
3.15
3.13
3.11
3.09
2.24
2.22
2.20
2.18
2.16
1.00
4.21
2.15
12.0 11.5 11.0 10.5 10.0 9.5 9.0 8.5 8.0 7.5 7.0 6.5 6.0 5.5 5.0 4.5 4.0 3.5 3.0 2.5 2.0 1.5 1.0
f1 (ppm)

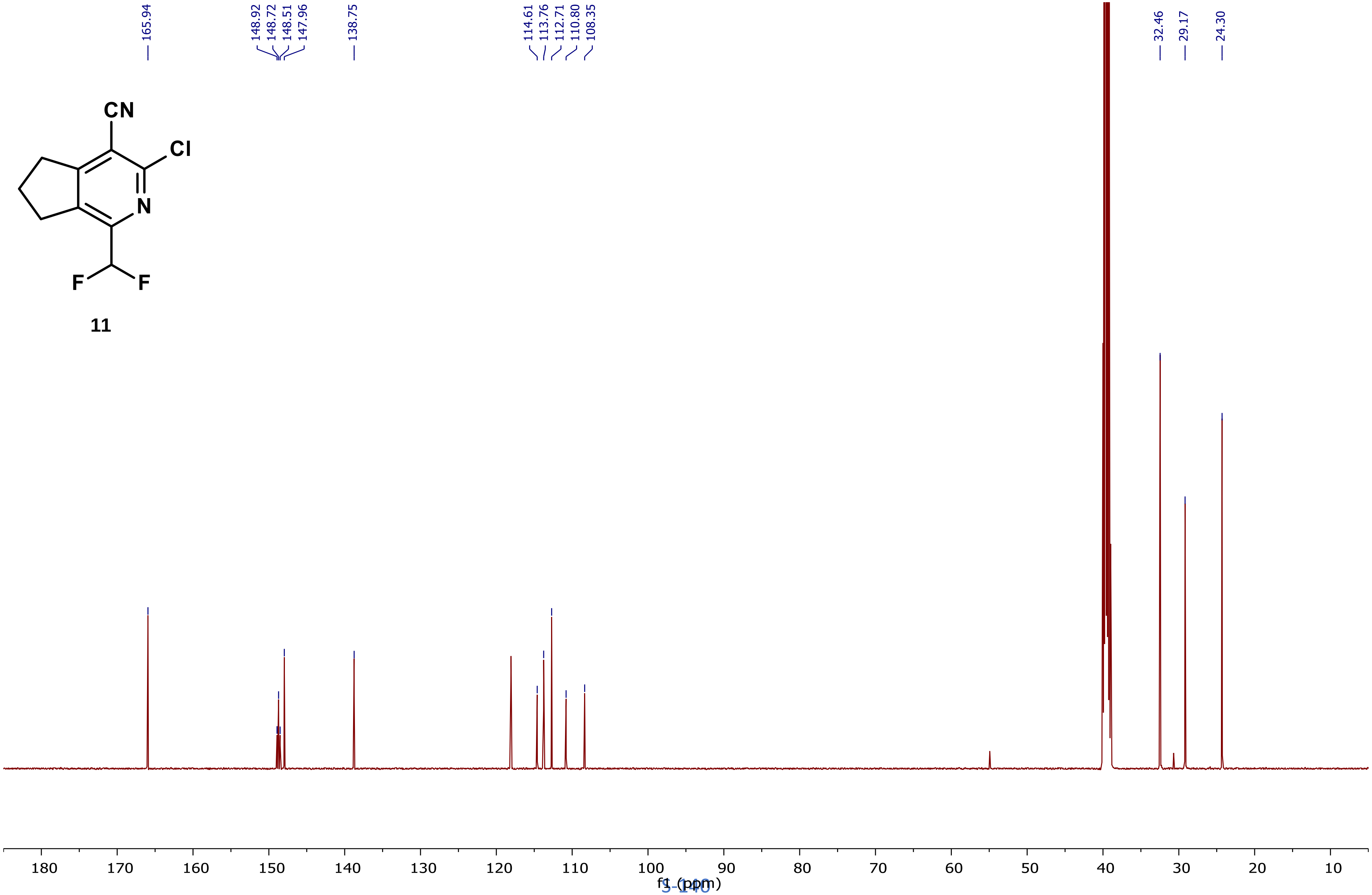
CN
Cl
N
F
F
11
165.94
148.92
148.72
148.51
147.96
138.75
114.61
113.76
112.71
110.80
108.35
32.46
29.17
24.30
180 170 160 150 140 130 120 110 100 90 80 70 60 50 40 30 20 10
f1 (ppm)

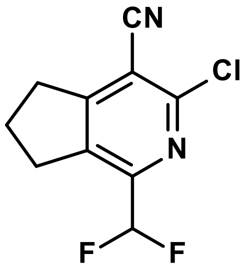

11

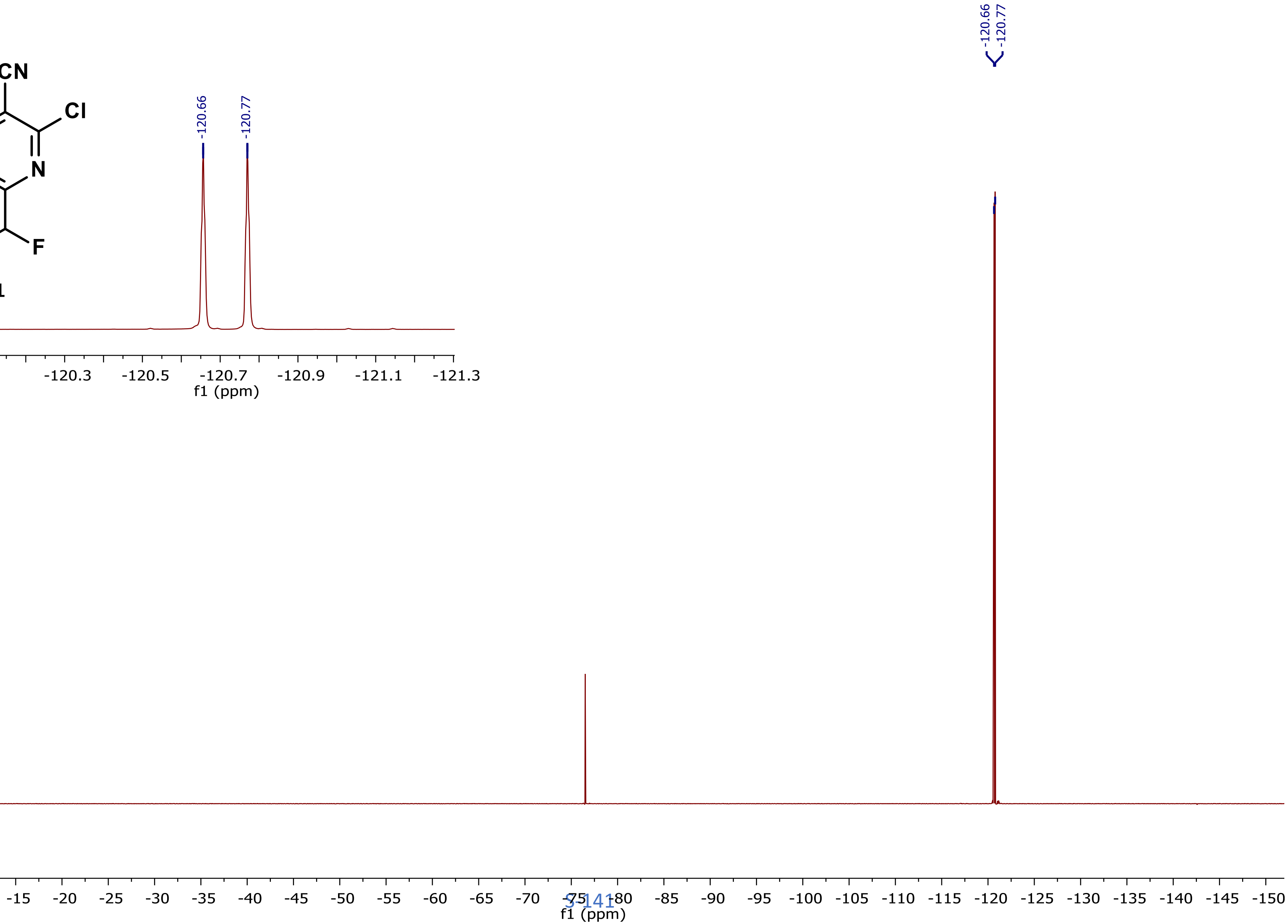
-120.66
-120.77
-120.3 -120.5 -120.7 -120.9 -121.1 -121.3
f1 (ppm)
-5 -10 -15 -20 -25 -30 -35 -40 -45 -50 -55 -60 -65 -70 -75 -80 -85 -90 -95 -100 -105 -110 -115 -120 -125 -130 -135 -140 -145 -150
f1 (ppm)

o. ^{1}H, ^{13}C and ^{19}F NMR of 12

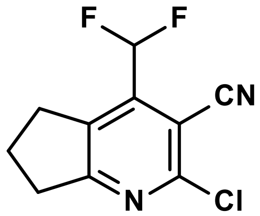

12

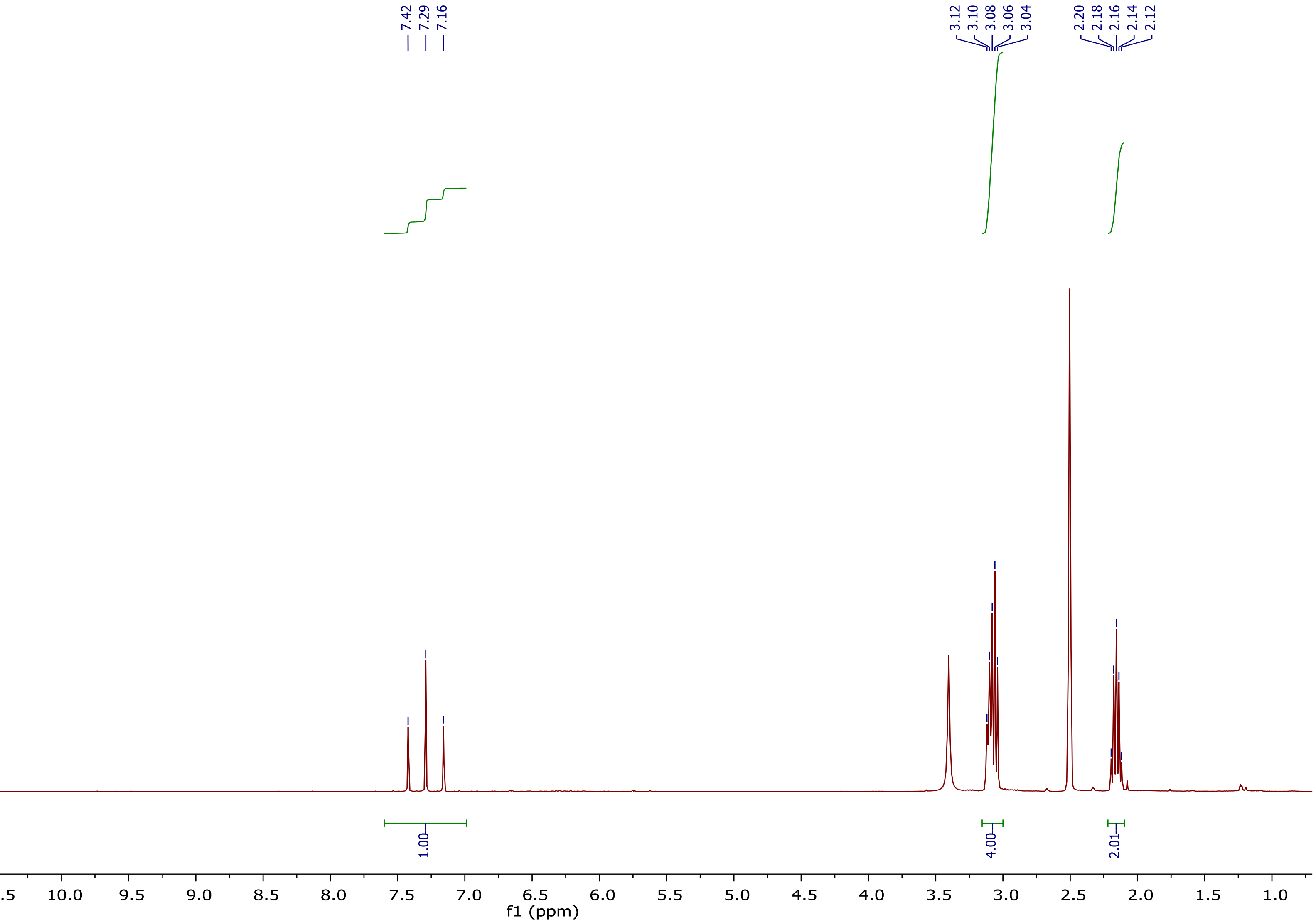
7.42
7.29
7.16
3.12
3.10
3.08
3.06
3.04
2.20
2.18
2.16
2.14
2.12
1.00
4.00
2.01
12.0
11.5
11.0
10.5
10.0
9.5
9.0
8.5
8.0
7.5
7.0
6.5
6.0
5.5
5.0
4.5
4.0
3.5
3.0
2.5
2.0
1.5
1.0
f1 (ppm)

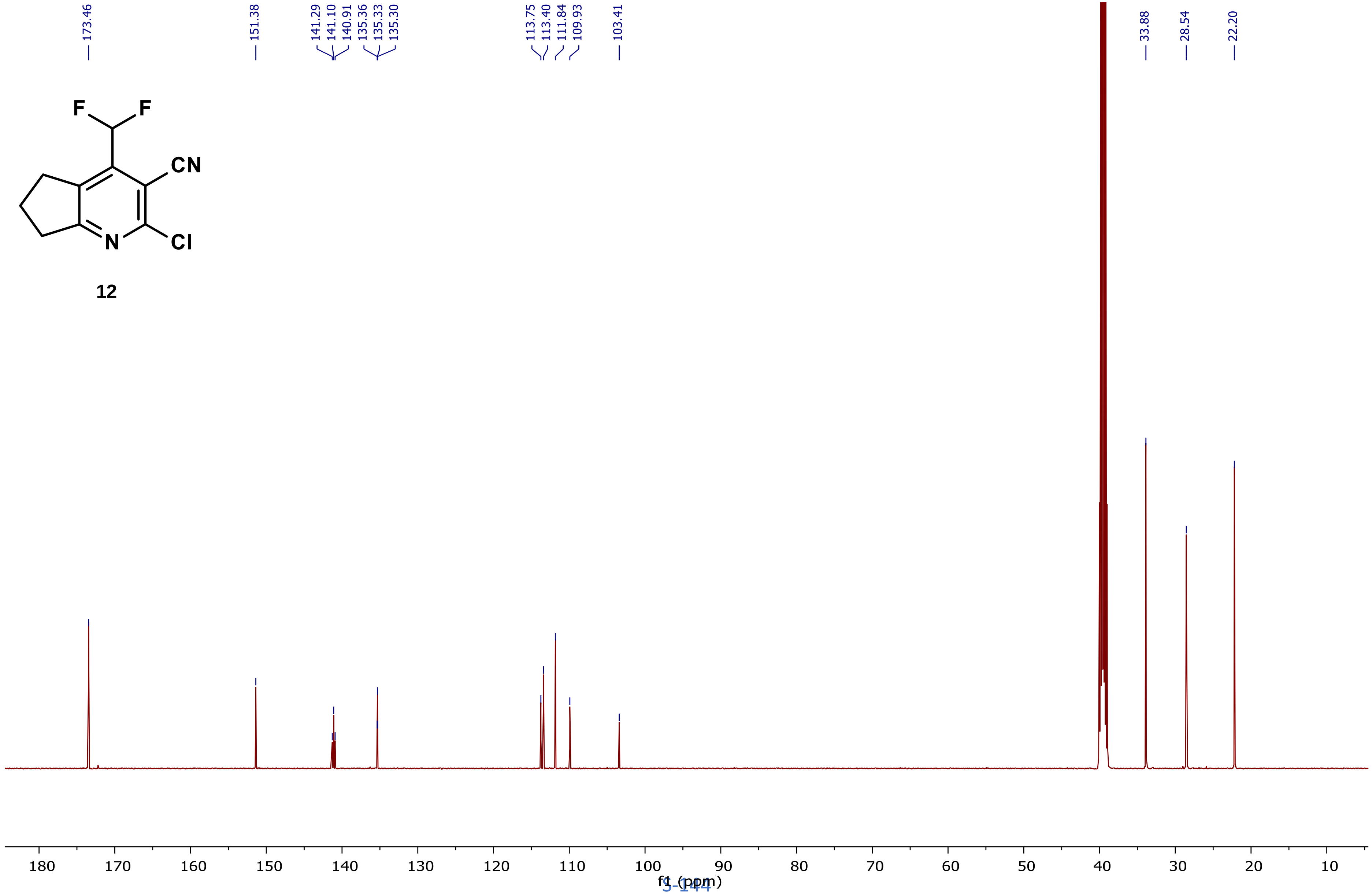
173.46
151.38
141.29
141.10
140.91
135.36
135.33
135.30
113.75
113.40
111.84
109.93
103.41
33.88
28.54
22.20
F
F
CN
N
Cl
12
180
170
160
150
140
130
120
110
100
90
80
70
60
50
40
30
20
10
f1 (ppm)

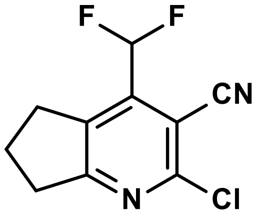
F
F
CN
N
Cl

12

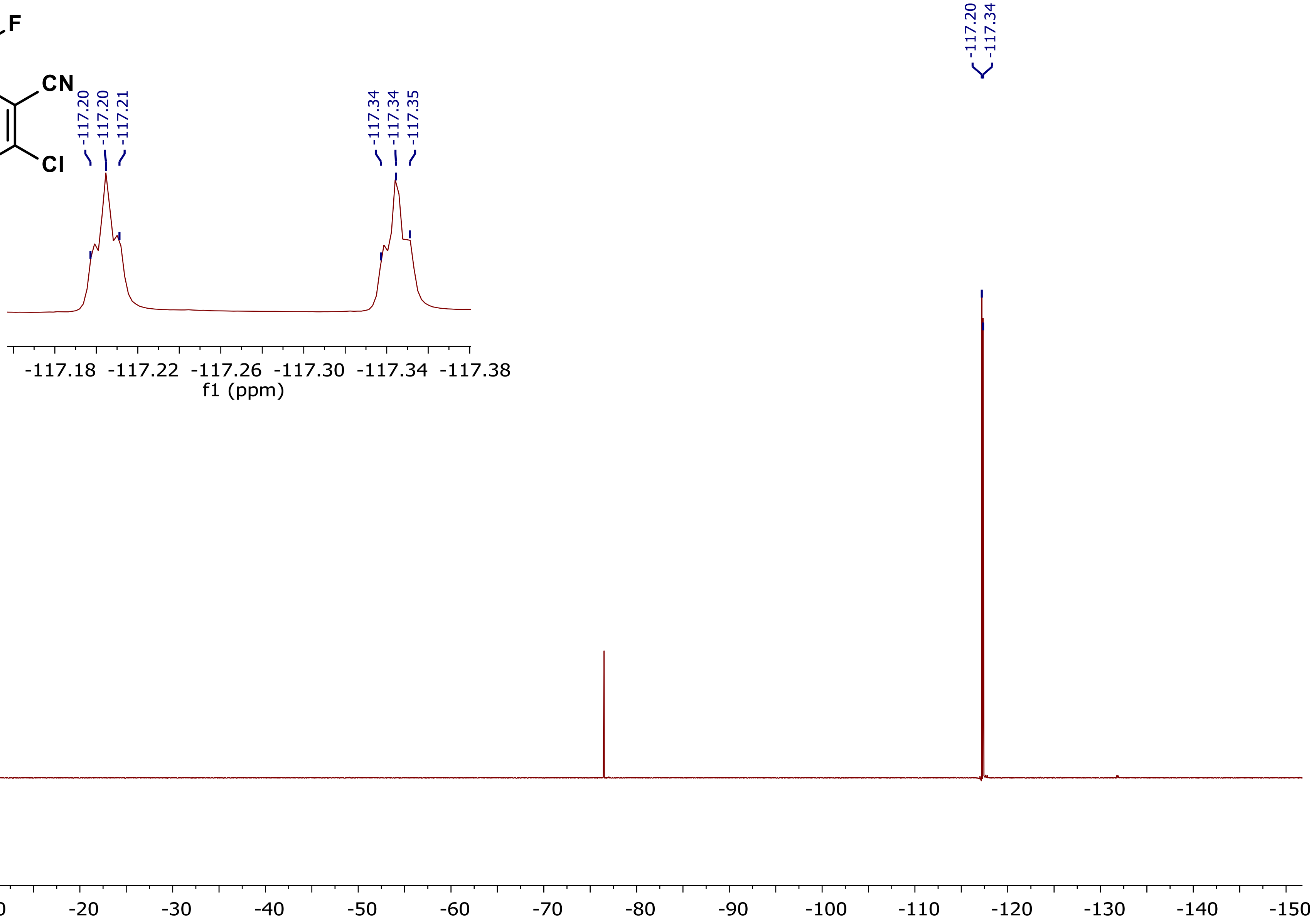
-117.20
-117.20
-117.21
-117.34
-117.34
-117.35
-117.18 -117.22 -117.26 -117.30 -117.34 -117.38
f1 (ppm)
-117.20
-117.34
-10 -20 -30 -40 -50 -60 -70 -80 -90 -100 -110 -120 -130 -140 -150
f1 (ppm)

p. ^{1}H, ^{13}C and ^{19}F NMR of 13

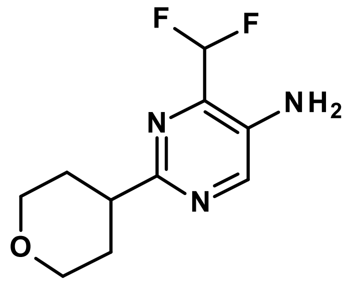

13

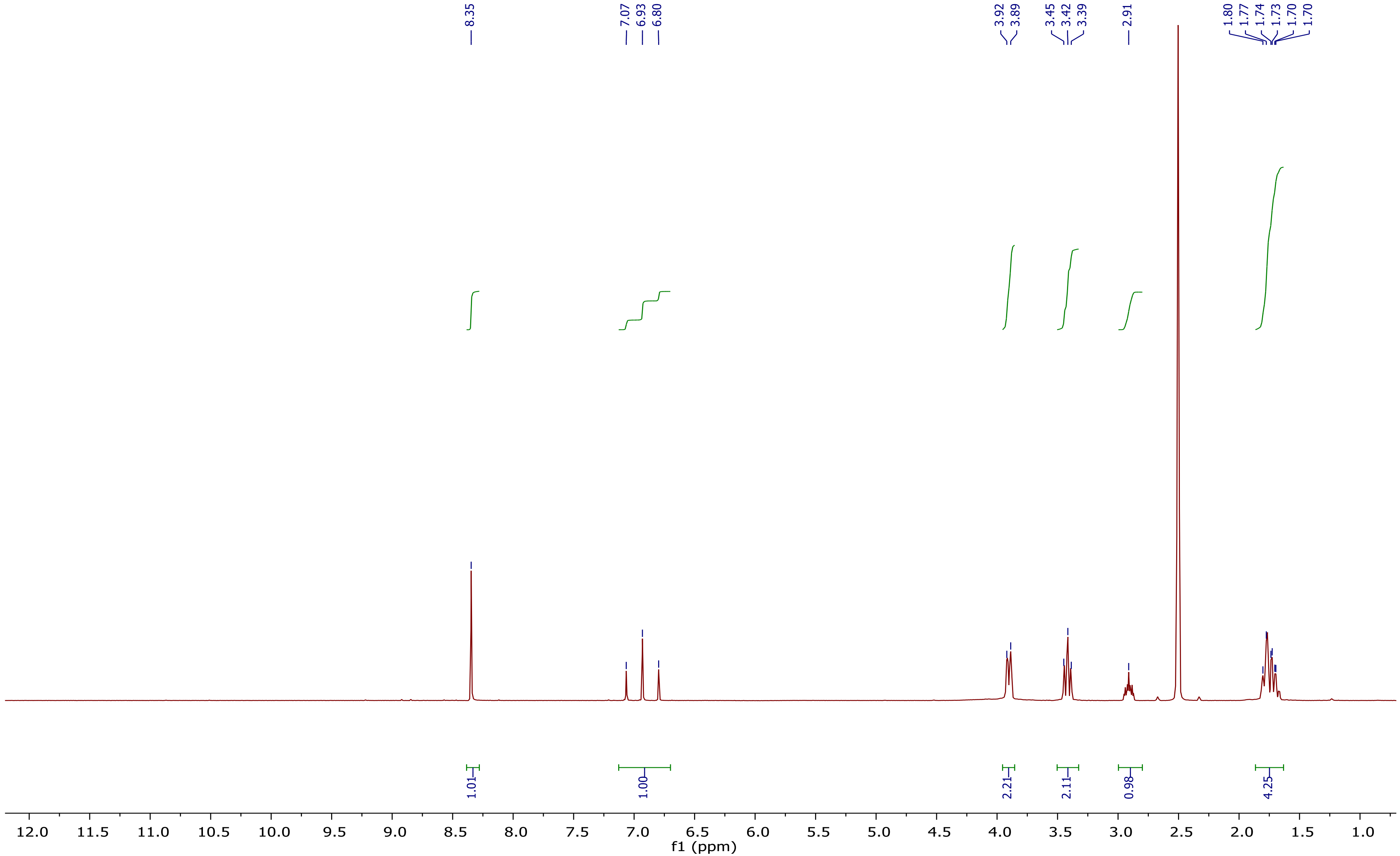
8.35
7.07
6.93
6.80
3.92
3.89
3.45
3.42
3.39
2.91
1.80
1.77
1.74
1.73
1.70
1.70
1.01
1.00
2.21
2.11
0.98
4.25
12.0 11.5 11.0 10.5 10.0 9.5 9.0 8.5 8.0 7.5 7.0 6.5 6.0 5.5 5.0 4.5 4.0 3.5 3.0 2.5 2.0 1.5 1.0
f1 (ppm)

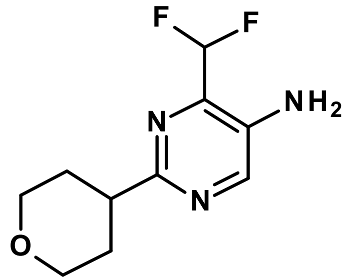

13

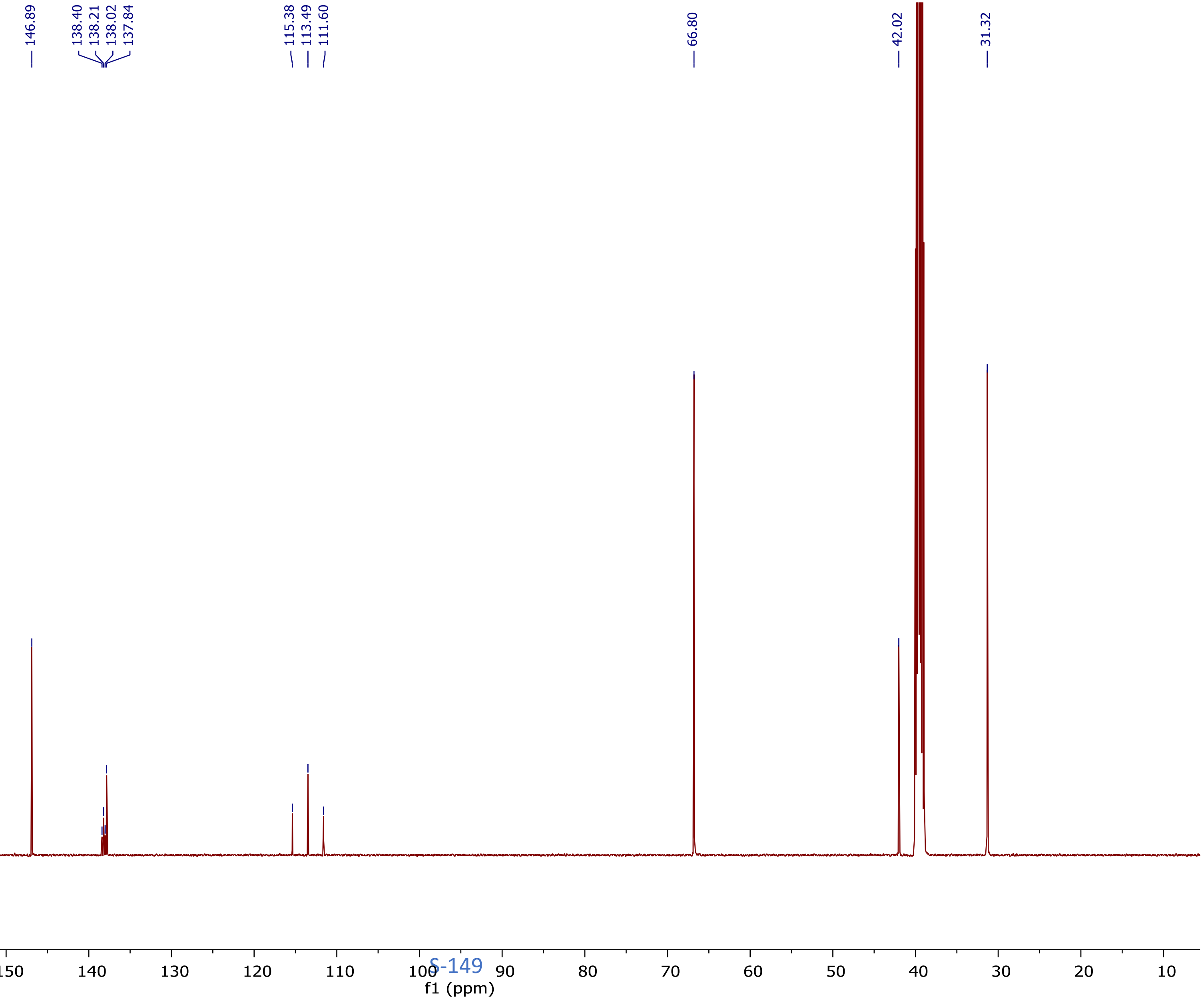
159.27
146.89
138.40
138.21
138.02
137.84
115.38
113.49
111.60
66.80
42.02
31.32
180
170
160
150
140
130
120
110
100
90
80
70
60
50
40
30
20
10
f1 (ppm)

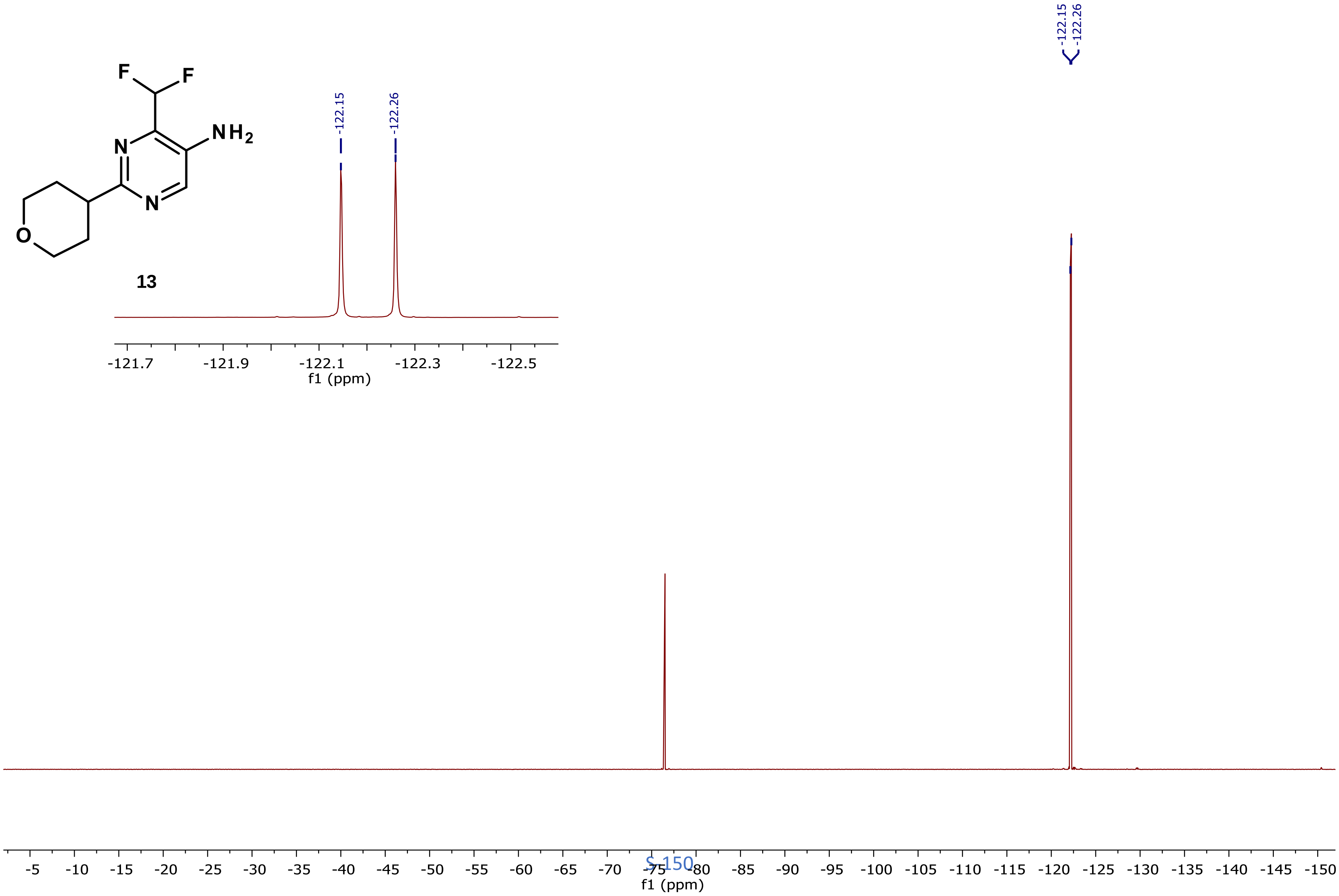
F
F
NH2
N
N
O
13
-122.15
-122.26
-121.7
-121.9
-122.1
-122.3
-122.5
f1 (ppm)
-5 -10 -15 -20 -25 -30 -35 -40 -45 -50 -55 -60 -65 -70 -75 -80 -85 -90 -95 -100 -105 -110 -115 -120 -125 -130 -135 -140 -145 -150
f1 (ppm)

q. ^{1}H, ^{13}C and ^{19}F NMR of 14

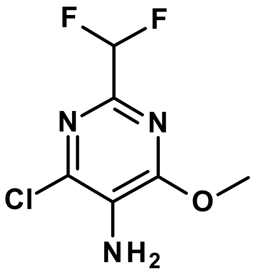

14

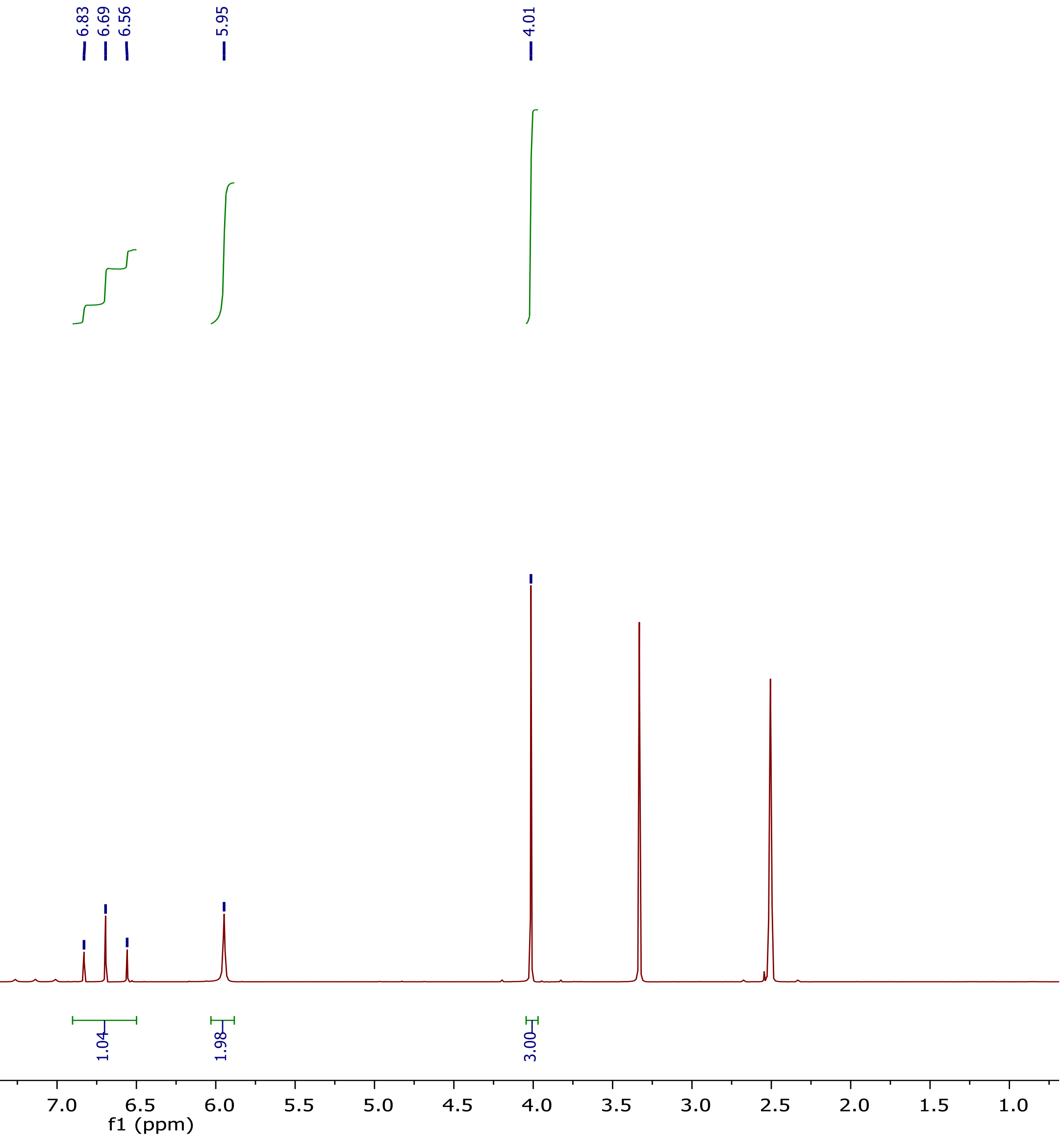
6.83
6.69
6.56
5.95
4.01
1.04
1.98
3.00
12.0
11.5
11.0
10.5
10.0
9.5
9.0
8.5
8.0
7.5
7.0
6.5
6.0
5.5
5.0
4.5
4.0
3.5
3.0
2.5
2.0
1.5
1.0
f1 (ppm)

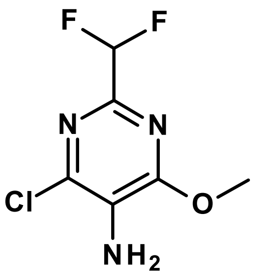

14

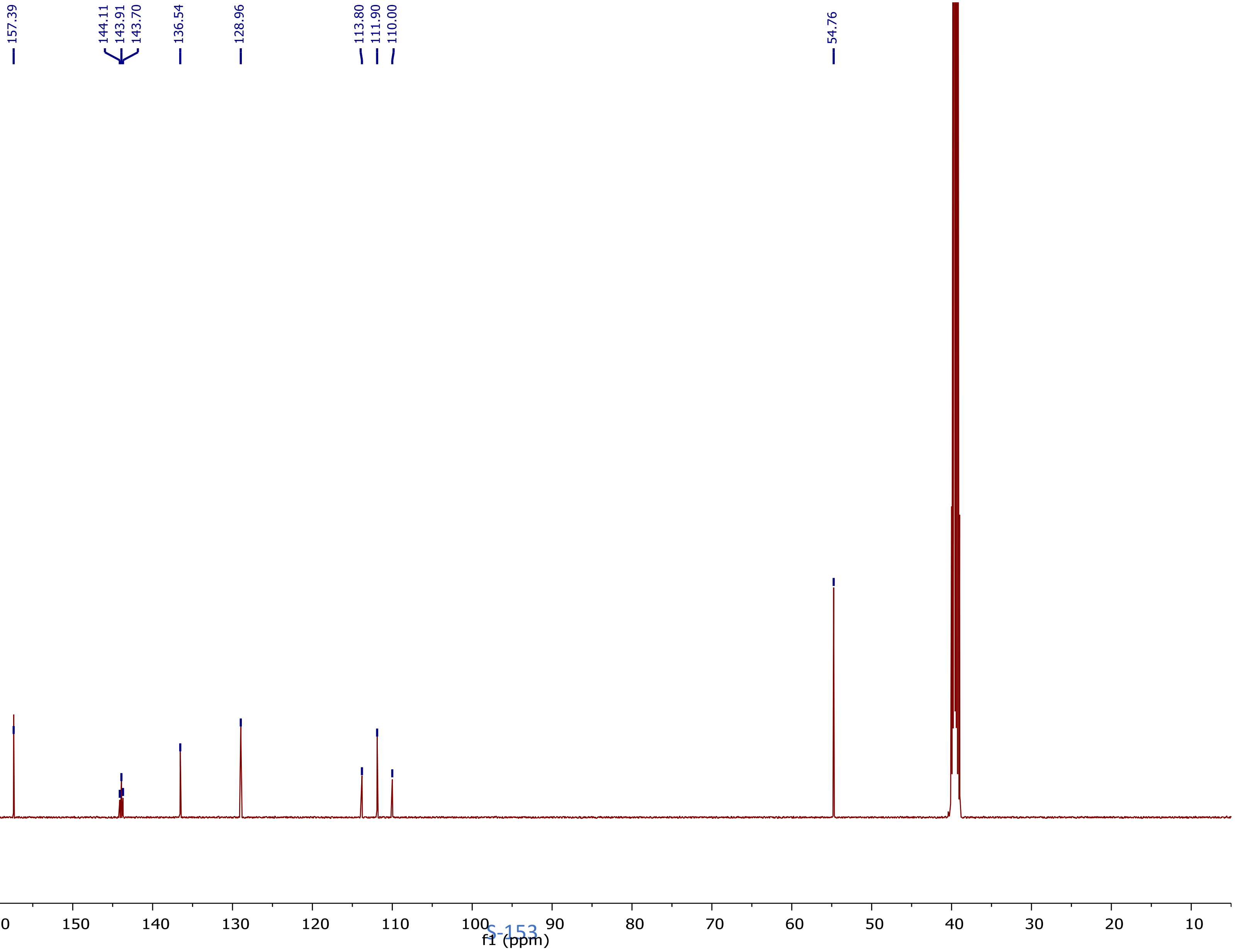
157.39
144.11
143.91
143.70
136.54
128.96
113.80
111.90
110.00
54.76
180
170
160
150
140
130
120
110
100
90
80
70
60
50
40
30
20
10
f1 (ppm)

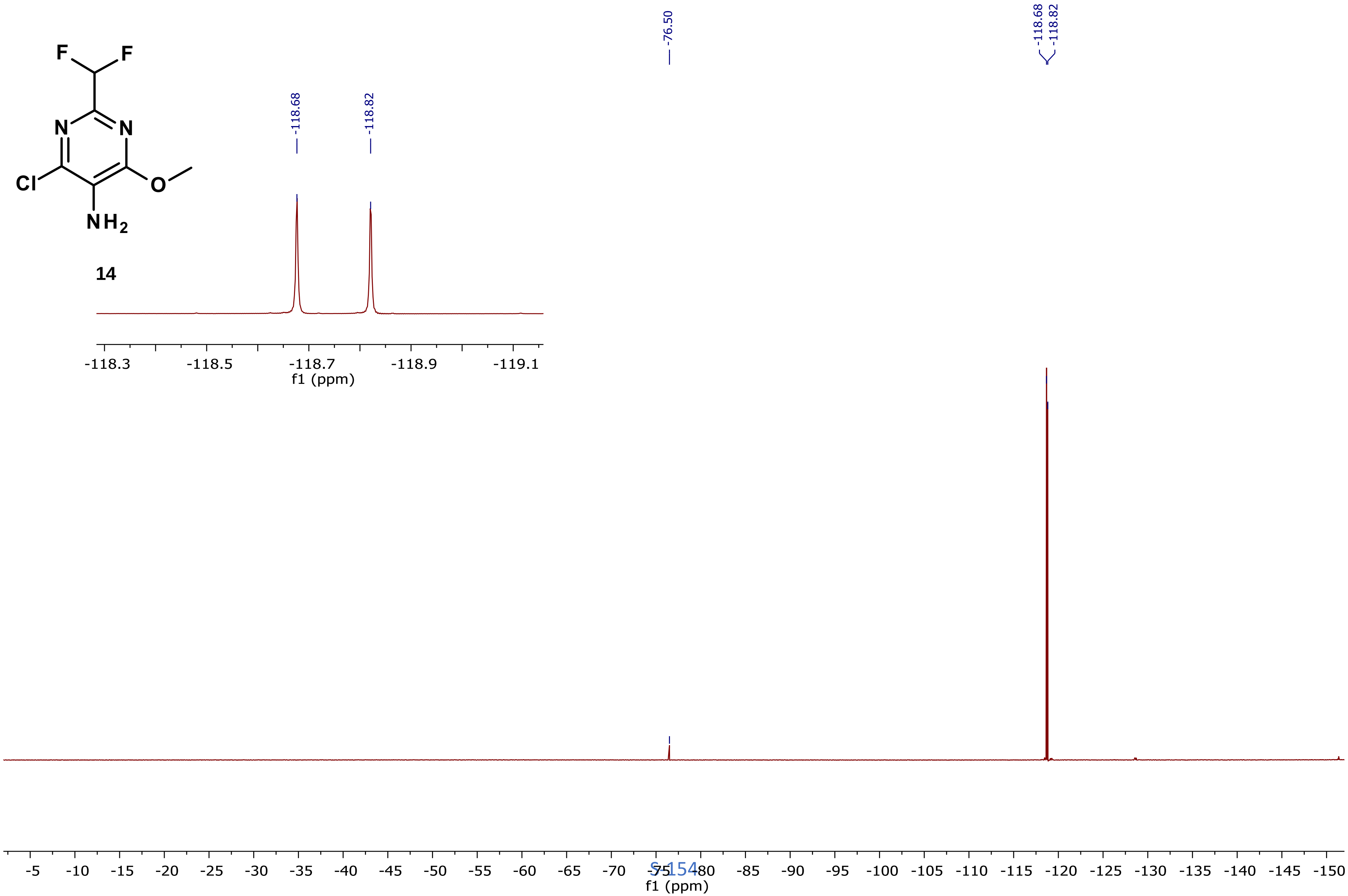
14
-118.68
-118.82
-118.3
-118.5
-118.7
-118.9
-119.1
f1 (ppm)
-76.50
-118.68
-118.82
-5
-10
-15
-20
-25
-30
-35
-40
-45
-50
-55
-60
-65
-70
-75
-80
-85
-90
-95
-100
-105
-110
-115
-120
-125
-130
-135
-140
-145
-150
f1 (ppm)

r. ^{1}H, ^{13}C and ^{19}F NMR of 15

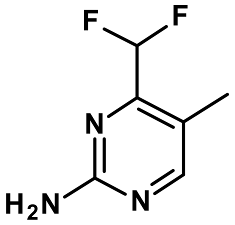

15

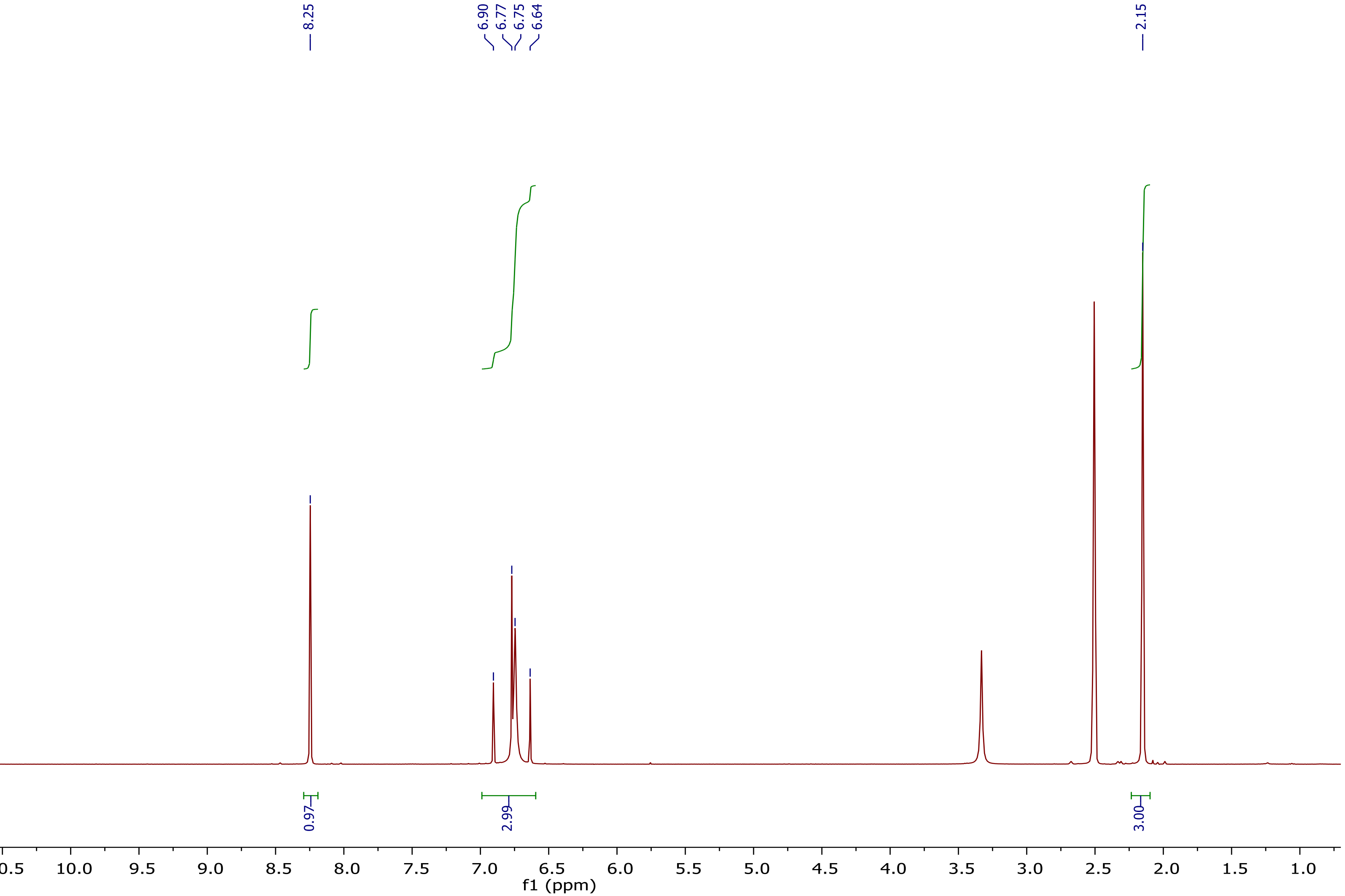
8.25
6.90
6.77
6.75
6.64
2.15
0.97
2.99
3.00
12.0
11.5
11.0
10.5
10.0
9.5
9.0
8.5
8.0
7.5
7.0
6.5
6.0
5.5
5.0
4.5
4.0
3.5
3.0
2.5
2.0
1.5
1.0
f1 (ppm)

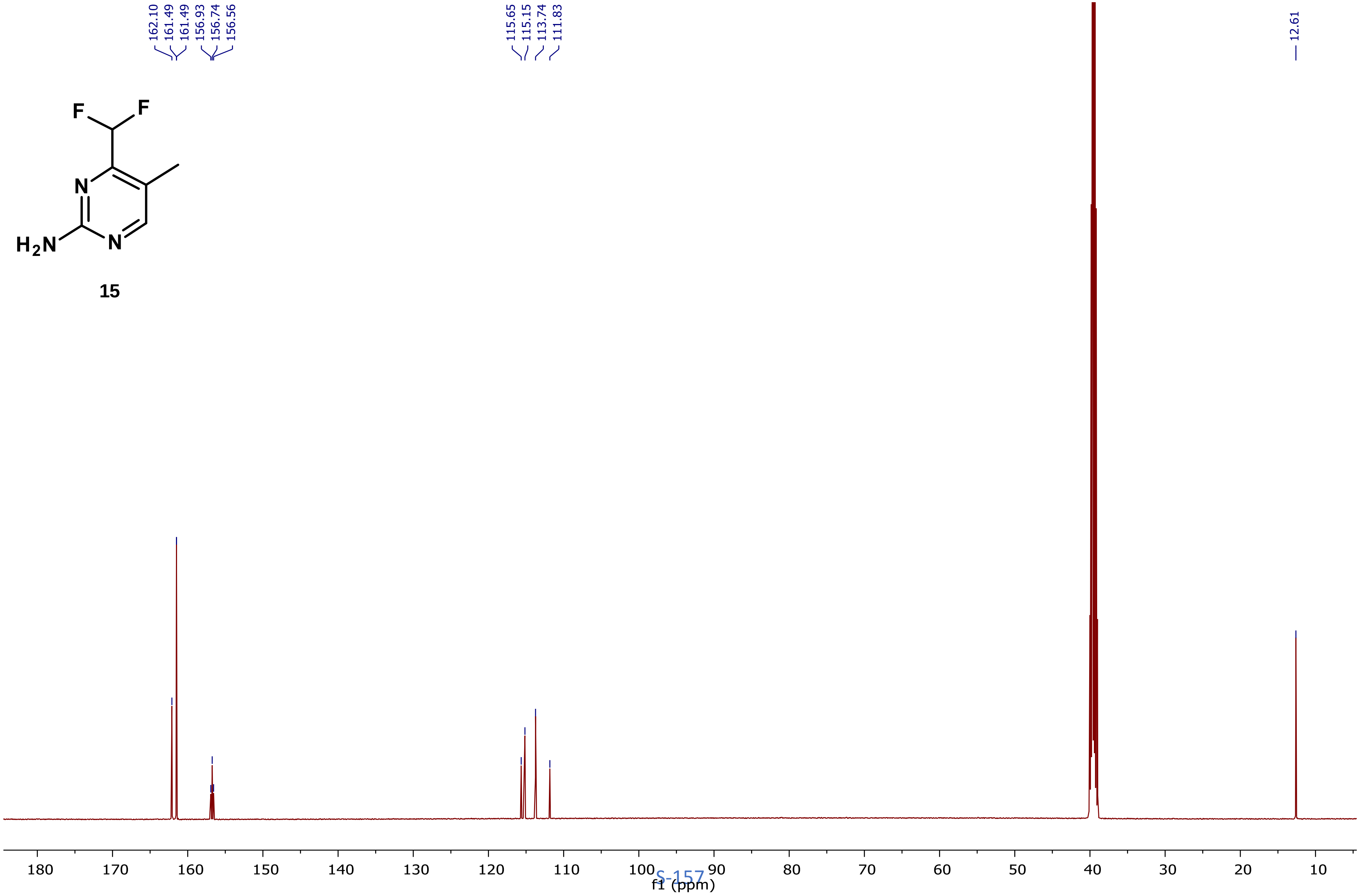
162.10
161.49
161.49
156.93
156.74
156.56
115.65
115.15
113.74
111.83
12.61
F
F
N
H_2N
N
15
180 170 160 150 140 130 120 110 100 90 80 70 60 50 40 30 20 10
f1 (ppm)

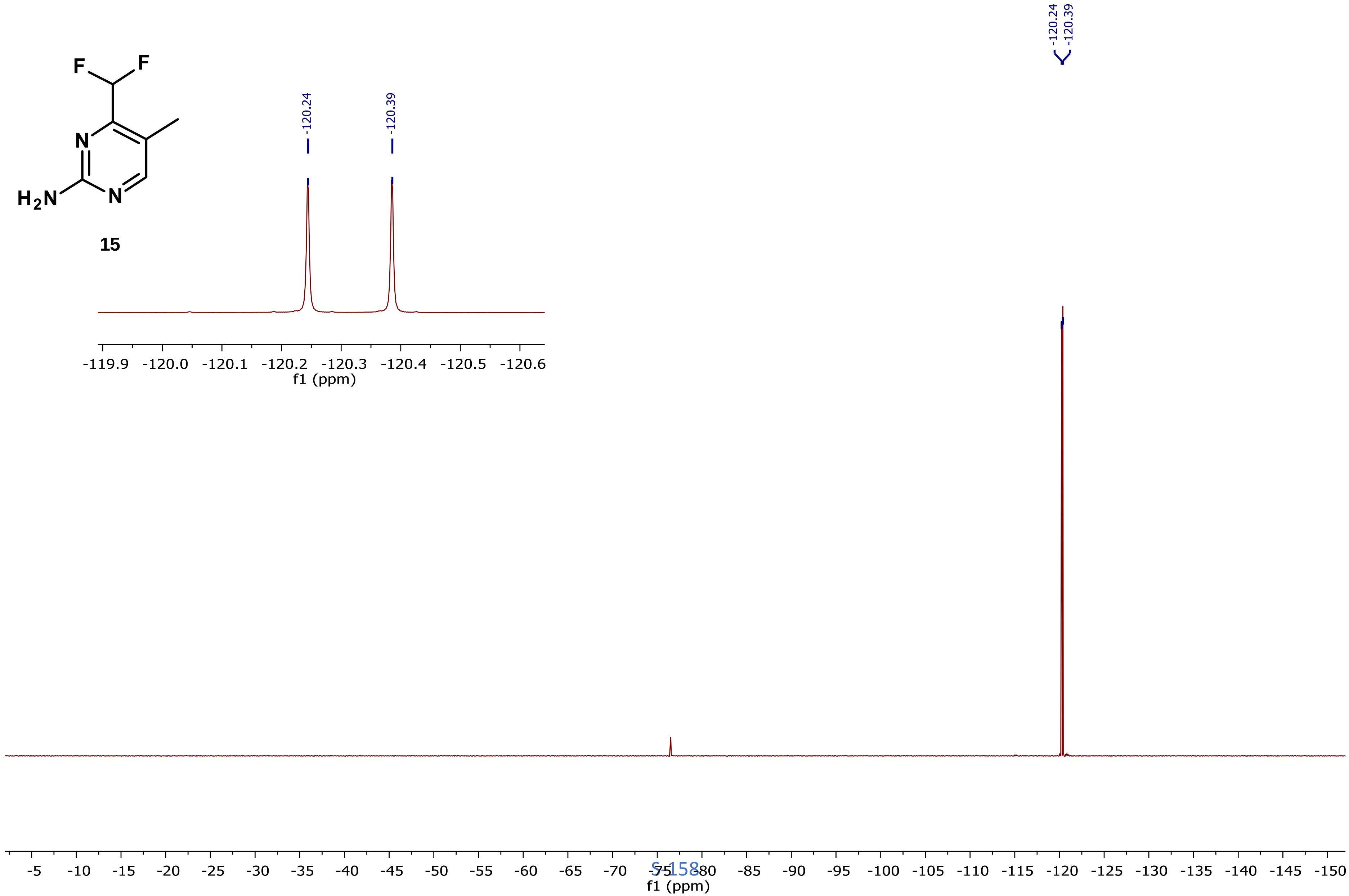

15
-120.24
-120.39
-119.9 -120.0 -120.1 -120.2 -120.3 -120.4 -120.5 -120.6
f1 (ppm)
-5 -10 -15 -20 -25 -30 -35 -40 -45 -50 -55 -60 -65 -70 -75 -80 -85 -90 -95 -100 -105 -110 -115 -120 -125 -130 -135 -140 -145 -150
f1 (ppm)

s. ^{1}H, ^{13}C and ^{19}F NMR of 16

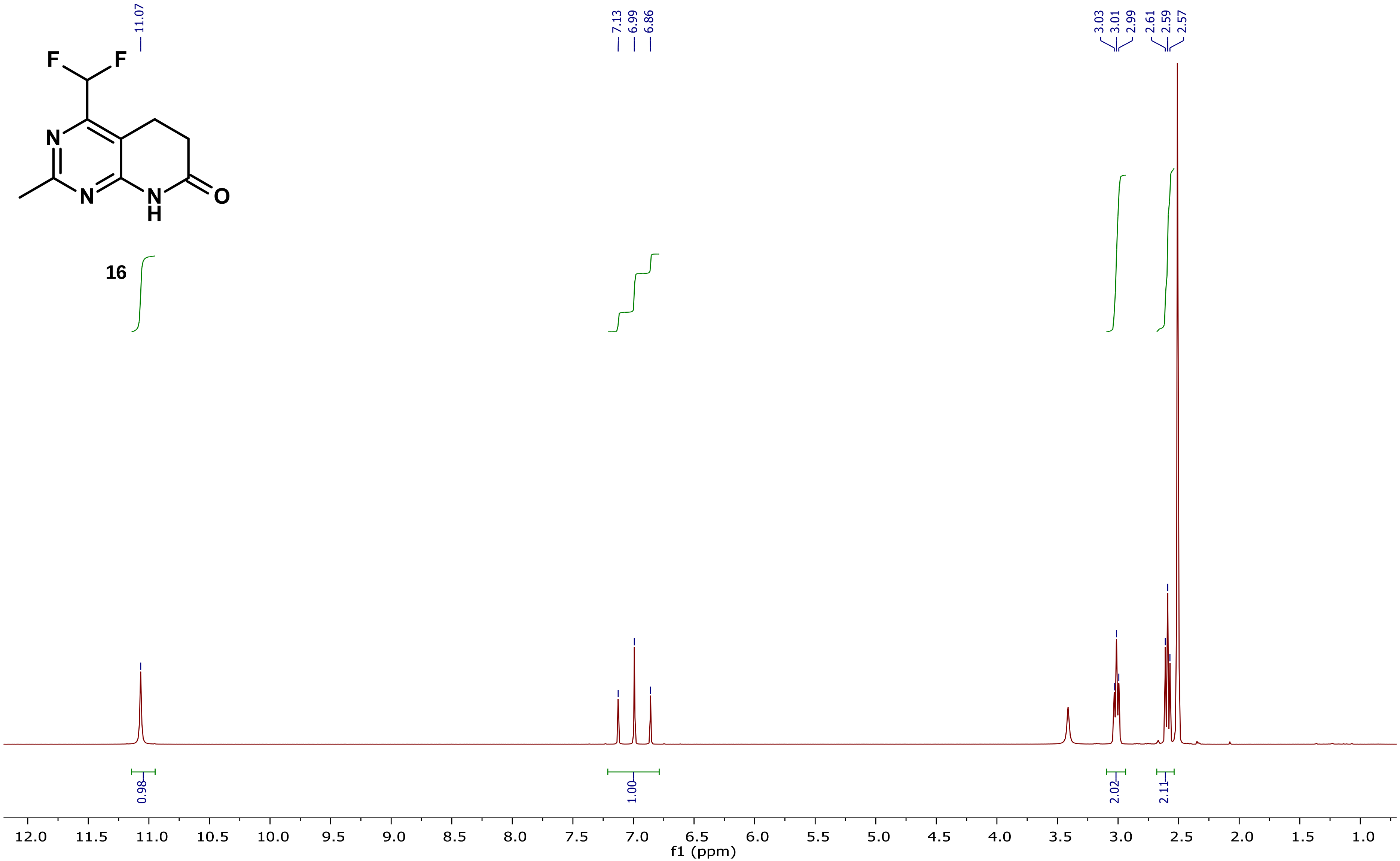
16
11.07
7.13
6.99
6.86
3.03
3.01
2.99
2.61
2.59
2.57
0.98
1.00
2.02
2.11
12.0
11.5
11.0
10.5
10.0
9.5
9.0
8.5
8.0
7.5
7.0
6.5
6.0
5.5
5.0
4.5
4.0
3.5
3.0
2.5
2.0
1.5
1.0
f1 (ppm)

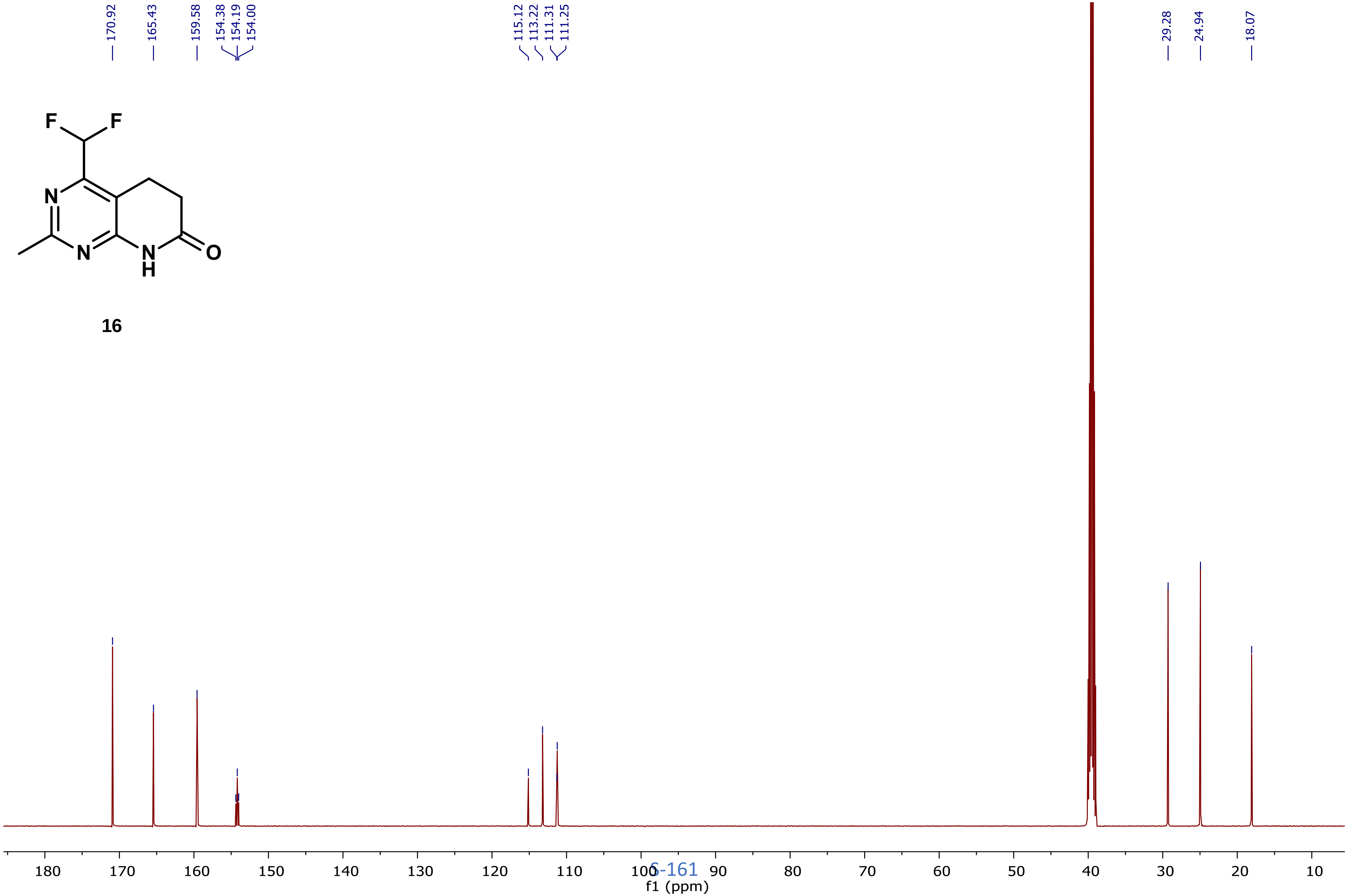

16
170.92
165.43
159.58
154.38
154.19
154.00
115.12
113.22
111.31
111.25
29.28
24.94
18.07
180 170 160 150 140 130 120 110 100 90 80 70 60 50 40 30 20 10
f1 (ppm)

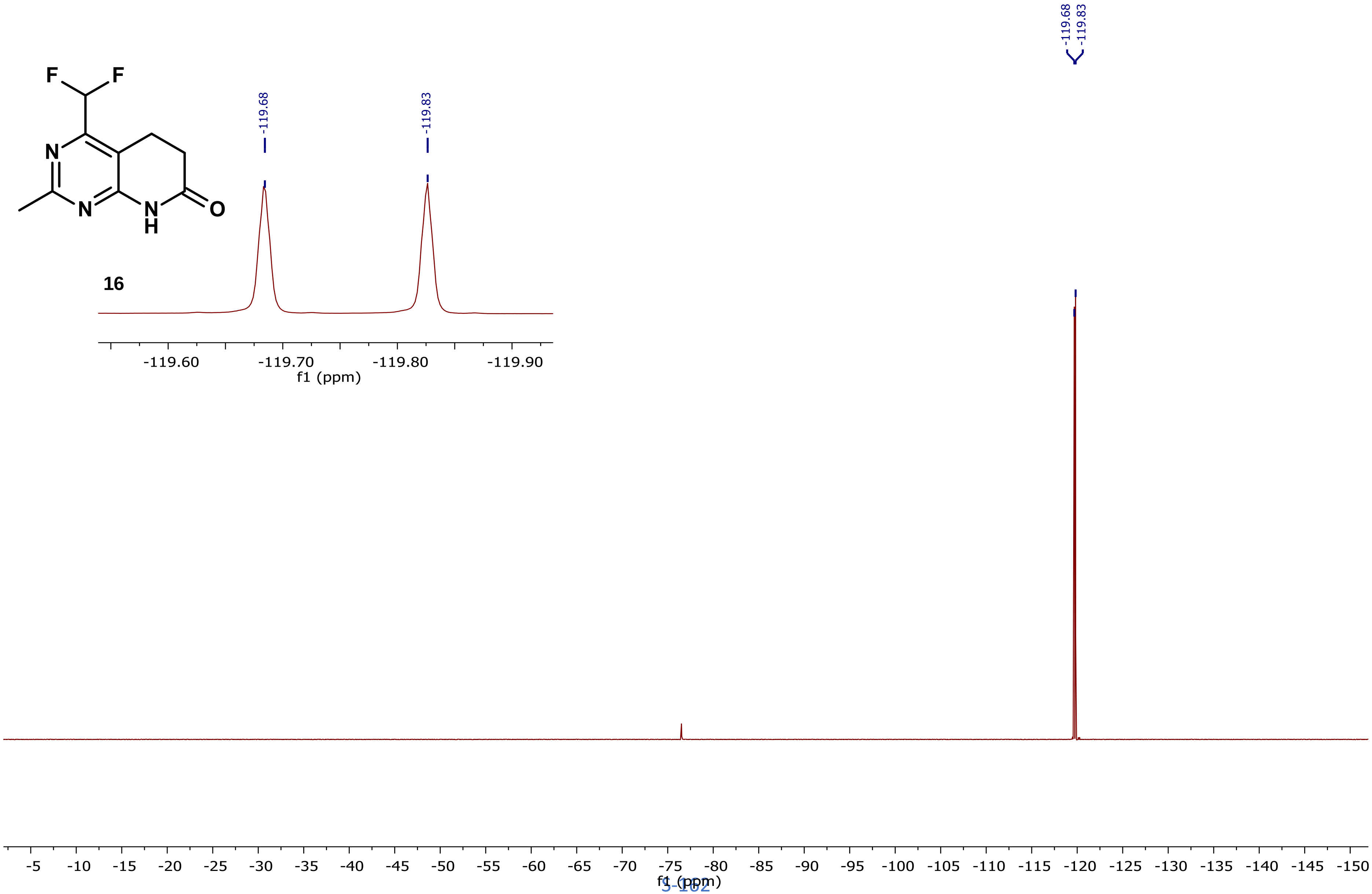
F
F
N
N
N
H
O
16
-119.68
-119.83
-119.60
-119.70
-119.80
-119.90
f1 (ppm)
-5 -10 -15 -20 -25 -30 -35 -40 -45 -50 -55 -60 -65 -70 -75 -80 -85 -90 -95 -100 -105 -110 -115 -120 -125 -130 -135 -140 -145 -150
f1 (ppm)

t. ^{1}H, ^{13}C and ^{19}F NMR of 17

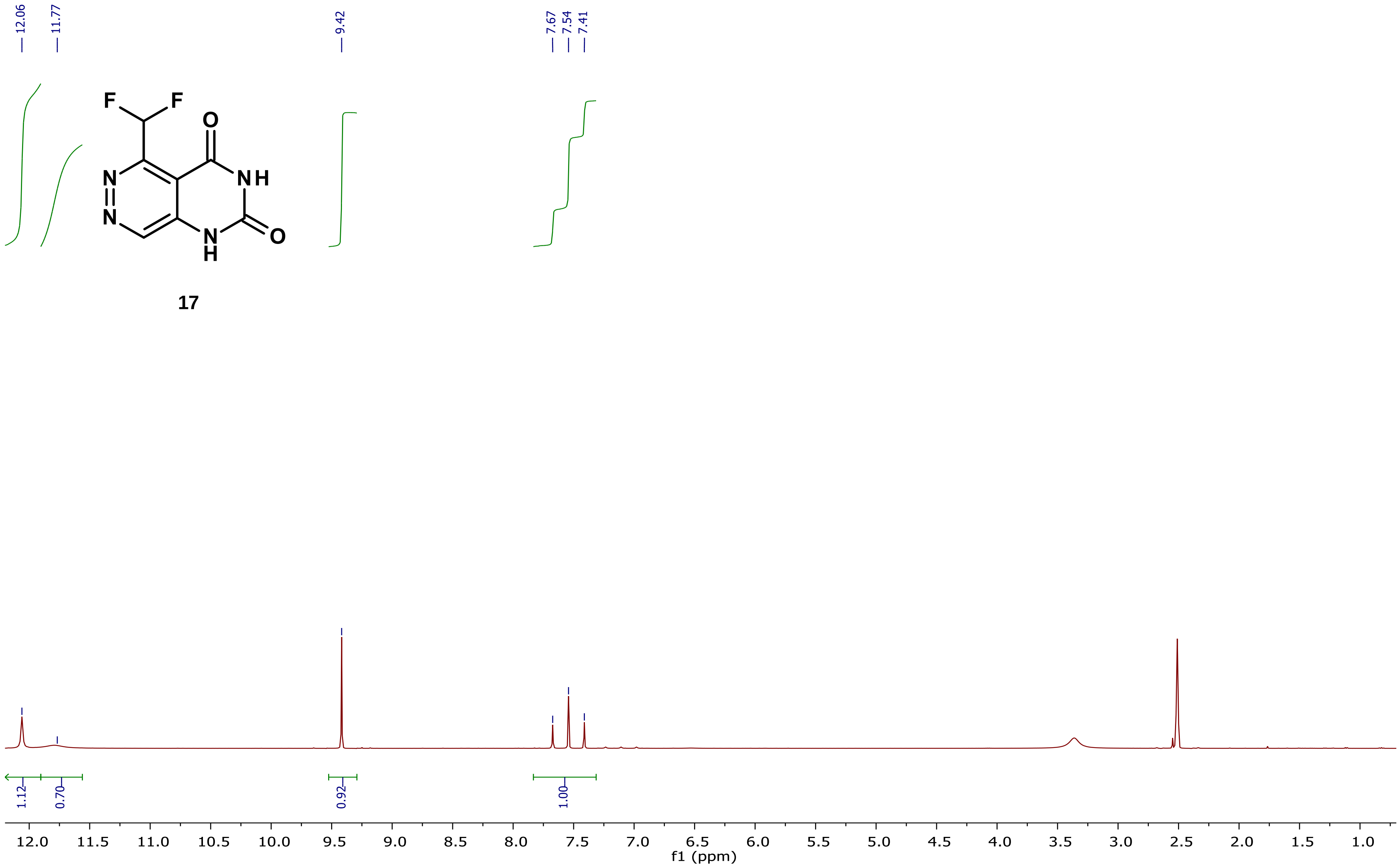
12.06
11.77
9.42
7.67
7.54
7.41
17
1.12
0.70
0.92
1.00
12.0 11.5 11.0 10.5 10.0 9.5 9.0 8.5 8.0 7.5 7.0 6.5 6.0 5.5 5.0 4.5 4.0 3.5 3.0 2.5 2.0 1.5 1.0
f1 (ppm)

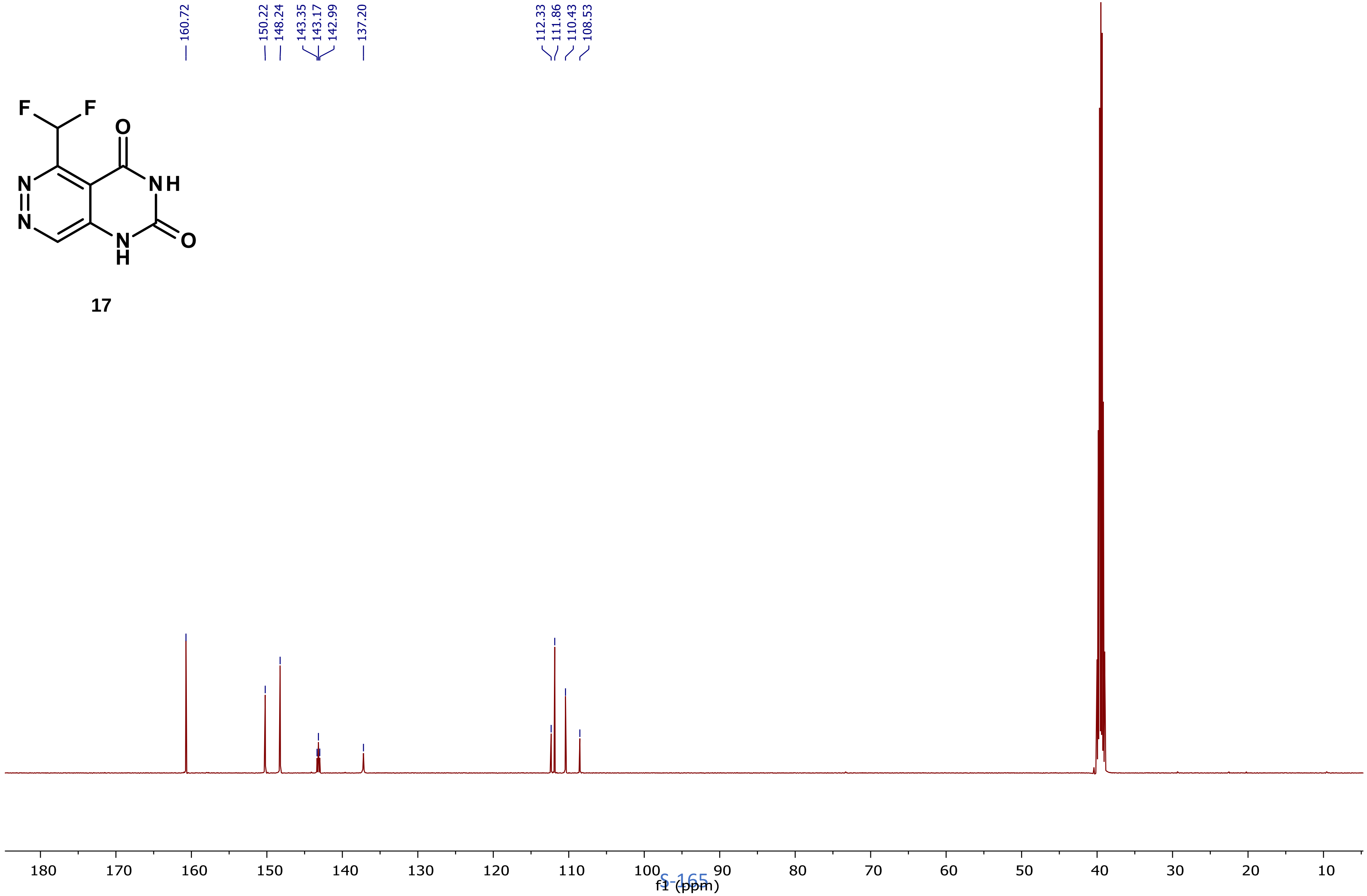
160.72
150.22
148.24
143.35
143.17
142.99
137.20
112.33
111.86
110.43
108.53
F
F
O
NH
N
N
O
N
H
17
180
170
160
150
140
130
120
110
100
90
80
70
60
50
40
30
20
10
f1 (ppm)

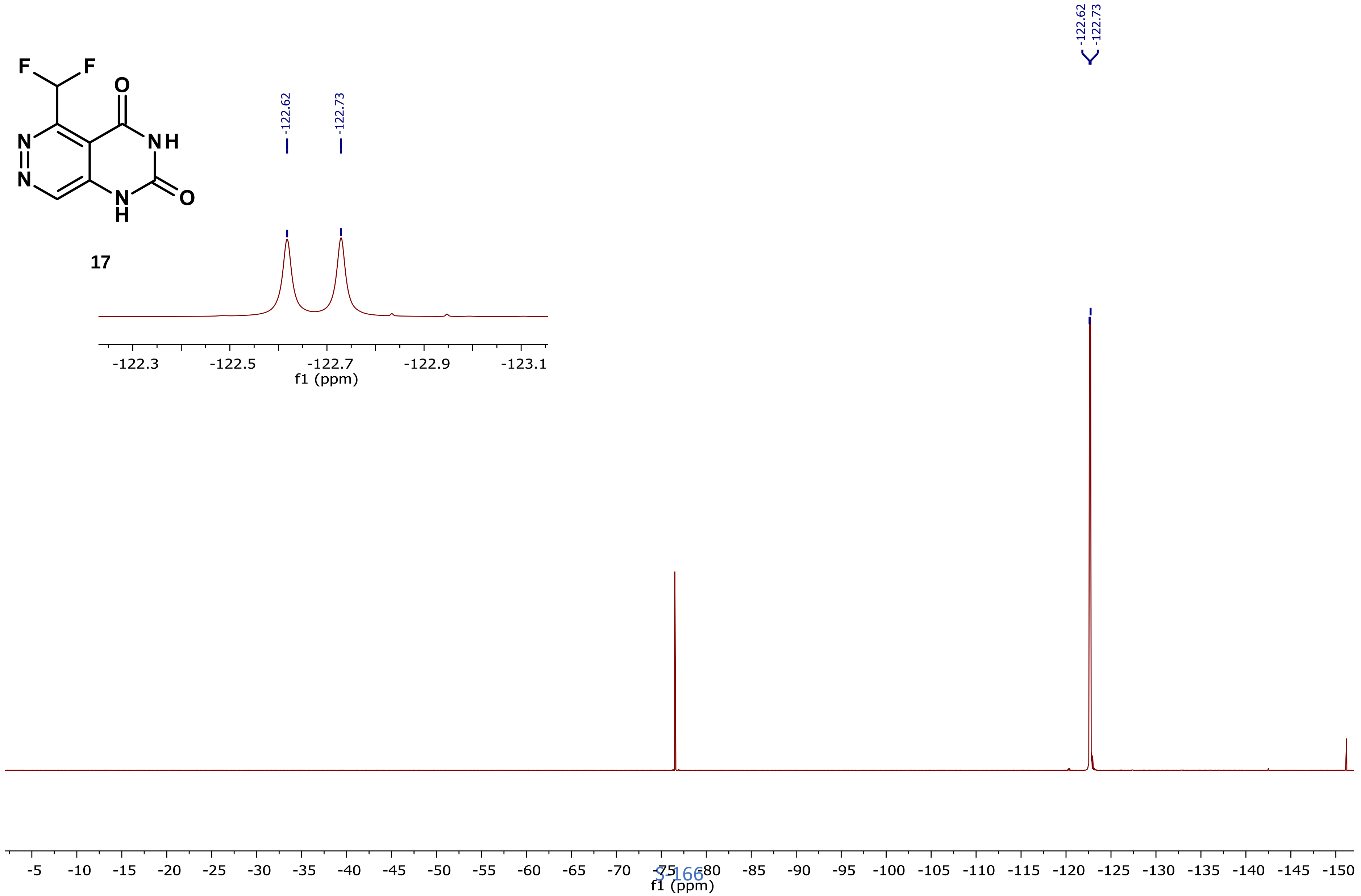
17
-122.62
-122.73
-122.3
-122.5
-122.7
-122.9
-123.1
f1 (ppm)
-5 -10 -15 -20 -25 -30 -35 -40 -45 -50 -55 -60 -65 -70 -75 -80 -85 -90 -95 -100 -105 -110 -115 -120 -125 -130 -135 -140 -145 -150
f1 (ppm)

u. ^{1}H, ^{13}C and ^{19}F NMR of 18

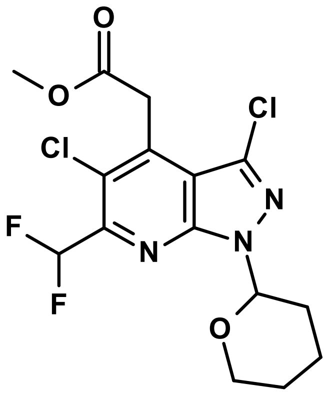

18

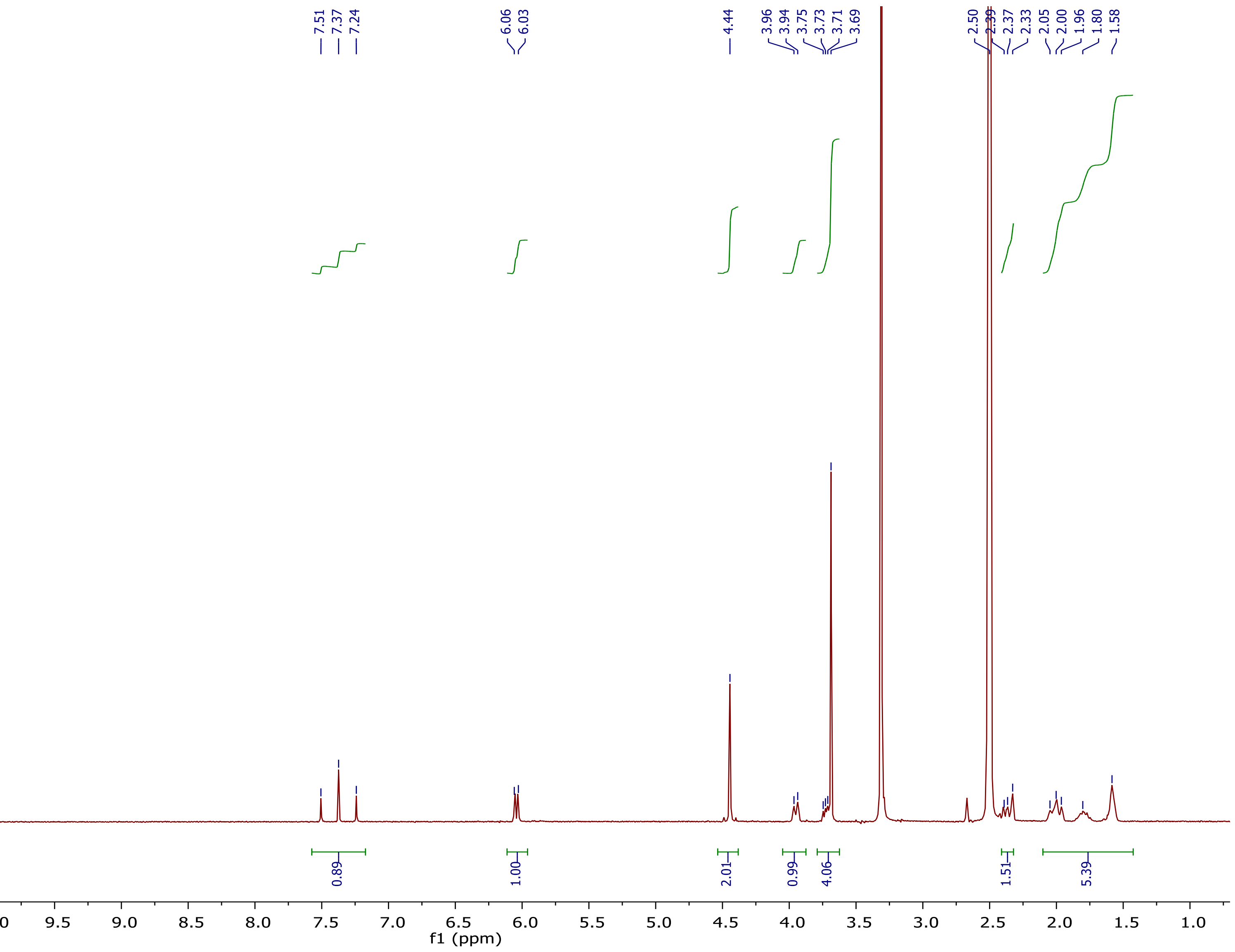

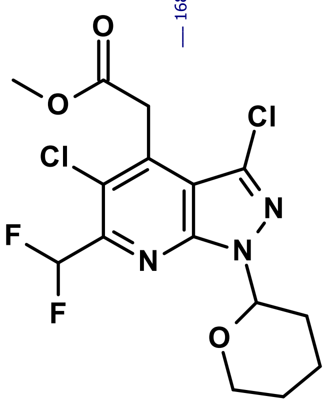
O
O
Cl
Cl
F
F
N
N
N
O

18

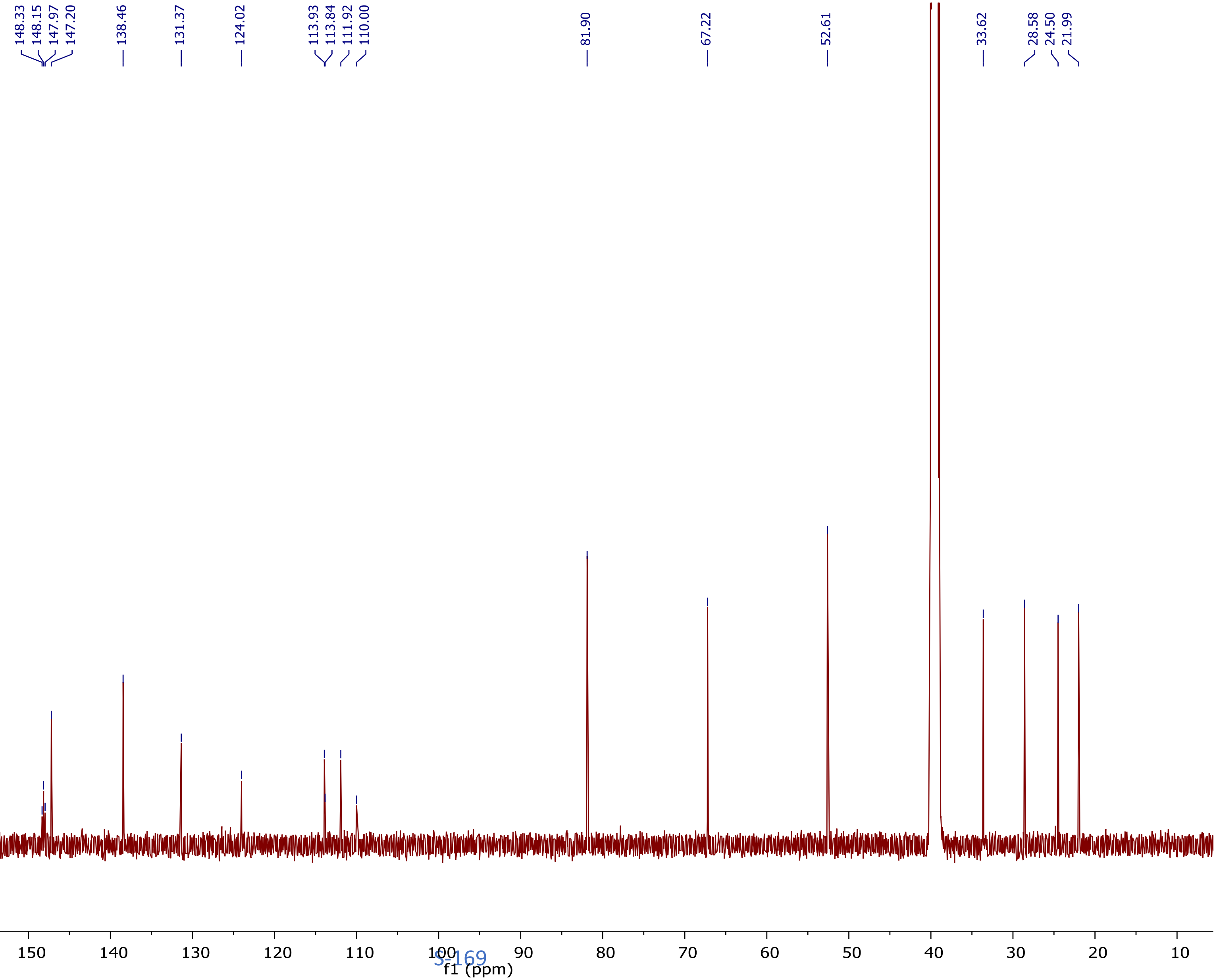
168.47
148.33
148.15
147.97
147.20
138.46
131.37
124.02
113.93
113.84
111.92
110.00
81.90
67.22
52.61
33.62
28.58
24.50
21.99
180 170 160 150 140 130 120 110 100 90 80 70 60 50 40 30 20 10
f1 (ppm)

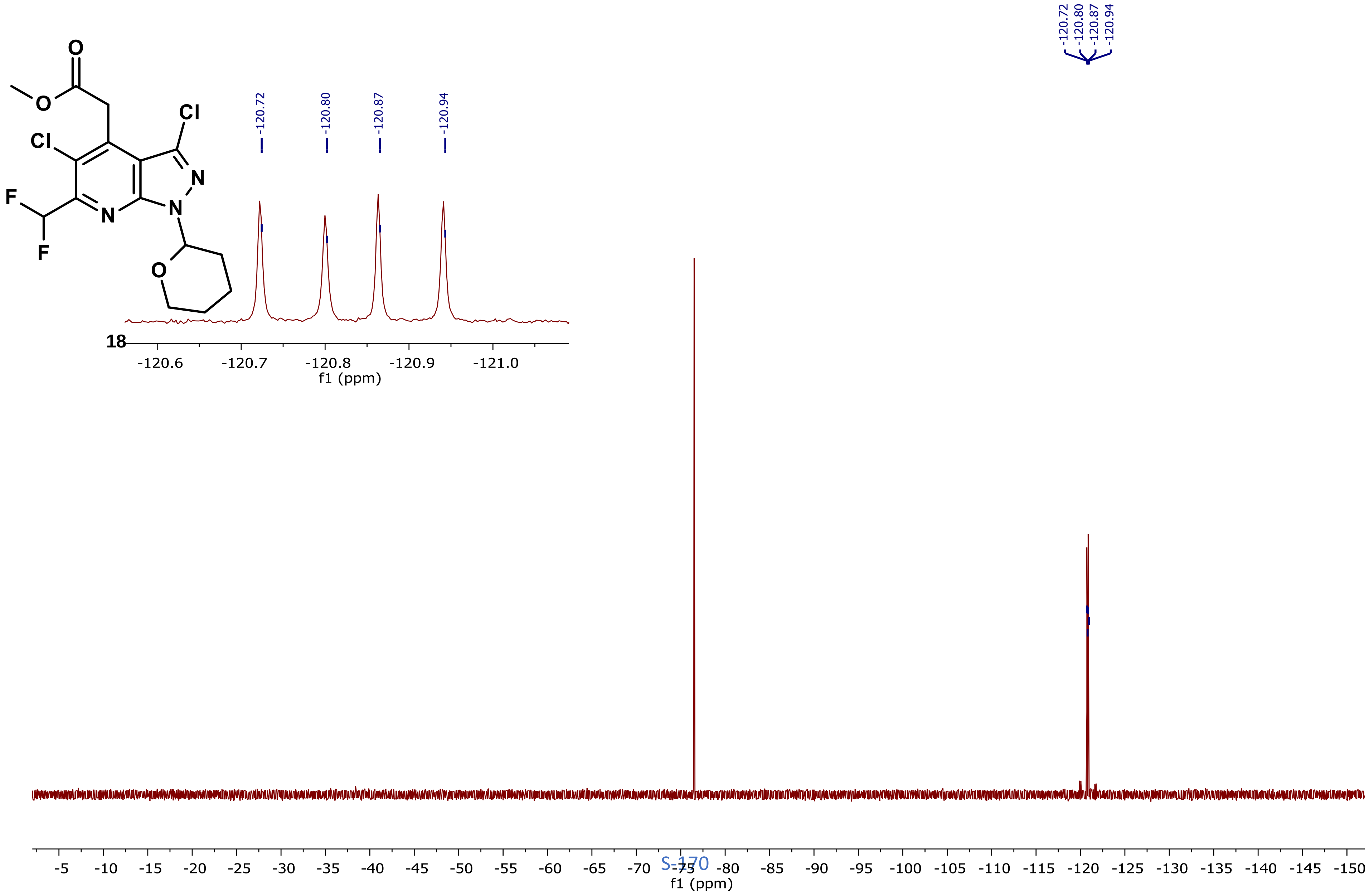
-120.72
-120.80
-120.87
-120.94
O
O
Cl
Cl
N
N
N
F
F
O
18
-120.6
-120.7
-120.8
-120.9
-121.0
f1 (ppm)
-5
-10
-15
-20
-25
-30
-35
-40
-45
-50
-55
-60
-65
-70
-75
-80
-85
-90
-95
-100
-105
-110
-115
-120
-125
-130
-135
-140
-145
-150
f1 (ppm)

v. ^{1}H, ^{13}C and ^{19}F NMR of 19

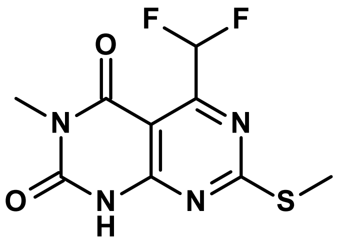

19

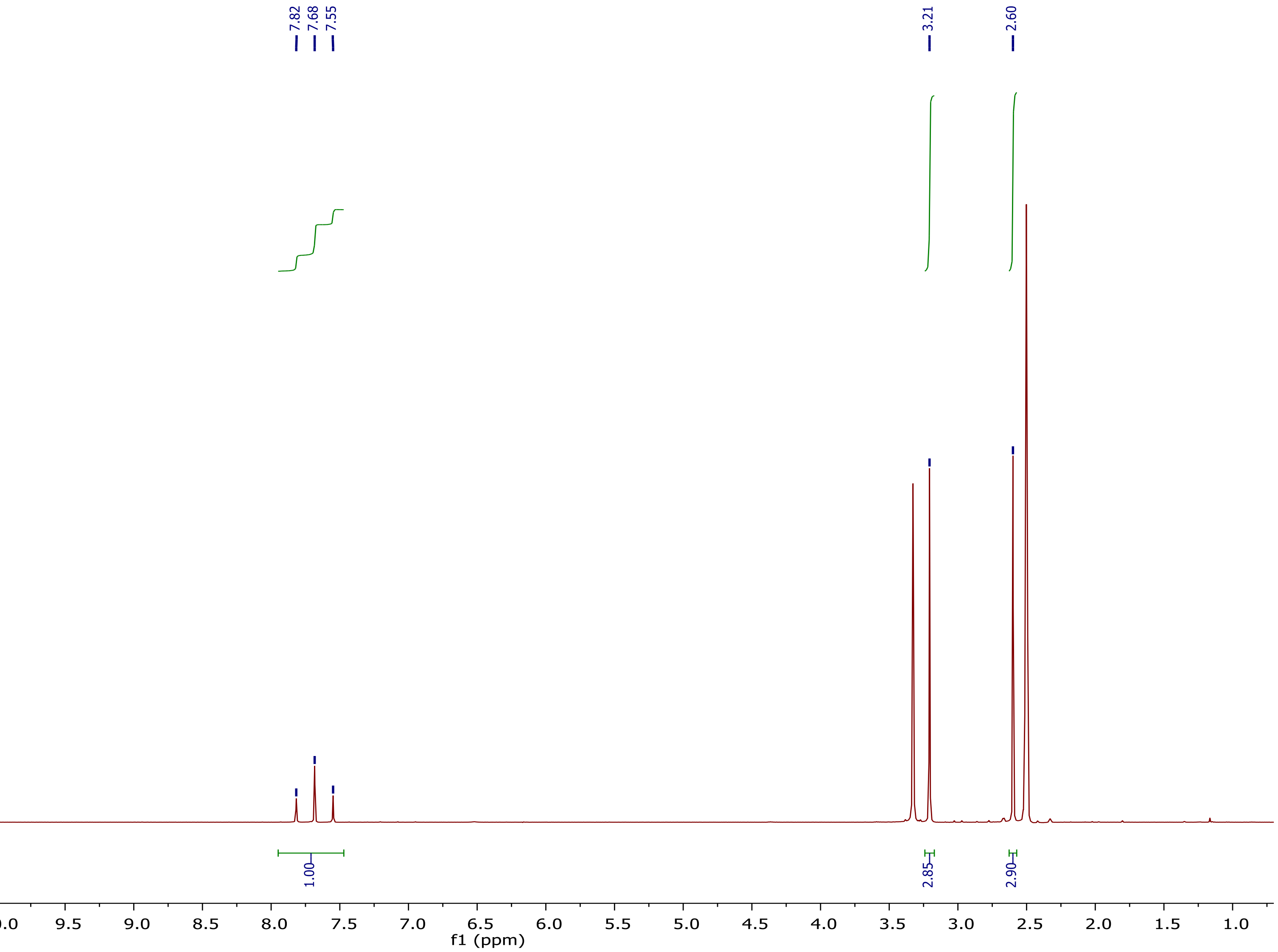

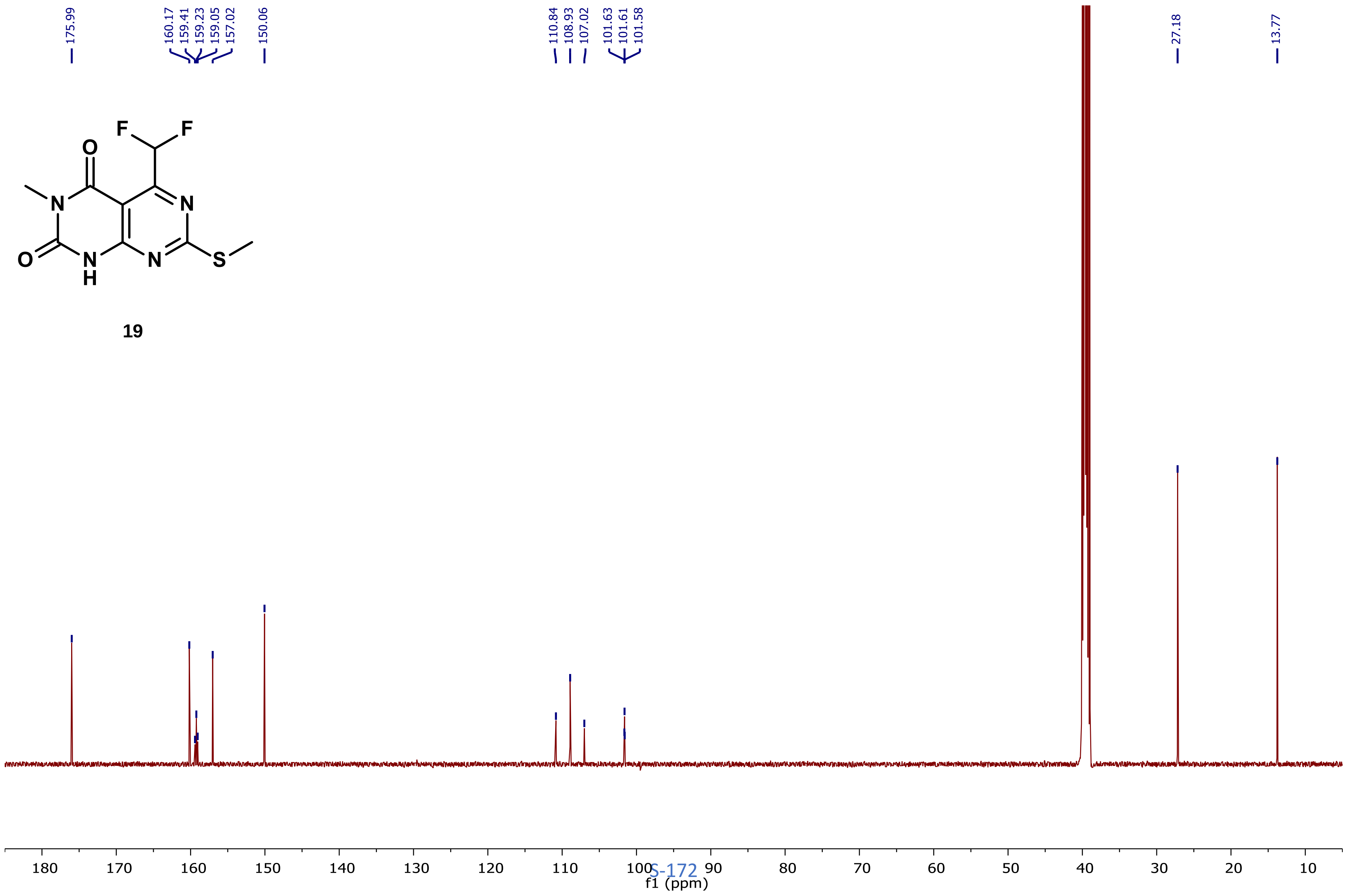
175.99
160.17
159.41
159.23
159.05
157.02
150.06
110.84
108.93
107.02
101.63
101.61
101.58
27.18
13.77
180
170
160
150
140
130
120
110
100
90
80
70
60
50
40
30
20
10
f1 (ppm)

19

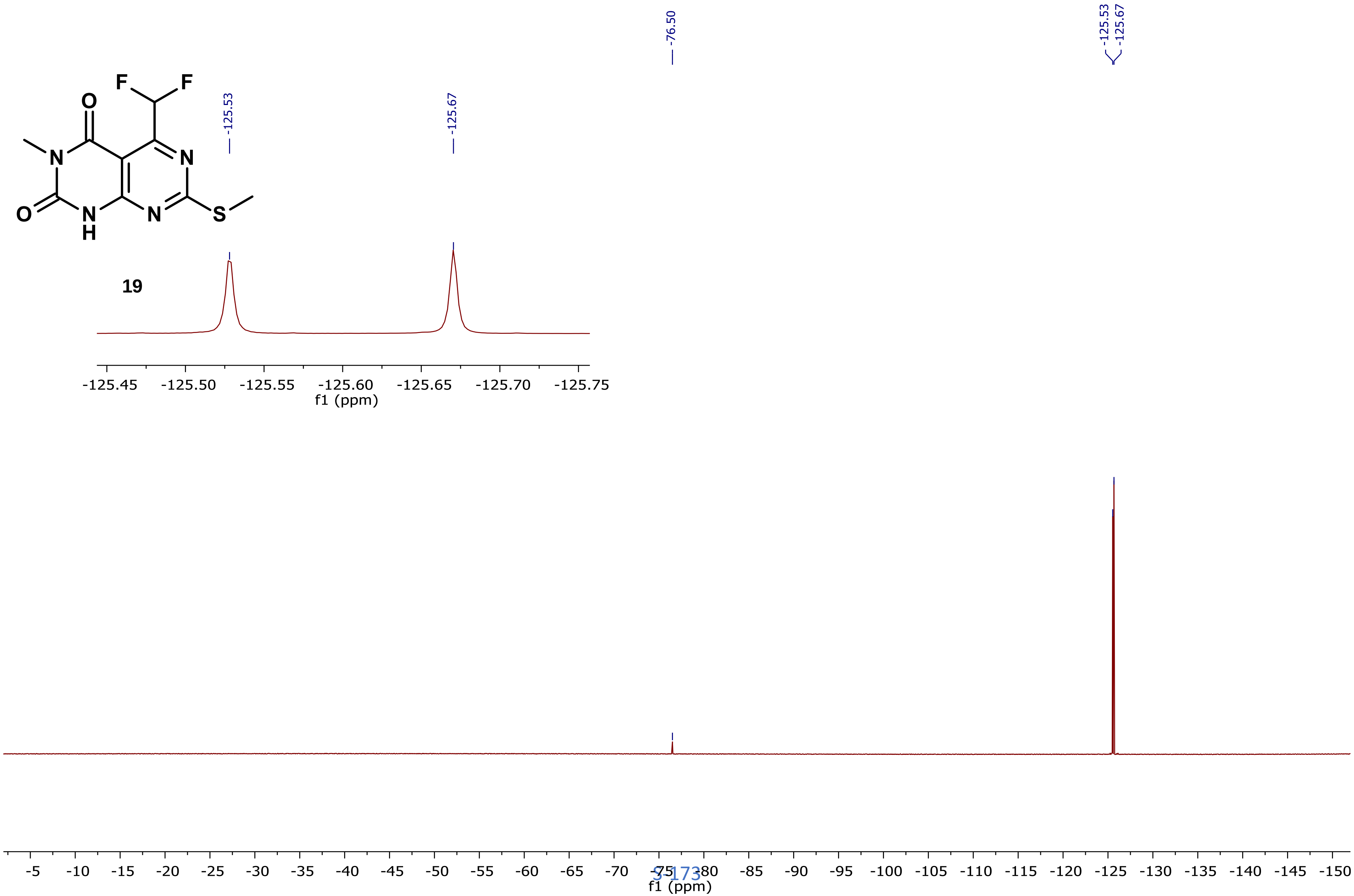

-76.50
-125.53
-125.67
F
F
O
N
N
O
N
H
N
S
-125.45 -125.50 -125.55 -125.60 -125.65 -125.70 -125.75
f1 (ppm)
-5 -10 -15 -20 -25 -30 -35 -40 -45 -50 -55 -60 -65 -70 -75 -80 -85 -90 -95 -100 -105 -110 -115 -120 -125 -130 -135 -140 -145 -150
f1 (ppm)

19

w. ^{1}H, ^{13}C and ^{19}F NMR of 20

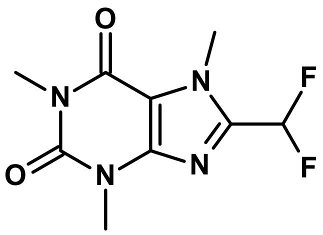

20

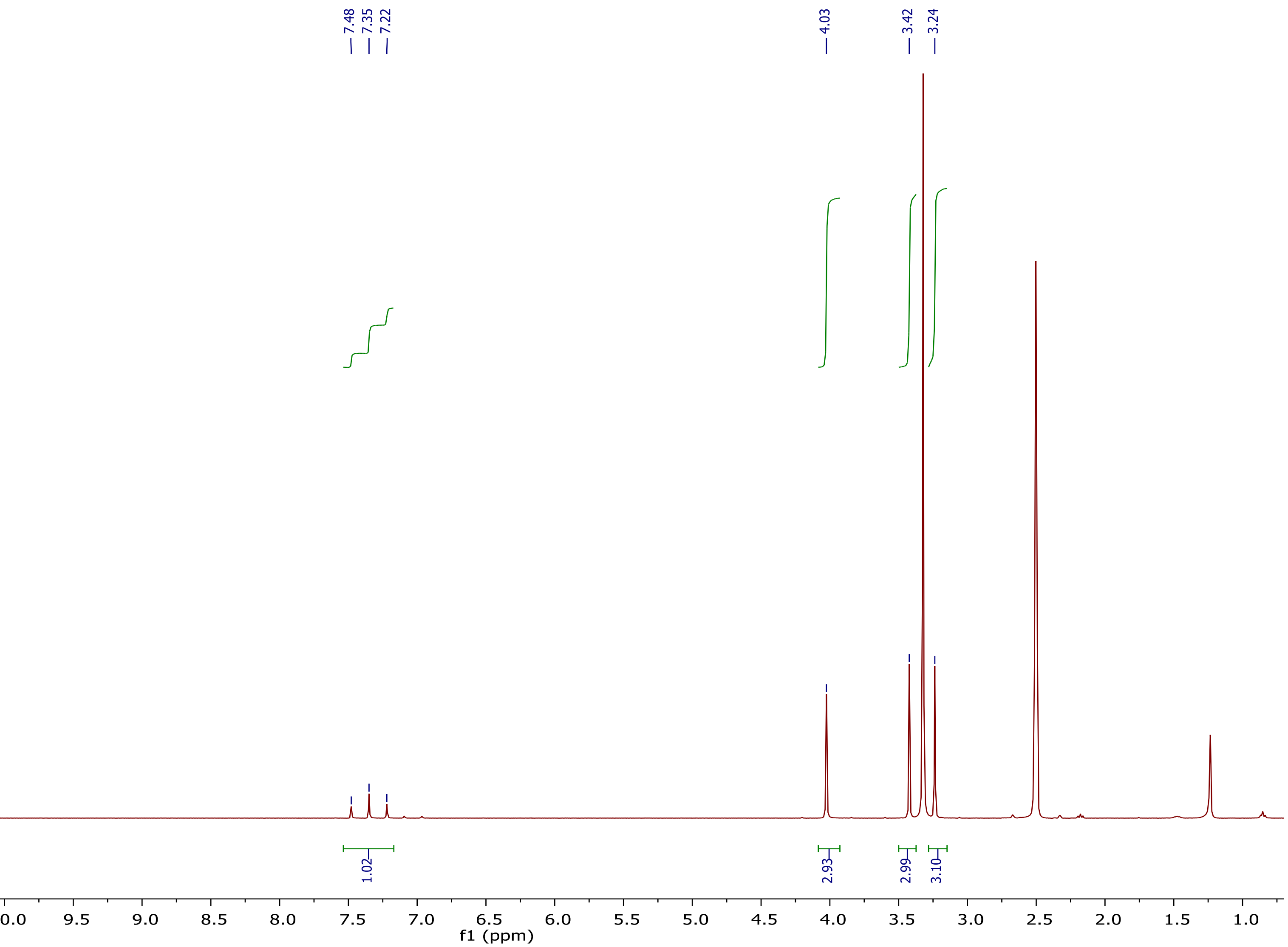

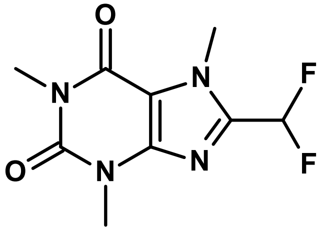

20

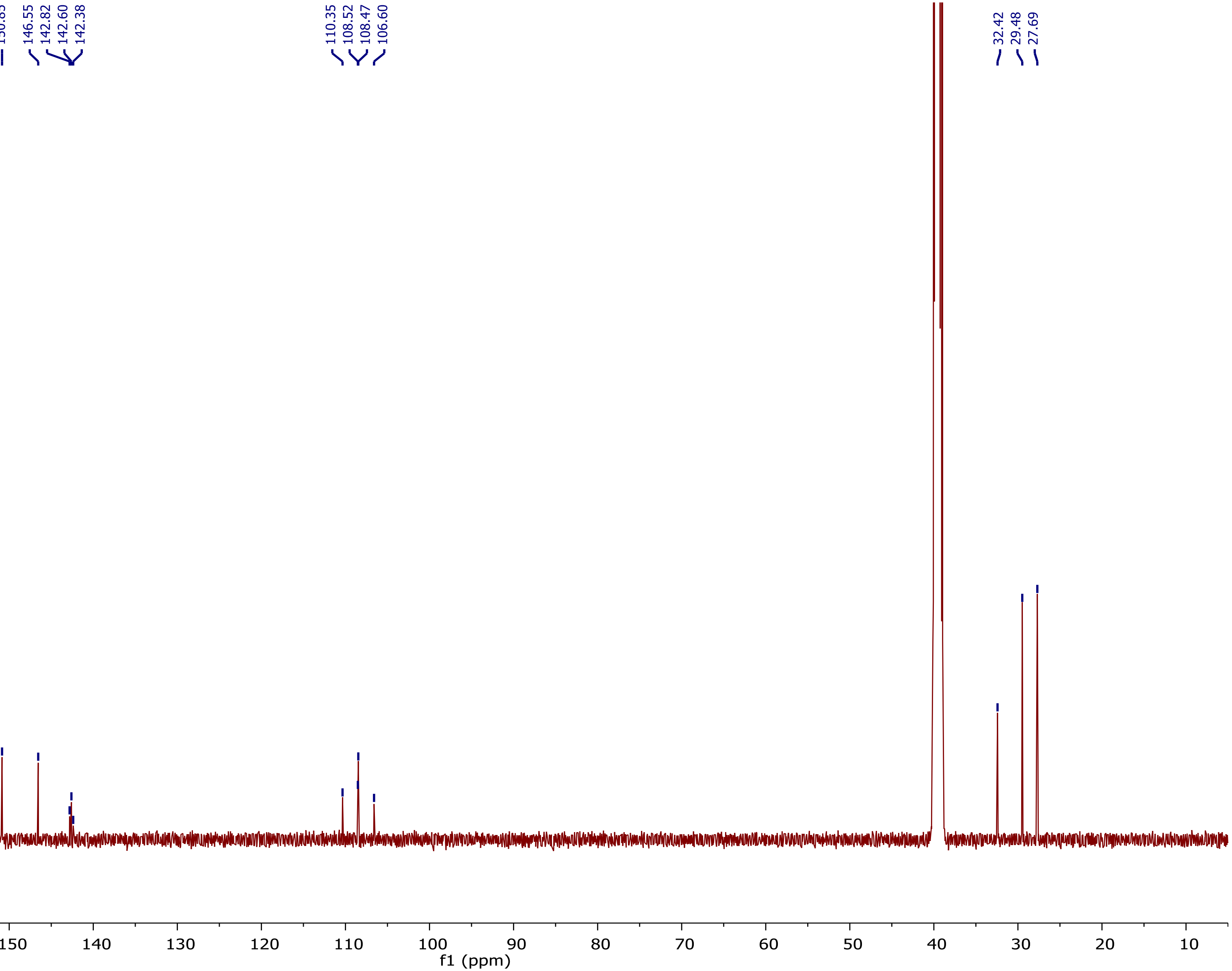
154.85
150.85
146.55
142.82
142.60
142.38
110.35
108.52
108.47
106.60
32.42
29.48
27.69
180
170
160
150
140
130
120
110
100
90
80
70
60
50
40
30
20
10
f1 (ppm)

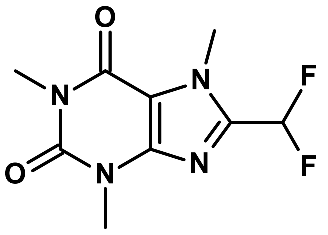
O
N
N
O
N
N
F
F

20

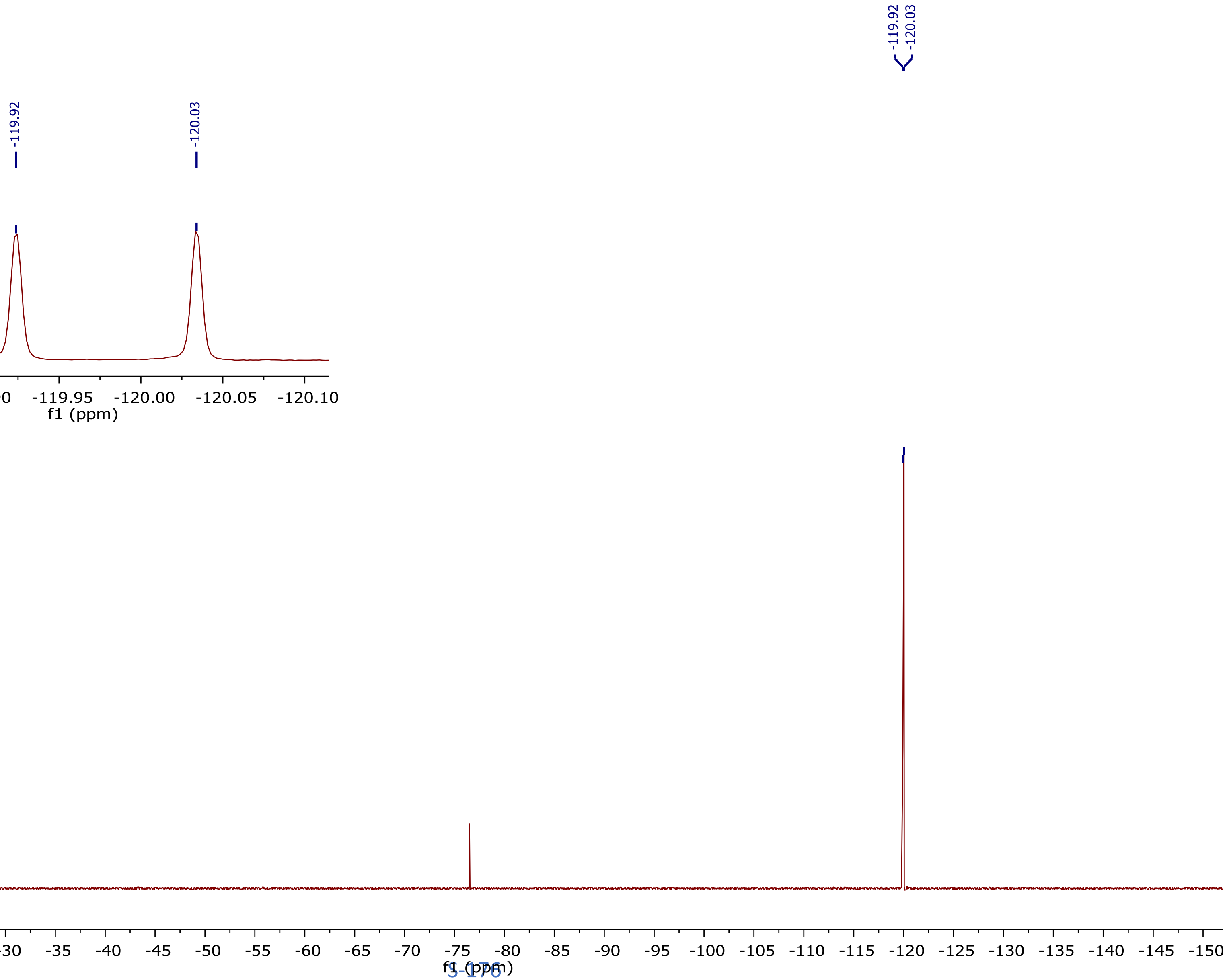
-119.92
-120.03
-119.85
-119.90
-119.95
-120.00
-120.05
-120.10
f1 (ppm)
-5
-10
-15
-20
-25
-30
-35
-40
-45
-50
-55
-60
-65
-70
-75
-80
-85
-90
-95
-100
-105
-110
-115
-120
-125
-130
-135
-140
-145
-150
f1 (ppm)

x. ^{1}H, ^{13}C and ^{19}F NMR of 21

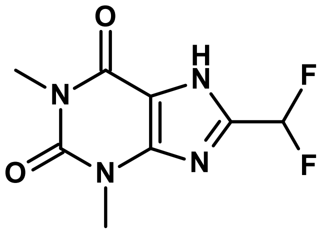

21

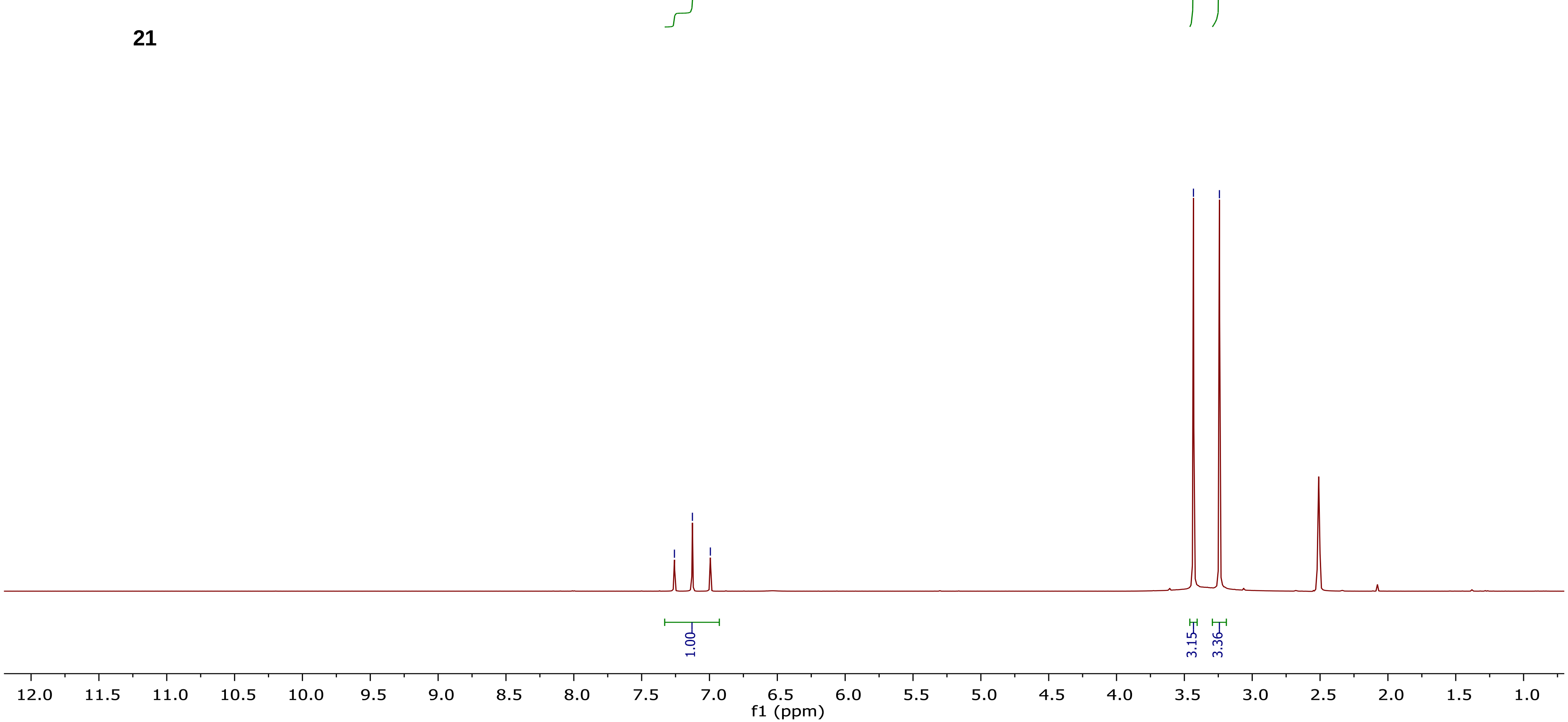
7.26
7.13
6.99
3.43
3.24
1.00
3.15
3.36
12.0 11.5 11.0 10.5 10.0 9.5 9.0 8.5 8.0 7.5 7.0 6.5 6.0 5.5 5.0 4.5 4.0 3.5 3.0 2.5 2.0 1.5 1.0
f1 (ppm)

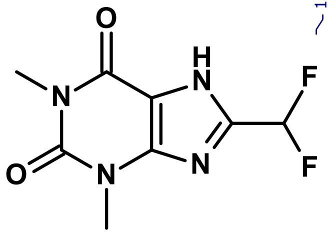
O
H
N
N
F
N
F
O
N

21

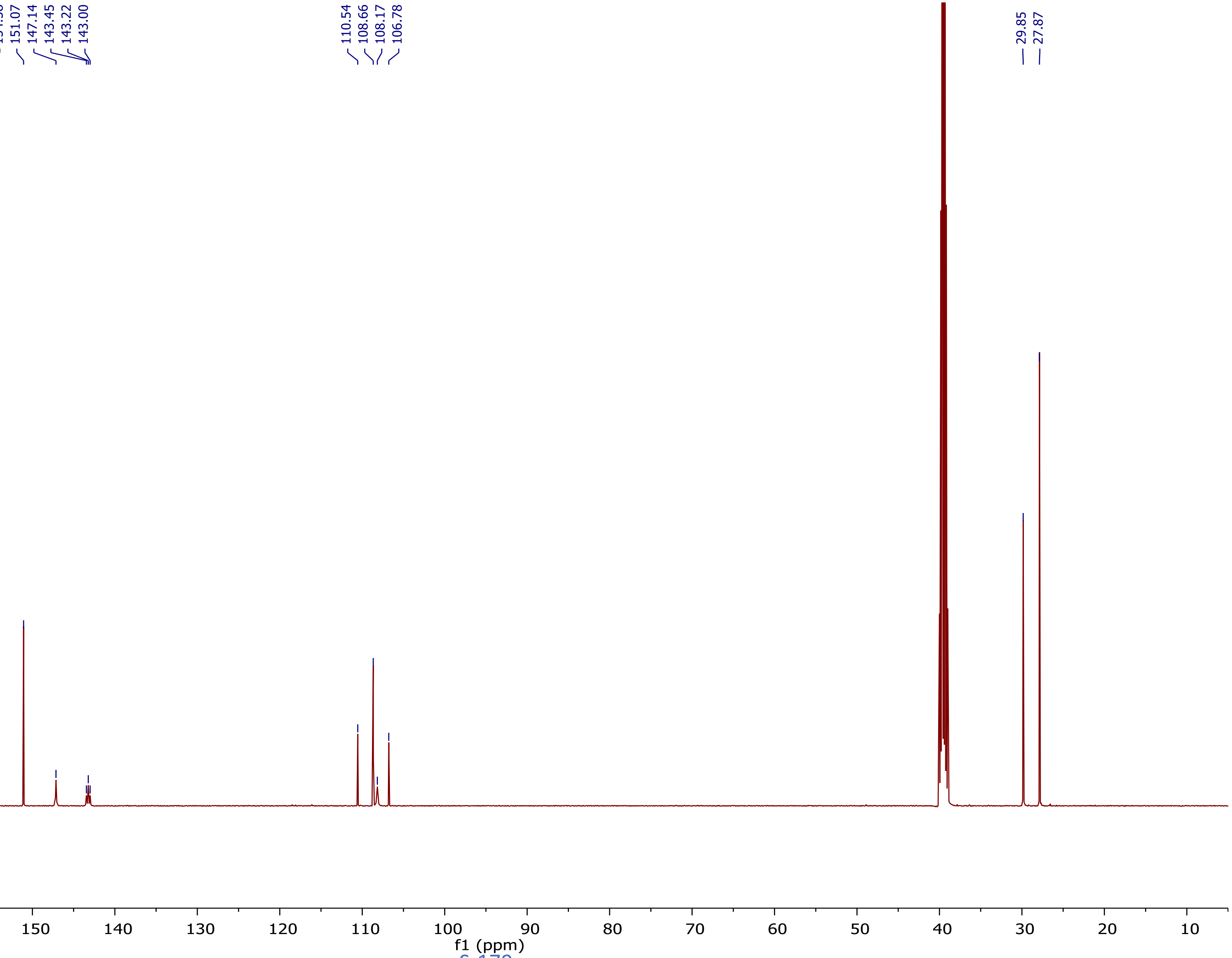
154.58
151.07
147.14
143.45
143.22
143.00
110.54
108.66
108.17
106.78
29.85
27.87
180
170
160
150
140
130
120
110
100
90
80
70
60
50
40
30
20
10
f1 (ppm)

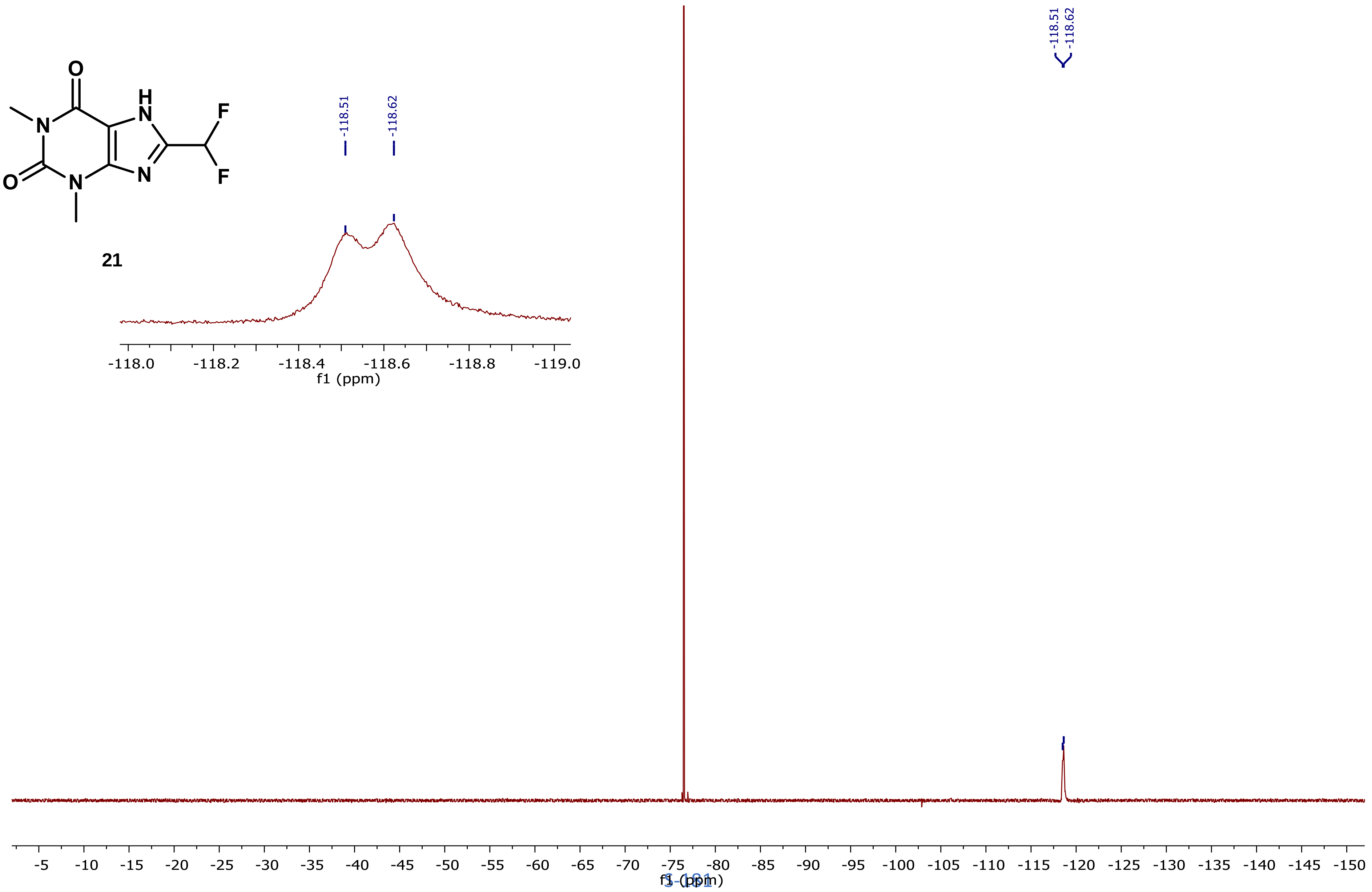
21
-118.51
-118.62
-118.0
-118.2
-118.4
-118.6
-118.8
-119.0
f1 (ppm)
-5 -10 -15 -20 -25 -30 -35 -40 -45 -50 -55 -60 -65 -70 -75 -80 -85 -90 -95 -100 -105 -110 -115 -120 -125 -130 -135 -140 -145 -150
f1 (ppm)

y. ^{1}H, ^{13}C and ^{19}F NMR of 22

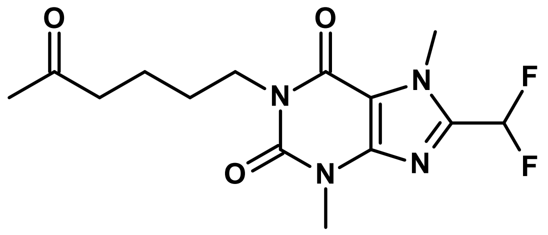

22

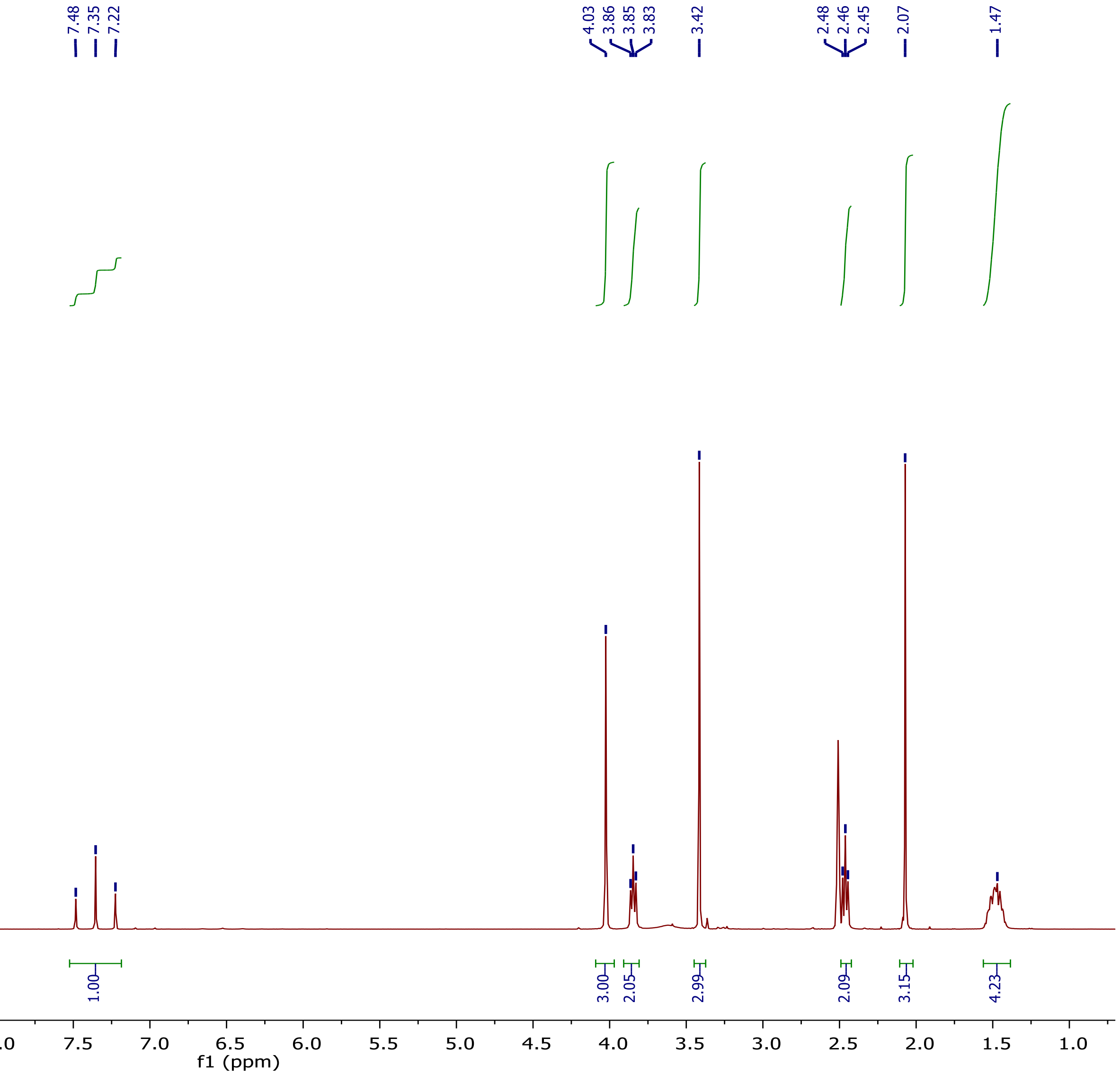
7.48
7.35
7.22
4.03
3.86
3.85
3.83
3.42
2.48
2.46
2.45
2.07
1.47
1.00
3.00
2.05
2.99
2.09
3.15
4.23
12.0
11.5
11.0
10.5
10.0
9.5
9.0
8.5
8.0
7.5
7.0
6.5
6.0
5.5
5.0
4.5
4.0
3.5
3.0
2.5
2.0
1.5
1.0
f1 (ppm)

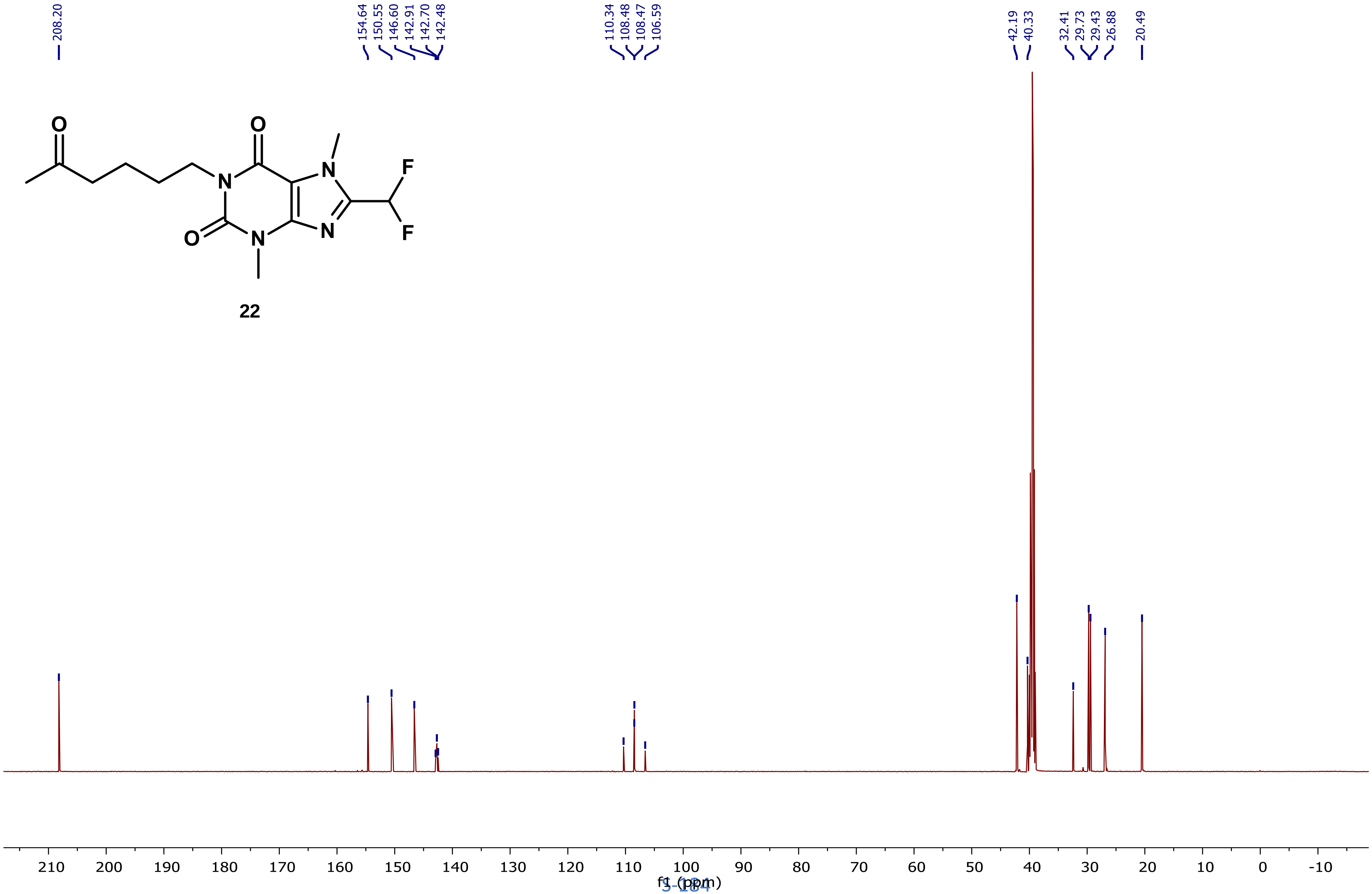
208.20
154.64
150.55
146.60
142.91
142.70
142.48
110.34
108.48
108.47
106.59
42.19
40.33
32.41
29.73
29.43
26.88
20.49
O
O
N
N
O
N
N
F
F
210
200
190
180
170
160
150
140
130
120
110
100
90
80
70
60
50
40
30
20
10
0
-10
f1 (ppm)

22

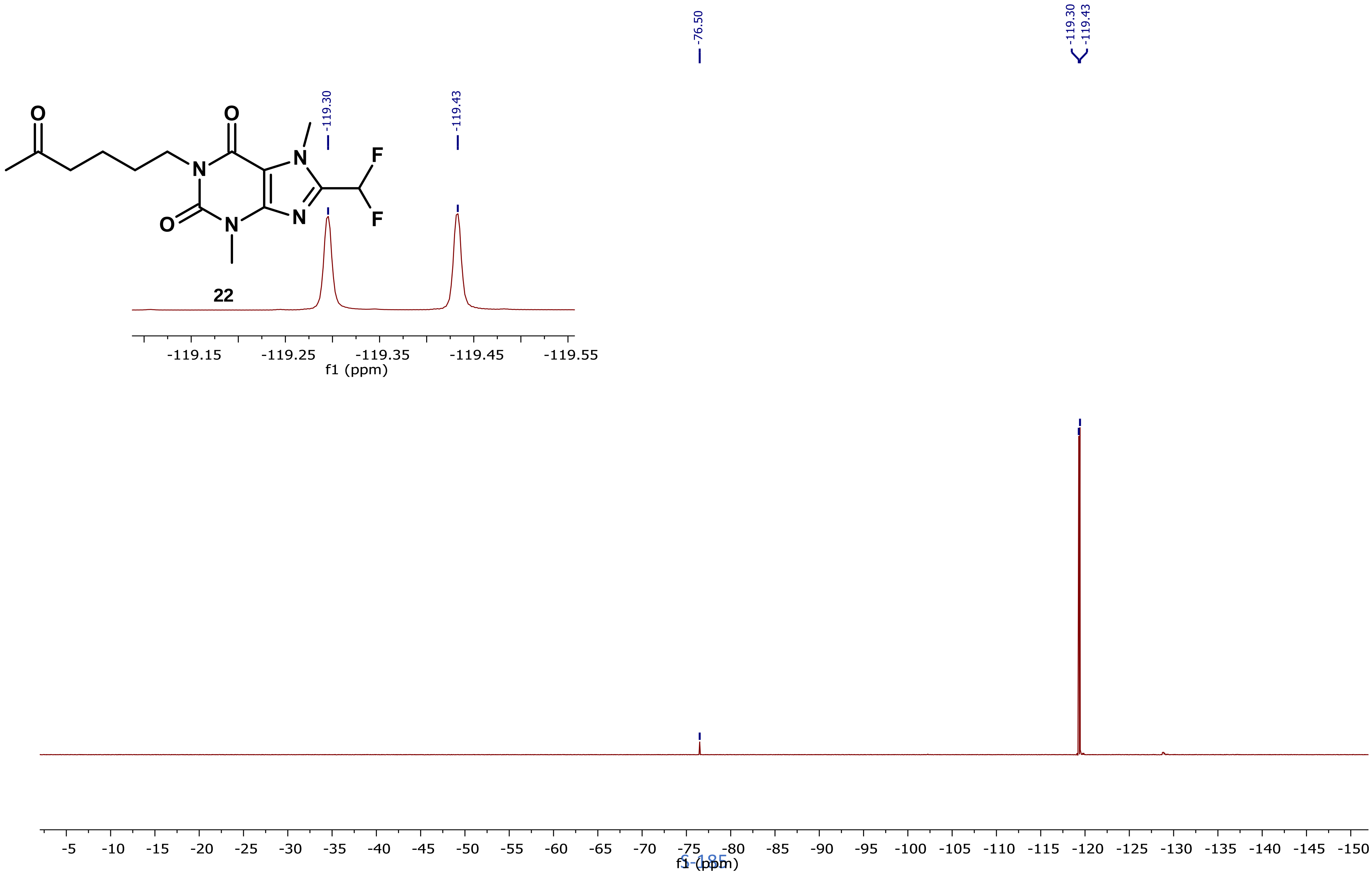
-76.50
-119.30
-119.43
22
-119.15 -119.25 -119.35 -119.45 -119.55
f1 (ppm)
-5 -10 -15 -20 -25 -30 -35 -40 -45 -50 -55 -60 -65 -70 -75 -80 -85 -90 -95 -100 -105 -110 -115 -120 -125 -130 -135 -140 -145 -150
f1 (ppm)

z. ^{1}H, ^{13}C and ^{19}F NMR of 23

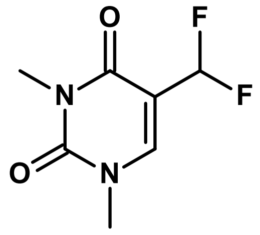

23

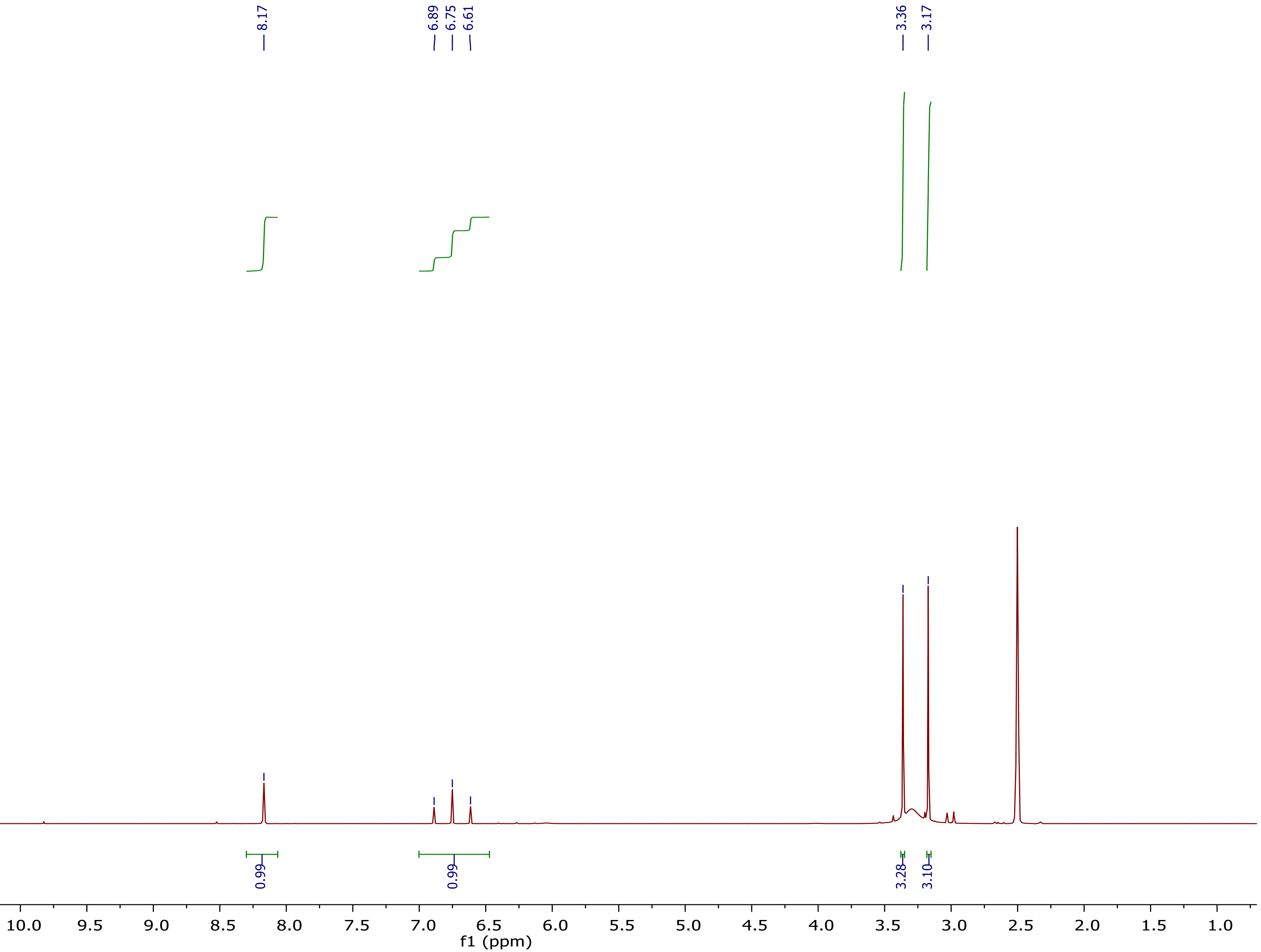
8.17
6.89
6.75
6.61
3.36
3.17
0.99
0.99
3.28
3.10
12.0
11.5
11.0
10.5
10.0
9.5
9.0
8.5
8.0
7.5
7.0
6.5
6.0
5.5
5.0
4.5
4.0
3.5
3.0
2.5
2.0
1.5
1.0
f1 (ppm)

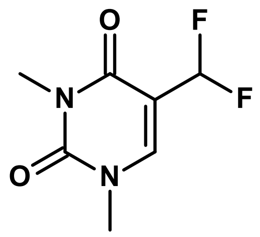

23

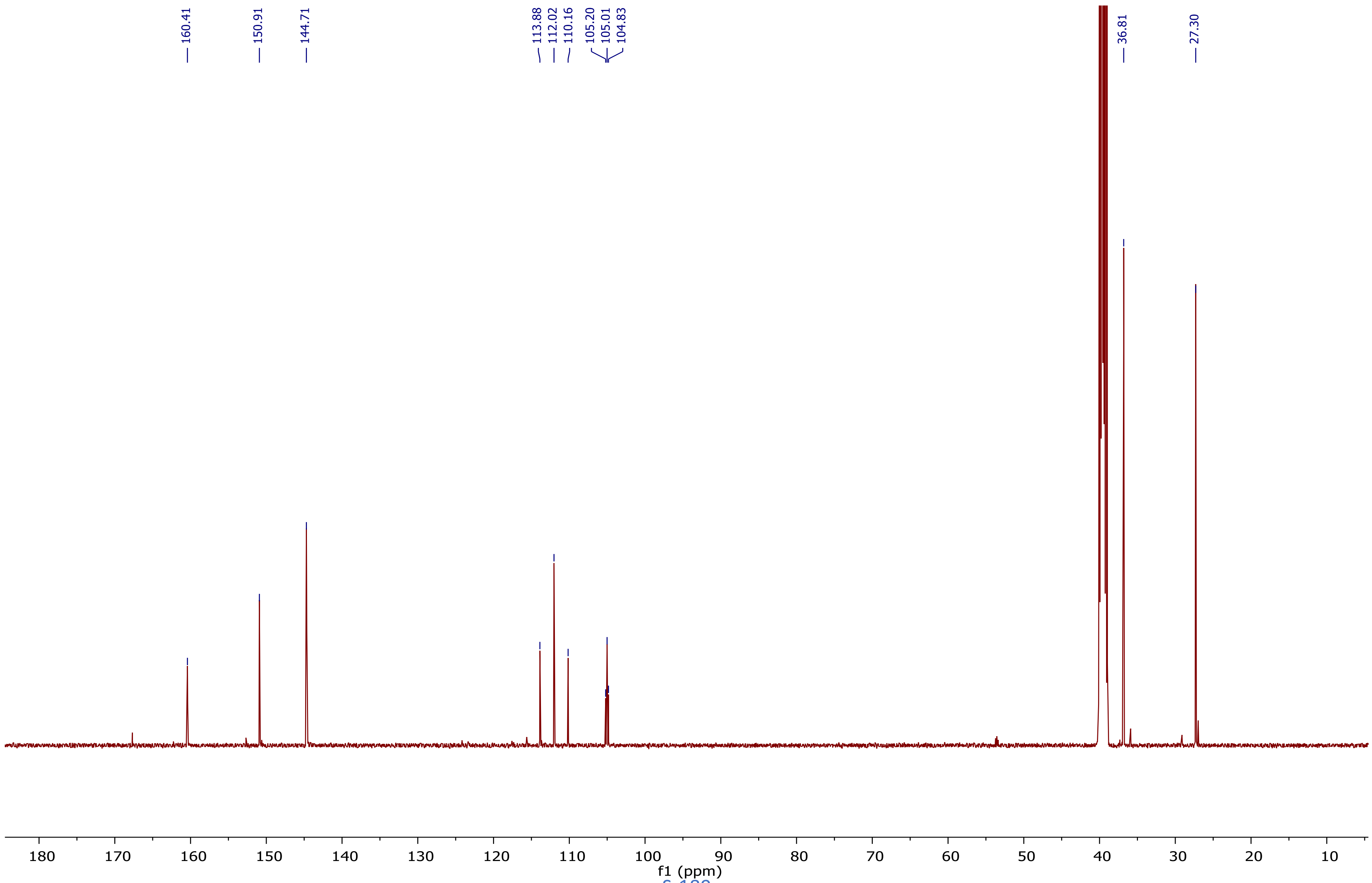
160.41
150.91
144.71
113.88
112.02
110.16
105.20
105.01
104.83
36.81
27.30
180
170
160
150
140
130
120
110
100
90
80
70
60
50
40
30
20
10
f1 (ppm)

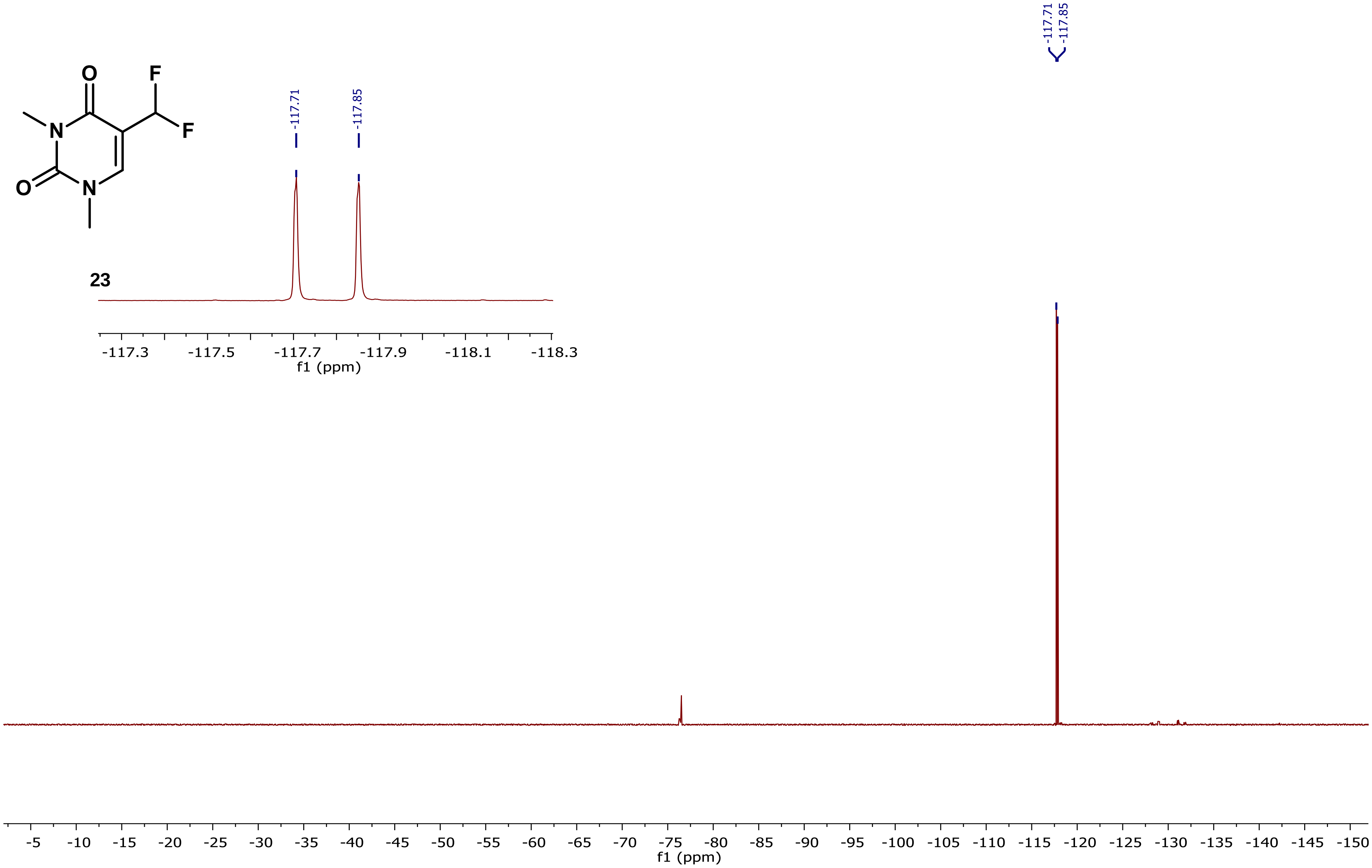
23
-117.71
-117.85
-117.3
-117.5
-117.7
-117.9
-118.1
-118.3
f1 (ppm)
-5 -10 -15 -20 -25 -30 -35 -40 -45 -50 -55 -60 -65 -70 -75 -80 -85 -90 -95 -100 -105 -110 -115 -120 -125 -130 -135 -140 -145 -150
f1 (ppm)

aa. ^{1}H, HSQC, HMBC and ^{19}F NMR of 24a

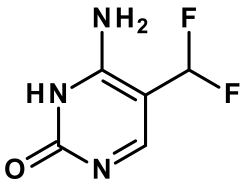

24a

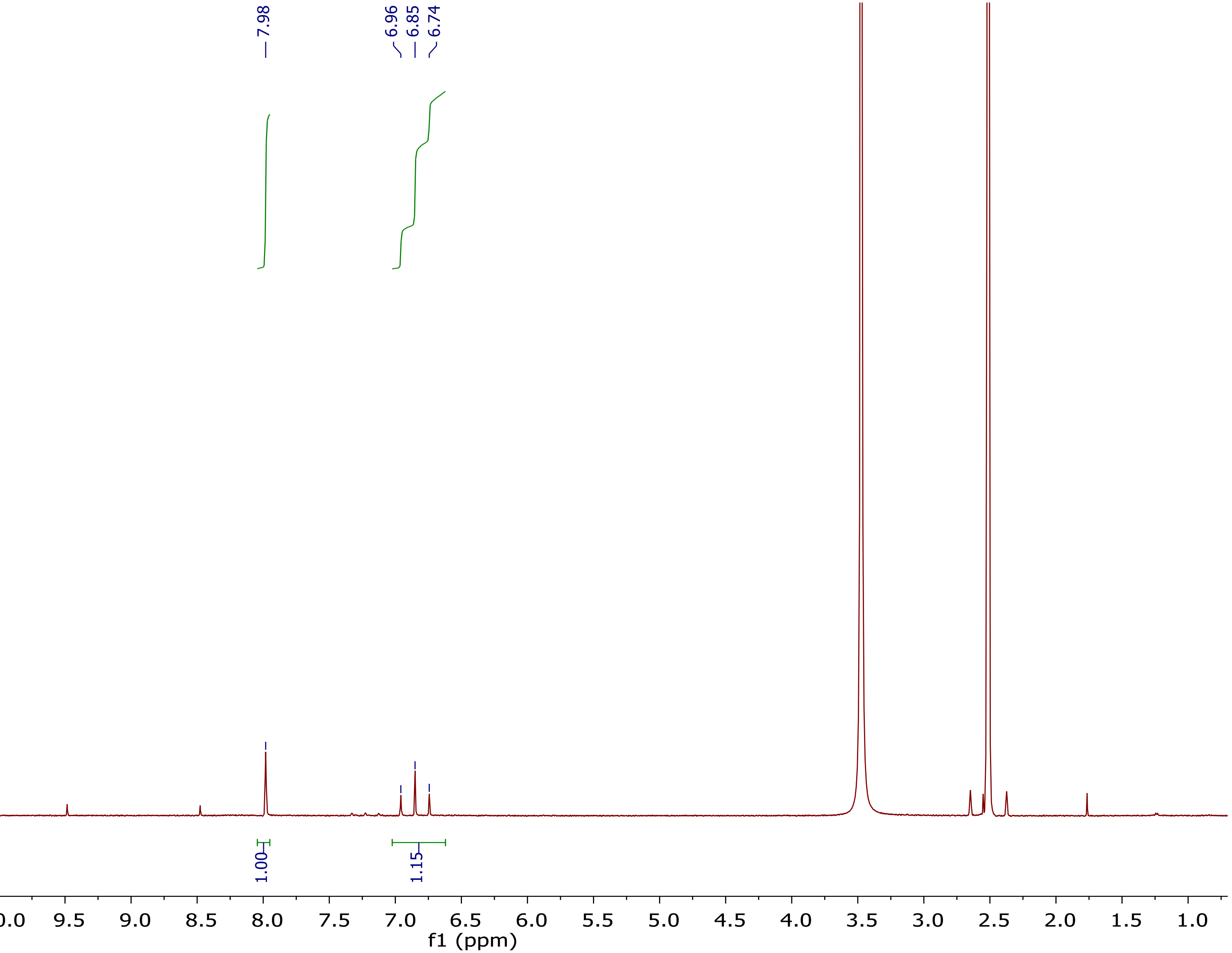
7.98
6.96
6.85
6.74
1.00
1.15
12.0 11.5 11.0 10.5 10.0 9.5 9.0 8.5 8.0 7.5 7.0 6.5 6.0 5.5 5.0 4.5 4.0 3.5 3.0 2.5 2.0 1.5 1.0
f1 (ppm)

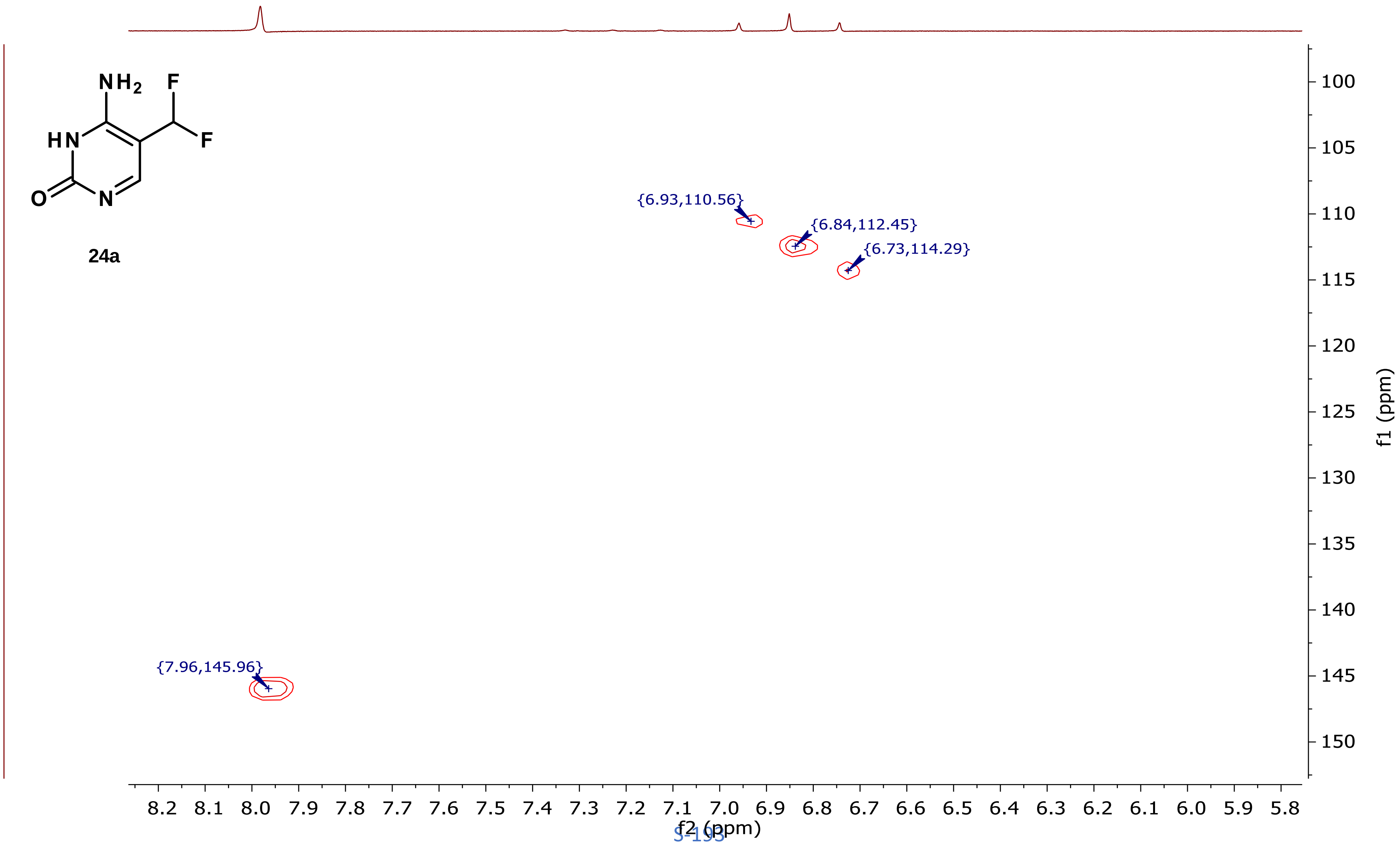
24a
{6.93,110.56}
{6.84,112.45}
{6.73,114.29}
{7.96,145.96}
f1 (ppm)
f2 (ppm)

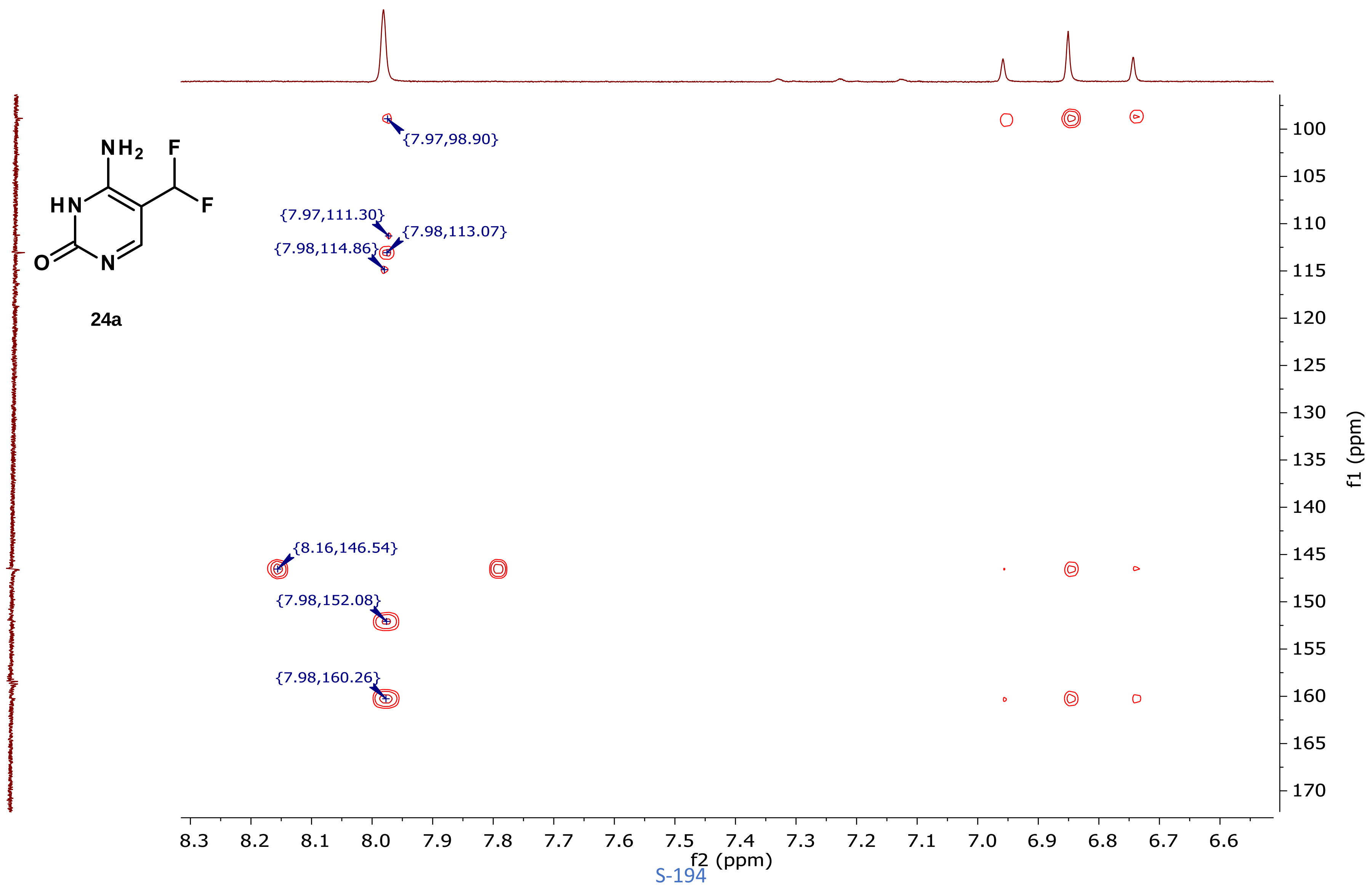
NH2
F
HN
F
O
N
24a
{7.97,98.90}
{7.97,111.30}
{7.98,113.07}
{7.98,114.86}
{8.16,146.54}
{7.98,152.08}
{7.98,160.26}
100
105
110
115
120
125
130
135
140
145
150
155
160
165
170
f1 (ppm)
8.3
8.2
8.1
8.0
7.9
7.8
7.7
7.6
7.5
7.4
7.3
7.2
7.1
7.0
6.9
6.8
6.7
6.6
f2 (ppm)

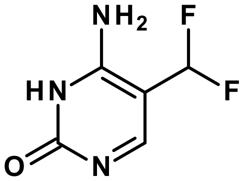

24a

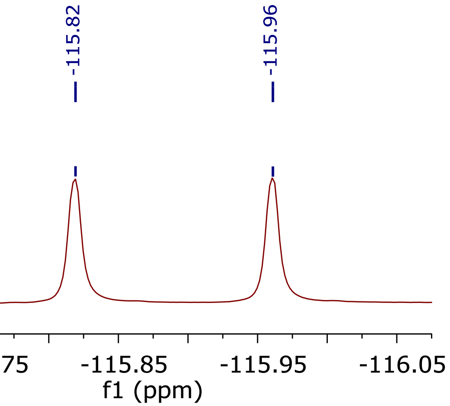
-115.82
-115.96
-115.75
-115.85
-115.95
-116.05
f1 (ppm)

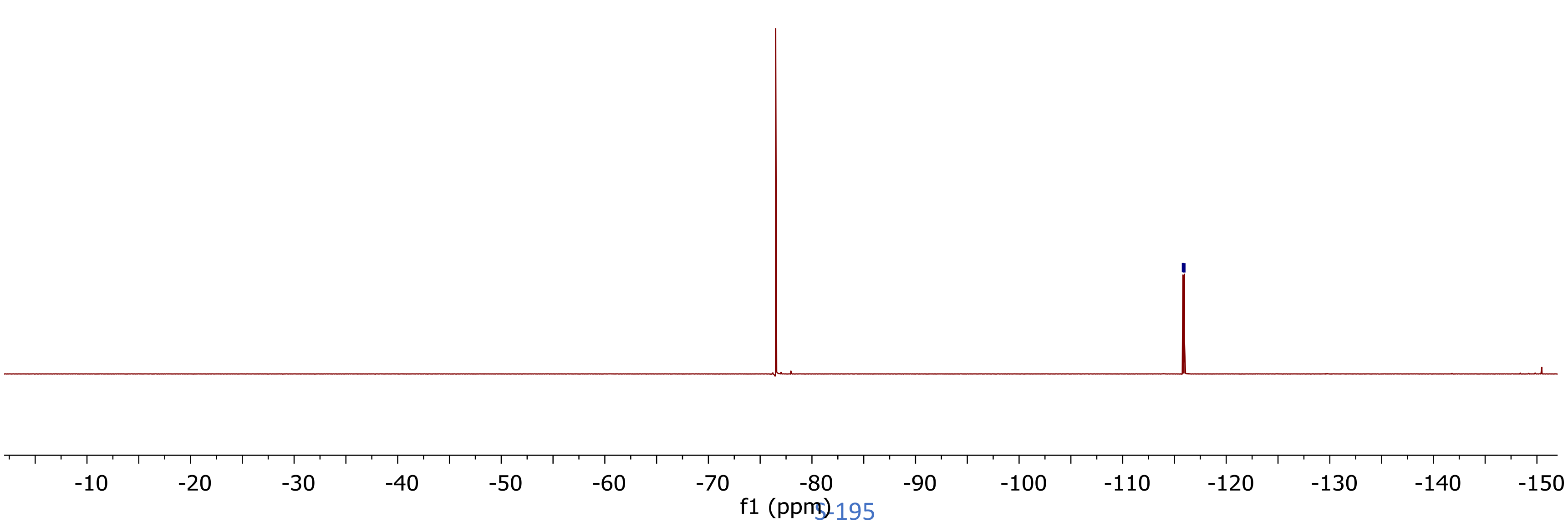
-115.82
-115.96
-10
-20
-30
-40
-50
-60
-70
-80
-90
-100
-110
-120
-130
-140
-150
f1 (ppm)

bb. ^{1}H, ^{13}C and ^{19}F NMR of 24b

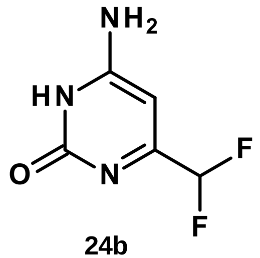

24b

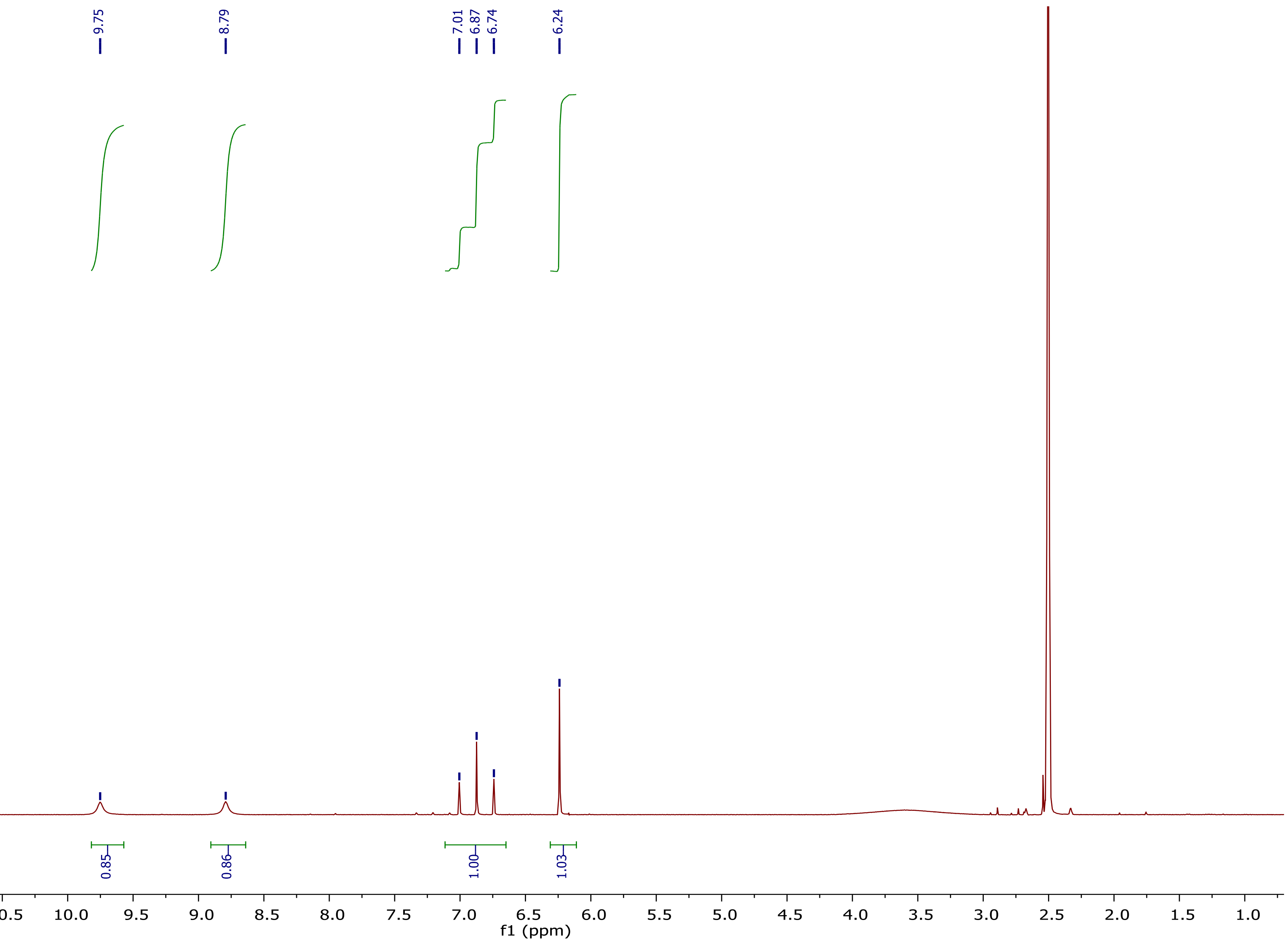
9.75
8.79
7.01
6.87
6.74
6.24
0.85
0.86
1.00
1.03
12.0
11.5
11.0
10.5
10.0
9.5
9.0
8.5
8.0
7.5
7.0
6.5
6.0
5.5
5.0
4.5
4.0
3.5
3.0
2.5
2.0
1.5
1.0
f1 (ppm)

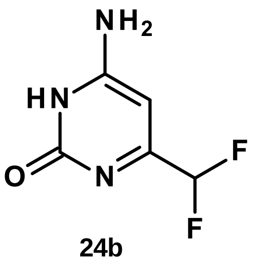

24b

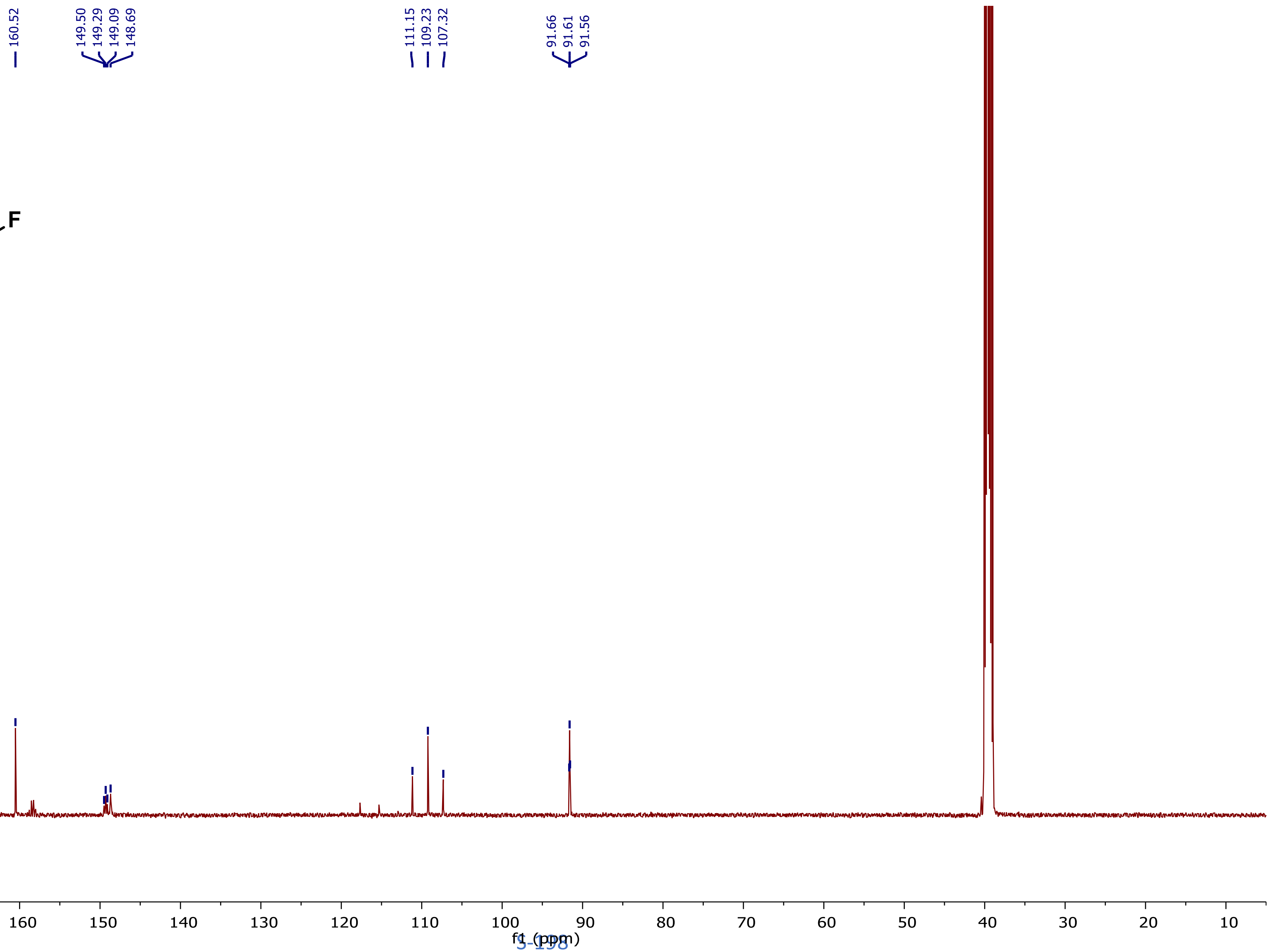
160.52
149.50
149.29
149.09
148.69
111.15
109.23
107.32
91.66
91.61
91.56
180
170
160
150
140
130
120
110
100
90
80
70
60
50
40
30
20
10
f1 (ppm)

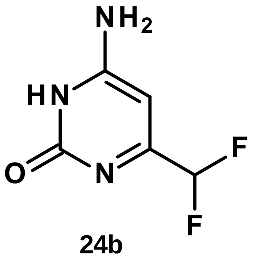
NH2
HN
O
N
F
F

24b

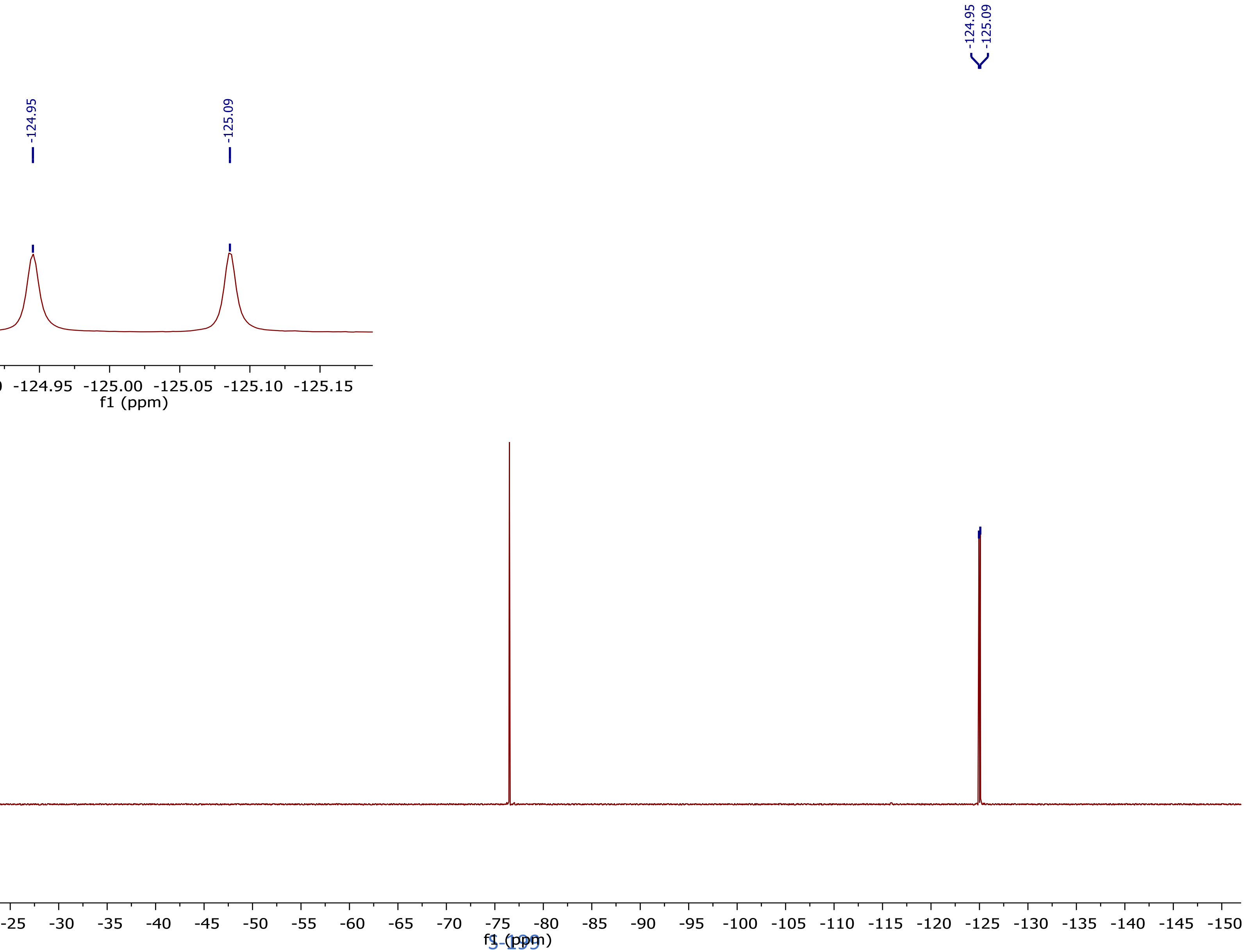
-124.95
-125.09
-124.85 -124.90 -124.95 -125.00 -125.05 -125.10 -125.15
f1 (ppm)
-5 -10 -15 -20 -25 -30 -35 -40 -45 -50 -55 -60 -65 -70 -75 -80 -85 -90 -95 -100 -105 -110 -115 -120 -125 -130 -135 -140 -145 -150
f1 (ppm)

cc. ^{1}H, ^{13}C and ^{19}F NMR of 25

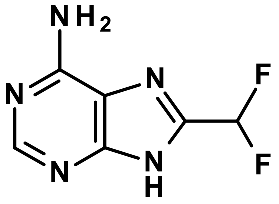
NH2
N
N
N
N
H
F
F

25

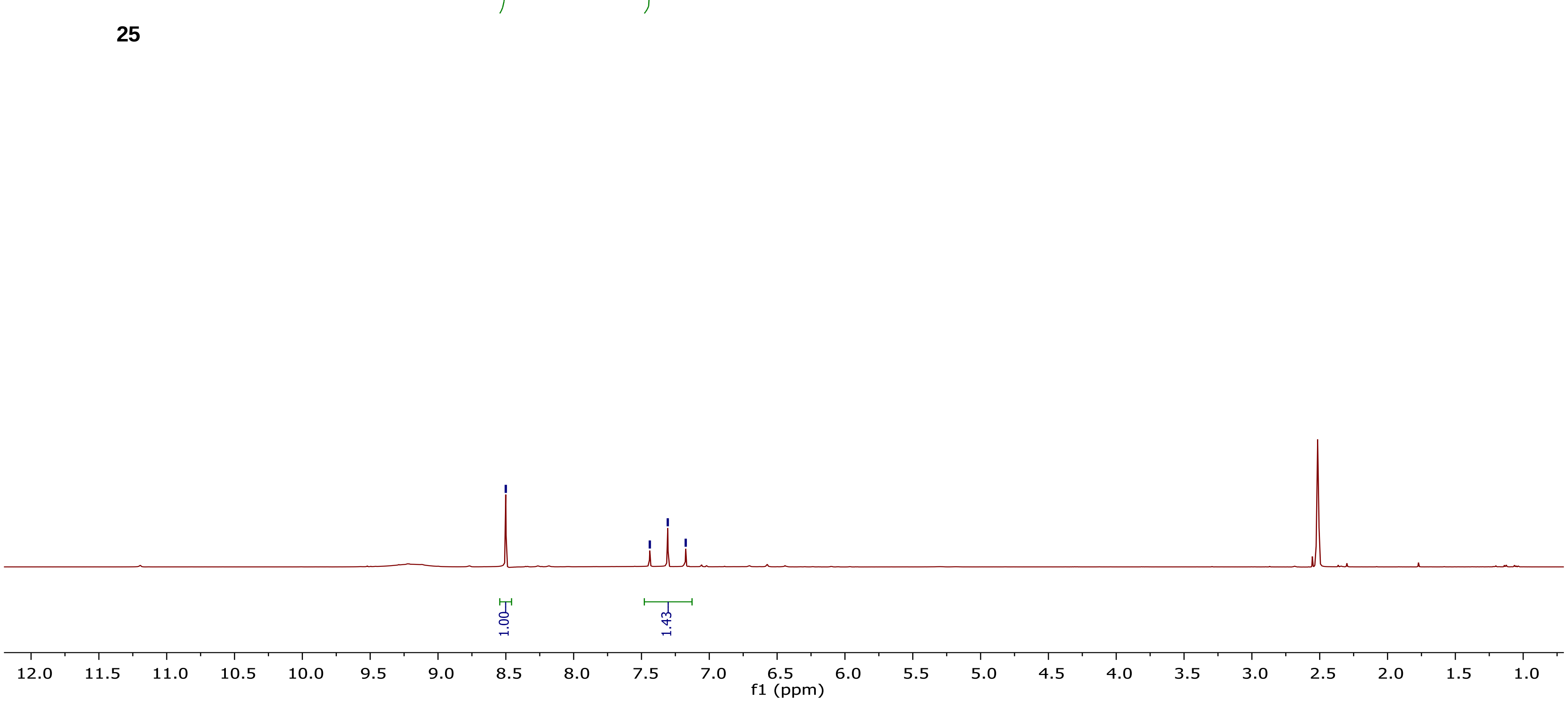
8.50
7.44
7.31
7.17
1.00
1.43
12.0
11.5
11.0
10.5
10.0
9.5
9.0
8.5
8.0
7.5
7.0
6.5
6.0
5.5
5.0
4.5
4.0
3.5
3.0
2.5
2.0
1.5
1.0
f1 (ppm)

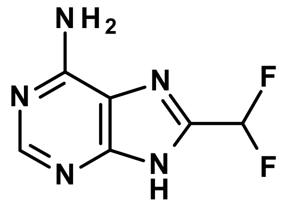
NH2
N
N
F
N
N
H
F

25

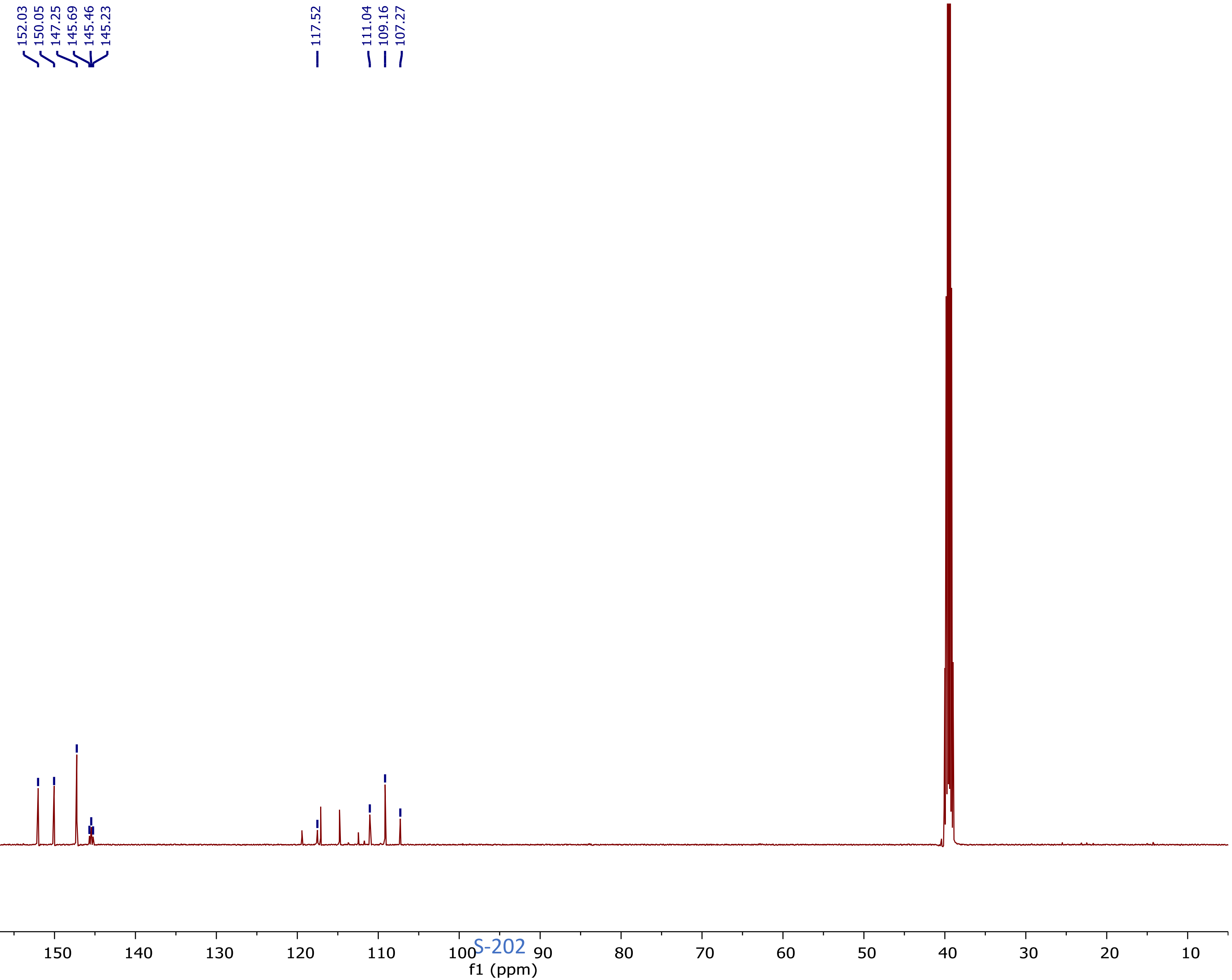
152.03
150.05
147.25
145.69
145.46
145.23
117.52
111.04
109.16
107.27
180
170
160
150
140
130
120
110
100
90
80
70
60
50
40
30
20
10
f1 (ppm)

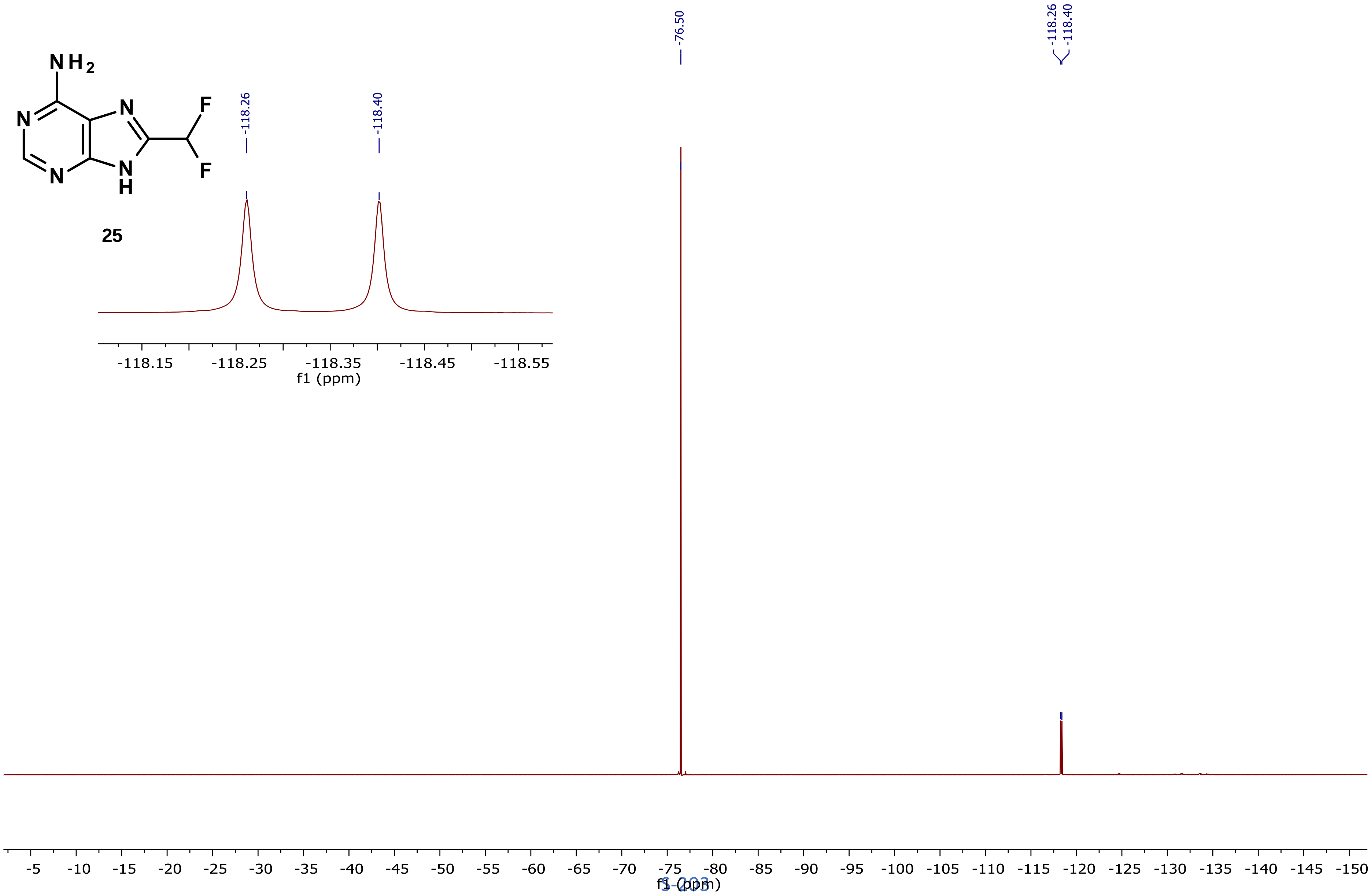
NH2
N
N
N
N
H
F
F
25
-76.50
-118.26
-118.40
-118.15
-118.25
-118.35
-118.45
-118.55
f1 (ppm)
-5 -10 -15 -20 -25 -30 -35 -40 -45 -50 -55 -60 -65 -70 -75 -80 -85 -90 -95 -100 -105 -110 -115 -120 -125 -130 -135 -140 -145 -150
f1 (ppm)

dd. ^{1}H, ^{13}C and ^{19}F NMR of 26

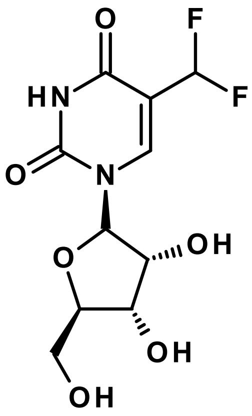

26

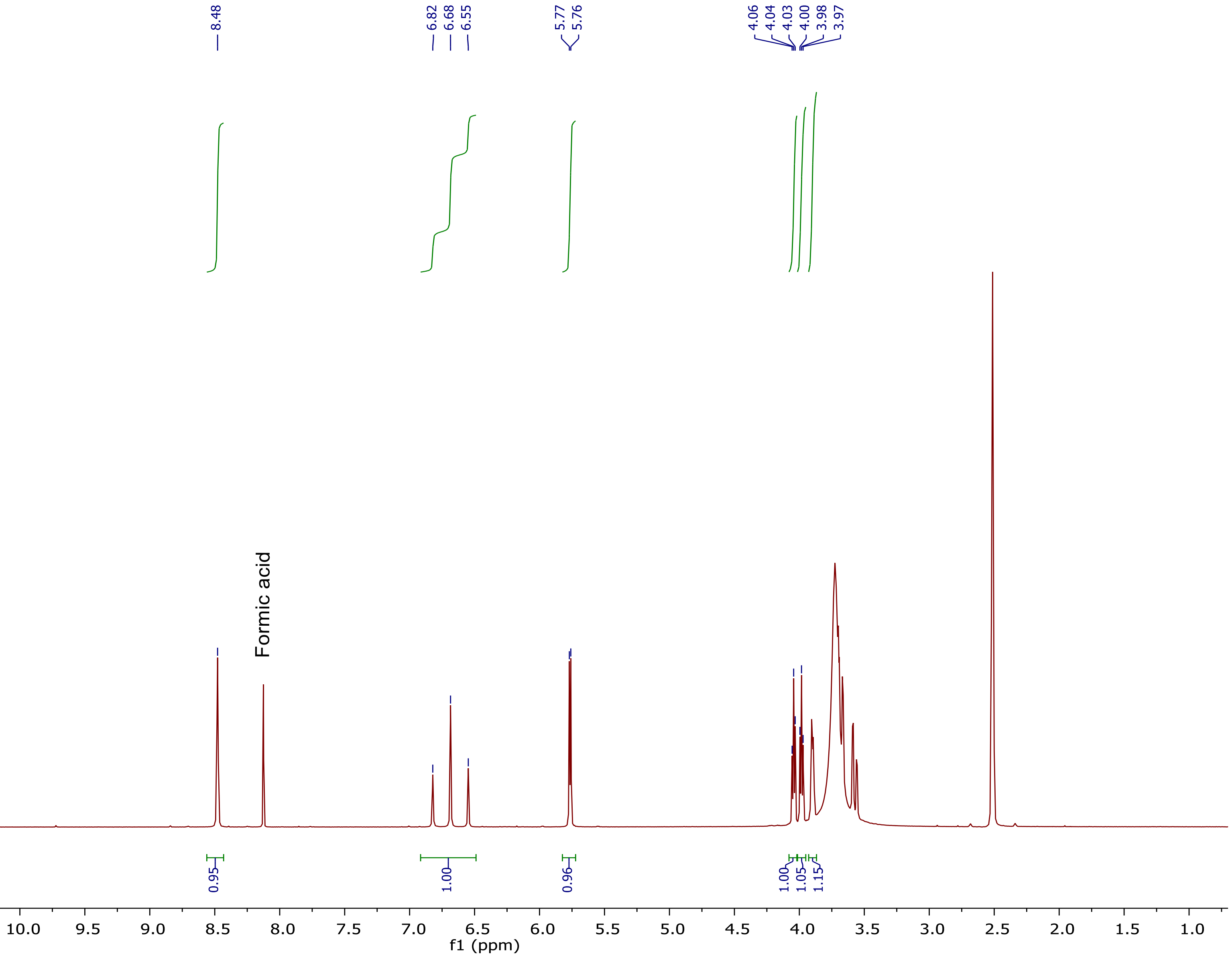
8.48
6.82
6.68
6.55
5.77
5.76
4.06
4.04
4.03
4.00
3.98
3.97
Formic acid
0.95
1.00
0.96
1.00
1.05
1.15
12.0 11.5 11.0 10.5 10.0 9.5 9.0 8.5 8.0 7.5 7.0 6.5 6.0 5.5 5.0 4.5 4.0 3.5 3.0 2.5 2.0 1.5 1.0
f1 (ppm)

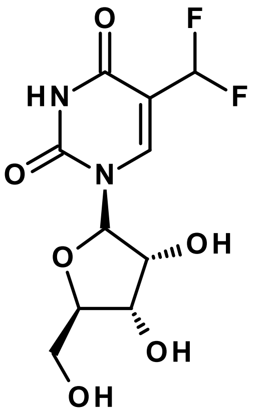

26

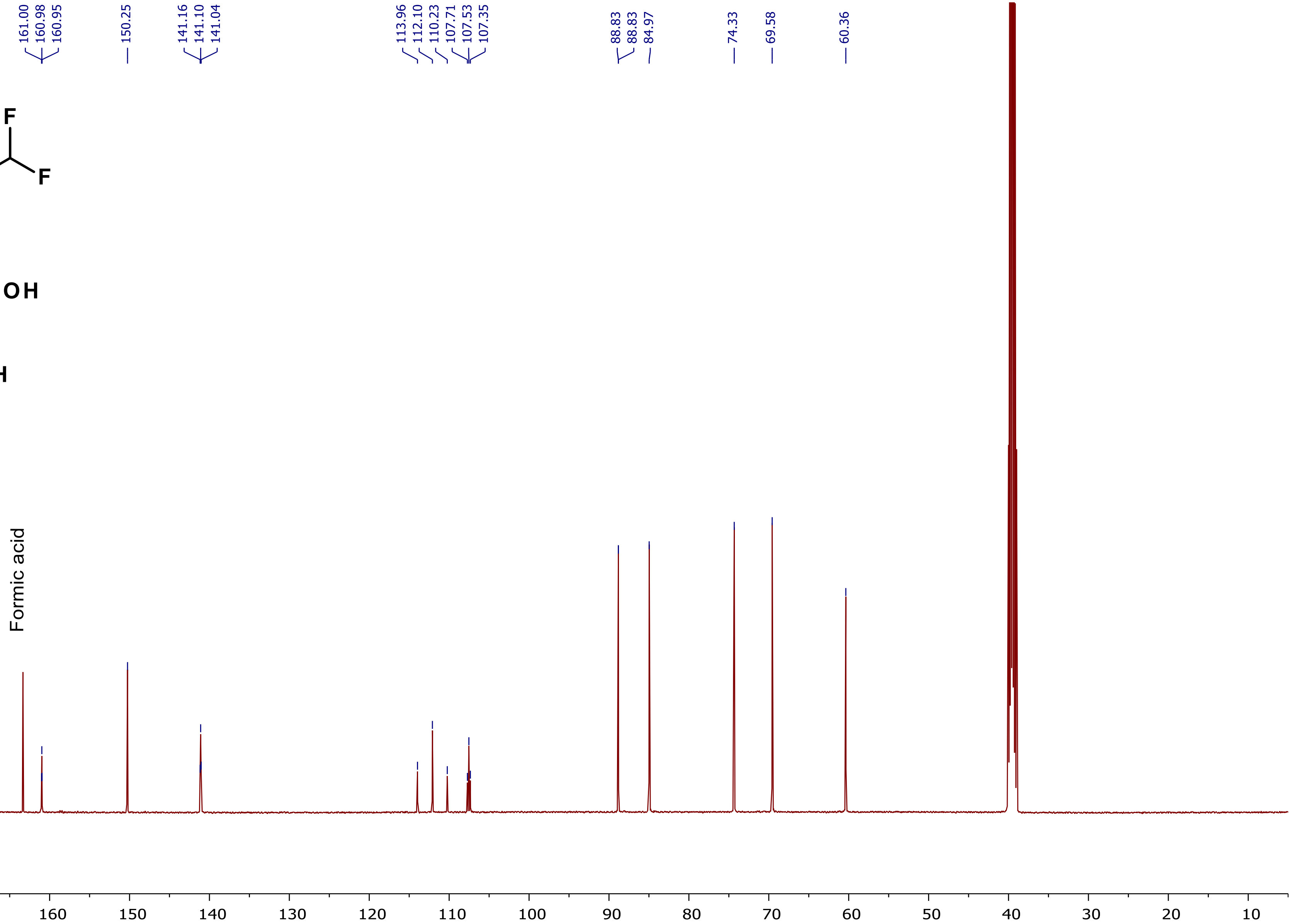
161.00
160.98
160.95
150.25
141.16
141.10
141.04
113.96
112.10
110.23
107.71
107.53
107.35
88.83
88.83
84.97
74.33
69.58
60.36
Formic acid
180
170
160
150
140
130
120
110
100
90
80
70
60
50
40
30
20
10
f1 (ppm)

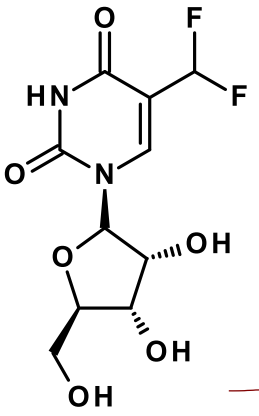

26

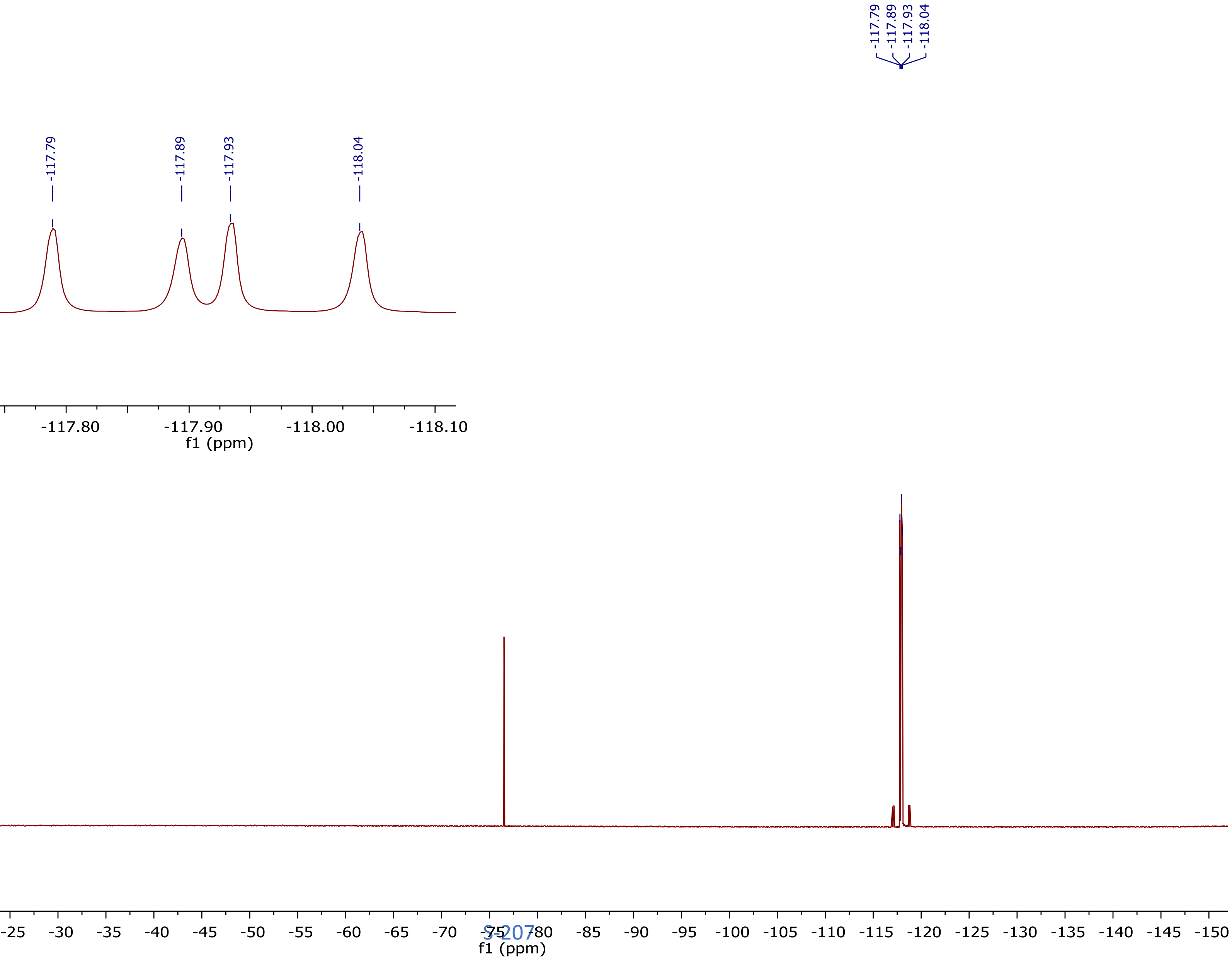
-117.79
-117.89
-117.93
-118.04
-117.80
-117.90
-118.00
-118.10
f1 (ppm)
-5
-10
-15
-20
-25
-30
-35
-40
-45
-50
-55
-60
-65
-70
-75
-80
-85
-90
-95
-100
-105
-110
-115
-120
-125
-130
-135
-140
-145
-150

ee. ^{1}H, HSQC/HMBC and ^{19}F NMR of 27

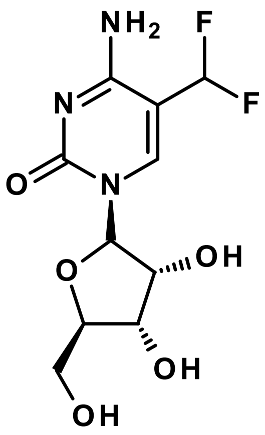

27

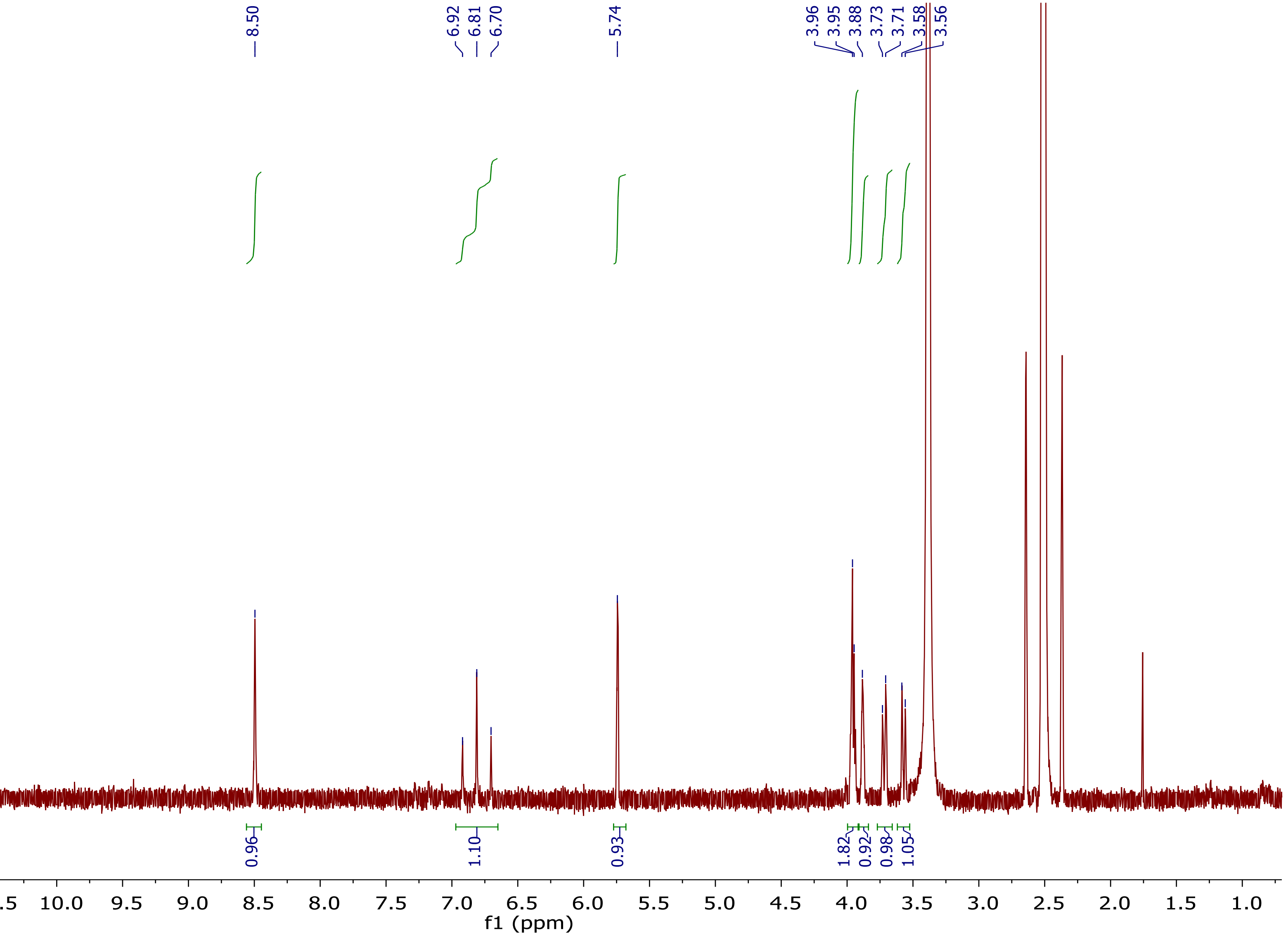
8.50
6.92
6.81
6.70
5.74
3.96
3.95
3.88
3.73
3.71
3.58
3.56
0.96
1.10
0.93
1.82
0.92
0.98
1.05
12.0 11.5 11.0 10.5 10.0 9.5 9.0 8.5 8.0 7.5 7.0 6.5 6.0 5.5 5.0 4.5 4.0 3.5 3.0 2.5 2.0 1.5 1.0
f1 (ppm)

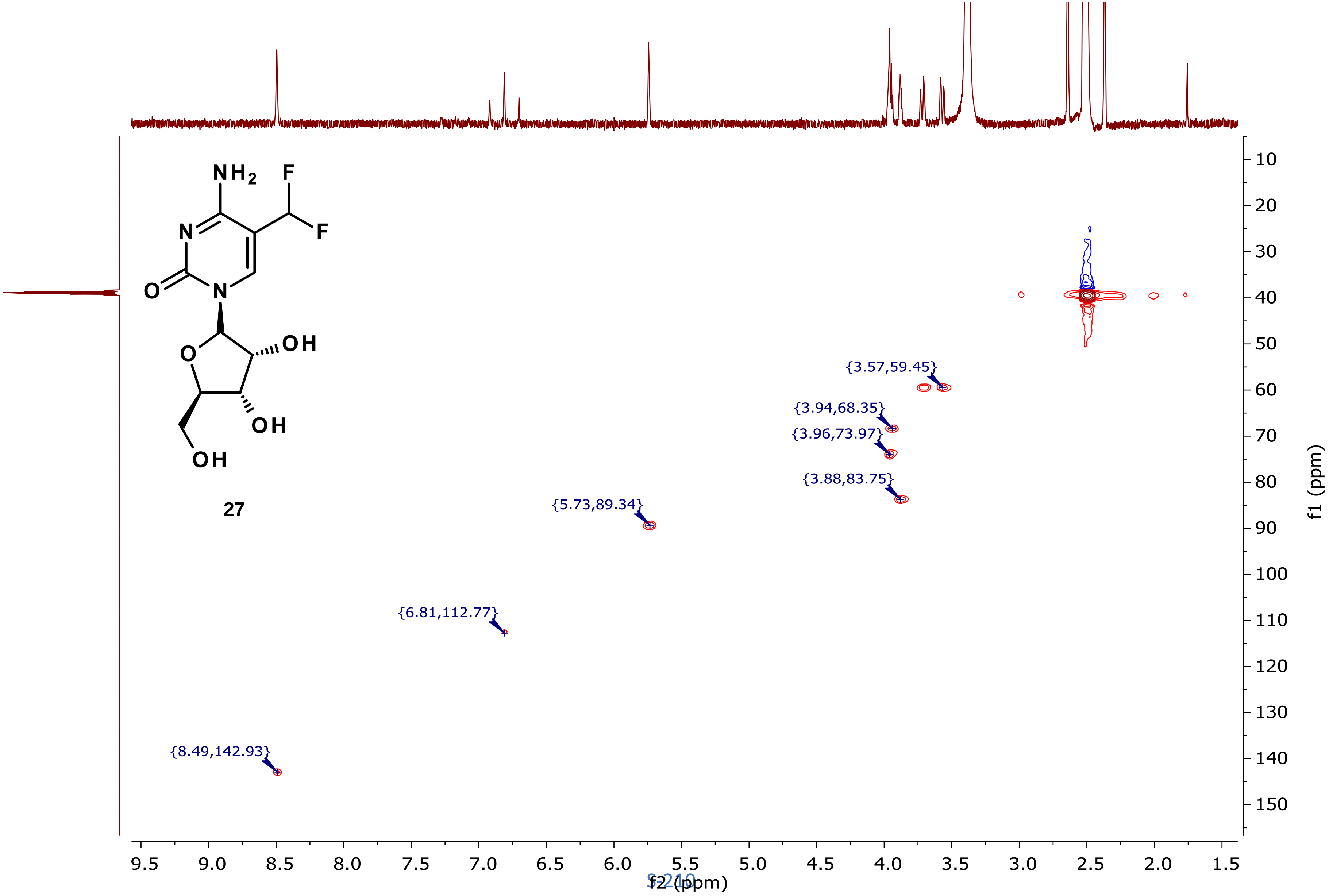
27
{3.57,59.45}
{3.94,68.35}
{3.96,73.97}
{3.88,83.75}
{5.73,89.34}
{6.81,112.77}
{8.49,142.93}
f2 (ppm)
f1 (ppm)

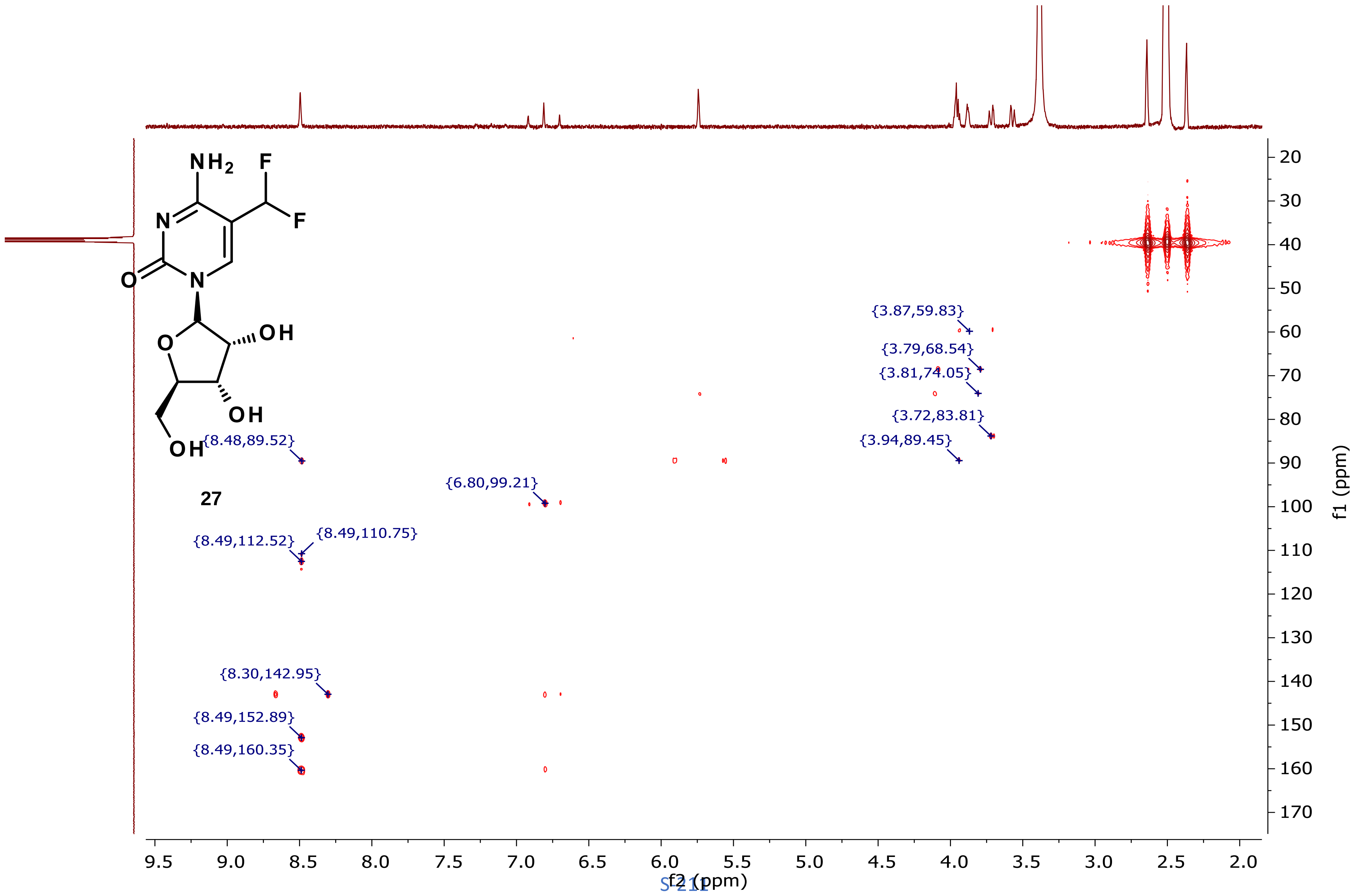
NH2
F
F
N
O
N
O
OH
OH
OH
27
{3.87,59.83}
{3.79,68.54}
{3.81,74.05}
{3.72,83.81}
{3.94,89.45}
{8.48,89.52}
{6.80,99.21}
{8.49,112.52}
{8.49,110.75}
{8.30,142.95}
{8.49,152.89}
{8.49,160.35}
f1 (ppm)
f2 (ppm)

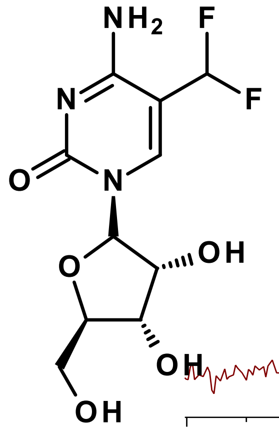

27

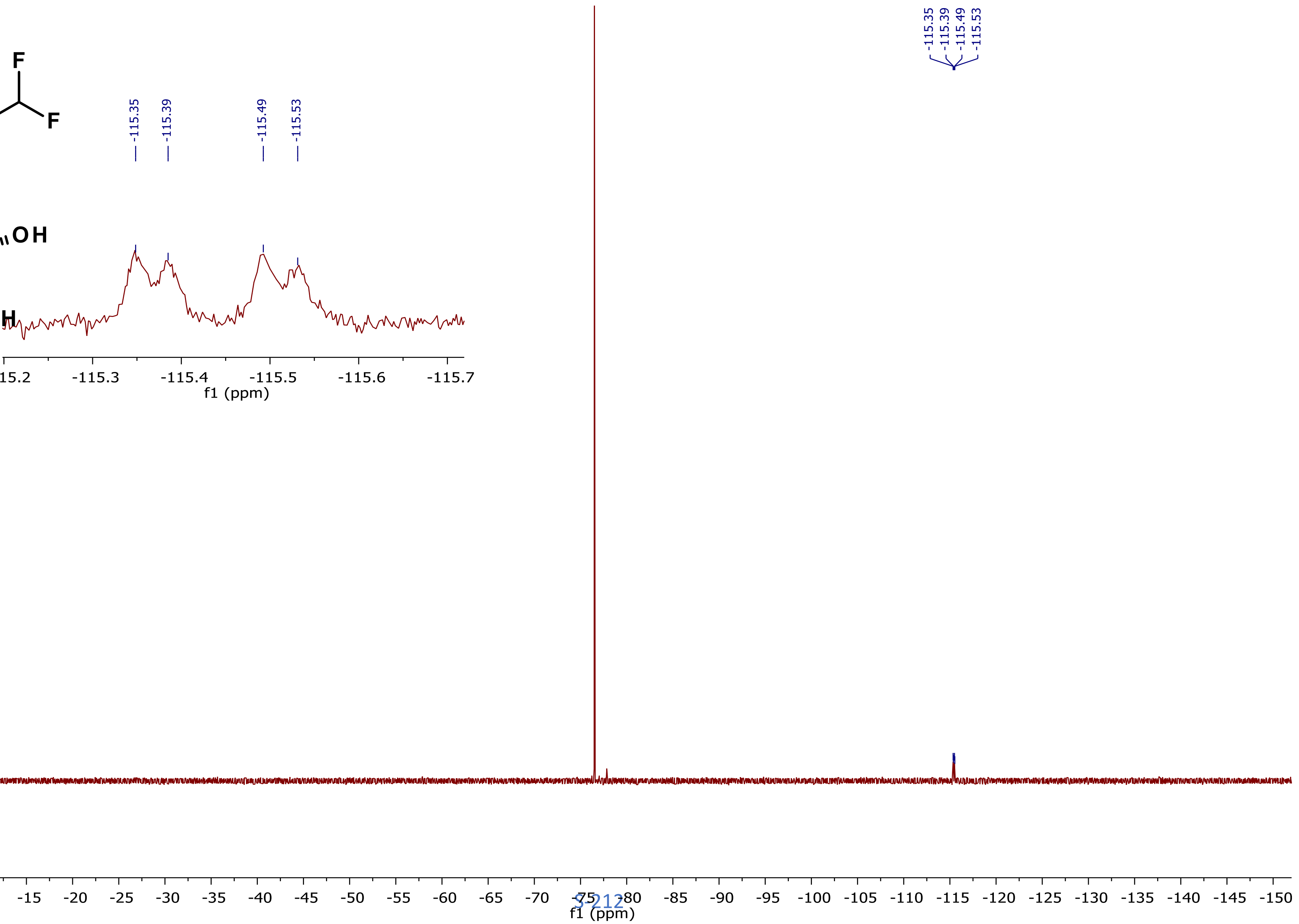
-115.35
-115.39
-115.49
-115.53
-115.2
-115.3
-115.4
-115.5
-115.6
-115.7
f1 (ppm)
-5
-10
-15
-20
-25
-30
-35
-40
-45
-50
-55
-60
-65
-70
-75
-80
-85
-90
-95
-100
-105
-110
-115
-120
-125
-130
-135
-140
-145
-150

ff. ^{1}H, ^{13}C and ^{19}F NMR of 28

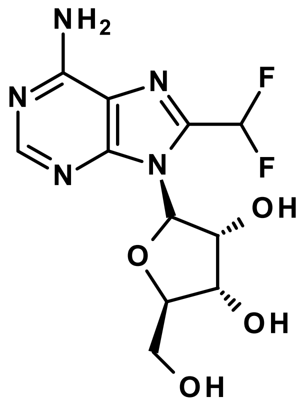

28

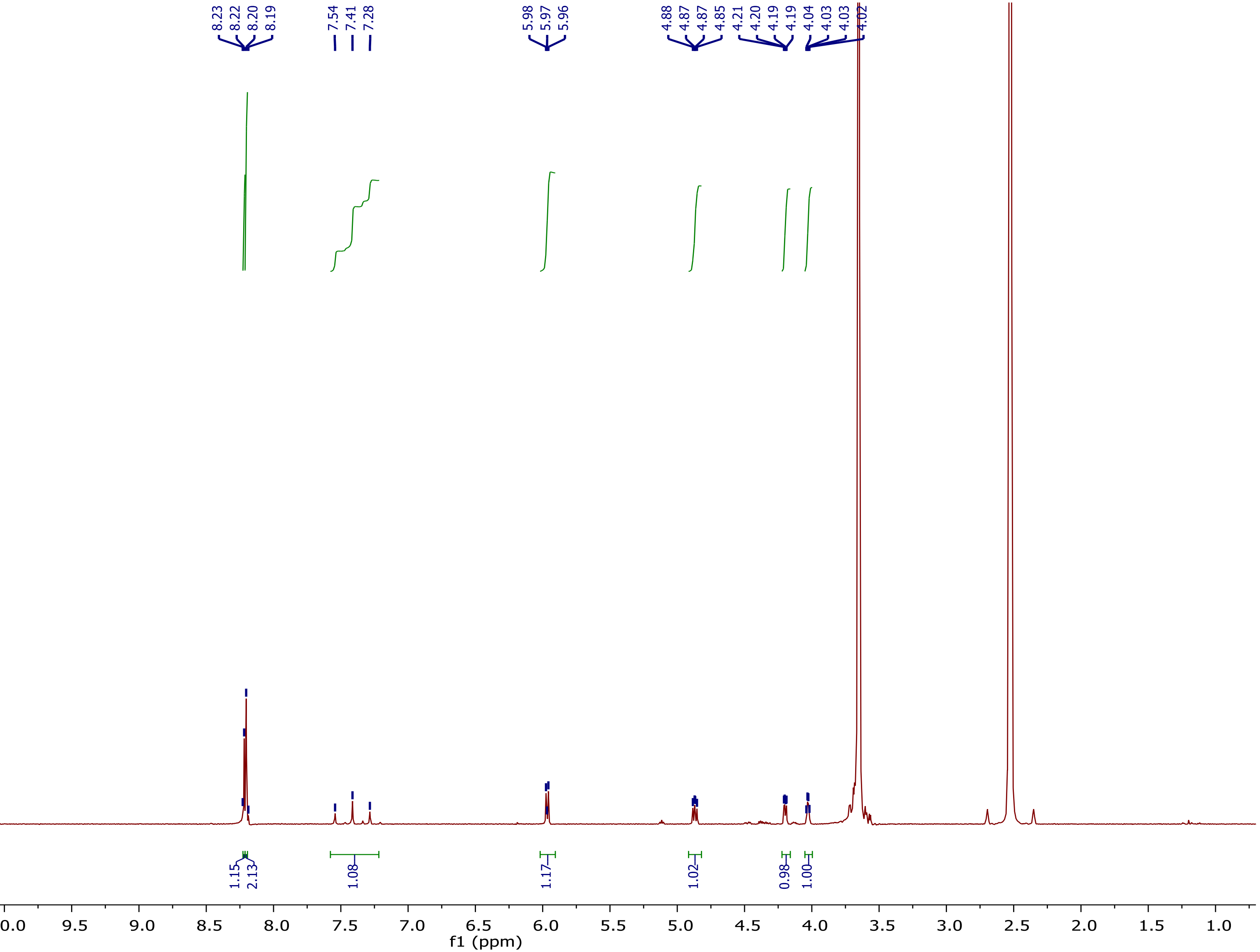
8.23
8.22
8.20
8.19
7.54
7.41
7.28
5.98
5.97
5.96
4.88
4.87
4.87
4.85
4.21
4.20
4.19
4.19
4.04
4.03
4.03
4.02
1.15
2.13
1.08
1.17
1.02
0.98
1.00
12.0 11.5 11.0 10.5 10.0 9.5 9.0 8.5 8.0 7.5 7.0 6.5 6.0 5.5 5.0 4.5 4.0 3.5 3.0 2.5 2.0 1.5 1.0
f1 (ppm)

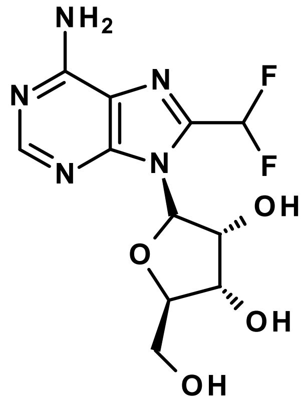
NH2
N
N
F
N
N
F
OH
O
OH
OH

28

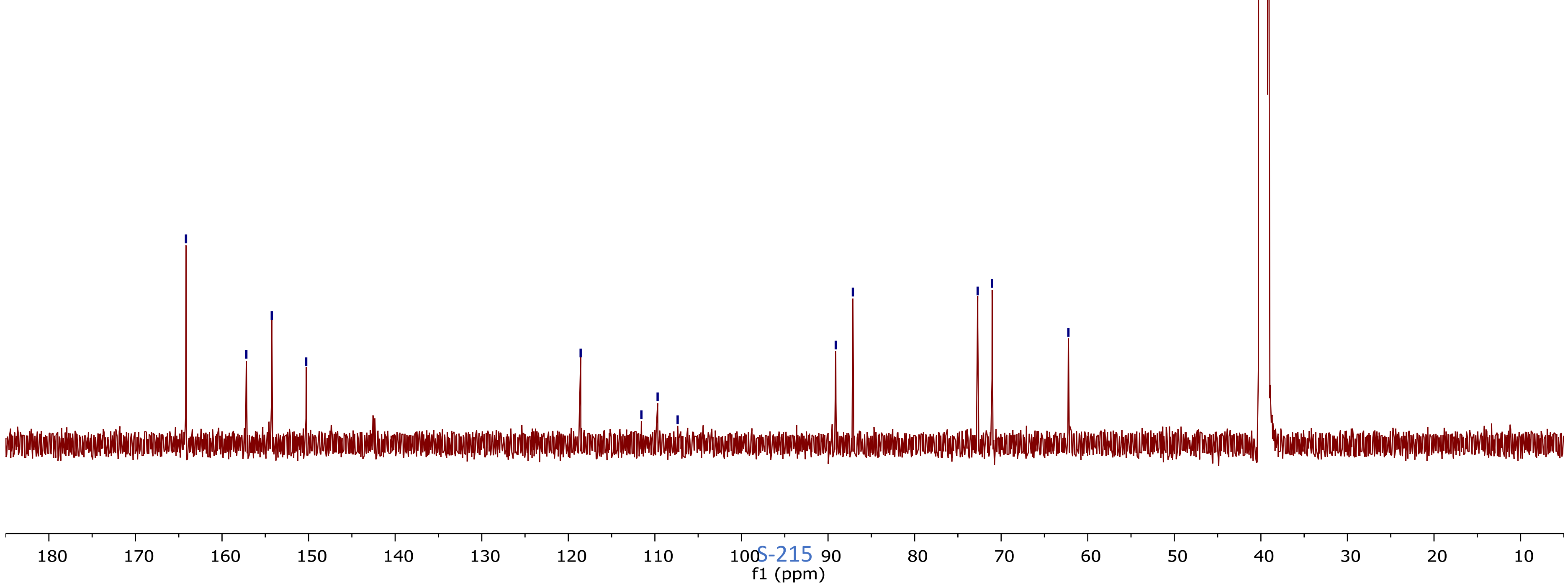
164.16
157.19
154.25
150.28
118.57
111.56
109.68
107.37
89.11
87.13
72.71
71.05
62.23
180
170
160
150
140
130
120
110
100
90
80
70
60
50
40
30
20
10
f1 (ppm)

-114.62
-114.75
-115.46
-115.60
-118.07
-118.21
-118.91
-119.05

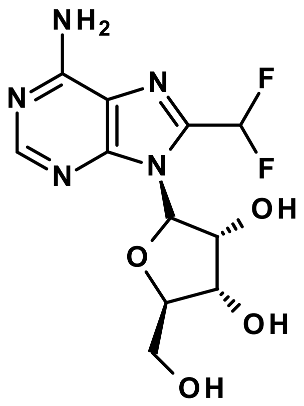

28

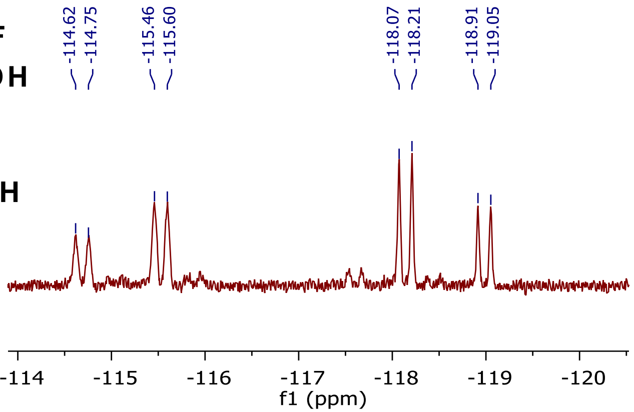

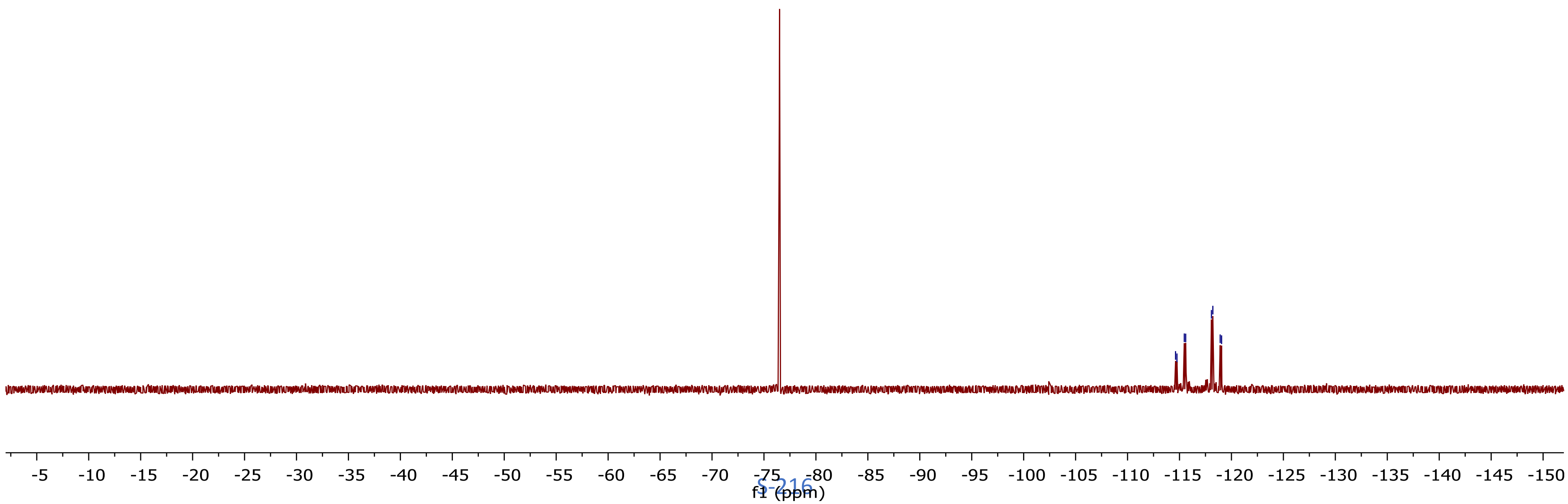

gg. ^{1}H, ^{13}C and ^{19}F NMR of 29

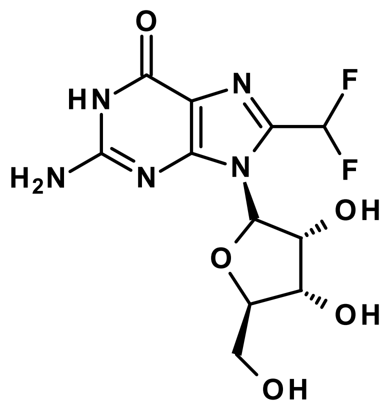
O
HN
N
F
F
H2N
N
N
O
OH
OH
OH

29

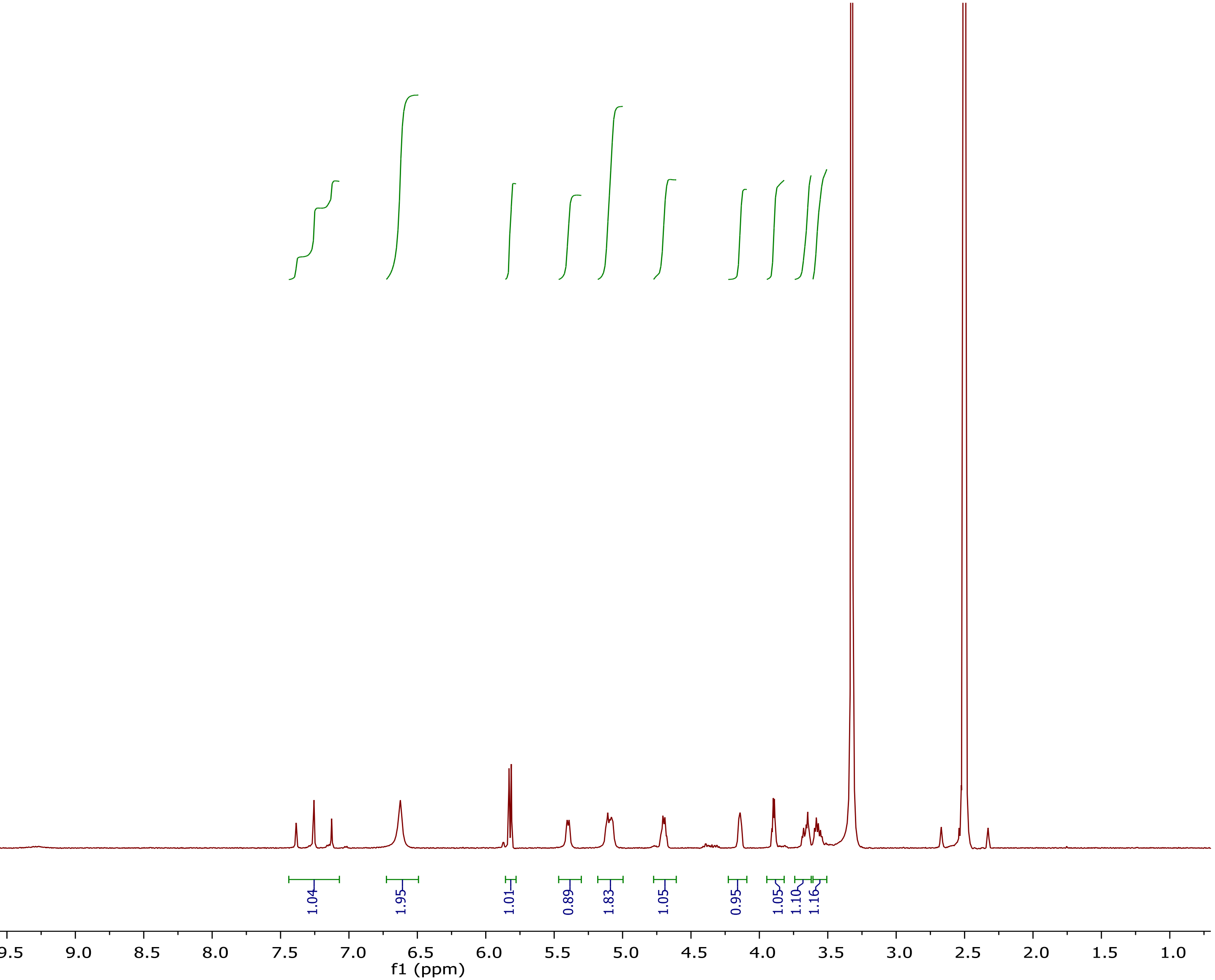
1.04
1.95
1.01
0.89
1.83
1.05
0.95
1.05
1.10
1.16
12.0 11.5 11.0 10.5 10.0 9.5 9.0 8.5 8.0 7.5 7.0 6.5 6.0 5.5 5.0 4.5 4.0 3.5 3.0 2.5 2.0 1.5 1.0
f1 (ppm)

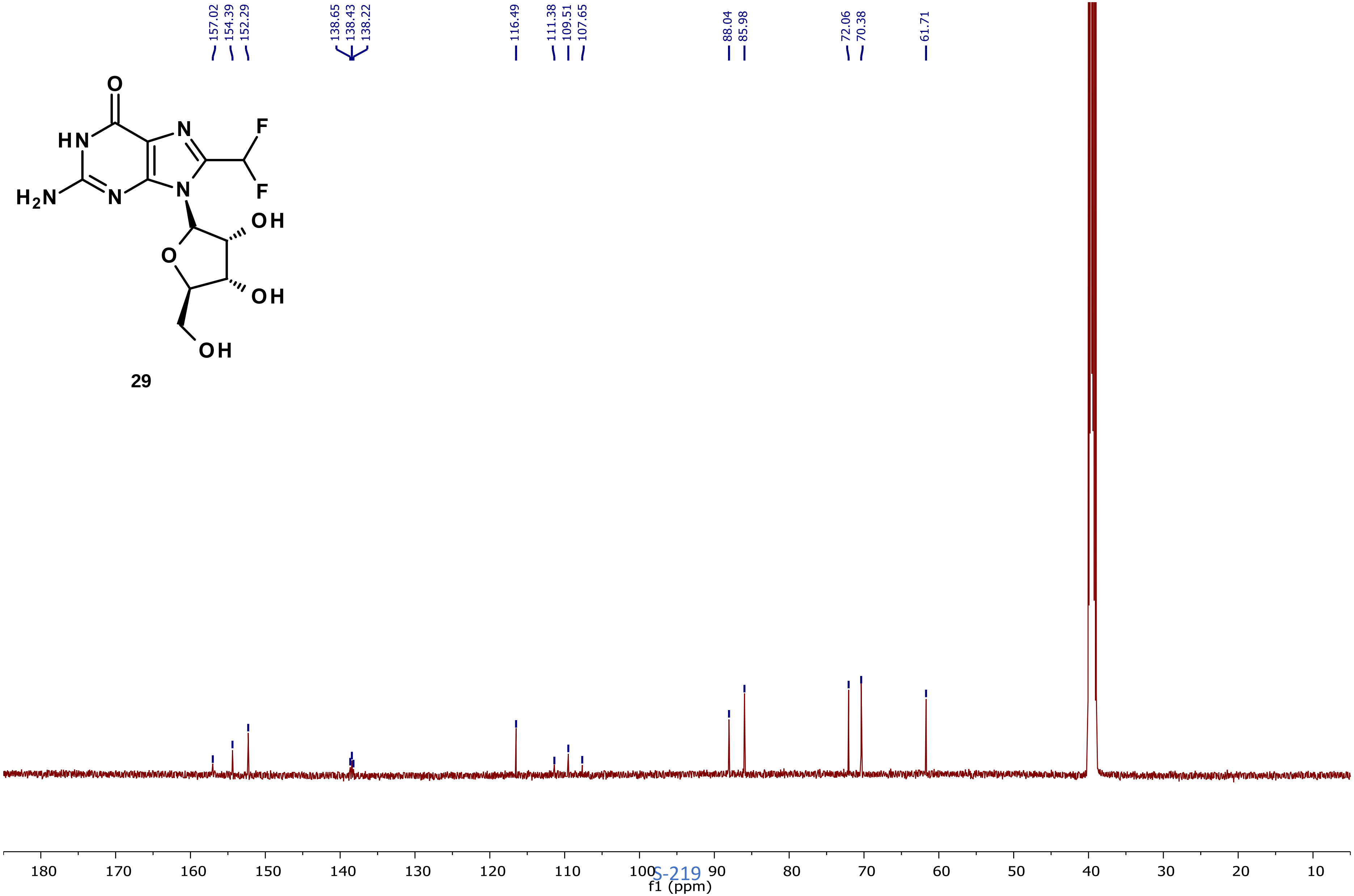
157.02
154.39
152.29
138.65
138.43
138.22
116.49
111.38
109.51
107.65
88.04
85.98
72.06
70.38
61.71
180
170
160
150
140
130
120
110
100
90
80
70
60
50
40
30
20
10
f1 (ppm)

29

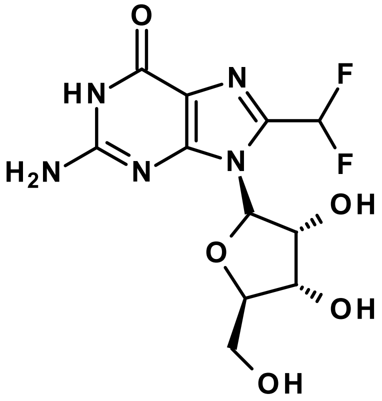

29

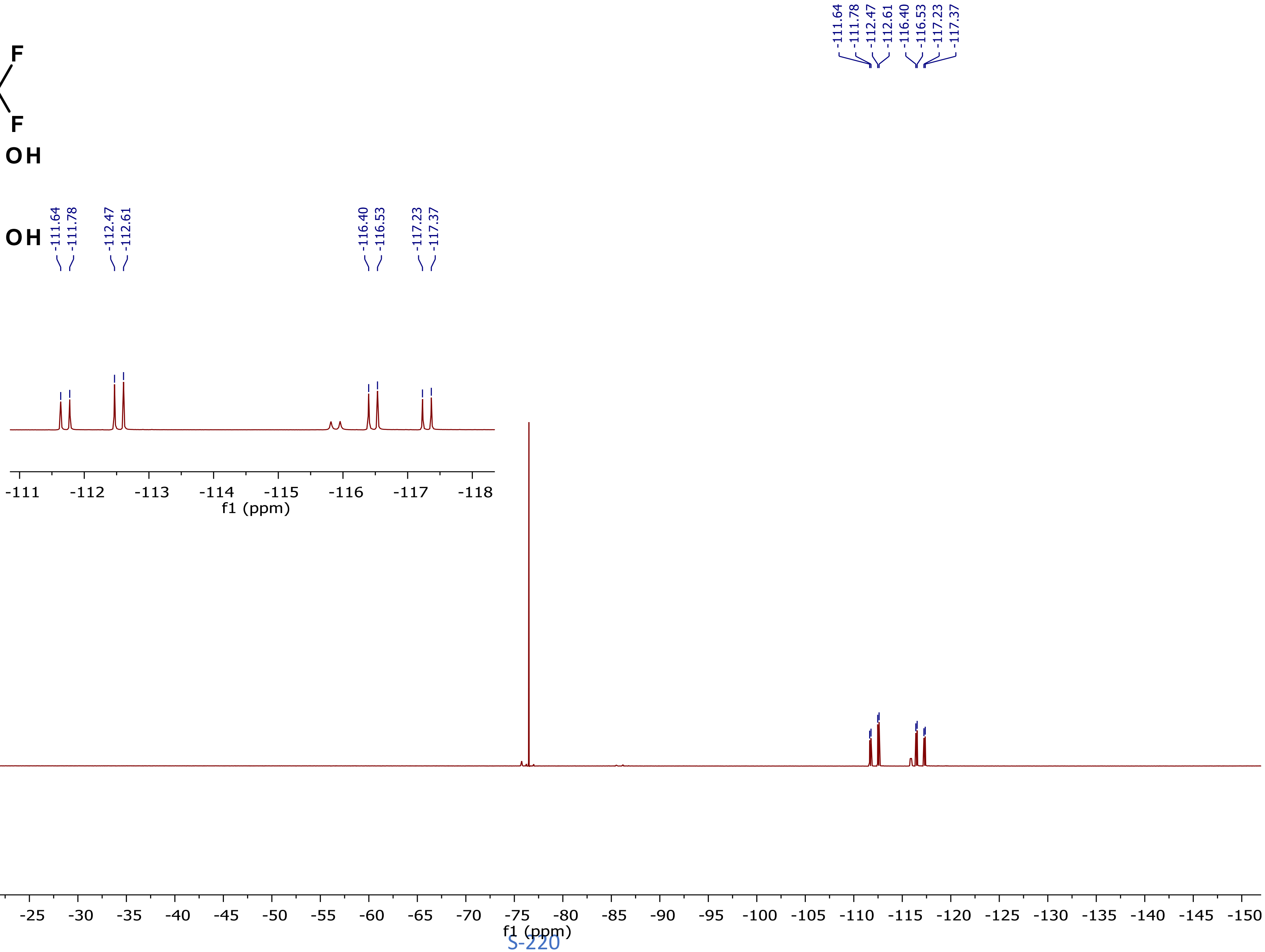
-111.64
-111.78
-112.47
-112.61
-116.40
-116.53
-117.23
-117.37
-111 -112 -113 -114 -115 -116 -117 -118
f1 (ppm)
-5 -10 -15 -20 -25 -30 -35 -40 -45 -50 -55 -60 -65 -70 -75 -80 -85 -90 -95 -100 -105 -110 -115 -120 -125 -130 -135 -140 -145 -150
f1 (ppm)

hh. ^{1}H, ^{13}C and ^{19}F NMR of 30

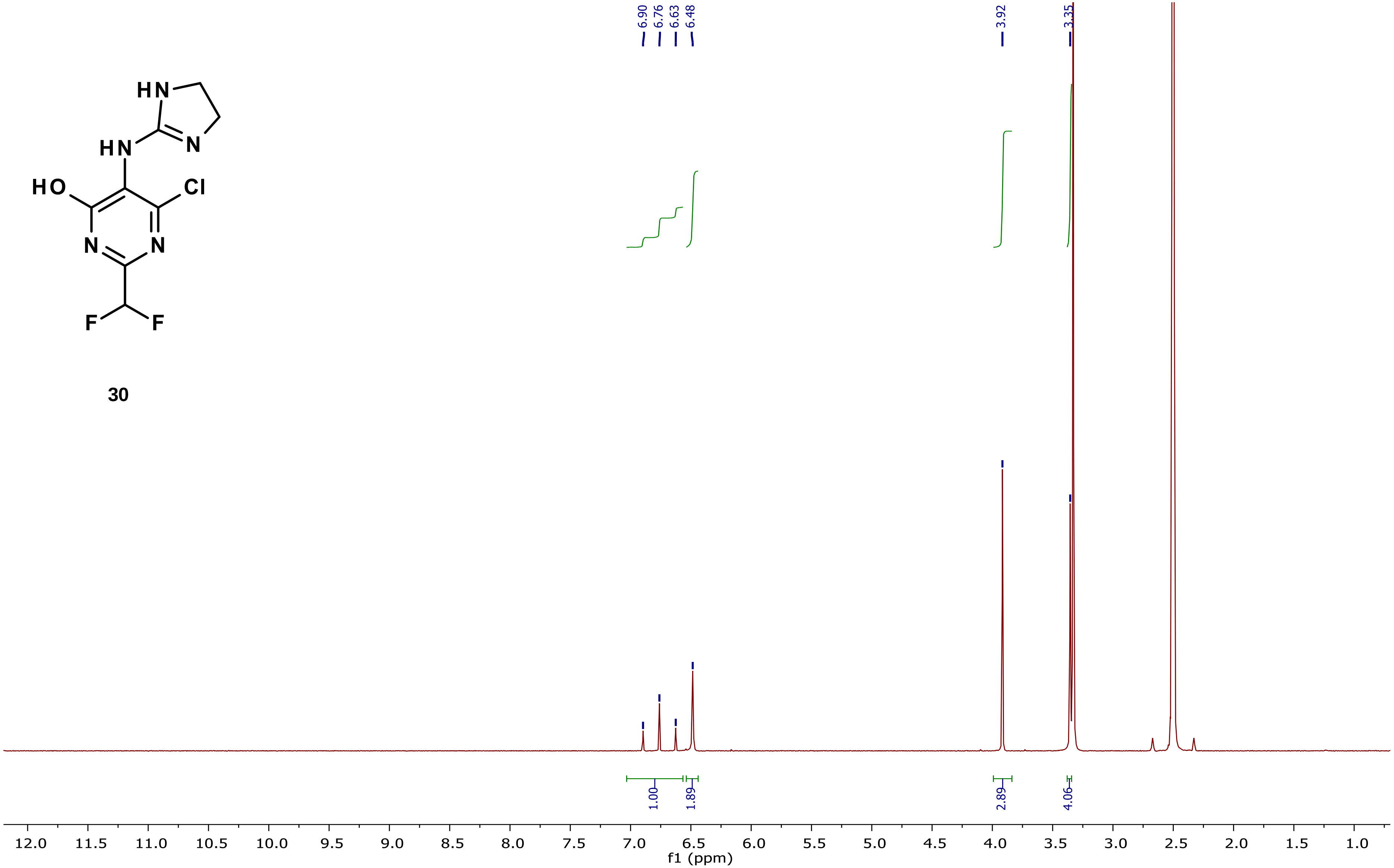
HN
HN
N
HO
Cl
N
N
F
F
30
6.90
6.76
6.63
6.48
3.92
3.35
1.00
1.89
2.89
4.06
12.0
11.5
11.0
10.5
10.0
9.5
9.0
8.5
8.0
7.5
7.0
6.5
6.0
5.5
5.0
4.5
4.0
3.5
3.0
2.5
2.0
1.5
1.0
f1 (ppm)

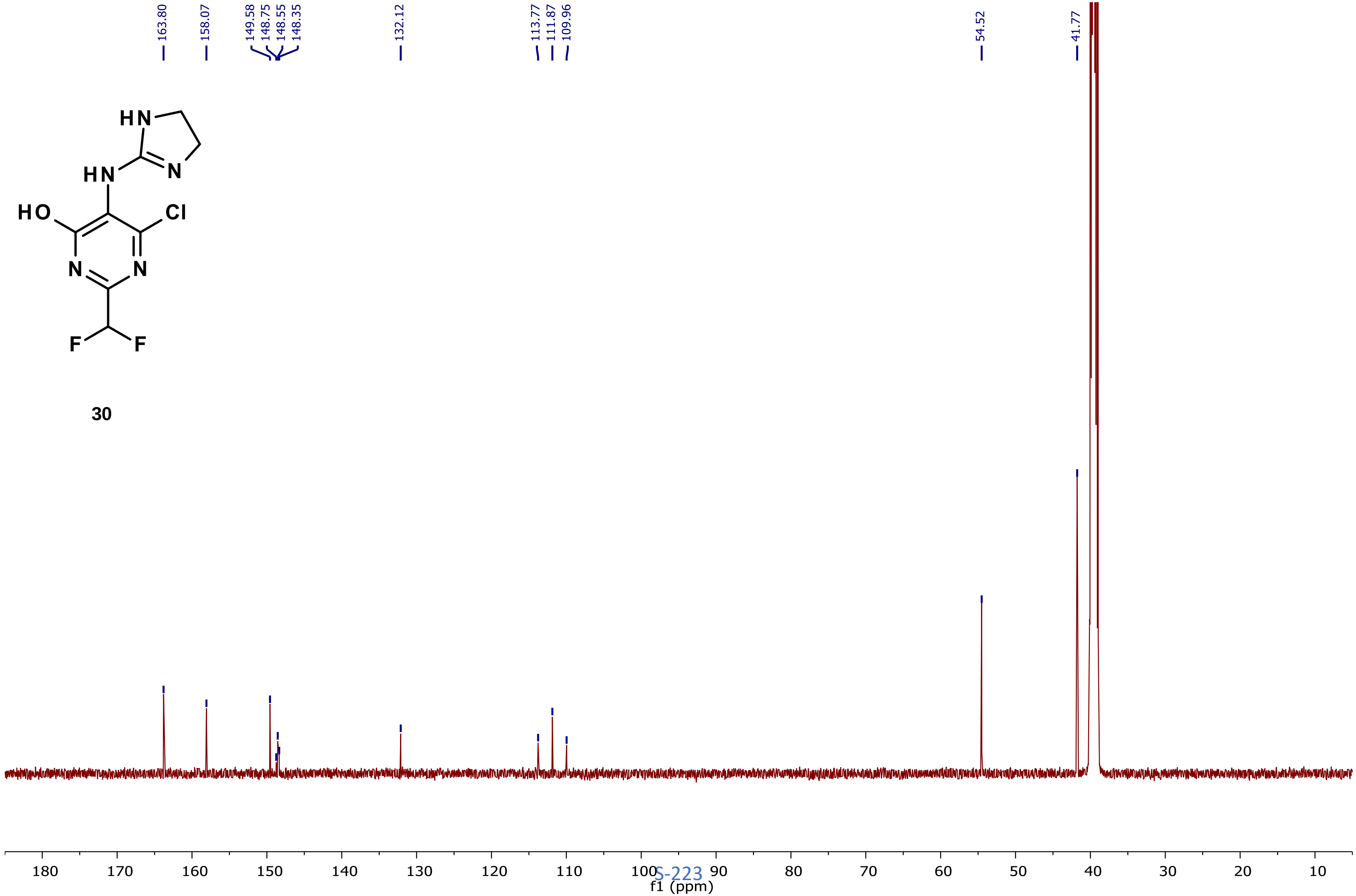
163.80
158.07
149.58
148.75
148.55
148.35
132.12
113.77
111.87
109.96
54.52
41.77
180 170 160 150 140 130 120 110 100 90 80 70 60 50 40 30 20 10
f1 (ppm)

30

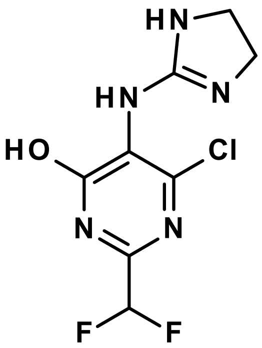

30

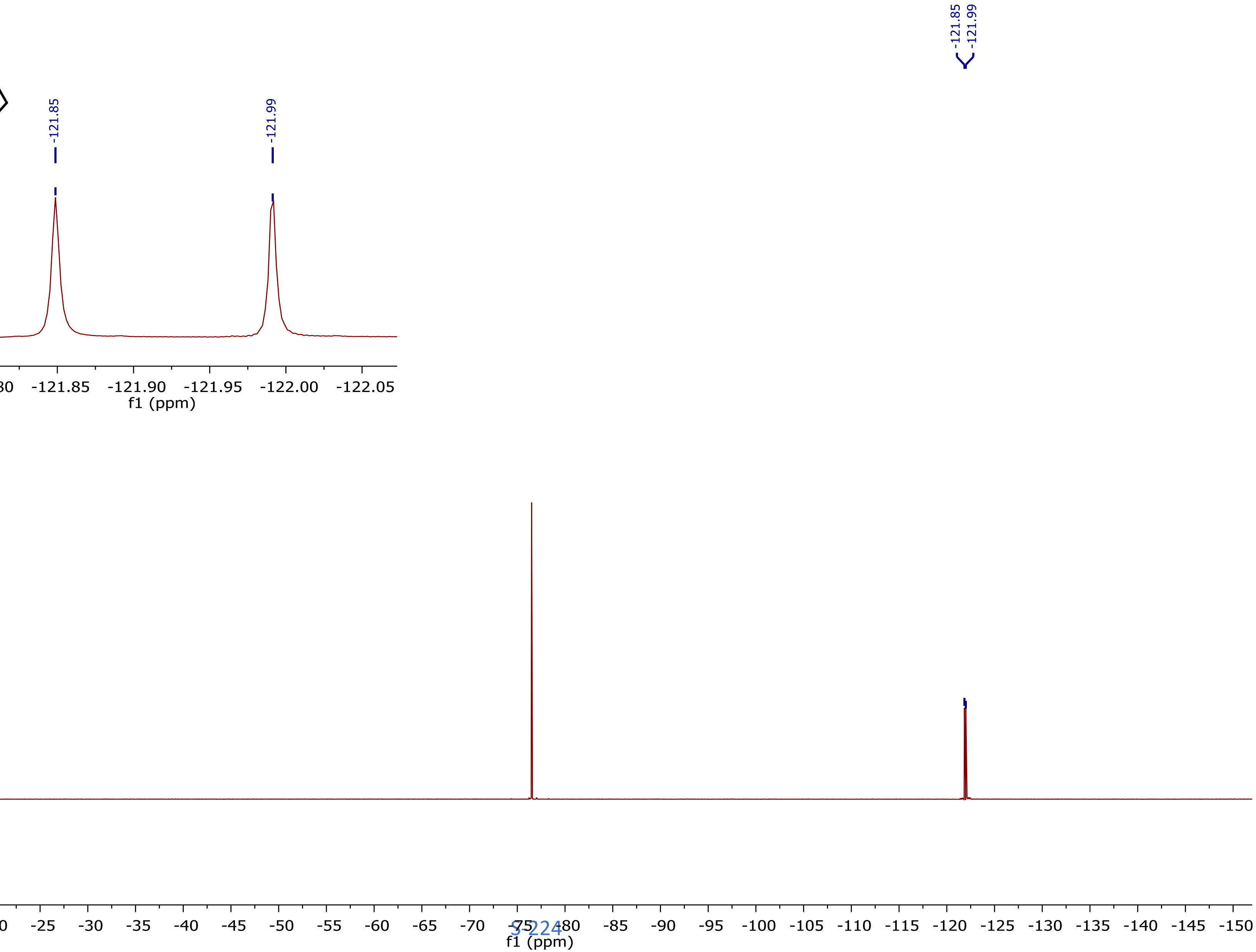
-121.85
-121.99
-121.80
-121.85
-121.90
-121.95
-122.00
-122.05
f1 (ppm)
-5
-10
-15
-20
-25
-30
-35
-40
-45
-50
-55
-60
-65
-70
-75
-80
-85
-90
-95
-100
-105
-110
-115
-120
-125
-130
-135
-140
-145
-150
f1 (ppm)

ii. ^{1}H, ^{13}C and ^{19}F NMR of 31a

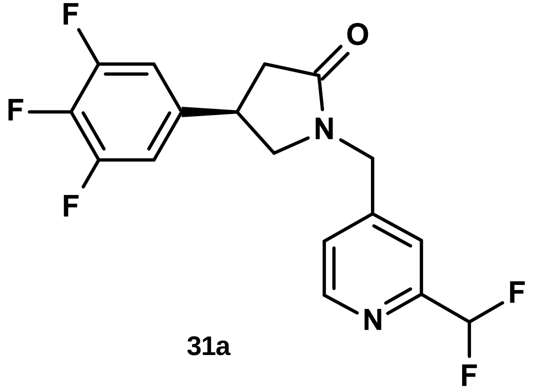

31a

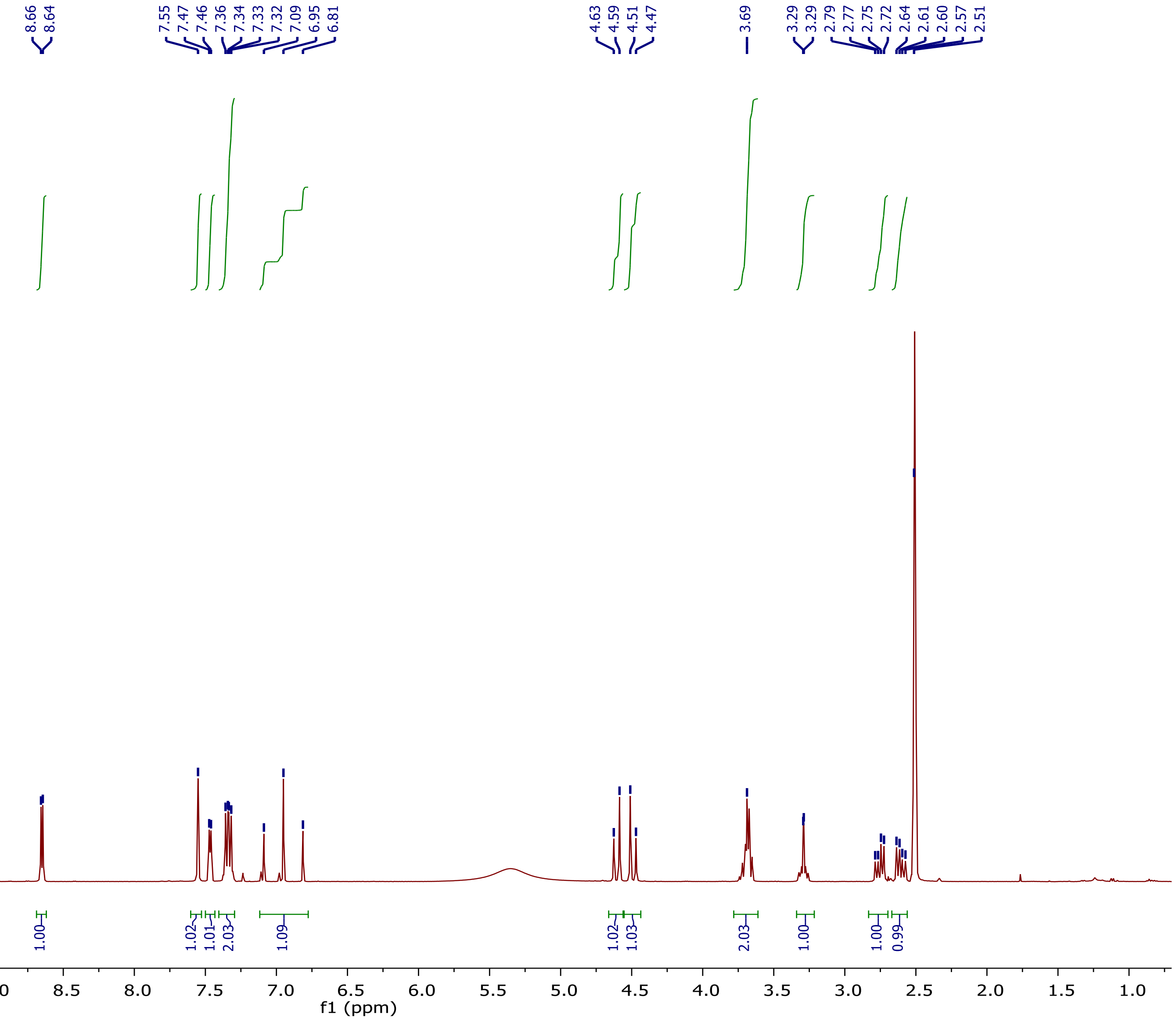
8.66
8.64
7.55
7.47
7.46
7.36
7.34
7.33
7.32
7.09
6.95
6.81
4.63
4.59
4.51
4.47
3.69
3.29
3.29
2.79
2.77
2.75
2.72
2.64
2.61
2.60
2.57
2.51
1.00
1.02
1.01
2.03
1.09
1.02
1.03
2.03
1.00
1.00
0.99
12.0
11.5
11.0
10.5
10.0
9.5
9.0
8.5
8.0
7.5
7.0
6.5
6.0
5.5
5.0
4.5
4.0
3.5
3.0
2.5
2.0
1.5
1.0
f1 (ppm)

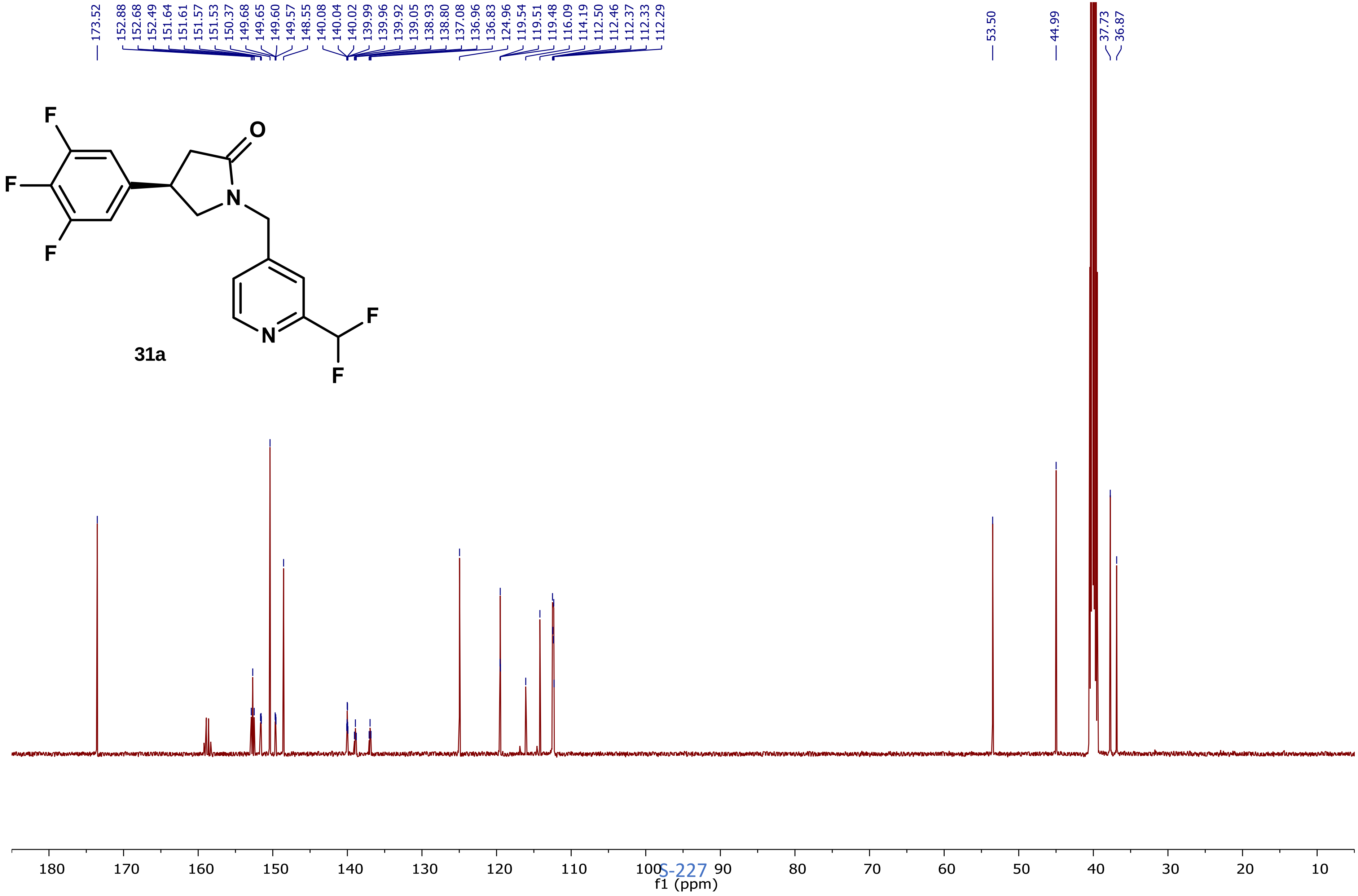
173.52
152.88
152.68
152.49
151.64
151.61
151.57
151.53
150.37
149.68
149.65
149.60
149.57
148.55
140.08
140.04
140.02
139.99
139.96
139.92
139.05
138.93
138.80
137.08
136.96
136.83
124.96
119.54
119.51
119.48
116.09
114.19
112.50
112.46
112.37
112.33
112.29
53.50
44.99
37.73
36.87
31a
f1 (ppm)

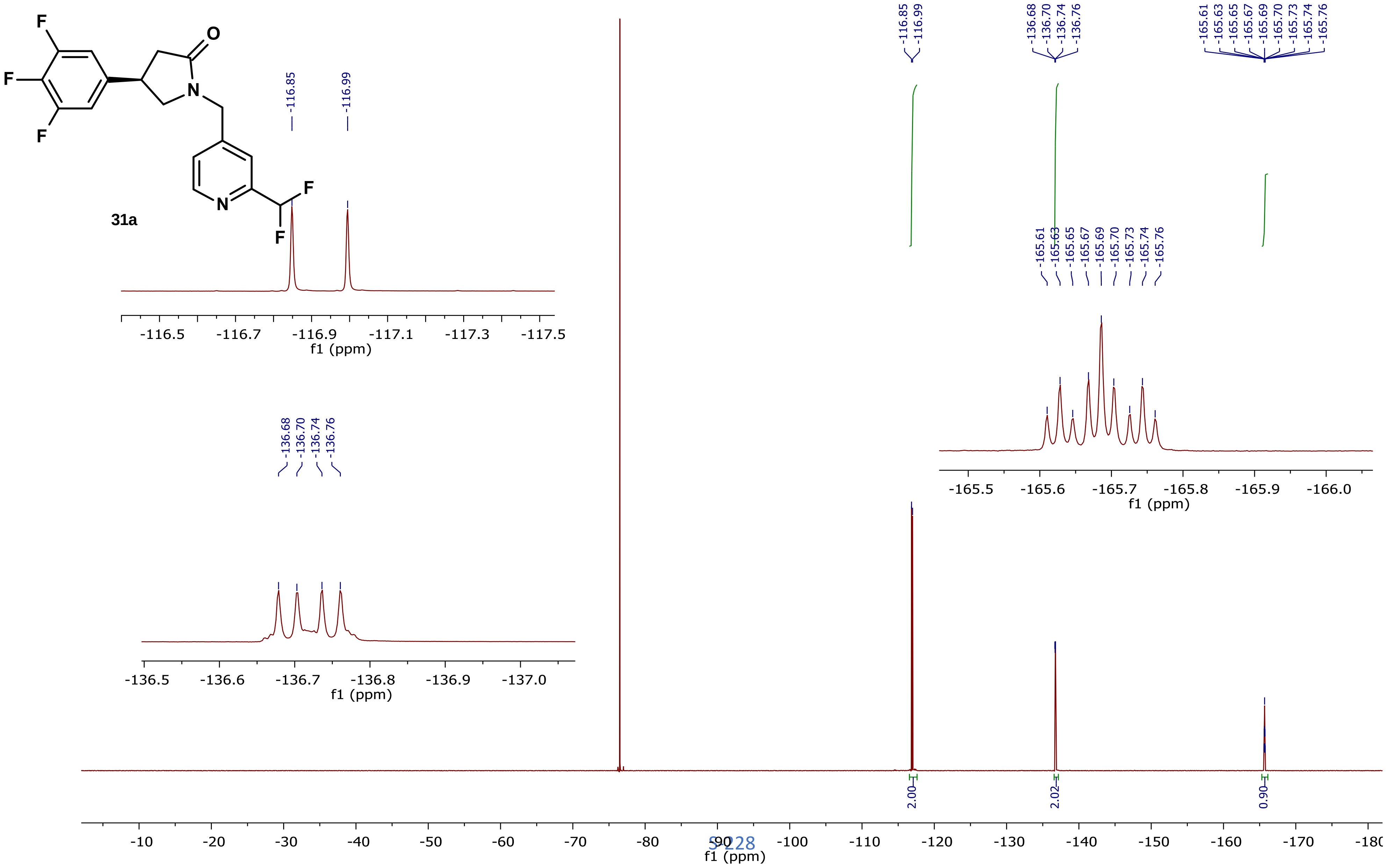

-116.85
-116.99
-136.68
-136.70
-136.74
-136.76
-165.61
-165.63
-165.65
-165.67
-165.69
-165.70
-165.73
-165.74
-165.76
-116.5 -116.7 -116.9 -117.1 -117.3 -117.5
f1 (ppm)
-136.5 -136.6 -136.7 -136.8 -136.9 -137.0
f1 (ppm)
-165.5 -165.6 -165.7 -165.8 -165.9 -166.0
f1 (ppm)
2.00
2.02
0.90
-10 -20 -30 -40 -50 -60 -70 -80 -90 -100 -110 -120 -130 -140 -150 -160 -170 -180
f1 (ppm)

31a

jj. ^{1}H, HSCQ, HMBC and ^{19}F NMR of 31b

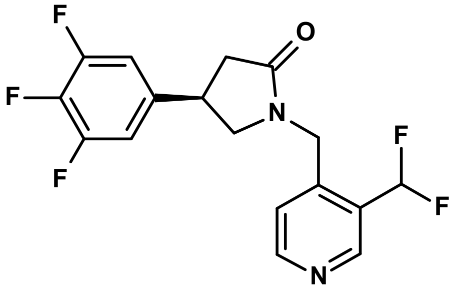

31b

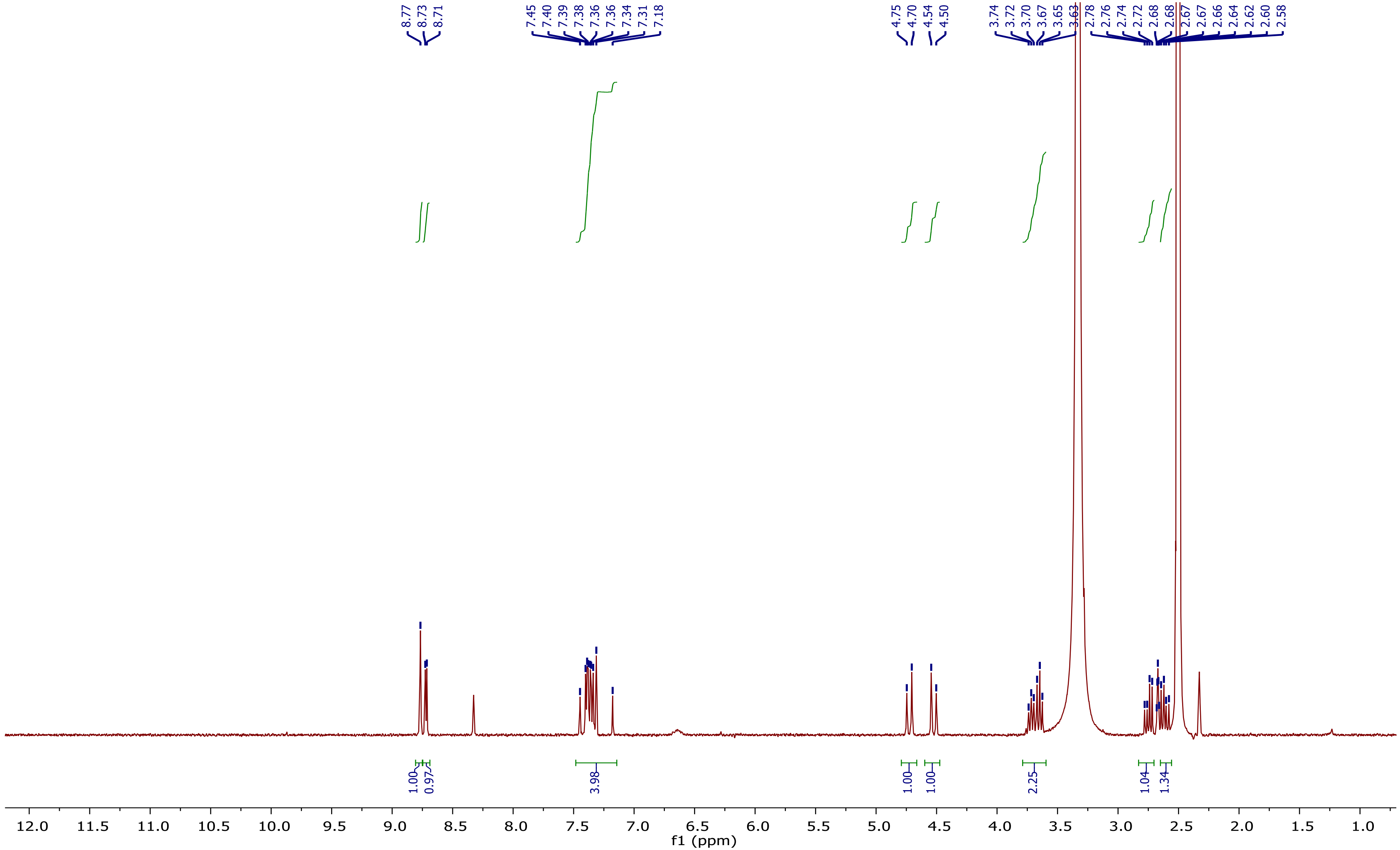
8.77
8.73
8.71
7.45
7.40
7.39
7.38
7.36
7.36
7.34
7.31
7.18
4.75
4.70
4.54
4.50
3.74
3.72
3.70
3.67
3.65
3.63
2.78
2.76
2.74
2.72
2.68
2.68
2.67
2.67
2.66
2.64
2.62
2.60
2.58
1.00
0.97
3.98
1.00
1.00
2.25
1.04
1.34
12.0 11.5 11.0 10.5 10.0 9.5 9.0 8.5 8.0 7.5 7.0 6.5 6.0 5.5 5.0 4.5 4.0 3.5 3.0 2.5 2.0 1.5 1.0
f1 (ppm)

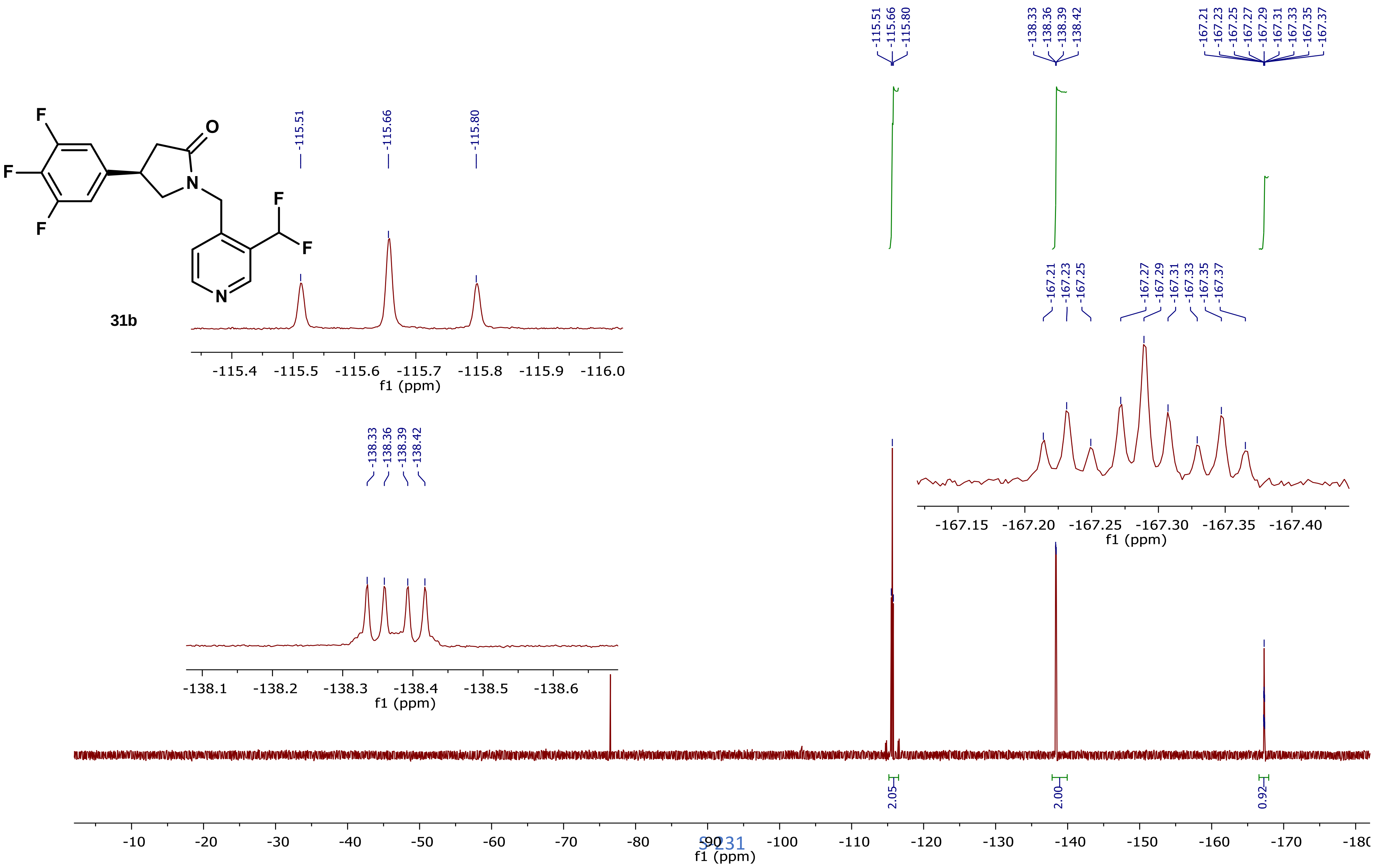
-115.51
-115.66
-115.80
-138.33
-138.36
-138.39
-138.42
-167.21
-167.23
-167.25
-167.27
-167.29
-167.31
-167.33
-167.35
-167.37
f1 (ppm)
2.05
2.00
0.92

31b

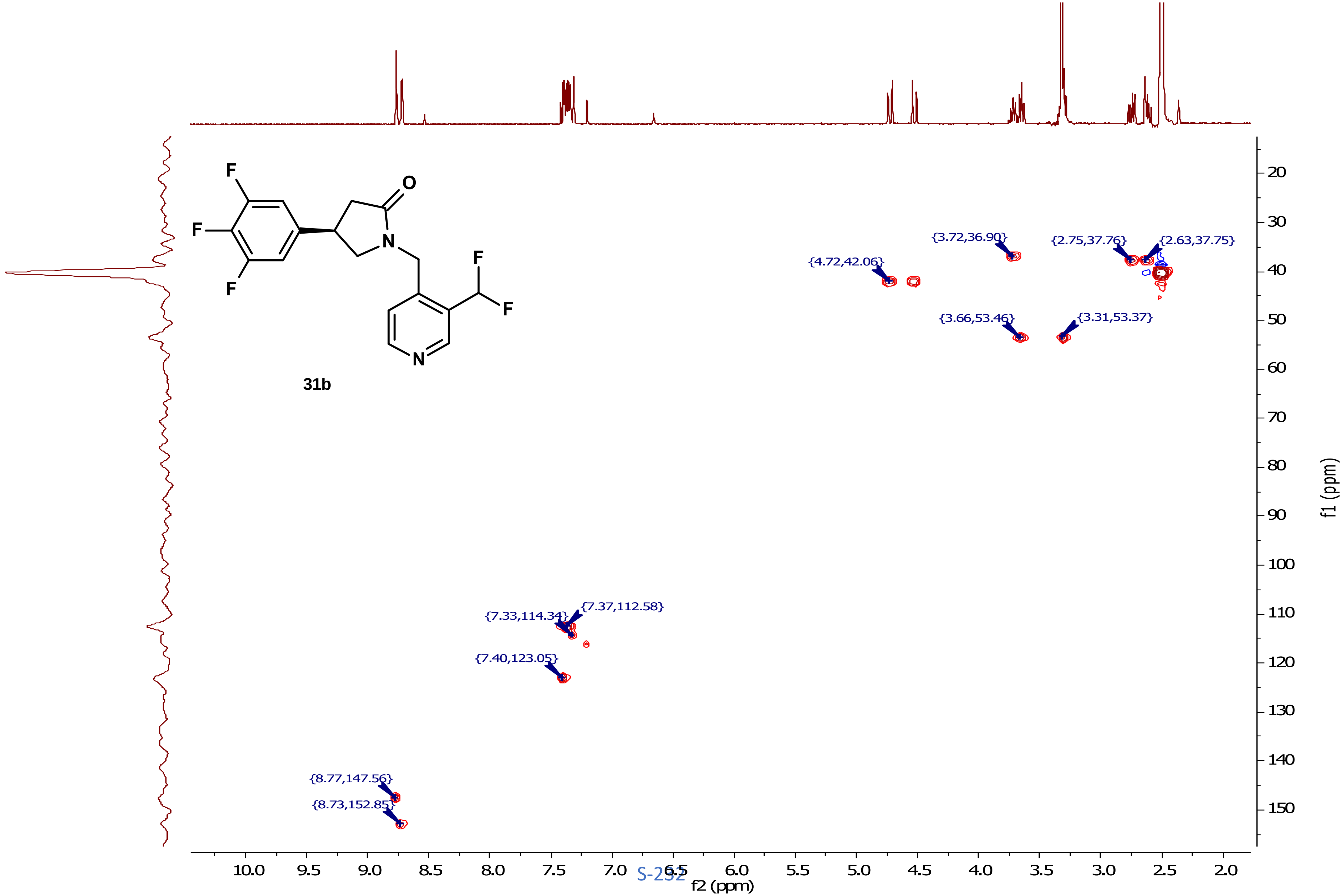
31b
{3.72,36.90}
{2.75,37.76}
{2.63,37.75}
{4.72,42.06}
{3.66,53.46}
{3.31,53.37}
{7.33,114.34}
{7.37,112.58}
{7.40,123.05}
{8.77,147.56}
{8.73,152.85}
f2 (ppm)
f1 (ppm)

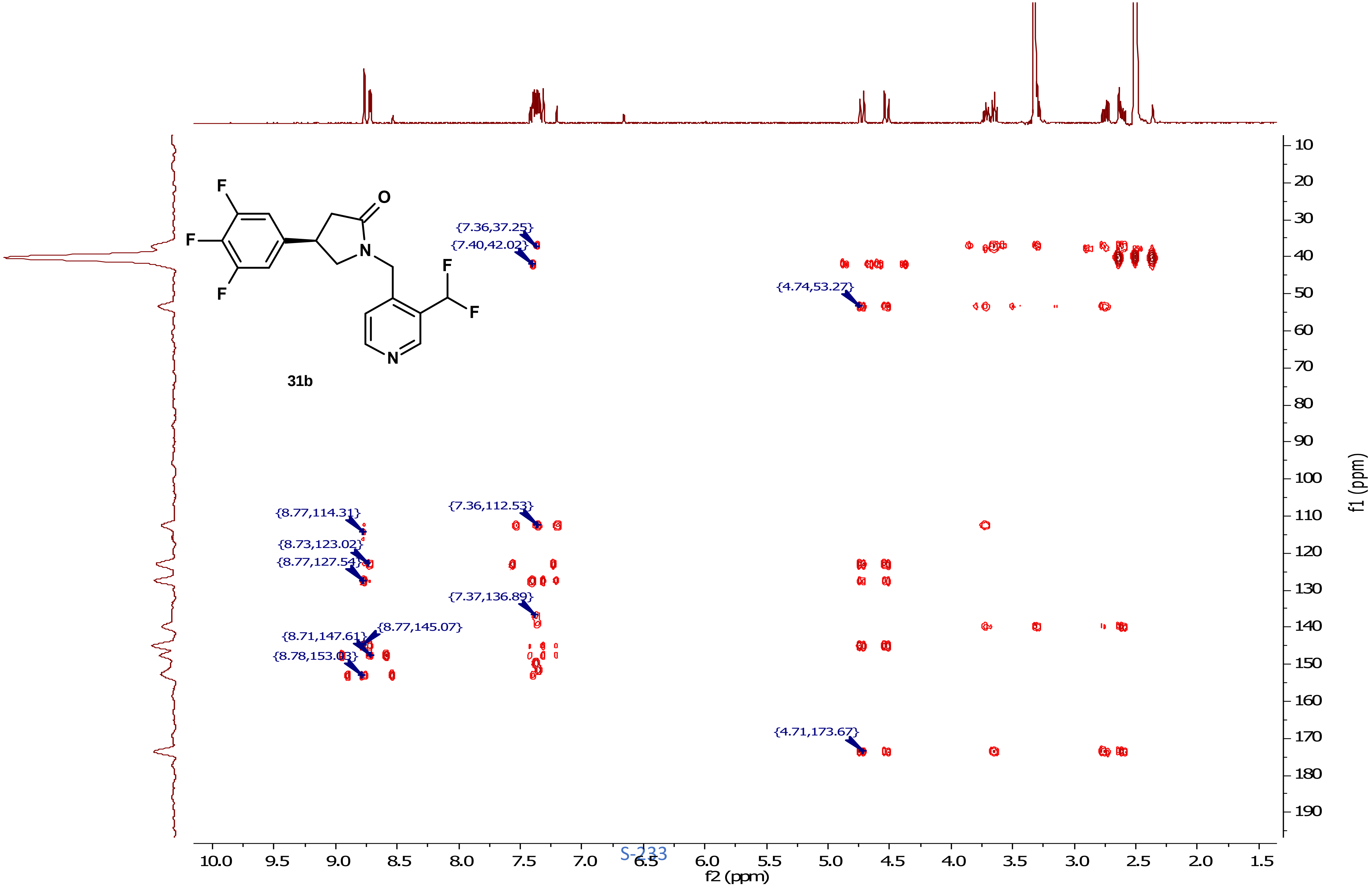
31b
{7.36,37.25}
{7.40,42.02}
{4.74,53.27}
{8.77,114.31}
{7.36,112.53}
{8.73,123.02}
{8.77,127.54}
{7.37,136.89}
{8.77,145.07}
{8.71,147.61}
{8.78,153.03}
{4.71,173.67}
f1 (ppm)
f2 (ppm)

kk. ^{1}H, HSCQ, HMBC and ^{19}F NMR of 31c

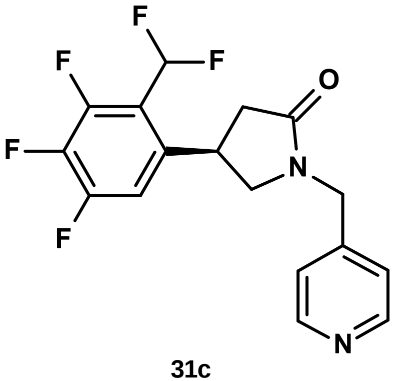

31c

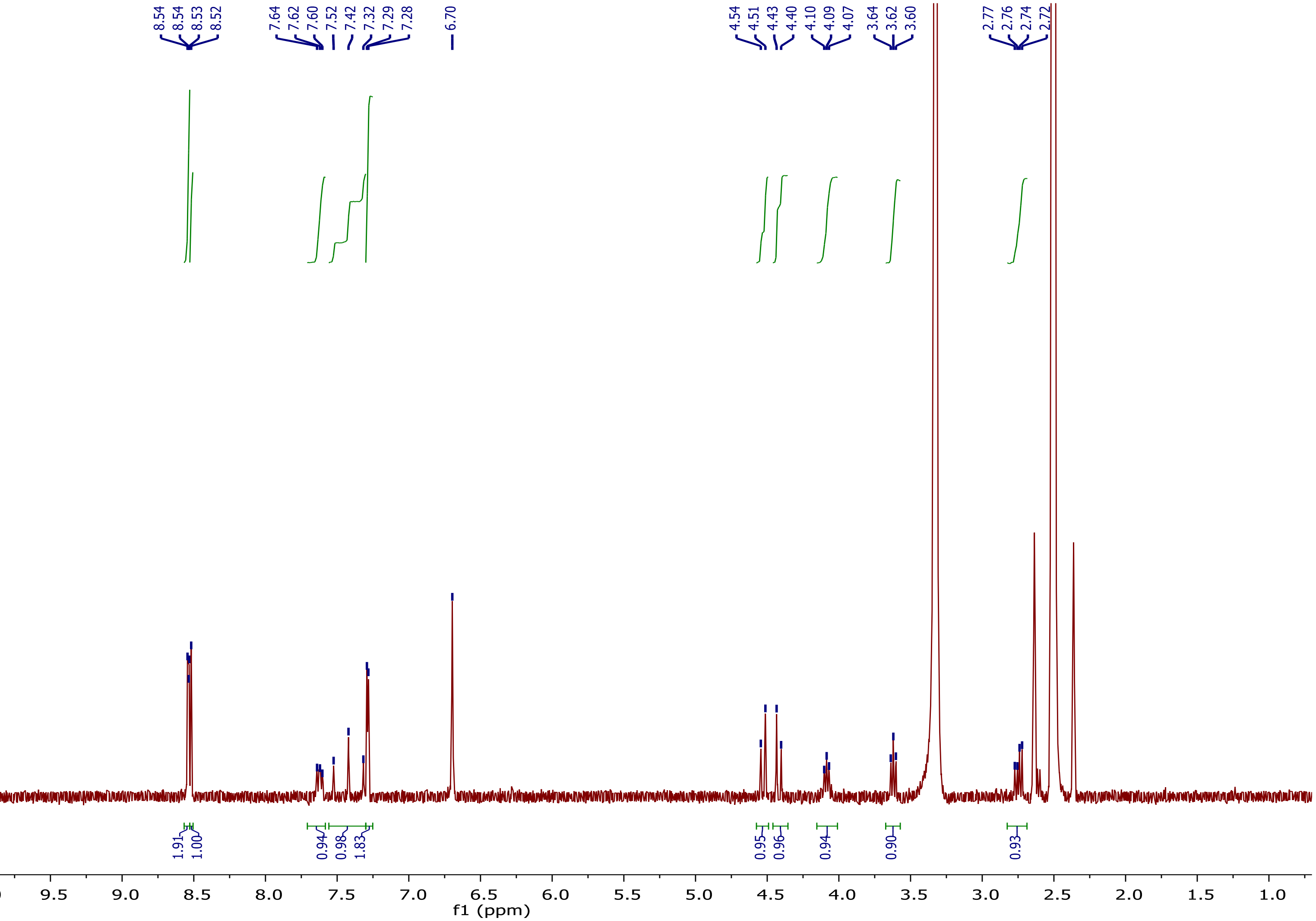

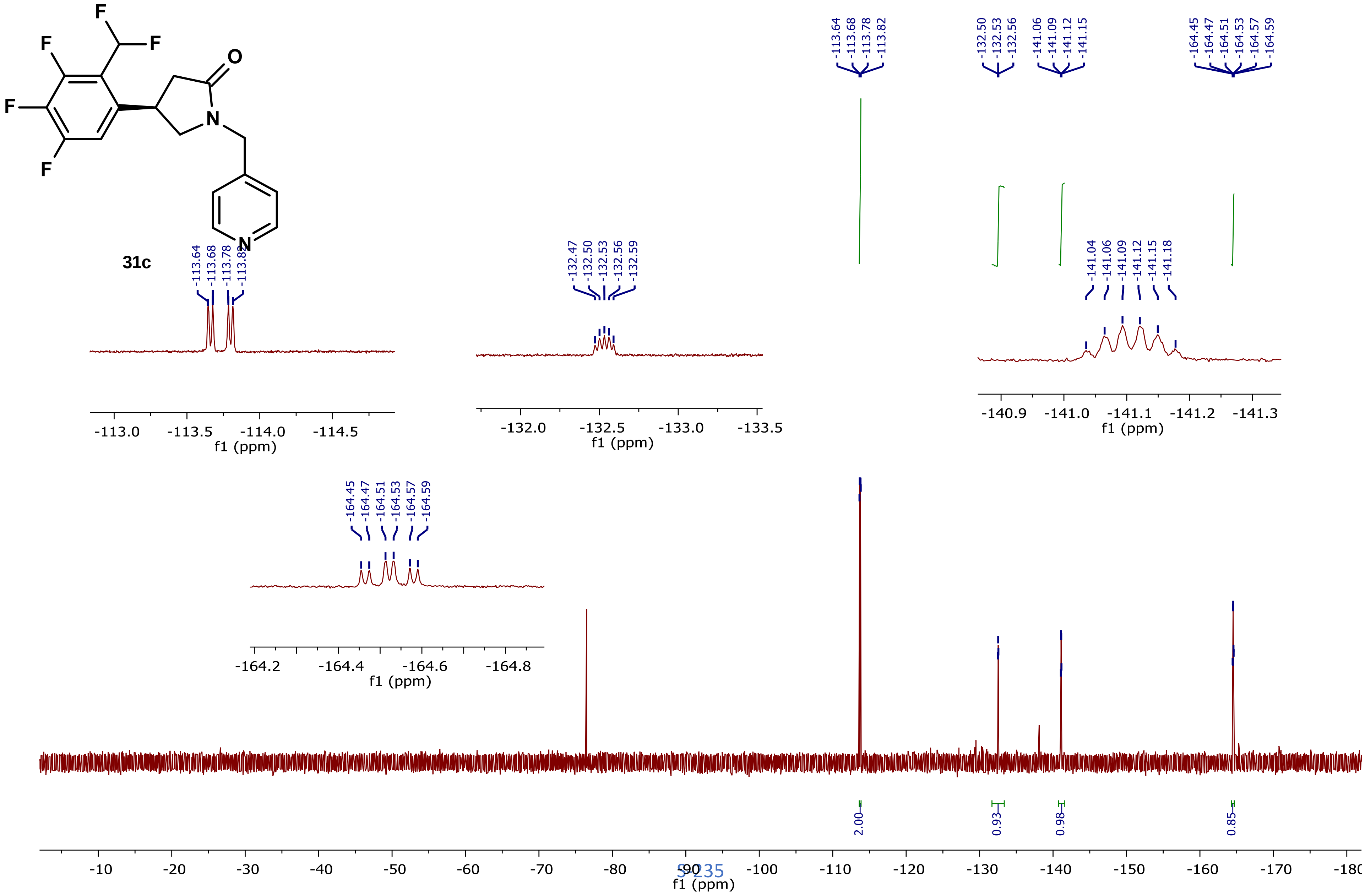
31c
-113.64
-113.68
-113.78
-113.82
-132.50
-132.53
-132.56
-141.06
-141.09
-141.12
-141.15
-164.45
-164.47
-164.51
-164.53
-164.57
-164.59
-132.47
-132.59
-141.04
-141.18
f1 (ppm)
2.00
0.93
0.98
0.85

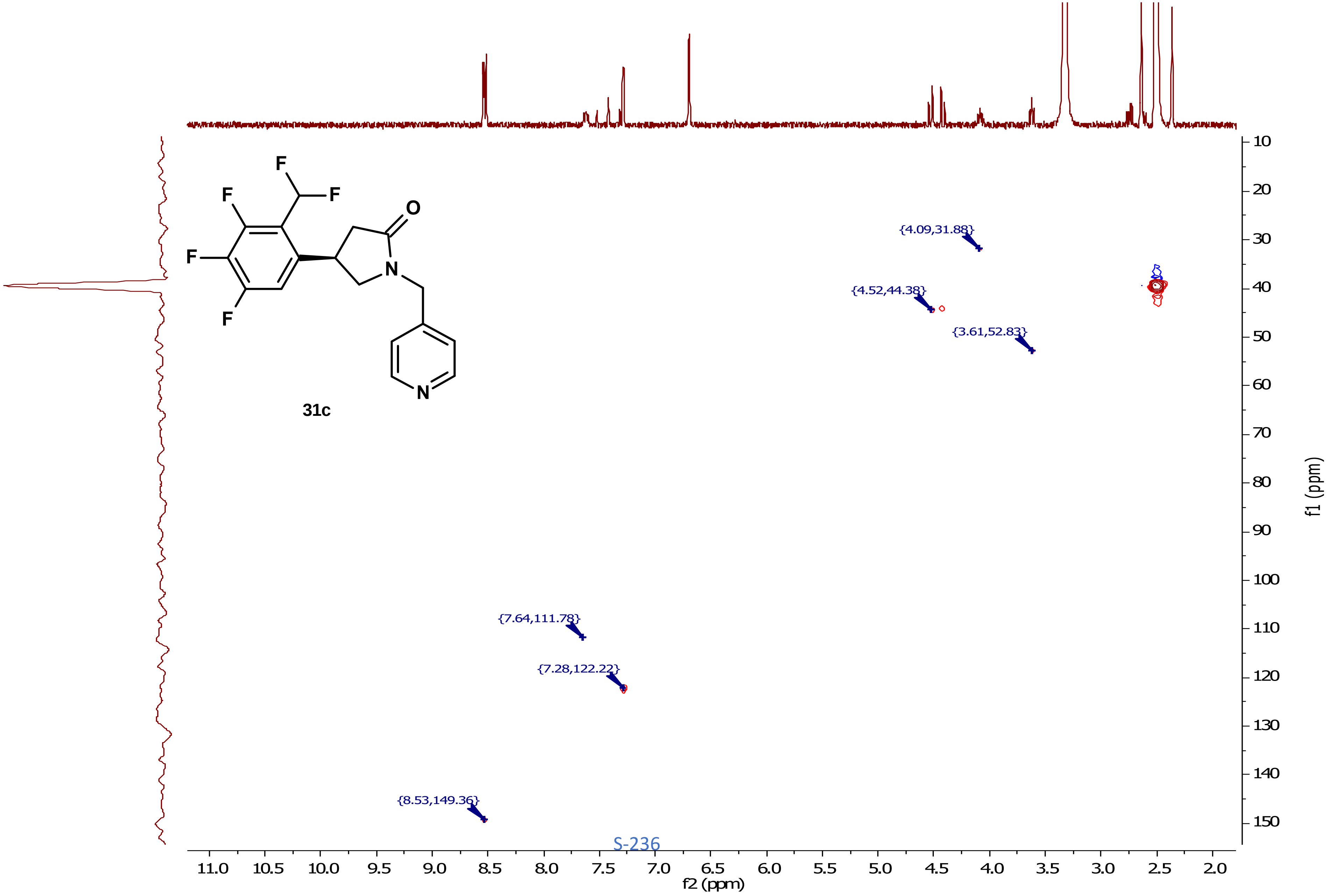
31c
{4.09,31.88}
{4.52,44.38}
{3.61,52.83}
{7.64,111.78}
{7.28,122.22}
{8.53,149.36}
f2 (ppm)
f1 (ppm)

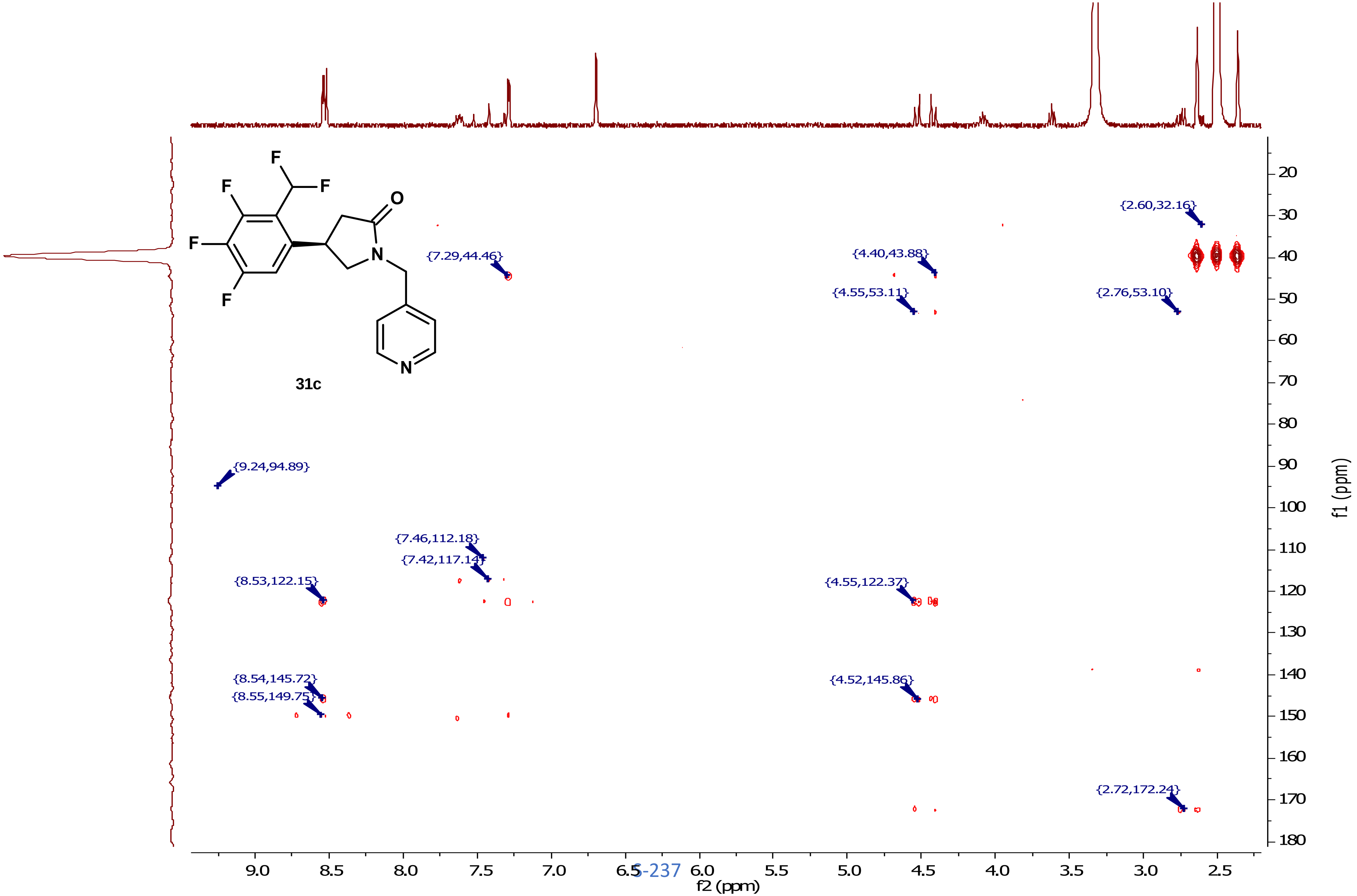
31c
{2.60,32.16}
{7.29,44.46}
{4.40,43.88}
{4.55,53.11}
{2.76,53.10}
{9.24,94.89}
{7.46,112.18}
{7.42,117.14}
{8.53,122.15}
{4.55,122.37}
{8.54,145.72}
{8.55,149.75}
{4.52,145.86}
{2.72,172.24}
f2 (ppm)
f1 (ppm)

II. ^{1}H and ^{13}C NMR of 35

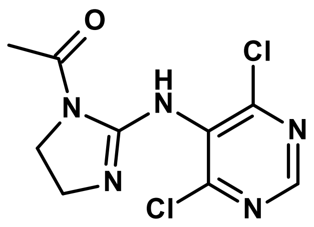

35

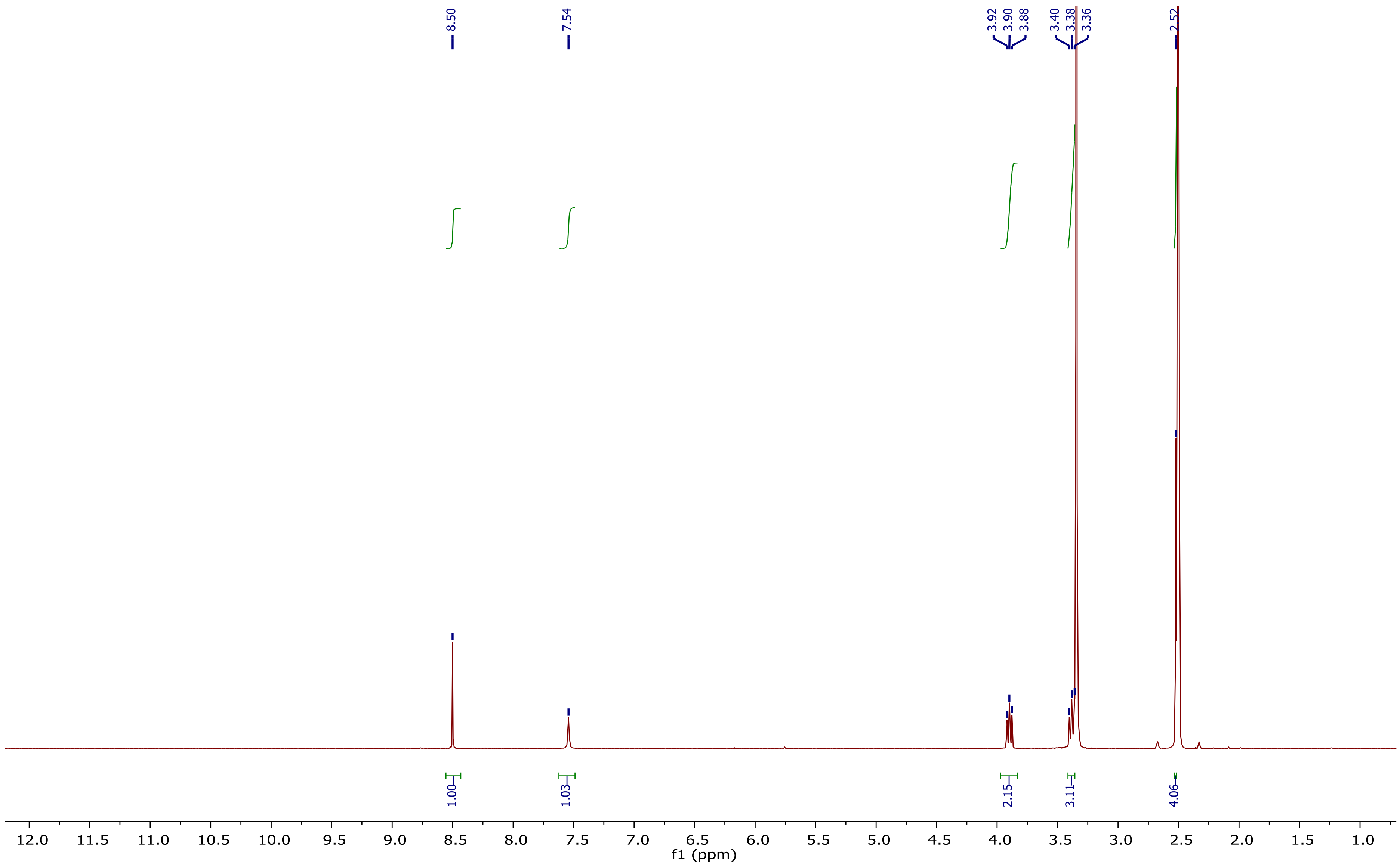
8.50
7.54
3.92
3.90
3.88
3.40
3.38
3.36
2.52
1.00
1.03
2.15
3.11
4.06
12.0 11.5 11.0 10.5 10.0 9.5 9.0 8.5 8.0 7.5 7.0 6.5 6.0 5.5 5.0 4.5 4.0 3.5 3.0 2.5 2.0 1.5 1.0
f1 (ppm)

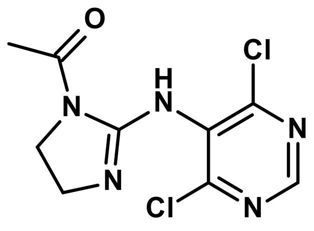
O
H
N
N
Cl
N
N
Cl
N

35

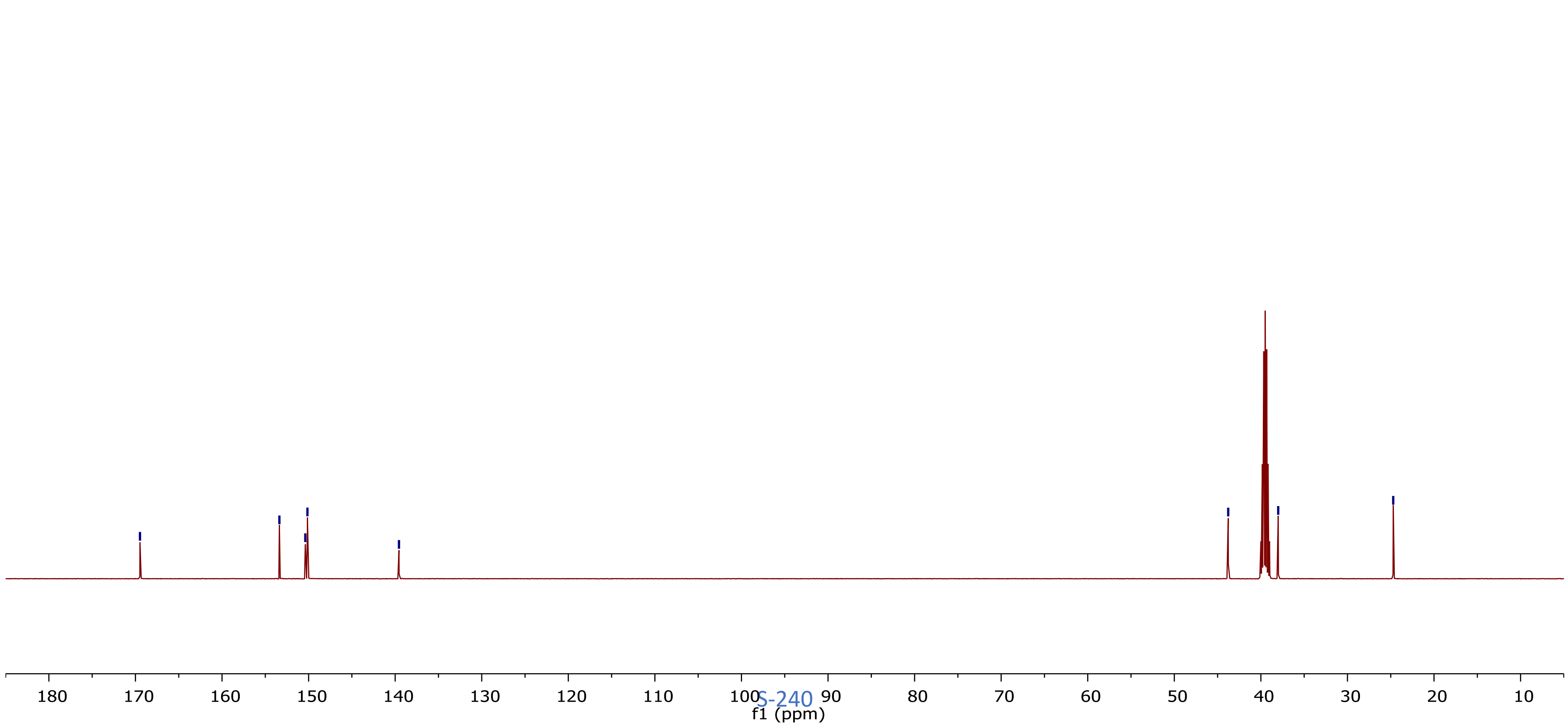
169.48
153.37
150.38
150.14
139.56
43.77
38.00
24.71
180 170 160 150 140 130 120 110 100 90 80 70 60 50 40 30 20 10
f1 (ppm)

mm. **^{1}H and ^{13}C NMR of 36**

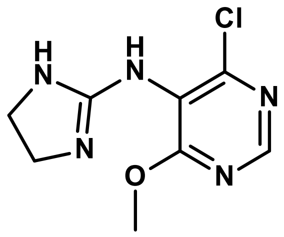

36

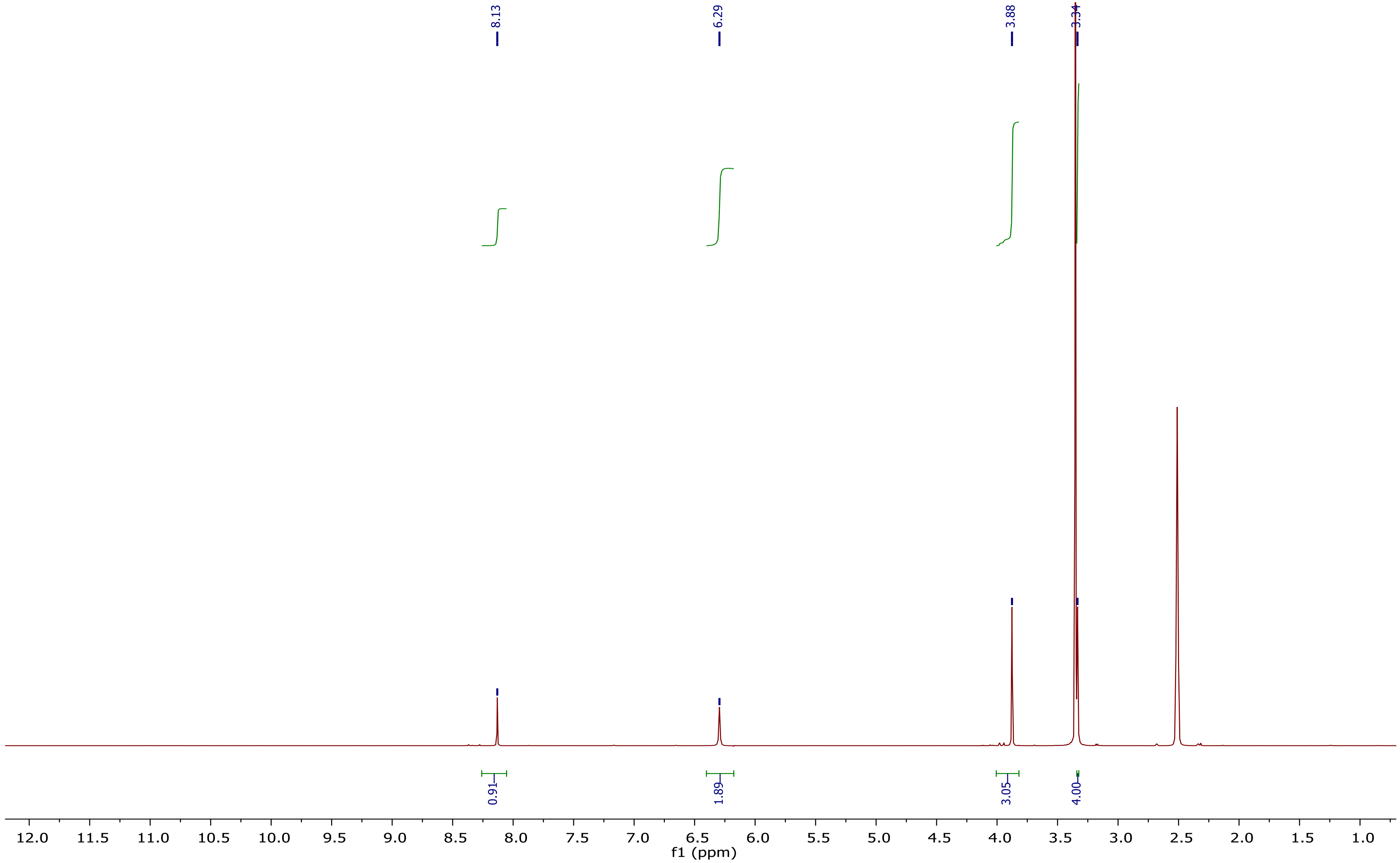
8.13
6.29
3.88
3.34
0.91
1.89
3.05
4.00
12.0 11.5 11.0 10.5 10.0 9.5 9.0 8.5 8.0 7.5 7.0 6.5 6.0 5.5 5.0 4.5 4.0 3.5 3.0 2.5 2.0 1.5 1.0
f1 (ppm)

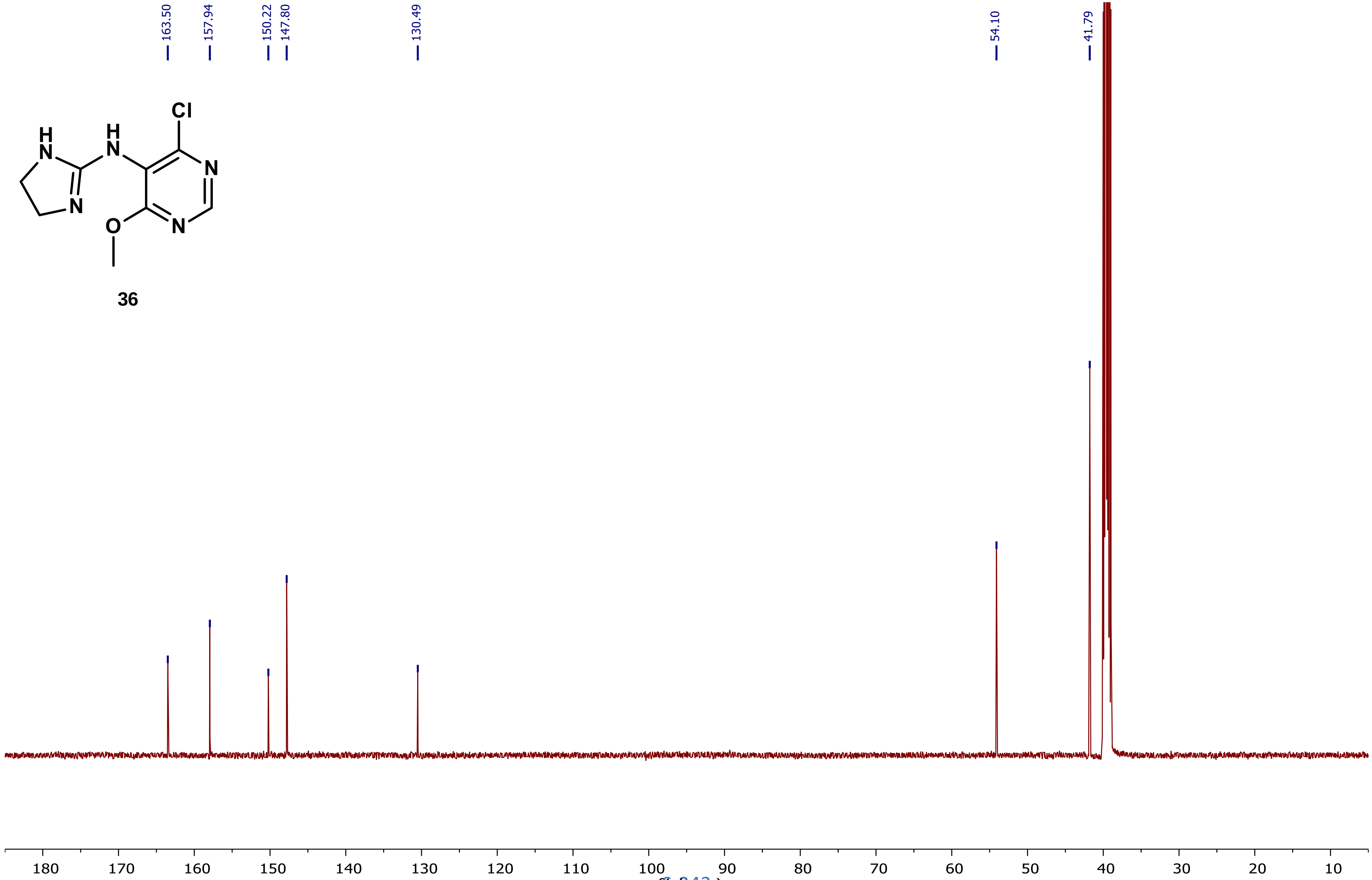
163.50
157.94
150.22
147.80
130.49
54.10
41.79
180 170 160 150 140 130 120 110 100 90 80 70 60 50 40 30 20 10
f1 (ppm)

36

nn.^{1}H, ^{13}C and ^{19}F NMR of 39

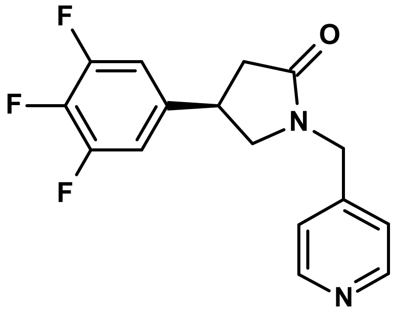
F
F
F
O
N
N

39

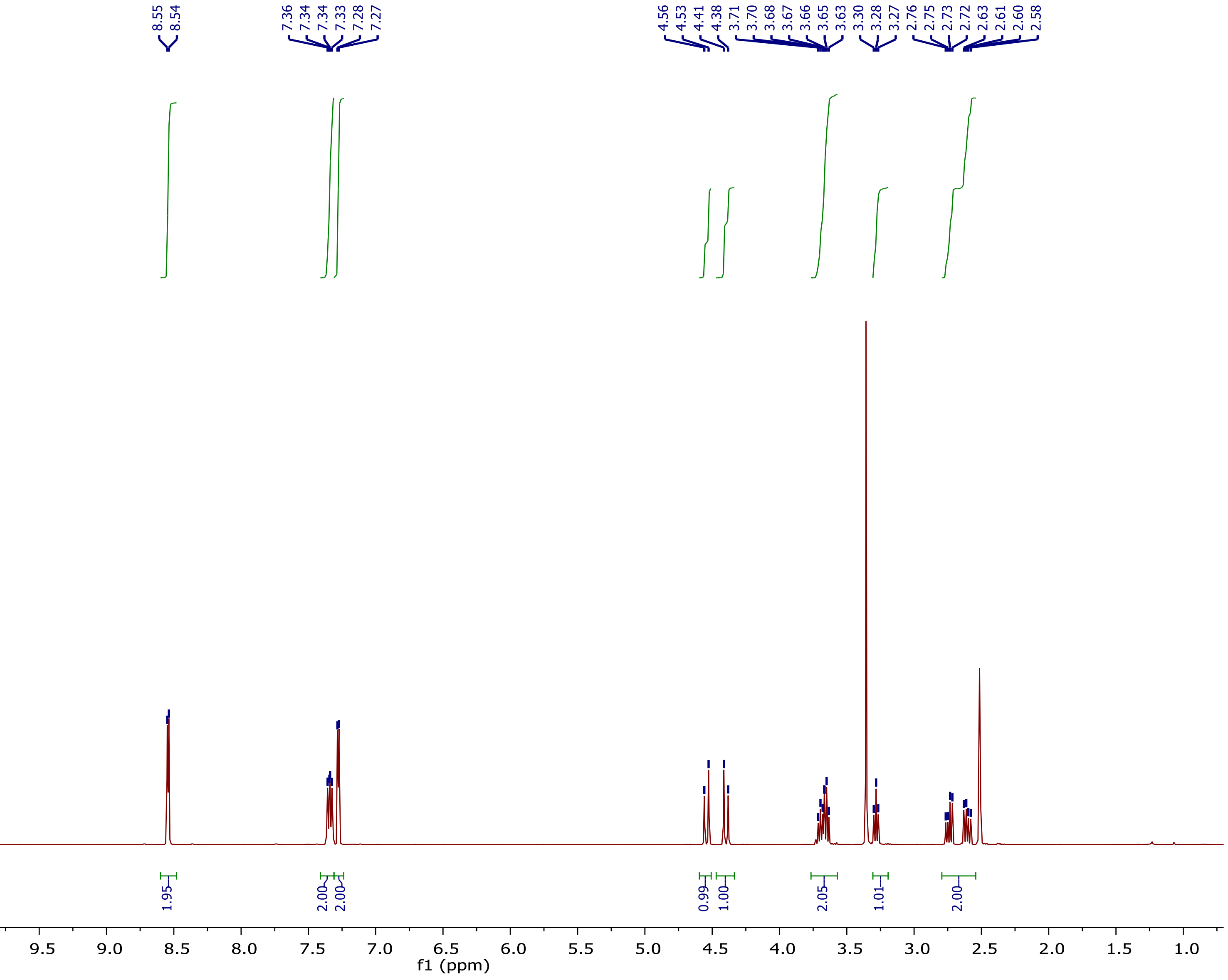
8.55
8.54
7.36
7.34
7.34
7.33
7.28
7.27
4.56
4.53
4.41
4.38
3.71
3.70
3.68
3.67
3.66
3.65
3.63
3.30
3.28
3.27
2.76
2.75
2.73
2.72
2.63
2.61
2.60
2.58
1.95
2.00
2.00
0.99
1.00
2.05
1.01
2.00
12.0 11.5 11.0 10.5 10.0 9.5 9.0 8.5 8.0 7.5 7.0 6.5 6.0 5.5 5.0 4.5 4.0 3.5 3.0 2.5 2.0 1.5 1.0
f1 (ppm)

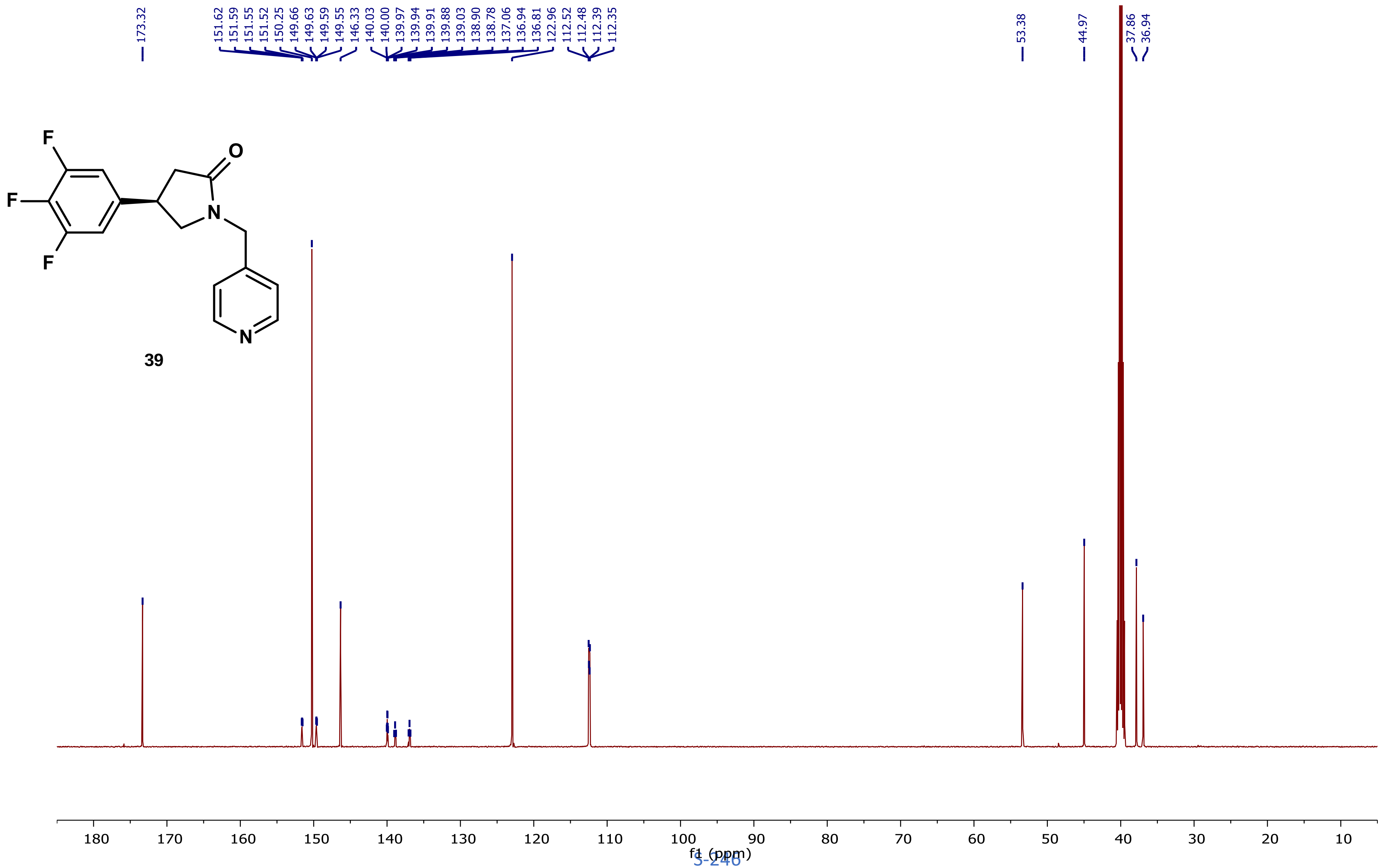
173.32
151.62
151.59
151.55
151.52
150.25
149.66
149.63
149.59
149.55
146.33
140.03
140.00
139.97
139.94
139.91
139.88
139.03
138.90
138.78
137.06
136.94
136.81
122.96
112.52
112.48
112.39
112.35
53.38
44.97
37.86
36.94
180 170 160 150 140 130 120 110 100 90 80 70 60 50 40 30 20 10
f1 (ppm)

39

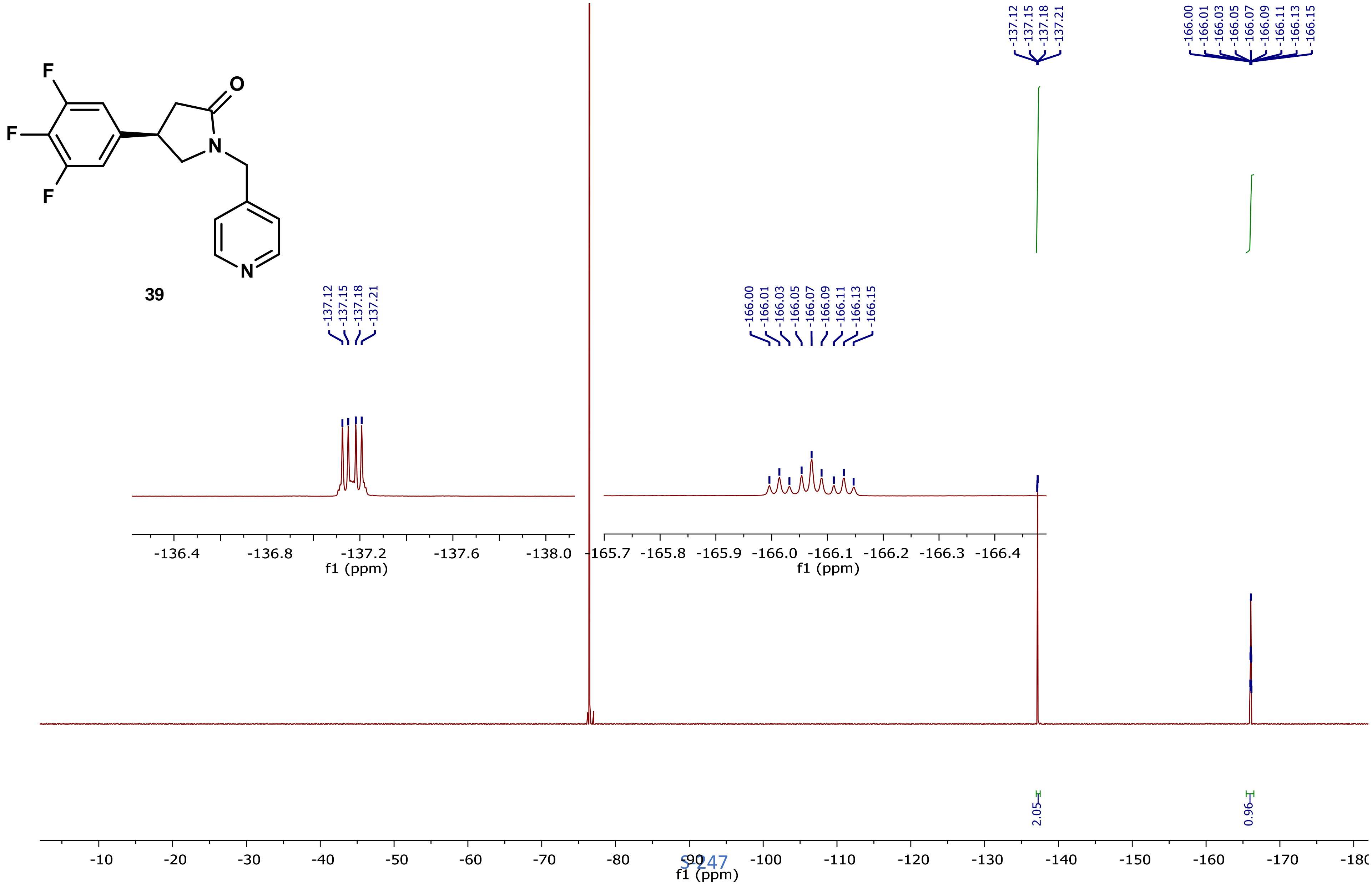
39
-137.12
-137.15
-137.18
-137.21
-166.00
-166.01
-166.03
-166.05
-166.07
-166.09
-166.11
-166.13
-166.15
-136.4 -136.8 -137.2 -137.6 -138.0
f1 (ppm)
-165.7 -165.8 -165.9 -166.0 -166.1 -166.2 -166.3 -166.4
f1 (ppm)
2.05
0.96
-10 -20 -30 -40 -50 -60 -70 -80 -90 -100 -110 -120 -130 -140 -150 -160 -170 -180
f1 (ppm)
